# Convergent Concordant Mode Approach for Molecular Vibrations: CMA-2

**DOI:** 10.1021/acs.jctc.4c01240

**Published:** 2024-12-13

**Authors:** Nathaniel L. Kitzmiller, Mitchell E. Lahm, Laura N. Olive Dornshuld, Jincan Jin, Wesley D. Allen, Henry F. Schaefer III

**Affiliations:** † Center for Computational Quantum Chemistry and Department of Chemistry, University of Georgia, Athens, Georgia 30602, United States; ‡ Allen Heritage Foundation, Dickson, Tennessee 37055, United States; § Indiana Wesleyan University, Marion, Indiana 46953, United States

## Abstract

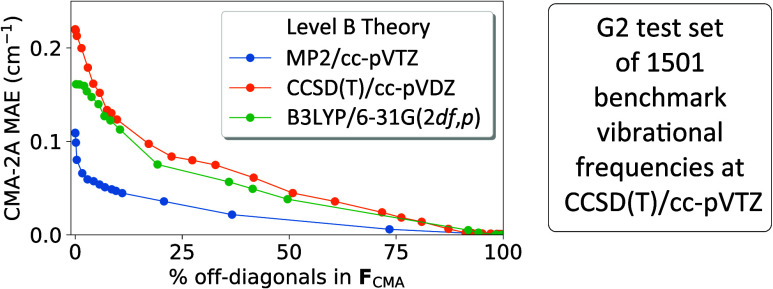

The concordant mode approach (CMA) is a promising new scheme for dramatically increasing the system size and level of theory achievable in quantum chemical computations of molecular vibrational frequencies. Here, we achieve advances in the CMA hierarchy by computations targeting CCSD(T)/cc-pVTZ (coupled cluster singles and doubles with perturbative triples using a correlation-consistent polarized-valence triple-ζ basis set) benchmarks within the G2 molecular test set, executing a statistical analysis for 1501 frequencies from 111 compounds and then separately solving the refractory case of pyridine. First, MP2/cc-pVTZ (second-order Møller–Plesset perturbation theory with the same basis set) proves to be an excellent and preferred choice for generating the underlying (Level B) normal modes of the CMA scheme. Utilizing this Level B within the CMA-0A method reproduces the 1501 benchmark frequencies with a mean absolute error (MAE) of only 0.11 cm^–1^ and an attendant standard deviation of 0.49 cm^–1^. Second, a convergent CMA-2 method is constituted that allows efficient computation of higher level (Level A) frequencies to any reasonable accuracy threshold by using only Hartree–Fock (HF) and MP2 or density functional theory (DFT) data to generate ξ parameters, which select the sparse off-diagonal force field elements for explicit evaluation at Level A. When Level B = MP2/cc-pVTZ, a cutoff of ξ = 0.02 provides an average maximum absolute error per molecule of only 0.17 cm^–1^ by incurring merely a 33% increase in average cost over CMA-0A. This CMA-2 method also eradicates the 4 problematic CMA-0A outliers of pyridine with even less effort (ξ = 0.04, 22% increase). Finally, the newly developed CMA procedures are shown to be highly successful when applied to 1-(1*H*-pyrrol-3-yl)ethanol, a new test molecule with diverse types of vibration.

## 1 Introduction

The force field for molecular vibrations is an indispensable tool for contemporary quantum chemistry, facilitating investigations on molecular structure, spectroscopy, and thermochemistry. Even after decades of research, transformative advances in the computation of force fields are still ongoing.^1^ For highly accurate *ab initio* quantum chemical methods such as coupled cluster singles and doubles with perturbative triples [CCSD(T)], computing the vibrational Hessian fully analytically^2−4^ is possible for small systems but rapidly becomes intractable for larger molecules. Alternatively, one can numerically compute the Hessian using finite differences of analytic gradients or single-point energies, coarsely parallelizing the task. In such schemes, numerical differentiation is less resource-demanding and a necessity for theoretical methods for which analytical gradients or Hessians have not been implemented, let alone derived.

The number of single-point energies required to compute the force constants in a Hessian for a polyatomic molecule scales quadratically with the number of atoms in the molecule. Computations of CCSD(T)/cc-pVTZ quadratic force constants for systems with up to 500 basis functions and 75 vibrational degrees of freedom are still currently feasible in our laboratory, but the inherent scaling causes the full process to rapidly become intractable as the size of the system increases further. Rectilinear Cartesian displacement coordinates are often used as a basis for computing the force constants numerically; however, this set of coordinates is not optimal for describing molecular motion on inherently curvilinear potential energy surfaces. Employing intuitive curvilinear internal coordinates as a basis reduces the coupling encountered with rectilinear coordinates, and a litany of research exists on this topic.^5−21^ The Natural Internal Coordinates (NICs) of Pulay and co-workers are a trenchant choice for describing molecular vibrations.^22,23^

NICs are linear combinations of chemically motivated internal coordinates, and algorithms exist for their automatic generation.^23^ While NICs are a felicitous choice to represent the force field, it is difficult to know *a priori* which force constants are negligible. The subject of vibrational coordinate optimization for the potential energy surface beyond the harmonic approximation is well-researched.^24−26^ The optimal coordinates for the harmonic oscillator are the normal modes of vibration, which simultaneously diagonalize both the quadratic kinetic and potential energy matrices. Accordingly, to maximize the sparsity of the force constant matrix, the coordinates selected should target the normal mode basis.

A breakthrough solution for selecting vibrational coordinates is the recently developed Concordant Mode Approach (CMA),^1^ whose protocol and notation scheme are fully specified in Section 3 below. The CMA-0A method is a highly accurate starting point centered on the computation of only diagonal force constants at the higher level theory A in a normal mode basis generated by a lower-level theory B. The remarkable accuracy of CMA-0A is demonstrated in the 1580 targeted CCSD(T)/cc-pVTZ benchmark vibrational frequencies of the G2 test set.^27^ Utilizing NICs to represent the normal modes, out of the 1580 cases CMA-0A is successful in reducing all but three and seven frequency residuals to less than 2.5 cm^–1^ for the Level B choices B3LYP/6-31G(2*df*,*p*) and CCSD(T)/cc-pVDZ, respectively. Simultaneously, CMA-0A reduces computational times by a factor of 7–10. A question not addressed in the initial study^1^ is the choice of preferred Level B theory in the CMA protocol. Therefore, the first section of the present research investigates the overall performance of various Level B methods and makes some explicit recommendations.

The second section of this report focuses on the systematic convergence of the CMA methodology by an astute selection of limited off-diagonal force constants for explicit evaluation at Level A. Our introductory paper^1^ formulated a CMA-1 method, where one hand-selects off-diagonal force constants, neglected in CMA-0, to eliminate frequency residuals. When a single off-diagonal force constant per molecule is introduced within CMA-1A(1)[A = CCSD(T)/cc-pVTZ, B = CCSD(T)/cc-pVDZ], all original CMA-0A outlier residuals of the G2 test set are reduced to below 1.2 cm^–1^. While the aforementioned CMA-1A scheme is impressive, it is not intended to be a generally practical method, because the off-diagonal couplings were judiciously selected with full knowledge of the target Level A force constant matrix. Here we propose CMA-2, an *a priori* method of selecting off-diagonal force constants to manage cost and accuracy. An auxiliary force field is obtained with a method henceforth denoted as Level C at little or no additional cost in the Level B computation. In this paper, we investigate Hartree–Fock (HF) theory as Level C. The Level C force field is transformed into the Level B normal mode basis and then cast into a dimensionless matrix **ξ**. The matrix elements ξ_*ij*_ above a given threshold correspond to the Level A off-diagonal force constants to be explicitly computed in the CMA basis.

## 2 Computational Methods

The reference geometries optimized at the CCSD(T)/cc-pVTZ^28−30^ level of theory, their corresponding quadratic force constants, and the NICs chosen in our first CMA study^1^ were also used in the present benchmark research on the G2 test set of molecules. In addition, the CCSD(T)/cc-pVDZ^28−30^ and B3LYP/6-31G(2*df*,*p*)^31−36^ force constants which were previously computed^1^ on top of the CCSD(T)/cc-pVTZ optimized geometries were once again employed with the caveat that symmetry was strictly enforced for all molecules, contrary to the first study where symmetry was only rigorously enforced for molecules belonging to non-Abelian symmetries. Normal modes of vibration that are the sole inhabitants of an irreducible representation will not mix with any other mode and are obtained exactly by all CMA methods. Such modes were retained in the statistical treatment of reference (1) but were omitted here in order improve measures of the efficacy of our CMA methods. For reasons discussed later in this work, pyridine is also omitted from our statistics and analyzed in greater detail in-text, reducing the number of frequencies considered in the G2 test set statistics to 1501. New force fields at the reference geometries were computed with CCSD, MP2,^38^ density-fitted MP2 (df-MP2),^39,40^ HF, and density-fitted HF (df-HF) electronic wave function methods, also complying with the full symmetry for all molecules.

As before,^1^ the cc-pV*X*Z (*X* = D, T) basis sets^41,42^ were employed for H and Li-F, while the corresponding cc-pV(*X*+*d*)Z basis sets^43^ were chosen for Na-Cl in order to incorporate the flexibility of tight *d*-functions. For brevity, the +*d* designations are assumed but not listed in the basis set notations used here. The Level B quadratic force constants were computed in the NIC basis via finite differences with fourth-order accuracy, and the corresponding vibrational normal modes were computed with the GF-matrix method.^44^ A displacement size of 0.01 or 0.005 in the units of the NICs was employed throughout.^44^ The Level A reference force constants were transformed from the Cartesian basis to the normal mode basis of Level B to obtain the matrix **F**_CMA_. In practical applications, the desired elements of **F**_CMA_ would be computed by finite differences of Level A energies. However, to completely eliminate numerical errors in the current benchmark research, we directly obtained CMA frequencies by zeroing out off-diagonal elements of **F**_CMA_ according to the CMA protocol being employed. The Molpro^45^ program was used to compute any conventional CC, MP2, or HF energies, and the SCF and CC energy residuals were reduced to 10^–12^ Hartrees upon convergence. The df-MP2 and df-HF results were computed with Psi4,^46^ and the SCF energy residuals were converged to 10^–10^ Hartrees in these cases. Core orbitals were frozen in all coupled cluster and MP2 computations. The optimized geometries and vibrational frequencies of the CCSD(T)/cc-pVTZ target theory, as well as the NICs employed, are compiled in the Supporting Information of this work.

## 3 CMA Protocol

The most general CMA protocol^1^ can be summarized as follows:

1. Choose a complete (preferably chemically intuitive) set of nonredundant internal coordinates **S**.
2. Optimize the molecular geometry for computing the higher level theory A vibrational frequencies and for constructing the concordant modes at a lower-level of theory B, as denoted by CMA[Level A, Level B].
3. Solve the **GF**_B_ eigenproblem^44^ after obtaining the lower-level force constants **F**_B_. The nonorthogonal **L**_B_ eigenvector tensor yields the concordant normal modes (**Q**_B_) according to **S** = **L**_B_**Q**_B_.
4. Adopt the ansatz **F**_A_ = (**L**_B_^–1^)^T^**F**_CMA_(A)**L**_B_^–1^, where **F**_A_ and **F**_CMA_(A) are the force constants at higher level theory A in the **S** and **Q**_B_ basis sets, respectively. Minimally, the elements to be included in **F**_CMA_(A) are the diagonal elements, whereas the transformation becomes exact if all elements are included.
5. Adopt a protocol (CMA-*N*) that specifies which elements of **F**_CMA_(A) to explicitly compute via finite differences at the higher level of theory; the CMA-0, CMA-1, and CMA-2 variants discussed below are those currently available. A chosen displacement δ**Q**_B_ can be mapped into a final set of Cartesian coordinates by iterative application of the linear relationship δ**x** = u**B**_A_^T^**G**_A_^–1^**L**_B_(δ**Q**_B_), where **B**_A_ is the customary B matrix for the **S** coordinates at the A reference geometry and **u** is a diagonal matrix of reciprocal atomic masses.^47,48^
6. Transform the resulting **F**_CMA_(A) to **F**_A_, and then solve the **GF**_A_ eigenproblem to find the higher level A harmonic vibrational frequencies and corresponding normal modes.

In the CMA protocols labeled as (CMA-*N*A, CMA-*N*B), the Level B force constants are computed on top of the (Level A, Level B) optimum geometries. When necessary for clarity, the various levels of theory are appended in brackets to the end of the overarching protocol, such as CMA-0A[Level A, Level B] or CMA-2A[Level A, Level B, Level C]. All CMA variants require the diagonal elements of **F**_CMA_(A) to be included, and the CMA-0A and CMA-0B variants include only these. In our first study,^1^ CMA-0A was found to be more accurate than CMA-0B, since the underlying reference geometry is essential to the accurate prediction of second- and higher-order force constants.^47^ The CMA-1(*n*) variant only differs from CMA-0 in that *n* hand-selected off-diagonal force constants are also chosen to be computed along with the diagonals. In the convergent CMA-2 method, the dimensionless matrix **ξ** is constructed via eq 1 from the Level C force field transformed to the **Q**_B_ normal mode basis [**F**_CMA_(C)]:
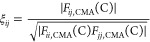
1In carrying out step 5 of the general protocol for the CMA-2 case, all matrix elements ξ_*ij*_ greater than a user-given threshold correspond to matrix elements of **F**_CMA_(A) that will be explicitly computed.

## 4 Benchmarking Level B for CMA-0A

The CMA-0A procedure was executed for eight Level B theories, as summarized in Table 1. The deviation of the pure Level B results from the CCSD(T)/cc-pVTZ target is generally reduced by at least an order of magnitude for every summary statistic when CMA-0A is applied. The mean absolute error (MAE) is the same statistic designated as the mean absolute deviation (MAD) in our prior work;^1^ MAE is used here to avoid confusion with common statistical definitions of MAD. The MAE of the CMA-0A residuals ranges from 0.11 to 1.09 cm^–1^ for all Level B choices tested. The mean CMA residuals fall within a remarkably narrow range of 0.02 to 0.14 cm^–1^, and the standard deviation (σ_ϵ_) ranges from 0.36 to 4.93 cm^–1^. The maximum absolute error (ϵ_MAX_) over the entire G2 test set is between 3.4 and 11.6 cm^–1^ for all CMA computations with correlated Level B theories, whereas Hartree–Fock proves to perform much more poorly in this regard. The MAEs for the zero-point vibrational energy (ZPVE) residuals (Δ) are a remarkably tight group of minuscule values across the Level B spectrum, ranging from 0.11 to 0.95 cm^–1^; similarly, the standard deviation (σ_Δ_) of the ZPVE Δ lies between 0.24 and 1.56 cm^–1^. As observed earlier^1^ a beneficial cancellation of individual frequency errors provides exceptional accuracy for the CMA-0A ZPVE values.

**Table 1:** Summary Statistics within the G2 Test Set^a^ of CMA-0A Residuals for CCSD(T)/cc-pVTZ Harmonic Vibrational Frequencies (ϵ, cm^–1^) and ZPVEs (Δ, cm^–1^) as Compared to the Corresponding Pure Level B Errors

Level B		MAE ϵ	mean ϵ	ϵ_max_^b^	σ_ϵ_	ϵ_MAX_^c^	MAE Δ	mean Δ	σ_Δ_
MP2/cc-pVTZ^d^	pure	9.44	5.62	28.2	13.68	145	40.6	38.3	39.1
	CMA	0.11	0.02	0.54	0.49	9.3	0.12	0.12	0.24
df-MP2/cc-pVTZ^d^^,^^e^	pure	9.00	5.19	27.2	12.55	146	37.9	35.6	32.0
	CMA	0.11	0.02	0.53	0.49	9.3	0.11	0.11	0.24
HF/cc-pVTZ	pure	56.92	54.95	94.7	34.41	251	374.4	371.5	294.7
	CMA	1.09	0.14	5.18	4.93	86.3	0.95	0.95	1.56
B3LYP/6-31G(2*df,p*)	pure	8.82	4.66	23.1	10.86	69	36.0	31.5	33.3
	CMA	0.16	0.04	0.59	0.36	3.4	0.27	0.27	0.32
CCSD(T)/cc-pVDZ	pure	47.54	47.12	120.0	54.46	173	319.0	318.6	244.6
	CMA	0.22	0.04	0.74	0.45	4.0	0.27	0.27	0.29
CCSD/cc-pVDZ	pure	54.34	54.05	128.2	53.55	165	365.5	365.5	267.4
	CMA	0.23	0.04	0.77	0.46	3.6	0.29	0.29	0.29
MP2/cc-pVDZ^d^	pure	52.52	52.15	139.4	60.38	213	355.3	355.3	253.4
	CMA	0.32	0.06	1.18	0.83	11.6	0.38	0.38	0.41
HF/cc-pVDZ	pure	85.11	83.88	132.8	47.55	247	567.8	567.1	440.4
	CMA	0.72	0.12	3.45	3.02	58.4	0.81	0.81	1.17

a The instructive case of pyridine is omitted from these statistics and dissected in a later section, leaving 1501 frequencies in the data set.

b Average maximum absolute ϵ per molecule.

c Maximum absolute ϵ over the entire data set.

d Excluding ω_3_(O_3_), for which MP2 is a catastrophic failure.^49^

e Excluding ω_3_(NO_2_), for which df-MP2 in Psi4 does not run successfully.

For a deeper analysis, we first focus on the CMA-0A results in which a cc-pVDZ basis set was used for Level B. Both CMA-0A with B = CCSD/cc-pVDZ and B = CCSD(T)/cc-pVDZ perform very well, and there is no significant statistical difference differentiating the two. The CMA-0A MP2/cc-pVDZ statistics are almost as good as the coupled cluster results, making MP2 a promising candidate as a Level B theory which scales as *N*^5^ with basis set size, compared to the *N*^6^ and *N*^7^ scaling of conventional CCSD and CCSD(T), respectively. The CMA HF/cc-pVDZ results do not compare as well, displaying a standard deviation (3.02 cm^–1^) and maximum absolute residual (58.4 cm^–1^) that are considerably larger than the corresponding coupled cluster results. Apparently, even a minimal treatment of electron correlation in Level B is important in CMA computations targeting CCSD(T)/cc-pVTZ frequencies. Nonetheless, HF may still be an apt Level B for obtaining ZPVEs, as its MAE is only 0.81 cm^–1^.

We now turn our attention to the CMA-0A results for which the larger cc-pVTZ and 6-31G(2*df*,*p*) basis sets are used. Selecting Level B = MP2/cc-pVTZ gives the best MAE (0.11 cm^–1^) and mean error (+0.02 cm^–1^). The conventional and density-fitted MP2 results are nearly indistinguishable, a quite promising finding because df-MP2 scales as *N*^4^ with basis set size, compared to the *N*^5^ scaling of conventional MP2. The summary statistics for the HF residuals once again show that electron correlation should be included at Level B, but not necessarily in ZPVE computations. While B3LYP/6-31G(2*df*,*p*) yields excellent CMA-0A results, this DFT method is outperformed by both MP2/cc-pVTZ and df-MP2/cc-pVTZ for the MAE ϵ, mean ϵ, ϵ_max_, and ZPVE statistics. The only caveat is that MP2 and df-MP2 tend to exhibit a few larger maximum absolute residuals; for example, in six out of 1501 cases this quantity is greater than 4 cm^–1^ for MP2/cc-pVTZ. Overall, the summary statistics indicate that basis set quality is more important than higher-order treatment of electron correlation in the selection of Level B. Pending further research, the key conclusion is that if one is targeting CCSD(T)/cc-pV*X*Z frequencies with the CMA-0A approach, then MP2/cc-pV*X*Z or its df-variant is the preferred Level B method.

## 5 Development of CMA-2A Theory

For the purpose of developing a convergent CMA-2 theory, in this section we consider a CMA outlier as any frequency residual greater than 1.5 cm^–1^ in magnitude. Such outliers for CMA-0A[CCSD(T)/cc-pVTZ, MP2/cc-pVTZ] are presented in Table 2, where 16 cases are found from 9 distinct molecules out of the test set of 1501 frequencies. The size of these residuals ranges from 1.56 to 9.34 cm^–1^. Pyridine presented a unique challenge to CMA-0A with four outliers at −22.61, −6.46, 4.87, and 27.60 cm^–1^. Because this molecule proved to be so abnormal, it is excluded from the statistics in Table 2 and treated in greater detail below. When the vibrational modes highlighted in Table 2 are treated by CMA-1A(1) (fifth column), all absolute residuals are less than 1.2 cm^–1^. In other words, the inclusion of a single off-diagonal force constant per molecule is sufficient to make all outliers disappear! This mathematical fact shows that CMA-0A is tantalizingly close to being a flawless method for this limited test set. However, finding the optimal mathematical solution without prior knowledge of the full force constant matrix remains a considerable challenge. There may be circumstances in which chemical principles can effectively guide one to choose the correct mode couplings, but the development of a generally applicable, automated approach is clearly warranted.

**Table 2:** Only Cases within the Set of 1501 Benchmark Frequencies with CMA-0A[CCSD(T)/cc-pVTZ, MP2/cc-pVTZ] Residuals (ϵ, cm^–1^) Greater than 1.5 cm^–1^ in Magnitude, Together with the Corresponding CMA-1A and CMA-2A Results That Target These Outliers

Molecule and mode	Description	Benchmark	ϵ[CMA-0A]	ϵ[CMA-1A(1)]	ϵ[CMA-2A](*n*)^a^	η^b^ (%)
nitrous oxide, ω_3_(σ)	sym. stretch	1297.09	9.34	0.00	0.00 (1)	25
ketene, ω_9_(*b*_2_)	C=C=O bend	514.86	7.12	0.00	0.00 (2)	22
ketene, ω_8_(*b*_2_)	CH_2_ wag	584.48	–6.28	0.00	0.00 (2)	22
nitrous oxide, ω_1_(σ)	antisym. stretch	2282.57	–5.33	0.00	0.00 (1)	25
benzene, ω_18_(*b*_*1u*_)	ring def.	1158.88	4.90	0.00	0.00 (10)	36
benzene, ω_17_(*b*_1u_)	ring def.	1328.17	–4.29	0.00	0.00 (10)	36
spiropentane, ω_4_(*a*_1_)	ring breathing	1054.41	3.67	0.00	0.03 (7)	21
spiropentane, ω_3_(*a*_1_)	sym. CH_2_ wag	1076.02	–3.60	0.01	0.01 (7)	21
methyl nitrite, ω_12_(*a*)	N–O stretch	595.01	3.55	0.68	–0.12 (9)	60
nitromethane, ω_4_(*a*)	NO_2_ rock	1642.57	–2.87	–1.11	0.04 (13)	87
nitromethane, ω_10_(*a*)	CH_3_ rock	1110.76	2.71	0.12	0.12 (13)	87
*n*–butane, ω_28_(*b*_*u*_)	CH_3_ stretch	3028.05	1.81	0.00	1.81 (10)	28
*n*–butane, ω_29_(*b*_*u*_)	CH_2_ stretch	3023.91	–1.79	–0.03	–1.79 (10)	28
aziridine, ω_1_(*a*′)	CH_2_ rock	783.89	1.76	1.19	1.11 (12)	67
isobutane, ω_3_(*a*_1_)	CH stretch	3019.64	1.62	0.02	1.62 (5)	14
isobutane, ω_2_(*a*_1_)	CH_3_ sym. stretch	3024.21	–1.56	0.04	–1.56 (5)	14

a *n* = the number of off-diagonal elements included for the ξ = 0.020 cutoff.

b η = number of **F**_CMA_(A) off-diagonal elements included as a percentage of the vibrational degrees of freedom for the given molecule.

An algorithm for automatically selecting off-diagonal force constants should have a variable tolerance so that the user has ultimate control over the cost and accuracy of a convergent CMA protocol. A diagnostic can be imagined with matrix elements that correctly predict the importance of off-diagonal force constants of **F**_CMA_(A). A simple approach would be to compute another set of force constants at a level of theory C in the **Q**_B_ normal mode basis, such that the magnitude of **F**_CMA_(C) off-diagonal force constants mimics the ordering of the **F**_CMA_(A) elements. Preferably, this third Level C should scale better than theory B, and the ideal circumstance would not incur any additional computational cost. If B = MP2/cc-pV*X*Z single-point energies are computed to target A = CCSD(T)/cc-pV*X*Z frequencies using CMA, then the C = HF/cc-pV*X*Z quadratic force field can indeed be obtained without extra cost.

With this choice, the level of theory manifestly increases in the sequence C → B → A. Because **F**_CMA_(B) is a strictly diagonal matrix by construction, then the **F**_CMA_(C) off-diagonal elements of greatest size directly indicate which couplings in the molecule are most sensitive to the C → B increase in level of theory. These are precisely the couplings that are expected to yield significant nonzero values for the off-diagonal elements of **F**_CMA_(A) as a consequence of the B → A change in level of theory. In this sense, **F**_CMA_(C) can be considered a photographic negative of **F**_CMA_(A), although this characterization is not meant to imply that the coupling elements in these matrices are always opposite in sign. In order to translate this correspondence into a viable, universal diagnostic for identifying which parts of **F**_CMA_(A) should be explicitly computed, the **F**_CMA_(C) off-diagonal elements should be cast into a dimensionless form that also takes into account their size relative to the associated diagonal force constants. For this purpose we have adopted eq 1 to define the ξ_*ij*_ diagnostics for our CMA-2 theory.

For the remainder of the study, when Level B is a correlated wave function method such as CCSD or MP2 with a cc-pV*X*Z basis set, then Level C is chosen as HF/cc-pV*X*Z so that C → B → A forms a series of increasing electron correlation with a fixed basis set. When B = B3LYP/6-31G(2*df*,*p*), we have employed C = HF/6-31G(2*df*,*p*) for lack of a more transparent choice, even though extra cost is incurred because a second set of SCF orbitals must be optimized. The CMA-2A protocol that incorporates all **F**_CMA_ force constants corresponding to ξ > 0.020 is assessed in Table 2. Overall, 12 of the 16 outliers are successfully eliminated by CMA-2A (|ϵ| < 1.11 cm^–1^). The remaining four residuals are hardly significant, corresponding to the (1.81, −1.79) and (1.62, −1.56) cm^–1^ pairs for (ω_28_, ω_29_) and (ω_3_, ω_2_) of *n*-butane and isobutane, respectively. For the (*n*-butane, isobutane) C–H stretching modes, the ξ cutoff identified (10, 5) off-diagonal elements for explicit evaluation at Level A, but the key coupling discovered in CMA-1A(1) that eliminates these outliers escaped inclusion. The η parameter appearing in Table 2 is equal to the number of included off-diagonal **F**_CMA_ elements as a percentage of the molecular vibrational degrees of freedom that are present. This metric turns out to also be very nearly the percentage increase in computational cost in going beyond CMA-0A to CMA-2A. Demonstrating the success of CMA-2A with a ξ = 0.020 cutoff, 8 of the outliers are eliminated with η < 37%, and in the remaining instances η = 60–87%. Given that CMA-0A already reduces the cost of Level A frequency computations by 700–1000%,^1^ any η value less than 100% still constitutes a rather marginal price to pay for the added certainty afforded by CMA-2A.

**Figure 1:**
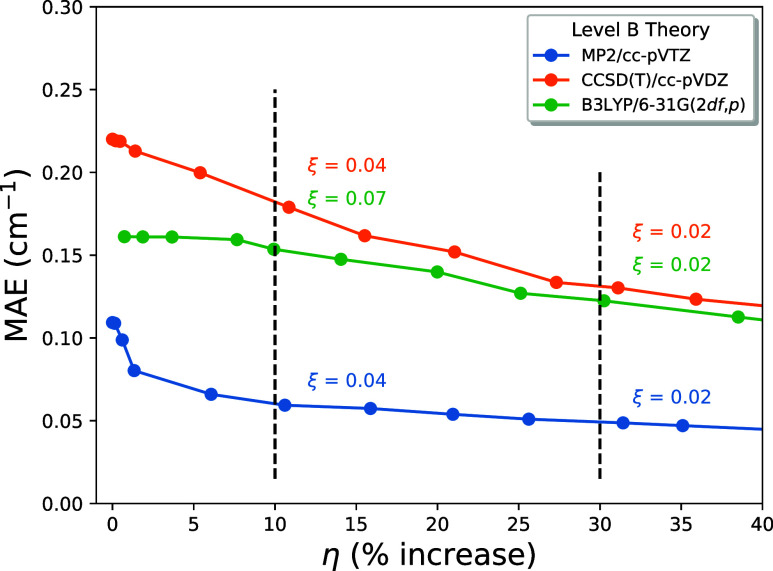
CMA-2A MAE of the 1501 CCSD(T)/cc-pVTZ benchmark frequencies plotted as a function of η, where Level C = HF and the basis set is the same as employed in Level B.

The performance of CMA-2A for the entire data set of CCSD(T)/cc-pVTZ benchmark frequencies is illustrated in Figure 1, where the MAE is plotted vs η. It is abundantly clear from the curves that MP2/cc-pVTZ (blue) is once again superior to both CCSD(T)/cc-pVDZ (orange) and B3LYP/6-31G(2*df*,*p*) (green); hence, B = MP2/cc-pVTZ is the method of choice for both CMA-0A and CMA-2A. In this case an increase in cost of only 10% is sufficient to lower the MAE to a mere 0.06 cm^–1^! The corresponding blue curve has an underlying ξ = 0.040 cutoff at η = 10%, and any further reduction of ξ improves the MAE very little. In contrast, the B = CCSD(T)/cc-pVDZ curve reveals a steady MAE reduction as η ranges up to 40% and as the ξ cutoff is diminished to 0.020. While not optimal, this choice of Level B still provides excellent final results once CMA-2A is applied. Although B = B3LYP/6-31G(2*df*,*p*) achieves much success in CMA-0A, the lag in further improvement via CMA-2A is somewhat disappointing, presumably because the C → B → A series is not systematic in this circumstance.

**Figure 2:**
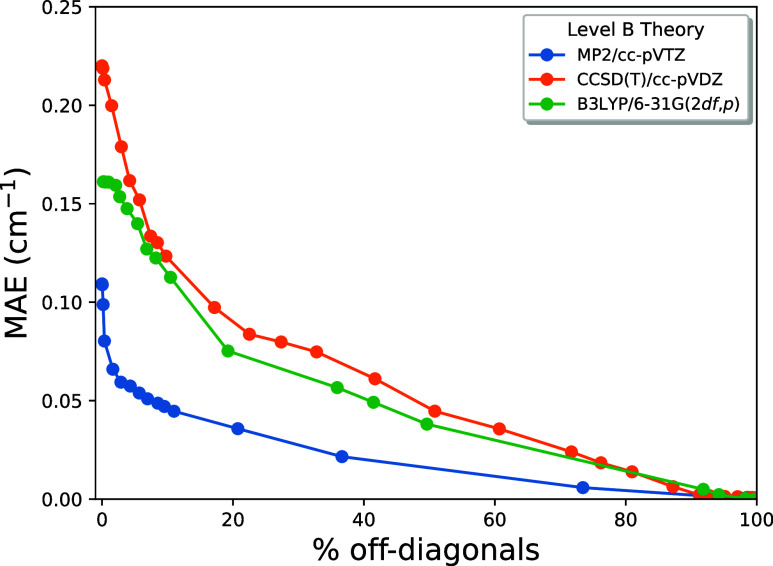
CMA-2A MAE of the 1501 CCSD(T)/cc-pVTZ benchmark frequencies plotted as a function of % nonzero off-diagonal matrix elements included in **F**_CMA_(A).

It is worth emphasizing that the maximum value of η in Figure 1 still corresponds to a very small fraction of off-diagonal elements included in **F**_CMA_. In order to reveal the asymptotic nature of CMA-2A, the MAE over the entire data set is plotted against the percent of included nonzero **F**_CMA_ off-diagonals in Figure 2. Of course, all the MAE curves must decay to zero when 100% of the off-diagonals are explicitly computed. For all intents and purposes, this asymptotic value is reached at the 90% mark on the abscissa, regardless of the choice of Level B. The key point of Figure 2 is that CMA-2A is truly a convergent method, and the smooth and rapid decay of the blue curve for B = MP2/cc-pVTZ shows how striking the performance can be. Statistics for the overall performance of CMA-2A on the benchmark frequencies are collected in Table 3, where MAE ϵ, σ_ϵ_, ϵ_max_, ϵ_MAX_, and η are given as the ξ cutoff ranges from 0.005 to ∞ and as Level B is varied. These data allow the CMA-2A user to make a wise choice for ξ and Level B in accord with the accuracy goals and computational costs of the chemical application at hand. A salient feature of Table 3 is that the MAE, standard deviation, and largest residual per molecule all converge much more rapidly as the ξ cutoff is made more stringent when B = MP2/cc-pVTZ as compared to B = CCSD(T)/cc-pVDZ or B3LYP/6-31G(2*df*,*p*). This observation further amplifies the conclusions from Figures 1 and 2 on the selection of Level B. Because MAE ϵ, σ_ϵ_, and ϵ_max_ are already much less than 1 cm^–1^ when no off-diagonal elements of **F**_CMA_ are accounted for (ξ cutoff = ∞), CMA-0A will be more than sufficient in a preponderance of applications. However, a very low probability remains that an isolated frequency error might occur on the order of ϵ_MAX_. Informed by Table 3, the user can apply CMA-2A to eliminate this concern, while still maintaining vast savings in computing the harmonic frequencies of the molecule at higher level A. For example, application of CMA-2A with our recommended Level B = MP2/cc-pVTZ and a ξ cutoff of 0.02 yields truly outstanding accuracy while only increasing the average cost by 33% over CMA-0A. The convincing residual statistics in this case can be spelled out as follows: mean absolute error = 0.047 cm^–1^, average maximum absolute residual per molecule = 0.17 cm^–1^, standard deviation of residuals = 0.15 cm^–1^, and global maximum absolute residual = 1.81 cm^–1^.

**Table 3:** Metrics for CMA-2A Performance of the 1501 CCSD(T)/cc-pVTZ Benchmark Frequencies as a Function of the ξ Cutoff and the Choice of Level B^a^

	B = MP2/cc-pVTZ					B = CCSD(T)/cc-pVDZ					B = B3LYP/6-31G(2*df,p*)				
ξ Cutoff	MAE ϵ	ϵ_max_	σ_ϵ_	ϵ_MAX_	η (%)	MAE ϵ	ϵ_max_	σ_ϵ_	ϵ_MAX_	η (%)	MAE ϵ	ϵ_max_	σ_ϵ_	ϵ_MAX_	η (%)
∞	0.11	0.54	0.49	9.34	0	0.22	0.74	0.45	3.96	0	0.16	0.59	0.36	3.42	0
0.20	0.099	0.45	0.40	7.12	0.6	0.22	0.73	0.45	3.96	0.5	0.16	0.58	0.36	3.42	7.7
0.18	0.099	0.45	0.40	7.12	0.7	0.22	0.73	0.45	3.96	0.5	0.16	0.58	0.36	3.42	7.8
0.16	0.093	0.40	0.37	7.12	0.8	0.22	0.73	0.45	3.96	0.6	0.16	0.58	0.36	3.42	7.9
0.14	0.093	0.40	0.37	7.12	0.8	0.22	0.73	0.45	3.96	0.9	0.16	0.58	0.35	3.42	8.1
0.12	0.081	0.33	0.25	3.67	1.0	0.22	0.72	0.45	3.96	1.0	0.16	0.58	0.35	3.42	8.4
0.10	0.080	0.32	0.25	3.67	1.3	0.21	0.72	0.44	3.96	1.4	0.16	0.56	0.35	3.42	8.8
0.08	0.079	0.32	0.25	3.67	1.9	0.21	0.73	0.44	3.96	1.8	0.15	0.55	0.35	3.42	9.3
0.06	0.070	0.27	0.21	3.55	4.3	0.21	0.71	0.44	3.96	3.1	0.15	0.55	0.35	3.42	11
0.04	0.059	0.22	0.18	2.78	11	0.19	0.62	0.39	2.87	8.2	0.15	0.53	0.34	3.42	14
0.02	0.047	0.17	0.15	1.81	33	0.13	0.40	0.28	2.56	29	0.12	0.39	0.28	2.77	33
0.01	0.036	0.14	0.13	1.81	76	0.10	0.29	0.23	2.66	63	0.08	0.25	0.20	2.77	71
0.005	0.022	0.10	0.11	1.81	135	0.07	0.24	0.20	2.68	121	0.06	0.20	0.18	2.77	132

a See footnotes to Tables 1 and 2 for definitions of the metrics. Level C = HF with the same basis used for Level B.

## 6 Solution of the Pyridine Problem

**Figure 3:**
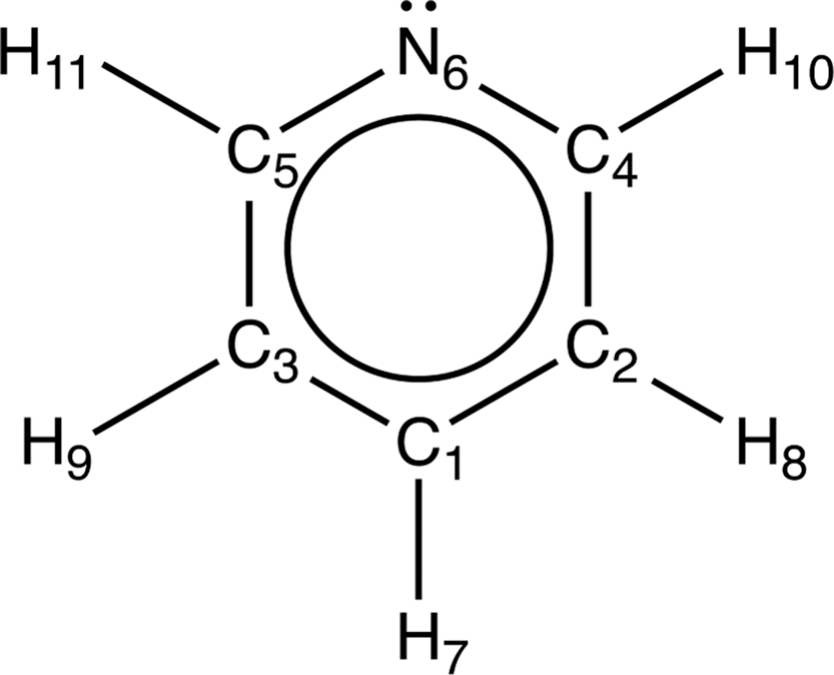
Enumerated carbon backbone of pyridine, corresponding to selected internal coordinates utilized for all CMA computations.

The unusual behavior of pyridine in CMA applications demands that this molecule be analyzed in greater detail as an instructive, isolated benchmark species rather than as an outlier that excessively skews the statistics of the G2 test set. Pyridine is depicted in Figure 3 with atomic labeling, and corresponding NICs for our CMA analyses are defined in Table 4. The ring coordinates are constructed with the 6-fold symmetry of a regular hexagon, but the CMA frequencies would be mathematically invariant if linear combinations were made only with the *C*_2v_ symmetry of pyridine itself.

**Table 4:** Pyridine Natural Internal Coordinates for CMA Applications

Description	Unnormalized natural internal coordinate^a^
ring breathing	*S*_1_(*a*_1_) = *r*(5,3) + *r*(3,1) + *r*(1,2) + *r*(2,4) + *r*(4,6) + *r*(6,5)
ring stretching def.	*S*_2_(*b*_2_) = *r*(5,3) – *r*(3,1) + *r*(1,2) – *r*(2,4) + *r*(4,6) – *r*(6,5)
ring stretching def.	*S*_3_(*a*_1_) = 2*r*(5,3) – *r*(3,1) – *r*(1,2) + 2*r*(2,4) – *r*(4,6) – *r*(6,5)
ring stretching def.	*S*_4_(*b*_2_) = 2*r*(5,3) + *r*(3,1) – *r*(1,2) – 2*r*(2,4) – *r*(4,6) + *r*(6,5)
ring stretching def.	*S*_5_(*a*_1_) = *r*(3,1) + *r*(1,2) – *r*(4,6) – *r*(6,5)
ring stretching def.	*S*_6_(*b*_2_) = *r*(3,1) – *r*(1,2) + *r*(4,6) – *r*(6,5)
sym. CH stretch	*S*_7_(*a*_1_) = *r*(5,11) + *r*(4,10)
antisym. CH stretch	*S*_8_(*b*_2_) = *r*(5,11) – *r*(4,10)
sym. CH stretch	*S*_9_(*a*_1_) = *r*(3,9) + *r*(2,8)
antisym. CH stretch	*S*_10_(*b*_2_) = *r*(3,9) – *r*(2,8)
CH stretch	*S*_11_(*a*_1_) = *r*(1,7)
ring stellation	*S*_12_(*a*_1_) = θ(4,6,5) – θ(6,5,3) + θ(5,3,1) – θ(3,1,2) + θ(1,2,4) – θ(2,4,6)
ring rectangulation	*S*_13_(*a*_1_) = 2θ(4,6,5) – θ(6,5,3) – θ(5,3,1) + 2θ(3,1,2) – θ(1,2,4) – θ(2,4,6)
ring shearing	*S*_14_(*b*_2_) = θ(6,5,3) – θ(5,3,1) + θ(1,2,4) – θ(2,4,6)
sym. i.p. CH rock	*S*_15_(*a*_1_) = θ(11,5,6) – θ(11,5,3) + θ(10,4,6) – θ(10,4,2)
antisym. i.p. CH rock	*S*_16_(*b*_2_) = θ(11,5,6) – θ(11,5,3) – θ(10,4,6) + θ(10,4,2)
sym. i.p. CH rock	*S*_17_(*a*_1_) = θ(9,3,5) – θ(9,3,1) + θ(8,2,4) – θ(8,2,1)
antisym. i.p. CH rock	*S*_18_(*b*_2_) = θ(9,3,5) – θ(9,3,1) – θ(8,2,4) + θ(8,2,1)
i.p. CH rock	*S*_19_(*b*_2_) = θ(7,1,2) – θ(7,1,3)
chair ring pucker	*S*_20_(*b*_1_) = τ(6,5,3,1) + τ(3,1,2,4) – τ(5,3,1,2) – τ(1,2,4,6) + τ(2,4,6,5) – τ(4,6,5,3)
boat ring pucker	*S*_21_(*b*_1_) = τ(3,1,2,4) – τ(5,3,1,2) – τ(2,4,6,5) + τ(4,6,5,3)
ring twist	*S*_22_(*a*_2_) = 2τ(6,5,3,1) – τ(5,3,1,2) – τ(3,1,2,4) + 2τ(1,2,4,6) – τ(2,4,6,5) – τ(4,6,5,3)
sym. o.o.p. CH wag	*S*_23_(*b*_1_) = γ(11,5,3,6) + γ(10,4,6,2)
antisym. o.o.p. CH wag	*S*_24_(*a*_2_) = γ(11,5,3,6) – γ(10,4,6,2)
sym. o.o.p. CH wag	*S*_25_(*b*_1_) = γ(9,3,1,5) + γ(8,2,4,1)
antisym. o.o.p. CH wag	*S*_26_(*a*_2_) = γ(9,3,1,5) – γ(8,2,4,1)
o.o.p. CH wag	*S*_27_(*b*_1_) = γ(7,1,2,3)

a *r*(*i*,*j*) = *i*–*j* bond distance; θ(*i*,*j*,*k*) = *i*–*j*–*k* bond angle; τ(*i*,*j*,*k*,*l*) = dihedral angle between *i*–*j*–*k* and *j*–*k*–*l* plane; γ(*i*,*j*,*k*,*l*) = signed angle of *i*–*j* bond out of the *k*–*j*–*l* plane.

Comprehensive results for pyridine frequencies are listed in Table 5, where residuals for all modes with respect to the CCSD(T)/cc-pVTZ reference values are provided for seven different treatments: CMA-0A with B = CCSD(T)/cc-pVDZ, MP2/cc-pVTZ, B3LYP/6-31G(2*df*,*p*), CCSD/cc-pVTZ, and MP4/cc-pVTZ, as well as CMA-2A with B = MP2/cc-pVTZ (ξ = 0.04) and CCSD(T)/cc-pVDZ (ξ = 0.015). First we focus on the CMA-0A outlier residuals greater than 1.5 cm^–1^ in magnitude that are highlighted in boldface in Table 5. With B = CCSD(T)/cc-pVDZ, five such residuals appear of modest size, (ϵ_8_, ϵ_9_, ϵ_16_, ϵ_17_, ϵ_25_) = (−3.24, 4.61, −1.58, 2.46, 2.50) cm^–1^. With B = MP2/cc-pVTZ, a clustered set of four much larger residuals crops up, (ϵ_22_, ϵ_23_, ϵ_24_, ϵ_25_) = (−6.46, −22.61, 27.60, 4.87) cm^–1^, despite the fact this method is the clear overall champion for CMA applications within the G2 test set. One infers that both orbital basis set and electron correlation effects are unusually important for the normal modes of pyridine, because there is little overlap among the cases for which these two Level B methods struggle. Quite surprisingly, when B = B3LYP/6-31G(2*df*,*p*) all the CMA-0A residuals (|ϵ| < 0.8 cm^–1^) are well below the outlier threshold, and thus this method happens to capture both the basis set and correlation effects present in the CCSD(T)/cc-pVTZ normal modes of pyridine. While CMA-0A with B = CCSD/cc-pVTZ generally performs better than with B = MP2/cc-pVTZ, the large outliers (ϵ_24_, ϵ_25_) = (−18.15, 19.79) cm^–1^ still remain, the latter of which represents a considerable deterioration. This occurrence suggests that the triple-excitation effects described by the (T) correlation correction are an essential component in the physical description of the normal modes of pyridine. While MP4 is a largely defunct method in modern computational chemistry, correlation terms within this theory inspired the (T) ansatz.^28^ It is thus compelling that the two problematic outliers for B = CCSD/cc-pVTZ are greatly diminished to (ϵ_24_, ϵ_25_) = (−1.56, 2.00) cm^–1^ when B = MP4/cc-pVTZ is invoked.

**Table 5:** CMA-0A and CMA-2A Residuals (ϵ, cm^–1^) for CCSD(T)/cc-pVTZ Harmonic Frequencies (ω_ref_, cm^–1^) of Pyridine Obtained with Various Level B Methods

Mode	Description	ω_ref_	ϵ[CMA-0A]					ϵ[CMA-2A]	
			B = CCSD(T)/cc-pVDZ	B = MP2/cc-pVTZ	B = B3LYP/6-31G(2*df,p*)	B = CCSD/cc-pVTZ	B = MP4/cc-pVTZ	B = CCSD(T)/cc-pVDZ^a^	B = MP2/cc-pVTZ^b^
ω_1_(*a*_1_)	sym. CH str.	3212.85	–0.01	–0.05	–0.03	0.00	0.00	–0.01	–0.06
ω_2_(*a*_1_)	asym. CH str.	3187.57	–0.01	0.01	–0.08	–0.01	0.00	0.03	0.06
ω_3_(*a*_1_)	asym. CH str.	3169.83	0.01	0.05	0.08	0.01	0.01	–0.03	0.02
ω_4_(*a*_1_)	ring str. def.	1630.29	–0.47	–0.21	–0.02	–0.01	–0.06	–0.01	–0.18
ω_5_(*a*_1_)	sym. CH rock	1510.12	–0.48	0.10	–0.05	0.01	0.04	–0.15	0.10
ω_6_(*a*_1_)	asym. CH rock	1236.72	0.50	0.08	0.03	0.00	0.02	–0.02	0.08
ω_7_(*a*_1_)	sym. CH rock	1087.86	–0.37	–0.07	0.00	0.00	–0.06	–0.89	–0.07
ω_8_(*a*_1_)	ring stellation	1043.14	–**3.24**	0.00	–0.16	0.00	–0.02	1.06	0.00
ω_9_(*a*_1_)	ring breathing	1001.24	**4.61**	0.16	0.30	0.01	0.10	0.14	0.17
ω_10_(*a*_1_)	ring rectangle	603.31	0.10	0.02	0.08	0.01	0.01	0.03	0.02
ω_11_(*a*_2_)	asym. CH wag	995.11	–0.01	–0.51	–0.04	–0.01	–0.11	–0.01	–0.50
ω_12_(*a*_2_)	asym. CH wag	890.79	0.00	0.56	0.02	0.01	0.12	0.00	0.56
ω_13_(*a*_2_)	ring twist	378.44	0.01	0.01	0.06	0.01	0.02	0.01	0.01
ω_14_(*b*_1_)	asym. CH wag	996.87	–0.69	–0.41	–0.11	–0.06	–0.06	–0.15	–0.16
ω_15_(*b*_1_)	sym. CH wag	953.64	0.02	0.12	–0.02	0.01	0.03	0.12	0.12
ω_16_(*b*_1_)	ring chair	753.17	–**1.58**	0.02	0.03	–0.02	–0.14	0.00	–0.30
ω_17_(*b*_1_)	sym. CH wag	711.89	**2.46**	0.36	0.12	0.08	0.19	0.03	0.36
ω_18_(*b*_1_)	ring boat	409.22	0.21	0.01	0.03	0.00	0.00	0.03	0.01
ω_19_(*b*_2_)	asym. CH str.	3204.49	–0.01	–0.03	–0.02	0.00	–0.01	0.00	–0.02
ω_20_(*b*_2_)	asym. CH str.	3168.42	0.00	0.03	–0.04	0.00	0.01	–0.01	0.00
ω_21_(*b*_2_)	ring str. def.	1618.43	–0.50	–0.46	–0.01	–0.04	–0.03	–0.25	–0.48
ω_22_(*b*_2_)	ring str. def.	1464.66	–1.02	–**6.46**	–0.10	–0.17	–0.18	0.17	0.06
ω_23_(*b*_2_)	sym. CH wag	1379.21	–1.34	–**22.61**	–0.12	–0.04	–0.06	0.04	0.29
ω_24_(*b*_2_)	ring str. def.	1266.95	0.38	**27.60**	–0.37	–**18.15**	–**1.56**	–0.01	–1.05
ω_25_(*b*_2_)	asym. CH wag	1158.82	**2.50**	**4.87**	0.75	**19.79**	**2.00**	0.02	1.30
ω_26_(*b*_2_)	ring str. def.	1071.35	0.65	0.07	0.07	0.05	0.02	0.09	0.07
ω_27_(*b*_2_)	ring shearing	656.98	0.11	0.01	0.02	0.01	0.00	0.03	0.01

a ξ cutoff = 0.015, η = 74%, and *n* = 20 off-diagonals included.

b ξ cutoff = 0.04, η = 22%, and *n* = 6 off-diagonals included.

The outlier normal modes of pyridine are quantitatively characterized by the total energy distributions (TEDs)^50−52^ provided in Table 6, where the classical vibrational energy of each mode *k* is decomposed into the predominant percentage contributions *n* attributable to the NICs *S*_*i*_. When B = CCSD(T)/cc-pVDZ, all of the outliers with |ϵ| > 1.5 cm^–1^ are removed by merely adding three off-diagonal force constants that couple the (ω_8_, ω_9_), (ω_16_, ω_17_), and (ω_24_, ω_25_) pairs, in accord with our earlier CMA-1A analysis of pyridine.^1^Table 6 shows that these three pairs mostly involve strong remixing of [*S*_1_(*a*_1_), *S*_12_(*a*_1_)], [*S*_20_(*b*_1_), *S*_25_(*b*_1_)], and [*S*_2_(*b*_2_), *S*_18_(*b*_2_), *S*_19_(*b*_2_)], respectively, which Table 4 in turn identifies as the vibrations (ring breathing, ring stellation), [chair ring pucker, out-of-plane (C_2_–H_8_) + (C_3_–H_9_) wag], and [ring stretch deformation, (C_2_–H_8_) – (C_3_–H_9_) in-plane rock, (C_1_–H_7_) in-plane rock]. The cluster of 4 outliers for B = MP2/cc-pVTZ does not exhibit simple remixing vis-à-vis the CCSD(T)/cc-pVTZ target modes. Once again *S*_2_(*b*_2_), *S*_18_(*b*_2_), and *S*_19_(*b*_2_) are culprits, but now the fray is joined by an alternative ring stretching deformation *S*_4_(*b*_2_) and the (C_4_–H_10_) – (C_5_–H_11_) in-plane rock *S*_16_(*b*_2_). The two large outliers for B = CCSD/cc-pVTZ have similar origins as in the MP2 case, but the vibrational interactions are less intricate and mostly restricted to the [*S*_2_(*b*_2_), *S*_18_(*b*_2_), *S*_19_(*b*_2_)] set; accordingly, a single (ω_24_, ω_25_) coupling within CMA-1A(1) eliminates these outliers. Collectively, the TED analyses in Table 6 suggest that CMA-0A might generally encounter difficulties with aromatic ring vibrations that couple antisymmetric ring stretching deformations with CH in-plane rocks of the same symmetry. Indeed, our work has also found some moderate residuals up to 5 cm^–1^ in size for these types of vibrations in benzene, pyrrole, and furan.

**Table 6:** Total Energy Distributions (TEDs) for the Outlier Modes of Pyridine at Various Levels of Theory

Mode	Frequency (cm^–1^)	TED^a^
CCSD(T)/cc-pVTZ		
ω_8_(*a*_1_)	1043.14	*S*_12_(51) + *S*_1_(33) + *S*_5_(13)
ω_9_(*a*_1_)	1001.24	*S*_1_(65) – *S*_12_(32)
ω_16_(*b*_1_)	753.17	*S*_20_(68) – *S*_25_(15) – *S*_27_(13)
ω_17_(*b*_1_)	711.89	*S*_20_(53) + *S*_25_(27) + *S*_27_(10) + *S*_23_(9)
ω_22_(*b*_2_)	1464.66	*S*_4_(32) – *S*_18_(25) + *S*_19_(20) + *S*_16_(15)
ω_23_(*b*_2_)	1379.21	*S*_16_(67) + *S*_18_(18) – *S*_19_(13)
ω_24_(*b*_2_)	1266.95	*S*_2_(78) + *S*_6_(8) – *S*_19_(8) – *S*_18_(7)
ω_25_(*b*_2_)	1158.82	*S*_18_(36) + *S*_19_(35) + *S*_2_(20) – *S*_6_(8)
CMA-0A[CCSD(T)/cc-pVTZ, CCSD(T)/cc-pVDZ]		
ω_8_(*a*_1_)	1039.90	*S*_1_(64) + *S*_12_(30)
ω_9_(*a*_1_)	1005.85	*S*_12_(61) – *S*_1_(34)
ω_16_(*b*_1_)	751.59	*S*_20_(46) – *S*_25_(24) – *S*_27_(20) – *S*_23_(11)
ω_17_(*b*_1_)	714.35	*S*_20_(75) + *S*_25_(20)
ω_25_(*b*_2_)	1161.32	*S*_18_(41) + *S*_19_(40) + *S*_2_(9) – *S*_6_(8)
CMA-0A[CCSD(T)/cc-pVTZ, MP2/cc-pVTZ]		
ω_22_(*b*_2_)	1458.20	*S*_4_(29) – *S*_18_(23) + *S*_19_(17) + *S*_16_(17)
ω_23_(*b*_2_)	1356.60	*S*_16_(56) + *S*_18_(17) – *S*_2_(15) – *S*_6_(6)
ω_24_(*b*_2_)	1294.55	*S*_2_(70) – *S*_19_(13) + *S*_16_(9)
ω_25_(*b*_2_)	1163.69	*S*_18_(42) + *S*_19_(41) – *S*_6_(10) + *S*_2_(6)
CMA-0A[CCSD(T)/cc-pVTZ, CCSD/cc-pVTZ]		
ω_24_(*b*_2_)	1248.80	*S*_2_(38) – *S*_18_(26) – *S*_19_(25) + *S*_6_(12)
ω_25_(*b*_2_)	1178.61	*S*_2_(61) + *S*_19_(18) + *S*_18_(18)

a For each normal mode *k*, the percentage *n* of the vibrational energy attributable to internal coordinate *i* is listed as S_*i*_(*n*), as calculated from eq 32 of ref (50). The signs preceding these entries denote the relative phases in the normal-mode eigenvectors.

While pyridine is an abnormal case for which CMA-0A is not fully adequate for some isolated modes, this challenging test molecule is totally vanquished by CMA-2A employing either B = CCSD(T)/cc-pVDZ or B = MP2/cc-pVTZ, as shown in Table 5. With our recommended B = MP2/cc-pVTZ and a ξ cutoff of 0.04, only 6 off-diagonals are chosen, and the cost increase is merely 22%. Nevertheless, the maximum absolute residual is reduced to 1.30 cm^–1^, while the MAE and standard deviation are 0.22 and 0.39 cm^–1^, respectively. For B = CCSD(T)/cc-pVDZ, a ξ cutoff of 0.015 is necessary to eliminate all outliers, resulting in 20 chosen off-diagonals and a 74% increase in cost; however, in the end the statistics achieved for the residuals are even better than in the MP2/cc-pVTZ case.

## 7 Application to 1-(1*H*-Pyrrol-3-yl)ethanol

**Figure 4:**
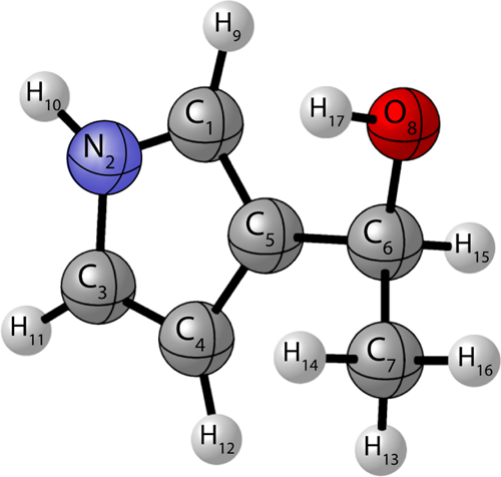
Enumerated atomic structure of 1-(1*H*-pyrrol-3-yl)ethanol, corresponding to selected internal coordinates utilized for all CMA computations.

The methodological advances achieved in this study were put to the test for the vibrations of a large molecule containing several challenging motifs, including a heterocyclic aromatic ring, aromatic N–H and C–H bonds, aliphatic O–H and C–H bonds, methyl and hydroxyl internal rotations, and torsions of entire monomer groups about a central C–C bond mediated by noncovalent interactions. In particular, the CMA-0A, CMA-1A(1), and CMA-2A methods with higher level A = CCSD(T)/cc-pVTZ and lower-level B = MP2/cc-pVTZ were applied to the 1-(1*H*-pyrrol-3-yl)ethanol conformer shown in Figure 4. This molecule lies outside the G2 test set, and very little is known about its vibrational spectrum. Benchmark CCSD(T)/cc-pVTZ harmonic frequencies and corresponding CMA residuals are reported in Table 7. The NICs were constructed by starting with the full set of customary coordinates for the individual P = pyrrole and E = ethanol monomers. Conceptually, the hydrogens on C_5_ and C_6_ were then removed while keeping the bond vectors fixed, whence the entire molecule was assembled by adding the C_5_–C_6_ distance and the intermonomer torsion angles about this linkage. The resulting 45 NICs for 1-(1*H*-pyrrol-3-yl)ethanol are provided in Table 8, and the Cartesian coordinates of all atoms are given in the Supporting Information.

**Table 7:** CMA-(0-2)A Residuals (ϵ, cm^–1^) for CCSD(T)/cc-pVTZ Harmonic Frequencies (ω_ref_, cm^–1^) of 1-(1*H*-Pyrrol-3-yl)ethanol Obtained with B = MP2/cc-pVTZ

			ω_ref_	ϵ[CMA-0A]	ϵ[CMA-1A(1)]	ϵ[CMA-2A]	ϵ[CMA-2A]
		Monomer^a^	CCSD(T)/cc-pVTZ	B = MP2/cc-pVTZ	B = MP2/cc-pVTZ	B = MP2/cc-pVTZ^b^	B = MP2/cc-pVTZ^c^
ω_1_	O–H stretch	E	3808.93	0.00	0.00	0.00	0.00
ω_2_	N–H stretch	P	3696.76	0.00	0.00	0.00	0.00
ω_3_	C_1_–H_9_ stretch	P	3276.98	0.03	0.03	0.03	0.03
ω_4_	C–H sym. stretch	P	3271.79	–0.01	–0.01	–0.01	–0.01
ω_5_	C–H antisym. stretch	P	3248.80	0.04	0.04	0.04	0.04
ω_6_	CH_3_ antisym. stretch	E	3124.60	–0.04	–0.04	–0.04	–0.04
ω_7_	CH_3_ sym. stretch	E	3108.32	–0.07	–0.07	–0.07	–0.07
ω_8_	C_6_–H_15_ stretch	E	3047.93	–0.00	0.00	0.00	0.00
ω_9_	CH_3_ sym. stretch	E	3031.56	0.05	0.05	0.05	0.05
ω_10_	ring stretching def.	P	1604.19	–0.70	–0.70	–0.25	–0.15
ω_11_	ring stretching def.	P	1521.94	–**1.61**	–**1.61**	–**1.56**	–0.11
ω_12_	CH_3_ def.	E	1498.72	–0.10	–0.10	–0.10	–0.10
ω_13_	CH_3_ scissor	E	1495.56	–0.09	–0.09	–0.09	–0.09
ω_14_	ring stretching def.	P	1475.70	0.61	0.61	0.76	–0.09
ω_15_	H_15_–C_6_–C_5_ wag	E	1429.80	–0.23	–0.23	–0.00	–0.02
ω_16_	ring stretching def.	P	1408.44	0.04	0.04	0.22	0.33
ω_17_	CH_3_ umbrella	E	1401.83	0.01	0.01	0.01	0.01
ω_18_	H_15_–C_6_–C_5_ rock	E	1365.39	0.33	0.33	0.33	0.34
ω_19_	C–O–H bend	E	1306.54	–0.29	–0.29	–0.29	–0.27
ω_20_	C_1_–H_9_ rock	P	1276.10	0.55	0.55	0.32	0.40
ω_21_	sym. C–H rock	P	1251.64	0.17	0.17	0.17	0.09
ω_22_	N–H rock	P	1156.61	0.72	0.72	0.15	0.02
ω_23_	C–O stretch	E	1125.73	–0.01	–0.01	–0.30	–0.36
ω_24_	ring breathing	P	1108.99	–0.10	–0.10	–0.10	–0.10
ω_25_	antisym. C–H rock	P	1088.87	0.67	0.67	0.37	0.54
ω_26_	CH_3_ rock	E	1067.33	0.48	0.47	0.48	0.23
ω_27_	CH_3_ wag	E	1034.92	0.14	0.14	0.14	0.13
ω_28_	ring shearing	P	958.88	0.06	0.06	0.06	0.06
ω_29_	ring bending	P	898.10	–0.01	–0.01	–0.01	–0.01
ω_30_	C_6_–C_7_ stretch	E	892.03	0.11	0.11	0.11	0.10
ω_31_	antisym. C–H wag	P	830.69	–0.47	–0.47	–0.21	–0.12
ω_32_	sym. C–H wag	P	789.78	–0.07	–0.07	–0.07	0.10
ω_33_	C_5_–C_6_ E wag	P ∩ E	701.34	–0.42	–0.42	–0.42	–0.03
ω_34_	antisym. C–H wag	P	696.28	0.52	0.52	0.29	–0.08
ω_35_	ring puckering	P	635.44	0.20	0.20	0.12	0.06
ω_36_	ring twisting	P	614.14	0.10	0.10	0.10	0.07
ω_37_	C_7_–C_6_–O_8_ bend	E	480.63	–**10.90**	0.04	0.04	–0.02
ω_38_	C_5_–C_6_ E rock	P ∩ E	457.57	–1.05	–1.05	–1.05	–1.05
ω_39_	N–H wag	P	442.97	**13.10**	**1.56**	**1.56**	1.20
ω_40_	C_5_–C_6_ stretch	P ∩ E	357.34	0.06	0.06	0.06	0.05
ω_41_	O–H torsion	E	320.88	0.04	0.04	0.04	0.04
ω_42_	CH_3_ torsion	E	261.50	0.02	0.02	0.02	0.02
ω_43_	C_6_–C_5_ P rock	P ∩ E	196.99	0.02	0.02	0.02	0.01
ω_44_	C_6_–C_5_ P wag	P ∩ E	172.27	0.04	0.04	0.04	0.02
ω_45_	P–E torsion	P ∩ E	47.91	0.03	0.03	0.03	0.02

a (P, E) = (pyrrole, ethanol).

b ξ cutoff = 0.04, η = 26.7%, and *n* = 12 off-diagonals included.

c ξ cutoff = 0.02, η = 140.0%, and *n* = 63 off-diagonals included.

**Table 8:** 1-(1*H*-Pyrrol-3-yl)ethanol Natural Internal Coordinates

Description	Monomer^a^	Unnormalized natural internal coordinate^b^
ring breathing	P	S_1_ = *r*(5,4) + *r*(1,5) + *r*(4,3) + *r*(2,1) + *r*(2,3)
ring stretching def.	P	S_2_ = *r*(5,4) + B *r*(1,5) + B *r*(4,3) + A *r*(2,1) + A *r*(2,3)
ring stretching def.	P	S_3_ = D *r*(1,5) – D *r*(4,3) + C *r*(2,1) – C *r*(2,3)
ring stretching def.	P	S_4_ = *r*(5,4) + A *r*(1,5) + A *r*(4,3) + B *r*(2,1) + B *r*(2,3)
ring stretching def.	P	S_5_ = C *r*(1,5) – C *r*(4,3) – D *r*(2,1) + D *r*(2,3)
C–C stretch	P ∩ E	S_6_ = *r*(5,6)
C–C stretch	E	S_7_ = *r*(6,7)
C–O stretch	E	S_8_ = *r*(6,8)
C–H stretch	P	S_9_ = *r*(1,9)
N–H stretch	P	S_10_ = *r*(2,10)
C–H stretch	P	S_11_ = *r*(3,11)
C–H stretch	P	S_12_ = *r*(4,12)
C–H stretch	E	S_13_ = *r*(6,15)
O–H stretch	E	S_14_ = *r*(8,17)
CH_3_ sym. stretch	E	S_15_ = *r*(7,13) + *r*(7,14) + *r*(7,16)
CH_3_ sym. stretch	E	S_16_ = 2*r*(7,13) – *r*(7,14) – *r*(7,16)
CH_3_ antisym. stretch	E	S_17_ = *r*(7,14) – *r*(7,16)
ring bending	P	S_18_ = θ(1,2,3) + A θ(2,1,5) + A θ(2,3,4) + B θ(1,5,4) + B θ(3,4,5)
ring shearing	P	S_19_ = (A – B) θ(2,1,5) – (A – B) θ(2,3,4) + (1 – A) θ(1,5,4) – (1 – A) θ(3,4,5)
C_6_–C_5_ P rock	P ∩ E	S_20_ = θ(6,5,4) – θ(6,5,1)
C_7_–C_6_–O bend	E	S_21_ = θ(8,6,7)
C_5_–C_6_ E wag	P ∩ E	S_22_ = θ(5,6,7) + θ(5,6,8)
C_5_–C_6_ E rock	P ∩ E	S_23_ = θ(5,6,7) – θ(5,6,8)
C–H rock	P	S_24_ = θ(9,1,2) – θ(9,1,5)
N–H rock	P	S_25_ = θ(10,2,3) – θ(10,2,1)
C–H rock	P	S_26_ = θ(11,3,4) – θ(11,3,2)
C–H rock	P	S_27_ = θ(12,4,5) – θ(12,4,3)
C–O–H bend	E	S_28_ = θ(17,8,6)
H–C–C rock	E	S_29_ = θ(15,6,7) + θ(15,6,8)
H–C–C wag	E	S_30_ = θ(15,6,7) – θ(15,6,8)
CH_3_ umbrella	E	S_31_ = θ(13,7,6) + θ(14,7,6) + θ(16,7,6) – θ(14,7,16) – θ(13,7,14) – θ(13,7,16)
CH_3_ rock	E	S_32_ = 2θ(13,7,6) – θ(14,7,6) – θ(16,7,6)
CH_3_ wag	E	S_33_ = θ(14,7,6) – θ(16,7,6)
CH_3_ scissor	E	S_34_ = 2θ(14,7,16) – θ(13,7,14) – θ(13,7,16)
CH_3_ def.	E	S_35_ = θ(13,7,14) – θ(13,7,16)
ring twisting	P	S_36_ = τ(1,5,4,3) + B τ(4,3,2,1) + B τ(3,2,1,5) + A τ(5,4,3,2) + A τ(2,1,5,4)
ring puckering	P	S_37_ = (1–A) τ(4,3,2,1) – (1–A) τ(3,2,1,5) – (B–A) τ(5,4,3,2) + (B–A) τ(2,1,5,4)
CH_3_ torsion	E	S_38_ = τ(13,7,6,8) + τ(14,7,6,8) + τ(16,7,6,8)
O–H torsion	E	S_39_ = τ(17,8,6,7)
P–E torsion	P ∩ E	S_40_ = τ(1,5,6,7) + τ(1,5,6,8) + τ(4,5,6,7) + τ(4,5,6,8)
C_6_–*C*_5_ P wag	P ∩ E	S_41_ = γ(6,5,1,4)
C–H wag	P	S_42_ = γ(9,1,2,5)
N–H wag	P	S_43_ = γ(10,2,3,1)
C–H wag	P	S_44_ = γ(11,3,4,2)
C–H wag	P	S_45_ = γ(12,4,5,3)

a (P, E) = (pyrrole, ethanol).

b (A, B, C, D) = .

For all but two frequencies, the performance of CMA-0A is excellent, achieving a MAE and maximum absolute residual (ϵ_max_) of only 0.24 and 1.61 cm^–1^, respectively, for 43 of the 45 normal modes. The two prominent outliers (ϵ_37_, ϵ_39_) = (10.9, –13.1) cm^–1^ arise from (ω_37_, ω_39_) = (480.6, 443.0) cm^–1^, whose largest TED components occur for the (C_7_–C_6_–O_8_ bend, N–H out-of-plane wag) coordinates. If CMA-1A(1) is applied with a single (ω_37_, ω_39_) coupling, then the (ϵ_37_, ϵ_39_) residuals are dramatically reduced to (0.04, 1.56) cm^–1^. What is remarkable about this solution is that the largest contributors for the two modes occur on different monomers, so that the coupling occurs at long-range across the C_5_–C_6_ linkage. A likely explanation is that the coupling involves noncovalent interactions of the aromatic π cloud with the polar O–H group. In any event, the system clearly provides a rigorous test for CMA methods. When CMA-2A is invoked with ξ = 0.04 (η = 27%, *n* = 12 couplings), the automatic selection process indeed picks up the (ω_37_, ω_39_) coupling, so that the MAE becomes a mere 0.22 cm^–1^ for the entire system of 45 vibrations. The residuals of largest size for CMA-2A(ξ = 0.04) are (ϵ_11_, ϵ_39_) = (−1.56, 1.56) cm^–1^, which barely qualify as outliers by our adopted CMA-2A threshold of 1.5 cm^–1^. While this level of performance is impressive, the application of CMA-2A with ξ = 0.02 (η = 140%, *n* = 63 couplings) removes any hint of an outlier. Specifically, the (MAE, ϵ_max_) metrics for this final run are (0.15, 1.2) cm^–1^.

All computations for 1-(1*H*-pyrrol-3-yl)ethanol were performed using 6 cores with 60 GB of memory on an Intel Xeon Gold 6130 processor in conjunction with a Luster file system. The CMA-0A, CMA-2A(ξ = 0.04), and CMA-2A(ξ = 0.02) results required a mere 4.3%, 5.6%, and 10.5%, respectively, of the CPU time for the complete CCSD(T)/cc-pVTZ harmonic frequency computation. In summary, CMA-2A gives a commanding performance for this test molecule. CMA-2A(ξ = 0.04) cuts the CPU time by a factor of 18 while evaluating all frequencies to better than 1.6 cm^–1^, whereas CMA-2A(ξ = 0.02) provides additional assurance against outliers yet still cuts the original cost by almost a factor of 10.

## 8 Summary

The Concordant Mode Approach (CMA) is a general protocol by which harmonic vibrational frequencies at a higher level theory A can be computed using a normal mode basis generated by a lower-level theory B, with cost reductions approaching an order of magnitude for larger molecules. Our introductory study^1^ on the G2 test set showed that CMA-0A can produce vibrational frequencies with at least a 99% probability of being accurate to within 1.5 cm^–1^ but with a 0.2–0.4% chance of outliers greater than 2.5 cm^–1^. The current research has focused on creating new CMA methods that rapidly and systematically converge to the exact Level A frequencies while also eliminating outliers. Overall, MP2/cc-pVTZ is found to be the best Level B when targeting CCSD(T)/cc-pVTZ frequencies. Our results show the importance of going beyond HF to incorporate electron correlation at Level B, but matching the quality of the Level A orbital basis set becomes paramount thereafter. Another key conclusion for Level B is that density-fitted (df) MP2 performs just as well as conventional MP2; hence, the reduced scaling of df-MP2 provides a wealth of opportunities for CMA applications on large molecules. The CMA-2A procedure developed here is demonstrated to be a robust, convergent method (Figure 2) that selects which off-diagonal force field elements to explicitly evaluate at Level A based on dimensionless ξ parameters that can be evaluated at a Level C with essentially no additional computational cost. Our current recommendation for CMA-2A applied to CCSD(T)/cc-pV*X*Z frequencies is to choose B = MP2/cc-pV*X*Z (or its df-MP2 variant) and C = HF/cc-pV*X*Z with ξ in the 0.02–0.04 range depending on the required certainty of the results. With ξ = 0.02 the CMA-2A residuals exhibit extremely small (MAE, σ_ϵ_, ϵ_max_) statistics of (0.05, 0.15, 0.17) cm^–1^, and the maximum absolute discrepancy (ϵ_MAX_) over the entire database is only 1.8 cm^–1^, all achieved with only a 33% increase (η) in average cost over CMA-0A. Moreover, the troublesome pyridine and challenging 1-(1*H*-pyrrol-3-yl)ethanol vibrational problems can be fully solved by CMA-2A with the less stringent threshold ξ = 0.04, resulting in (ϵ_MAX_, η) = (1.3 cm^–1^, 22%) and (1.6 cm^–1^, 27%) for these two molecules, respectively. The hierarchy CMA-*N* is now in place for *N* = 0, 1, 2, and the thoroughgoing success of these methods promises continuing improvements in cost and accuracy, applications to ever-larger molecules, and diversification into intermolecular vibrations. Other advances are also readily envisioned for which CMA-*N* can provide an ideal solution, such as the inclusion of core electron correlation and complete basis set (CBS) extrapolation in the determination of vibrational frequencies for larger molecules.

## Supporting Information Available

CMA-2A (RMSD, ϵ_max_) versus (η, percent off-diagonals in **F**_CMA_) plots. Cartesian coordinates for the CCSD(T)/cc-pVTZ structure of 1-(1*H*-pyrrol-3-yl)ethanol and all molecules of the G2 test set. 1501 pure, CMA-0A, and CMA-2A frequencies at ξ = 0.02 with A = CCSD(T)/cc-pVTZ, B = [MP2/cc-pVTZ, CCSD(T)/cc-pVDZ, B3LYP/6-31G(2*df*,*p*)], and C = [HF/cc-pVTZ, HF/cc-pVDZ, HF/6-31G(2*df*,*p*)] (PDF)
