# Supplementary material for: Convergent Concordant Mode Approach for Molecular Vibrations: CMA-2
Source: J Chem Theory Comput. 2024 Dec 13;20(24):10886–98. doi: 10.1021/acs.jctc.4c01240 (PMC11673116; doi:10.1021/acs.jctc.4c01240)
Supplement: Supplementary file 1 — ct4c01240_si_001.pdf [file ct4c01240_si_001.pdf]

Supporting Information: A Convergent Concordant Mode Approach for Molecular Vibrations: CMA-2

Nathaniel L. Kitzmiller,[†¶] Mitchell E. Lahm,[†] Laura N. Olive Dornshuld,[†] Jincan Jin, Wesley D. Allen,[‡†] and Henry F. Schaefer, III[†]

[†] *Center for Computational Quantum Chemistry, University of Georgia, Athens, GA 30602 United States*
[‡] *Allen Heritage Foundation, Dickson, TN 37055, United States*
[¶] *Indiana Wesleyan University, Marion, Indiana 46953, USA*

Contents

S1 Internal Coordinate Definitions **S5**

S2 CMA-2A Summary Statistics Plots **S6**

S3 1-(1H-pyrrol-3-yl)ethanol Cartesian coordinates **S8**

S4 CMA-2A Frequencies **S9**

S4.1 cyclopropane . . . S9
S4.2 methane . . . S13
S4.3 ammonia . . . S16
S4.4 silane . . . S19
S4.5 benzene . . . S22
S4.6 cyclopropene . . . S26
S4.7 allene . . . S29
S4.8 spiropentane . . . S32
S4.9 aluminum trichloride . . . S36
S4.10 aluminum trifluoride . . . S39
S4.11 boron trichloride . . . S42
S4.12 boron trifluoride . . . S45
S4.13 tetrachloromethane . . . S48
S4.14 tetrafluoromethane . . . S51
S4.15 dichloromethane . . . S54
S4.16 difluoromethane . . . S57
S4.17 formic acid . . . S60
S4.18 formaldehyde . . . S63
S4.19 singlet methylene . . . S66
S4.20 chloromethane . . . S69
S4.21 thiomethanol . . . S72
S4.22 trichloromethane . . . S75
S4.23 trifluoromethane . . . S78
S4.24 methyl nitrite . . . S81
S4.25 nitromethane . . . S84
S4.26 methylamine . . . S87
S4.27 ethylene . . . S90
S4.28 tetrafluoroethylene . . . S93
S4.29 tetrachloroethylene . . . S96
S4.30 acetylene . . . S99
S4.31 glyoxal . . . S102
S4.32 ketene . . . S105
S4.33 vinyl fluoride . . . S108
S4.34 vinyl chloride . . . S111
S4.35 acetyl chloride . . . S114
S4.36 acetyl fluoride . . . S117
S4.37 acetic acid . . . S120
S4.38 methyl formate . . . S124
S4.39 acetaldehyde . . . S128
S4.40 ethyl chloride . . . S131
S4.41 ethane . . . S135
S4.42 dimethyl ether . . . S139
S4.43 ethanol . . . S143
S4.44 acetonitrile . . . S147
S4.45 propyne . . . S150
S4.46 trifluoroacetonitrile . . . S153
S4.47 silicon tetrachloride . . . S156
S4.48 silicon tetrafluoride . . . S159

S4.49 disilane . . . S162
S4.50 methyl silane . . . S166
S4.51 phosphane . . . S170
S4.52 phosphorus trifluoride . . . S173
S4.53 hypochlorous acid . . . S176
S4.54 nitrosyl chloride . . . S179
S4.55 ozone . . . S182
S4.56 oxygen difluoride . . . S185
S4.57 water . . . S188
S4.58 trifluoroamine . . . S191
S4.59 chlorine trifluoride . . . S194
S4.60 hydrogen peroxide . . . S197
S4.61 carbonyl fluoride . . . S200
S4.62 singlet silylene . . . S203
S4.63 nitrous oxide . . . S206
S4.64 hydrazine . . . S209
S4.65 cyanogen . . . S212
S4.66 aziridine . . . S215
S4.67 acetamide . . . S219
S4.68 dimethylamine . . . S223
S4.69 ethylamine . . . S227
S4.70 acetone . . . S231
S4.71 1-chloropropane . . . S235
S4.72 methoxyethane . . . S239
S4.73 isopropyl alcohol . . . S243
S4.74 propane . . . S247
S4.75 acrylonitrile . . . S251
S4.76 trimethylamine . . . S254
S4.77 isobutane . . . S258
S4.78 *n*-butane . . . S262
S4.79 furan . . . S266
S4.80 1,3−butadiene . . . S270
S4.81 2-butyne . . . S274
S4.82 bicyclobutane . . . S278
S4.83 cyclobutene . . . S282
S4.84 methylenecyclopropane . . . S286
S4.85 cyclobutane . . . S290
S4.86 isobutene . . . S294
S4.87 pyrrole . . . S298
S4.88 sulfur dioxide . . . S302
S4.89 hydrogen sulfide . . . S305
S4.90 carbonyl sulfide . . . S308
S4.91 thiirane . . . S311
S4.92 dimethyl sulfide . . . S314
S4.93 thioethanol . . . S318
S4.94 dimethyl sulfoxide . . . S322
S4.95 thiophene . . . S326
S4.96 methanol . . . S330
S4.97 propene . . . S333
S4.98 oxirane . . . S337
S4.99 hydrogen cyanide . . . S340
S4.100 triplet methylene . . . S343
S4.101 formyl radical . . . S346
S4.102 vinyl radical . . . S349
S4.103 acetyl radical . . . S352
S4.104 hydroxymethyl radical . . . S355

S4.105 triplet silylene S358
S4.106 silyl radical S361
S4.107 phosphino radical S364
S4.108 nitrogen dioxide S367
S4.109 amino radical S370
S4.110 ethyl radical S373
S4.111 *tert*−butyl radical S376

S1 Internal Coordinate Definitions

The following are mathematical and qualitative definitions of the symbols utilized to represent different internal coordinate motions in the definition of the symmetrized, unnormalized natural internal coordinates that follow below.

Vector directed from atom i to atom j.

$$\mathbf{r}_{ij} = \mathbf{r}_j - \mathbf{r}_i$$

Unit vector directed from atom i to atom j.

$$\mathbf{e}_{ij} = \frac{\mathbf{r}_{ij}}{|\mathbf{r}_{ij}|}$$

Bond distance between atoms i and j.

$$r_{ij} = |\mathbf{r}_{ij}| \qquad 0 < r_{ij} < \infty$$

Bond angle defined by atoms i, j, and k, where j lies at the center of the bend.

$$\phi_{ijk} = \cos^{-1}(\mathbf{e}_{jk} \cdot \mathbf{e}_{ji}) \qquad 0 < \phi_{ijk} < \pi$$

Torsional angle between the planes defined by i, j, k and j, k, l.

$$\tau_{ijkl} = \sin^{-1}(\mathbf{e}_{ji} \cdot (\mathbf{e}_{kj} \times \mathbf{e}_{kl})/[\sin\phi_{ijk}\sin\phi_{jkl}]) \qquad -\pi/2 < \tau_{ijkl} < 3\pi/2$$
$$\tau_{ijkl} = \cos^{-1}((\mathbf{e}_{ji} \times \mathbf{e}_{jk}) \cdot (\mathbf{e}_{kj} \times \mathbf{e}_{kl})/[\sin\phi_{ijk}\sin\phi_{jkl}])$$

Out-of-plane bend of the i, j bond out of the plane defined by the l, j, k atoms.

$$\gamma_{ijkl} = \sin^{-1}(\mathbf{e}_{ji} \cdot (\mathbf{e}_{jk} \times \mathbf{e}_{jl})/[\sin\phi_{jkl}]) \qquad -\pi < \gamma_{ijkl} < \pi$$

Linear bend, where $\mathbf{e}_X$ is a fixed direction vector perpendicular to the bending plane defined by a dummy atom, X.

$$\theta_{ijkX} = \sin^{-1}[\mathbf{e}_X \cdot (\mathbf{e}_{jk} \times \mathbf{e}_{ji})]$$

Linear bend involving the x component of the $k \to l$ unit vector in the local coordinate system in which the $j \to k$ vector defines the $+z$ axis and the i atom lies in the xz plane in the $+x$ direction.

$$\alpha^x_{ijkl} = (\cos\tau_{ijkl})(\sin\phi_{jkl})$$

Linear bend involving the y component of the $k \to l$ unit vector in the local coordinate system in which the $j \to k$ vector defines the $+z$ axis and the i atom lies in the xz plane in the $+x$ direction.

$$\alpha^y_{ijkl} = (\sin\tau_{ijkl})(\sin\phi_{jkl})$$

S2 CMA-2A Summary Statistics Plots

Figure S1: The CMA-2A $\epsilon_{\max}$ of the 1501 CCSD(T)/cc-pVTZ benchmark frequencies plotted as a function of η, where Level C = HF and the basis set is the same as employed in Level B

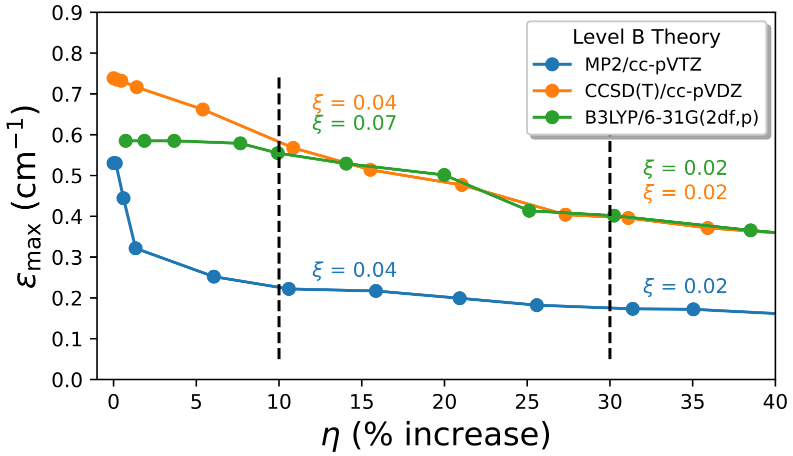

Figure S2: The CMA-2A RMSD of the 1501 CCSD(T)/cc-pVTZ benchmark frequencies plotted as a function of η, where Level C = HF and the basis set is the same as employed in Level B

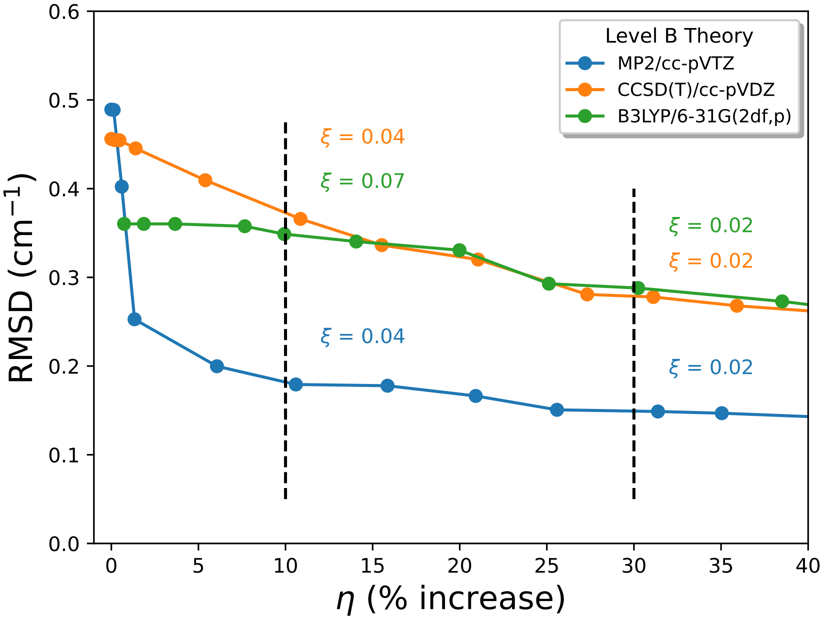

Figure S3: The CMA-2A $\epsilon_{\max}$ of the 1501 CCSD(T)/cc-pVTZ benchmark frequencies plotted as a function of % non-zero off-diagonal matrix elements included in $\mathbf{F}_{\mathrm{CMA}}(\mathrm{A})$

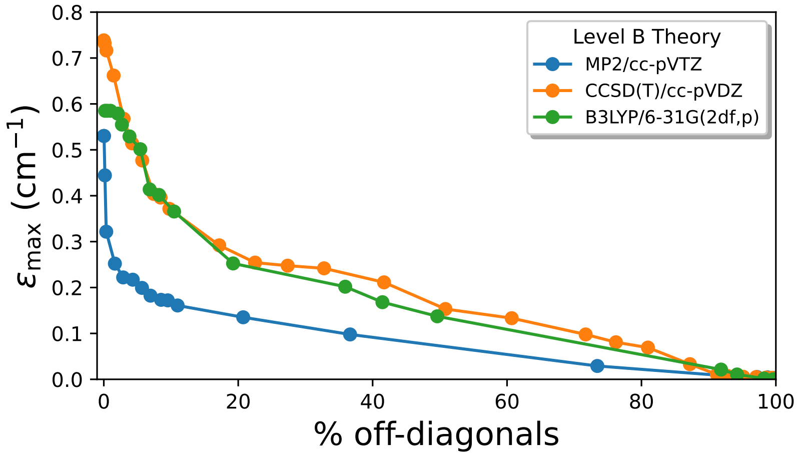

Figure S4: The CMA-2A RMSD of the 1501 CCSD(T)/cc-pVTZ benchmark frequencies plotted as a function of % non-zero off-diagonal matrix elements included in $\mathbf{F}_{\mathrm{CMA}}(\mathrm{A})$

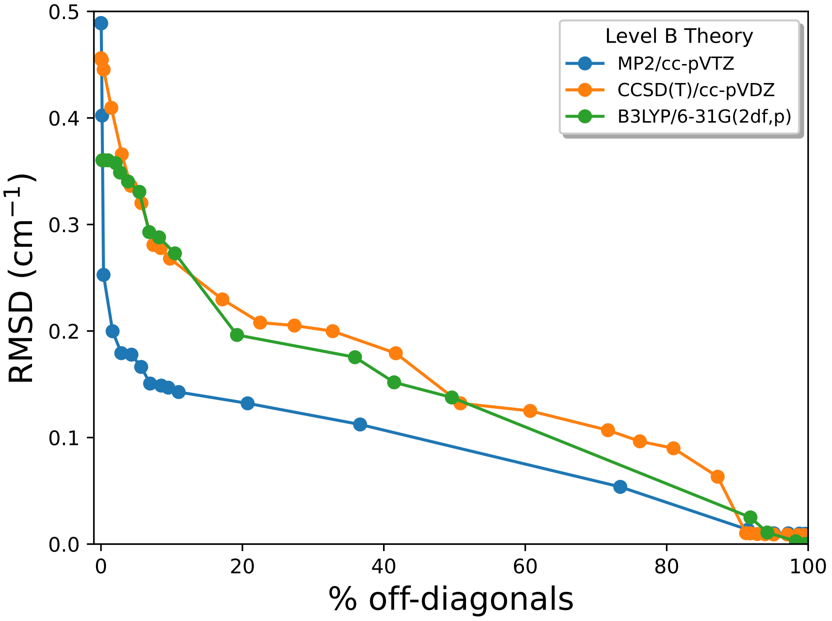

S3 1-(1H-pyrrol-3-yl)ethanol Cartesian coordinates

1-(1H-pyrrol-3-yl)ethanol Cartesian coordinates in bohr, optimized at CCSD(T)/cc-pVTZ

	X	Y	Z
C	0.8489210988	2.3962115053	0.4203945397
N	−1.1201865791	4.0834566335	0.2686904537
C	−3.2513769142	2.8724591283	−0.5805249925
C	−2.6398153189	0.3695666414	−0.9764274613
C	−0.0288888907	0.0573454839	−0.3406827522
C	1.5445264120	−2.3095417503	−0.4898725561
C	0.0435751715	−4.6686200430	0.2150310576
O	3.7681500684	−2.0852206753	1.0289210816
H	2.7056367112	2.9688534454	1.0228631156
H	−1.0206755773	5.9182273921	0.7349311276
H	−5.0097116242	3.8697874150	−0.8189836650
H	−3.9213447769	−1.0670336997	−1.6430077479
H	−1.5524659377	−4.9402871086	−1.0652587012
H	−0.6990000579	−4.4948396251	2.1360793372
H	2.2745647786	−2.5369365388	−2.4101383043
H	1.2622541295	−6.3283654748	0.1161828771
H	3.1939069316	−1.7609707648	2.7260744609

S4 CMA-2A Frequencies

S4.1 cyclopropane

Table S1: CCSD(T)/cc-pVTZ Optimum Cartesian coordinates (bohr)

1	C	−1.42667652	0.00000000	−0.82369207
2	C	−0.00000000	0.00000000	1.64738415
3	C	1.42667652	−0.00000000	−0.82369207
4	H	−2.37936667	1.72210724	−1.37372799
5	H	−2.37936667	−1.72210724	−1.37372799
6	H	−0.00000000	−1.72210724	2.74745597
7	H	−0.00000000	1.72210724	2.74745597
8	H	2.37936667	1.72210724	−1.37372799
9	H	2.37936667	−1.72210724	−1.37372799

Table S2: Reference [CCSD(T)/cc-pVTZ] CMA-0A and CMA-2A (ξ = 0.02) harmonic frequencies for Level B = MP2/cc-pVTZ and CCSD(T)/cc-pVDZ.

	Reference CCSD(T)/ cc-pVTZ	Pure MP2/ cc-pVTZ	CMA-0A MP2/ cc-pVTZ	CMA-2A MP2/ cc-pVTZ	Pure CCSD(T)/ cc-pVDZ	CMA-0A CCSD(T)/ cc-pVDZ	CMA-2A CCSD(T)/ cc-pVDZ
$\omega_1(a_1^{'})$	3156.95	3171.65	3156.94	3156.94	3297.40	3156.93	3156.93
$\omega_2(a_1^{'})$	1528.61	1524.50	1528.58	1528.58	1555.22	1527.57	1528.63
$\omega_3(a_1^{'})$	1214.39	1217.37	1214.43	1214.43	1250.91	1215.76	1214.42
$\omega_4(a_2^{'})$	1160.87	1163.77	1160.87	1160.87	1172.69	1160.87	1160.87
$\omega_{5a}(e^{'})$	3146.29	3163.15	3146.29	3146.29	3284.42	3146.28	3146.28
$\omega_{5b}(e^{'})$	3146.29	3163.10	3146.29	3146.29	3284.37	3146.28	3146.28
$\omega_{6a}(e^{'})$	1478.32	1478.46	1478.31	1478.31	1489.43	1478.26	1478.26
$\omega_{6b}(e^{'})$	1478.32	1478.46	1478.31	1478.31	1489.43	1478.26	1478.26
$\omega_{7a}(e^{'})$	1060.52	1047.53	1060.34	1060.34	1083.50	1059.32	1060.57
$\omega_{7b}(e^{'})$	1060.52	1047.48	1060.34	1060.34	1083.44	1059.32	1060.57
$\omega_{8a}(e^{'})$	891.96	894.37	892.19	892.19	923.73	893.53	892.03
$\omega_{8b}(e^{'})$	891.96	894.34	892.19	892.19	923.70	893.52	892.03
$\omega_9(a_1^{''})$	1088.57	1077.76	1088.57	1088.57	1100.41	1088.57	1088.57
$\omega_{10}(a_2^{''})$	3248.62	3271.17	3248.62	3248.62	3399.21	3248.61	3248.61
$\omega_{11}(a_2^{''})$	857.68	860.46	857.69	857.69	870.33	857.73	857.73
$\omega_{12a}(e^{''})$	3228.73	3252.66	3228.73	3228.73	3380.28	3228.72	3228.72
$\omega_{12b}(e^{''})$	3228.73	3252.60	3228.73	3228.73	3380.22	3228.72	3228.72
$\omega_{13a}(e^{''})$	1217.35	1213.99	1217.35	1217.35	1227.23	1217.34	1217.34
$\omega_{13b}(e^{''})$	1217.35	1213.98	1217.35	1217.35	1227.22	1217.34	1217.34
$\omega_{14a}(e^{''})$	740.85	740.69	740.86	740.86	749.03	740.89	740.89
$\omega_{14b}(e^{''})$	740.85	740.68	740.86	740.86	749.02	740.89	740.89

Table S3: CMA-0A and CMA-2A (ξ = 0.02) harmonic frequencies targeting CCSD(T)/cc-pVTZ for Level B = B3LYP/6-31G(2*df*,*p*).

	Pure B3LYP/ 6-31G(2*df*,*p*)	CMA-0A B3LYP/ 6-31G(2*df*,*p*)	CMA-2A B3LYP/ 6-31G(2*df*,*p*)
$\omega_1(a_1^{'})$	3167.76	3156.90	3156.90
$\omega_2(a_1^{'})$	1515.16	1528.61	1528.61
$\omega_3(a_1^{'})$	1214.85	1214.51	1214.51
$\omega_4(a_2^{'})$	1157.21	1160.87	1160.87
$\omega_{5\mathrm{a}}(e^{'})$	3159.72	3146.30	3146.30
$\omega_{5\mathrm{b}}(e^{'})$	3158.86	3146.27	3146.27
$\omega_{6\mathrm{a}}(e^{'})$	1475.90	1478.28	1478.28
$\omega_{6\mathrm{b}}(e^{'})$	1474.44	1478.28	1478.28
$\omega_{7\mathrm{a}}(e^{'})$	1065.26	1060.56	1060.56
$\omega_{7\mathrm{b}}(e^{'})$	1064.70	1060.49	1060.49
$\omega_{8\mathrm{a}}(e^{'})$	887.43	892.06	892.06
$\omega_{8\mathrm{b}}(e^{'})$	887.18	892.05	892.05
$\omega_9(a_1^{''})$	1093.92	1088.57	1088.57
$\omega_{10}(a_2^{''})$	3264.39	3248.62	3248.62
$\omega_{11}(a_2^{''})$	859.41	857.68	857.68
$\omega_{12\mathrm{a}}(e^{''})$	3241.72	3228.73	3228.73
$\omega_{12\mathrm{b}}(e^{''})$	3241.11	3228.73	3228.73
$\omega_{13\mathrm{a}}(e^{''})$	1215.33	1217.34	1217.34
$\omega_{13\mathrm{b}}(e^{''})$	1214.95	1217.34	1217.34
$\omega_{14\mathrm{a}}(e^{''})$	739.17	740.87	740.87
$\omega_{14\mathrm{b}}(e^{''})$	738.89	740.87	740.87

Table S4: Symmetrized, unnormalized natural internal coordinates for cyclopropane.

1	$r_{2,3} + r_{1,2} + r_{1,3}$
2	$2r_{2,3} - r_{1,2} - r_{1,3}$
3	$r_{1,2} - r_{1,3}$
4	$r_{1,4} + r_{1,5} + r_{2,7} + r_{2,6} + r_{3,8} + r_{3,9}$
5	$2r_{1,4} + 2r_{1,5} - r_{2,7} - r_{2,6} - r_{3,8} - r_{3,9}$
6	$r_{2,7} + r_{2,6} - r_{3,8} - r_{3,9}$
7	$r_{1,4} - r_{1,5} + r_{2,7} - r_{2,6} + r_{3,8} - r_{3,9}$
8	$2r_{1,4} - 2r_{1,5} - r_{2,7} + r_{2,6} - r_{3,8} + r_{3,9}$
9	$r_{2,7} - r_{2,6} - r_{3,8} + r_{3,9}$
10	$4\phi_{4,1,5} - \phi_{4,1,2} - \phi_{4,1,3} - \phi_{5,1,2} - \phi_{5,1,3} + 4\phi_{6,2,7} - \phi_{7,2,3} - \phi_{7,2,1} - \phi_{6,2,3} - \phi_{6,2,1}$ $+4\phi_{8,3,9} - \phi_{8,3,1} - \phi_{8,3,2} - \phi_{9,3,1} - \phi_{9,3,2}$
11	$8\phi_{4,1,5} - 2\phi_{4,1,2} - 2\phi_{4,1,3} - 2\phi_{5,1,2} - 2\phi_{5,1,3} - 4\phi_{6,2,7} + \phi_{7,2,3} + \phi_{7,2,1} + \phi_{6,2,3} + \phi_{6,2,1}$ $-4\phi_{8,3,9} + \phi_{8,3,1} + \phi_{8,3,2} + \phi_{9,3,1} + \phi_{9,3,2}$
12	$4\phi_{6,2,7} - \phi_{7,2,3} - \phi_{7,2,1} - \phi_{6,2,3} - \phi_{6,2,1} - 4\phi_{8,3,9} + \phi_{8,3,1} + \phi_{8,3,2} + \phi_{9,3,1} + \phi_{9,3,2}$
13	$\phi_{4,1,2} + \phi_{4,1,3} - \phi_{5,1,2} - \phi_{5,1,3} + \phi_{7,2,3} + \phi_{7,2,1} - \phi_{6,2,3} - \phi_{6,2,1} + \phi_{8,3,1} + \phi_{8,3,2}$ $-\phi_{9,3,1} - \phi_{9,3,2}$
14	$2\phi_{4,1,2} + 2\phi_{4,1,3} - 2\phi_{5,1,2} - 2\phi_{5,1,3} - \phi_{7,2,3} - \phi_{7,2,1} + \phi_{6,2,3} + \phi_{6,2,1} - \phi_{8,3,1} - \phi_{8,3,2}$ $+\phi_{9,3,1} + \phi_{9,3,2}$
15	$\phi_{7,2,3} + \phi_{7,2,1} - \phi_{6,2,3} - \phi_{6,2,1} - \phi_{8,3,1} - \phi_{8,3,2} + \phi_{9,3,1} + \phi_{9,3,2}$
16	$\phi_{4,1,2} - \phi_{4,1,3} + \phi_{5,1,2} - \phi_{5,1,3} + \phi_{7,2,3} - \phi_{7,2,1} + \phi_{6,2,3} - \phi_{6,2,1} + \phi_{8,3,1} - \phi_{8,3,2}$ $+\phi_{9,3,1} - \phi_{9,3,2}$
17	$2\phi_{4,1,2} - 2\phi_{4,1,3} + 2\phi_{5,1,2} - 2\phi_{5,1,3} - \phi_{7,2,3} + \phi_{7,2,1} - \phi_{6,2,3} + \phi_{6,2,1} - \phi_{8,3,1} + \phi_{8,3,2}$ $-\phi_{9,3,1} + \phi_{9,3,2}$
18	$\phi_{7,2,3} - \phi_{7,2,1} + \phi_{6,2,3} - \phi_{6,2,1} - \phi_{8,3,1} + \phi_{8,3,2} - \phi_{9,3,1} + \phi_{9,3,2}$
19	$\phi_{4,1,2} - \phi_{4,1,3} - \phi_{5,1,2} + \phi_{5,1,3} + \phi_{7,2,3} - \phi_{7,2,1} - \phi_{6,2,3} + \phi_{6,2,1} + \phi_{8,3,1} - \phi_{8,3,2}$ $-\phi_{9,3,1} + \phi_{9,3,2}$
20	$2\phi_{4,1,2} - 2\phi_{4,1,3} - 2\phi_{5,1,2} + 2\phi_{5,1,3} - \phi_{7,2,3} + \phi_{7,2,1} + \phi_{6,2,3} - \phi_{6,2,1} - \phi_{8,3,1} + \phi_{8,3,2}$ $+\phi_{9,3,1} - \phi_{9,3,2}$
21	$\phi_{7,2,3} - \phi_{7,2,1} - \phi_{6,2,3} + \phi_{6,2,1} - \phi_{8,3,1} + \phi_{8,3,2} + \phi_{9,3,1} - \phi_{9,3,2}$

S4.2 methane

Table S5: CCSD(T)/cc-pVTZ Optimum Cartesian coordinates (bohr)

1	C	0.00000000	0.00000000	0.00000000
2	H	0.00000000	−1.68028027	1.18813758
3	H	0.00000000	1.68028027	1.18813758
4	H	1.68028027	0.00000000	−1.18813758
5	H	−1.68028027	−0.00000000	−1.18813758

Table S6: Reference [CCSD(T)/cc-pVTZ] CMA-0A and CMA-2A (ξ = 0.02) harmonic frequencies for Level B = MP2/cc-pVTZ and CCSD(T)/cc-pVDZ.

	Reference CCSD(T)/ cc-pVTZ	Pure MP2/ cc-pVTZ	CMA-0A MP2/ cc-pVTZ	CMA-2A MP2/ cc-pVTZ	Pure CCSD(T)/ cc-pVDZ	CMA-0A CCSD(T)/ cc-pVDZ	CMA-2A CCSD(T)/ cc-pVDZ
$\omega_1(a_1)$	3034.66	3045.57	3034.66	3034.66	3167.65	3034.66	3034.66
$\omega_{2\text{a}}(e)$	1570.80	1578.61	1570.80	1570.80	1581.56	1570.80	1570.80
$\omega_{2\text{b}}(e)$	1570.80	1578.60	1570.80	1570.80	1581.55	1570.80	1570.80
$\omega_{3\text{a}}(t_2)$	3153.78	3179.02	3153.78	3153.78	3312.67	3153.74	3153.74
$\omega_{3\text{b}}(t_2)$	3153.78	3178.77	3153.78	3153.78	3312.41	3153.74	3153.74
$\omega_{3\text{c}}(t_2)$	3153.78	3178.77	3153.78	3153.78	3312.41	3153.74	3153.74
$\omega_{4\text{a}}(t_2)$	1343.99	1343.24	1344.00	1344.00	1359.68	1344.11	1344.11
$\omega_{4\text{b}}(t_2)$	1343.99	1343.17	1344.00	1344.00	1359.63	1344.11	1344.11
$\omega_{4\text{c}}(t_2)$	1343.99	1343.17	1344.00	1344.00	1359.63	1344.11	1344.11

Table S7: CMA-0A and CMA-2A (ξ = 0.02) harmonic frequencies targeting CCSD(T)/cc-pVTZ for Level B = B3LYP/6-31G(2*df*,*p*).

	Pure B3LYP/ 6-31G(2*df*,*p*)	CMA-0A B3LYP/ 6-31G(2*df*,*p*)	CMA-2A B3LYP/ 6-31G(2*df*,*p*)
$\omega_1(a_1)$	3063.69	3034.66	3034.66
$\omega_{2\text{a}}(e)$	1570.30	1570.80	1570.80
$\omega_{2\text{b}}(e)$	1570.25	1570.80	1570.80
$\omega_{3\text{a}}(t_2)$	3176.73	3153.78	3153.78
$\omega_{3\text{b}}(t_2)$	3176.53	3153.78	3153.78
$\omega_{3\text{c}}(t_2)$	3176.53	3153.78	3153.78
$\omega_{4\text{a}}(t_2)$	1351.95	1343.99	1343.99
$\omega_{4\text{b}}(t_2)$	1351.95	1343.99	1343.99
$\omega_{4\text{c}}(t_2)$	1351.94	1343.99	1343.99

Table S8: Symmetrized, unnormalized natural internal coordinates for methane.

1	$r_{1,2} + r_{1,3} + r_{1,4} + r_{1,5}$
2	$-r_{1,2} - r_{1,3} + r_{1,4} + r_{1,5}$
3	$-r_{1,2} + r_{1,3} - r_{1,4} + r_{1,5}$
4	$r_{1,2} - r_{1,3} - r_{1,4} + r_{1,5}$
5	$2\phi_{2,1,3} + 2\phi_{4,1,5} - \phi_{2,1,4} - \phi_{2,1,5} - \phi_{3,1,4} - \phi_{3,1,5}$
6	$\phi_{2,1,4} - \phi_{2,1,5} - \phi_{3,1,4} + \phi_{3,1,5}$
7	$-\phi_{2,1,3} + \phi_{4,1,5}$
8	$-\phi_{2,1,4} + \phi_{3,1,5}$
9	$\phi_{2,1,5} - \phi_{3,1,4}$

S4.3 ammonia

Table S9: CCSD(T)/cc-pVTZ Optimum Cartesian coordinates (bohr)

1	N	−0.13335323	−0.00000078	−0.00000000
2	H	0.61762190	−1.76309423	−0.00000000
3	H	0.61761729	0.88155253	1.52688219
4	H	0.61761729	0.88155253	−1.52688219

Table S10: Reference [CCSD(T)/cc-pVTZ] CMA-0A and CMA-2A (ξ = 0.02) harmonic frequencies for Level B = MP2/cc-pVTZ and CCSD(T)/cc-pVDZ.

	Reference CCSD(T)/ cc-pVTZ	Pure MP2/ cc-pVTZ	CMA-0A MP2/ cc-pVTZ	CMA-2A MP2/ cc-pVTZ	Pure CCSD(T)/ cc-pVDZ	CMA-0A CCSD(T)/ cc-pVDZ	CMA-2A CCSD(T)/ cc-pVDZ
$\omega_1(a_1)$	3471.91	3483.02	3471.91	3471.91	3575.04	3471.81	3471.91
$\omega_2(a_1)$	1109.21	1097.96	1109.22	1109.22	1097.45	1109.52	1109.21
$\omega_{3\mathrm{a}}(e)$	3597.54	3623.57	3597.54	3597.54	3720.21	3597.51	3597.51
$\omega_{3\mathrm{b}}(e)$	3597.54	3623.41	3597.54	3597.54	3720.03	3597.51	3597.51
$\omega_{4\mathrm{a}}(e)$	1687.93	1683.58	1687.93	1687.93	1688.35	1687.99	1687.99
$\omega_{4\mathrm{b}}(e)$	1687.93	1683.53	1687.93	1687.93	1688.30	1687.99	1687.99

Table S11: CMA-0A and CMA-2A (ξ = 0.02) harmonic frequencies targeting CCSD(T)/cc-pVTZ for Level B = B3LYP/6-31G(2*df*,*p*).

	Pure B3LYP/ 6-31G(2*df*,*p*)	CMA-0A B3LYP/ 6-31G(2*df*,*p*)	CMA-2A B3LYP/ 6-31G(2*df*,*p*)
$\omega_1(a_1)$	3494.96	3471.91	3471.91
$\omega_2(a_1)$	1093.50	1109.22	1109.22
$\omega_{3\mathrm{a}}(e)$	3614.73	3597.53	3597.53
$\omega_{3\mathrm{b}}(e)$	3614.55	3597.53	3597.53
$\omega_{4\mathrm{a}}(e)$	1683.34	1687.94	1687.94
$\omega_{4\mathrm{b}}(e)$	1683.25	1687.94	1687.94

Table S12: Symmetrized, unnormalized natural internal coordinates for ammonia.

1	$r_{1,2} + r_{1,3} + r_{1,4}$
2	$2r_{1,2} - r_{1,3} - r_{1,4}$
3	$r_{1,3} - r_{1,4}$
4	$2\phi_{2,1,3} - \phi_{2,1,4} - \phi_{3,1,4}$
5	$\phi_{2,1,4} - \phi_{3,1,4}$
6	$\gamma_{2,1,3,4} + \gamma_{3,1,4,2} + \gamma_{4,1,2,3}$

S4.4 silane

Table S13: CCSD(T)/cc-pVTZ Optimum Cartesian coordinates (bohr)

1	Si	0.00000000	0.00000000	0.00000000
2	H	0.00000000	−2.28308541	1.61437763
3	H	0.00000000	2.28308541	1.61437763
4	H	2.28308541	0.00000000	−1.61437763
5	H	−2.28308541	0.00000000	−1.61437763

Table S14: Reference [CCSD(T)/cc-pVTZ] CMA-0A and CMA-2A (ξ = 0.02) harmonic frequencies for Level B = MP2/cc-pVTZ and CCSD(T)/cc-pVDZ.

	Reference CCSD(T)/ cc-pVTZ	Pure MP2/ cc-pVTZ	CMA-0A MP2/ cc-pVTZ	CMA-2A MP2/ cc-pVTZ	Pure CCSD(T)/ cc-pVDZ	CMA-0A CCSD(T)/ cc-pVDZ	CMA-2A CCSD(T)/ cc-pVDZ
$\omega_1(a_1)$	2250.62	2284.43	2250.62	2250.62	2277.03	2250.62	2250.62
$\omega_{2\mathrm{a}}(e)$	985.49	1001.77	985.48	985.48	989.05	985.49	985.49
$\omega_{2\mathrm{b}}(e)$	985.48	1001.76	985.48	985.48	989.05	985.48	985.48
$\omega_{3\mathrm{a}}(t_2)$	2255.36	2291.66	2255.36	2255.36	2287.50	2255.36	2255.36
$\omega_{3\mathrm{b}}(t_2)$	2255.36	2291.56	2255.36	2255.36	2287.41	2255.36	2255.36
$\omega_{3\mathrm{c}}(t_2)$	2255.36	2291.56	2255.35	2255.35	2287.41	2255.36	2255.36
$\omega_{4\mathrm{a}}(t_2)$	933.80	948.87	933.80	933.80	940.48	933.80	933.80
$\omega_{4\mathrm{b}}(t_2)$	933.80	948.82	933.80	933.80	940.43	933.79	933.79
$\omega_{4\mathrm{c}}(t_2)$	933.79	948.81	933.79	933.79	940.43	933.79	933.79

Table S15: CMA-0A and CMA-2A (ξ = 0.02) harmonic frequencies targeting CCSD(T)/cc-pVTZ for Level B = B3LYP/6-31G(2*df*,*p*).

	Pure B3LYP/ 6-31G(2*df*,*p*)	CMA-0A B3LYP/ 6-31G(2*df*,*p*)	CMA-2A B3LYP/ 6-31G(2*df*,*p*)
$\omega_1(a_1)$	2261.16	2250.62	2250.62
$\omega_{2\mathrm{a}}(e)$	979.64	985.49	985.49
$\omega_{2\mathrm{b}}(e)$	979.50	985.48	985.48
$\omega_{3\mathrm{a}}(t_2)$	2271.95	2255.37	2255.37
$\omega_{3\mathrm{b}}(t_2)$	2271.15	2255.36	2255.36
$\omega_{3\mathrm{c}}(t_2)$	2271.15	2255.34	2255.34
$\omega_{4\mathrm{a}}(t_2)$	921.81	933.80	933.80
$\omega_{4\mathrm{b}}(t_2)$	921.35	933.79	933.79
$\omega_{4\mathrm{c}}(t_2)$	921.34	933.79	933.79

Table S16: Symmetrized, unnormalized natural internal coordinates for silane.

1	$r_{1,2} + r_{1,3} + r_{1,4} + r_{1,5}$
2	$-r_{1,2} - r_{1,3} + r_{1,4} + r_{1,5}$
3	$-r_{1,2} + r_{1,3} - r_{1,4} + r_{1,5}$
4	$r_{1,2} - r_{1,3} - r_{1,4} + r_{1,5}$
5	$2\phi_{2,1,3} + 2\phi_{4,1,5} - \phi_{2,1,4} - \phi_{2,1,5} - \phi_{3,1,4} - \phi_{3,1,5}$
6	$\phi_{2,1,4} - \phi_{2,1,5} - \phi_{3,1,4} + \phi_{3,1,5}$
7	$-\phi_{2,1,3} + \phi_{4,1,5}$
8	$-\phi_{2,1,4} + \phi_{3,1,5}$
9	$\phi_{2,1,5} - \phi_{3,1,4}$

S4.5 benzene

Table S17: CCSD(T)/cc-pVTZ Optimum Cartesian coordinates (bohr)

1	C	0.00000000	2.64096559	−0.00000000
2	C	−2.28714329	1.32048279	−0.00000000
3	C	−2.28714329	−1.32048279	0.00000000
4	C	−0.00000000	−2.64096559	0.00000000
5	C	2.28714329	−1.32048279	0.00000000
6	C	2.28714329	1.32048279	−0.00000000
7	H	0.00000000	4.68777554	−0.00000000
8	H	−4.05973271	2.34388777	−0.00000000
9	H	−4.05973271	−2.34388777	0.00000000
10	H	−0.00000000	−4.68777554	0.00000000
11	H	4.05973271	−2.34388777	0.00000000
12	H	4.05973271	2.34388777	−0.00000000

Table S18: Reference [CCSD(T)/cc-pVTZ] CMA-0A and CMA-2A (ξ = 0.02) harmonic frequencies for Level B = MP2/cc-pVTZ and CCSD(T)/cc-pVDZ.

	Reference CCSD(T)/ cc-pVTZ	Pure MP2/ cc-pVTZ	CMA-0A MP2/ cc-pVTZ	CMA-2A MP2/ cc-pVTZ	Pure CCSD(T)/ cc-pVDZ	CMA-0A CCSD(T)/ cc-pVDZ	CMA-2A CCSD(T)/ cc-pVDZ
$\omega_1(a_{1g})$	3209.15	3224.30	3209.15	3209.15	3348.27	3209.14	3209.14
$\omega_2(a_{1g})$	1004.54	1003.25	1004.54	1004.54	1041.17	1004.55	1004.55
$\omega_3(a_{2g})$	1370.18	1364.96	1370.18	1370.18	1386.82	1370.18	1370.18
$\omega_4(a_{2u})$	685.29	687.72	685.29	685.29	694.05	685.29	685.29
$\omega_5(b_{1u})$	3169.39	3186.60	3169.39	3169.39	3308.98	3169.39	3169.39
$\omega_6(b_{1u})$	1010.33	1012.70	1010.33	1010.33	1015.18	1010.34	1010.34
$\omega_7(b_{2g})$	966.66	971.83	966.57	966.66	978.12	966.05	966.66
$\omega_8(b_{2g})$	674.67	682.19	674.80	674.67	644.35	675.54	674.67
$\omega_9(b_{2u})$	1328.17	1439.45	1323.88	1328.17	1413.84	1326.49	1328.17
$\omega_{10}(b_{2u})$	1158.88	1164.22	1163.78	1158.88	1182.54	1160.80	1158.88
$\omega_{11a}(e_{1g})$	856.44	859.05	856.44	856.44	870.81	856.44	856.44
$\omega_{11b}(e_{1g})$	856.44	859.05	856.44	856.44	870.80	856.44	856.44
$\omega_{12a}(e_{1u})$	3198.33	3214.58	3198.33	3198.33	3337.35	3198.33	3198.33
$\omega_{12b}(e_{1u})$	3198.33	3214.52	3198.33	3198.33	3337.29	3198.33	3198.33
$\omega_{13a}(e_{1u})$	1506.94	1497.86	1506.94	1506.94	1542.40	1506.49	1506.94
$\omega_{13b}(e_{1u})$	1506.94	1497.84	1506.94	1506.94	1542.39	1506.48	1506.94
$\omega_{14a}(e_{1u})$	1054.36	1053.53	1054.37	1054.36	1084.14	1055.03	1054.38
$\omega_{14b}(e_{1u})$	1054.36	1053.52	1054.37	1054.36	1084.12	1055.03	1054.38
$\omega_{15a}(e_{2g})$	3180.74	3198.12	3180.74	3180.74	3319.85	3180.73	3180.73
$\omega_{15b}(e_{2g})$	3180.74	3198.06	3180.74	3180.74	3319.79	3180.73	3180.73
$\omega_{16a}(e_{2g})$	1637.23	1624.59	1637.21	1637.23	1699.02	1636.82	1637.20
$\omega_{16b}(e_{2g})$	1637.23	1624.57	1637.21	1637.23	1699.01	1636.82	1637.20
$\omega_{17a}(e_{2g})$	1190.57	1190.38	1190.61	1190.57	1211.98	1191.10	1190.58
$\omega_{17b}(e_{2g})$	1190.57	1190.38	1190.61	1190.57	1211.98	1191.10	1190.58
$\omega_{18a}(e_{2g})$	607.13	603.08	607.14	607.14	617.60	607.22	607.22
$\omega_{18b}(e_{2g})$	607.13	603.07	607.14	607.14	617.60	607.22	607.22
$\omega_{19a}(e_{2u})$	959.08	958.05	959.08	959.08	974.01	959.07	959.08
$\omega_{19b}(e_{2u})$	959.08	958.03	959.08	959.08	974.00	959.07	959.08
$\omega_{20a}(e_{2u})$	401.34	402.88	401.34	401.34	404.15	401.36	401.34
$\omega_{20b}(e_{2u})$	401.34	402.87	401.34	401.34	404.15	401.36	401.34

Table S19: CMA-0A and CMA-2A (ξ = 0.02) harmonic frequencies targeting CCSD(T)/cc-pVTZ for Level B = B3LYP/6-31G(2df,p).

	Pure B3LYP/ 6-31G(2df,p)	CMA-0A B3LYP/ 6-31G(2df,p)	CMA-2A B3LYP/ 6-31G(2df,p)
$\omega_1(a_{1g})$	3219.15	3209.14	3209.14
$\omega_2(a_{1g})$	1006.60	1004.55	1004.55
$\omega_3(a_{2g})$	1373.98	1370.18	1370.18
$\omega_4(a_{2u})$	696.71	685.29	685.29
$\omega_5(b_{1u})$	3180.31	3169.39	3169.39
$\omega_6(b_{1u})$	1013.66	1010.34	1010.34
$\omega_7(b_{2g})$	1008.62	964.26	966.66
$\omega_8(b_{2g})$	715.75	678.10	674.67
$\omega_9(b_{2u})$	1324.89	1327.54	1328.17
$\omega_{10}(b_{2u})$	1173.75	1159.61	1158.88
$\omega_{11a}(e_{1g})$	871.46	856.44	856.44
$\omega_{11b}(e_{1g})$	867.18	856.44	856.44
$\omega_{12a}(e_{1u})$	3210.35	3198.33	3198.33
$\omega_{12b}(e_{1u})$	3205.93	3198.32	3198.32
$\omega_{13a}(e_{1u})$	1509.58	1506.94	1506.95
$\omega_{13b}(e_{1u})$	1505.76	1506.91	1506.91
$\omega_{14a}(e_{1u})$	1054.26	1054.44	1054.44
$\omega_{14b}(e_{1u})$	1053.31	1054.39	1054.37
$\omega_{15a}(e_{2g})$	3197.58	3180.76	3180.76
$\omega_{15b}(e_{2g})$	3188.11	3180.64	3180.64
$\omega_{16a}(e_{2g})$	1630.26	1637.28	1637.28
$\omega_{16b}(e_{2g})$	1628.96	1637.20	1637.20
$\omega_{17a}(e_{2g})$	1201.32	1190.65	1190.65
$\omega_{17b}(e_{2g})$	1192.60	1190.59	1190.59
$\omega_{18a}(e_{2g})$	614.75	607.19	607.19
$\omega_{18b}(e_{2g})$	612.79	607.16	607.16
$\omega_{19a}(e_{2u})$	984.82	959.07	959.07
$\omega_{19b}(e_{2u})$	978.47	959.04	959.04
$\omega_{20a}(e_{2u})$	411.47	401.44	401.44
$\omega_{20b}(e_{2u})$	406.67	401.37	401.37

Table S20: Symmetrized, unnormalized natural internal coordinates for benzene.

1	$r_{1,2} + r_{2,3} + r_{3,4} + r_{4,5} + r_{5,6} + r_{6,1}$
2	$r_{1,2} - r_{2,3} + r_{3,4} - r_{4,5} + r_{5,6} - r_{6,1}$
3	$2r_{1,2} + r_{2,3} - r_{3,4} - 2r_{4,5} - r_{5,6} + r_{6,1}$
4	$r_{2,3} + r_{3,4} - r_{5,6} - r_{6,1}$
5	$2r_{1,2} - r_{2,3} - r_{3,4} + 2r_{4,5} - r_{5,6} - r_{6,1}$
6	$r_{2,3} - r_{3,4} + r_{5,6} - r_{6,1}$
7	$r_{1,7} + r_{2,8} + r_{3,9} + r_{4,10} + r_{5,11} + r_{6,12}$
8	$r_{1,7} - r_{2,8} + r_{3,9} - r_{4,10} + r_{5,11} - r_{6,12}$
9	$2r_{1,7} + r_{2,8} - r_{3,9} - 2r_{4,10} - r_{5,11} + r_{6,12}$
10	$r_{2,8} + r_{3,9} - r_{5,11} - r_{6,12}$
11	$2r_{1,7} - r_{2,8} - r_{3,9} + 2r_{4,10} - r_{5,11} - r_{6,12}$
12	$r_{2,8} - r_{3,9} + r_{5,11} - r_{6,12}$
13	$\phi_{6,1,2} - \phi_{1,2,3} + \phi_{2,3,4} - \phi_{3,4,5} + \phi_{4,5,6} - \phi_{5,6,1}$
14	$2\phi_{6,1,2} - \phi_{1,2,3} - \phi_{2,3,4} + 2\phi_{3,4,5} - \phi_{4,5,6} - \phi_{5,6,1}$
15	$\phi_{1,2,3} - \phi_{2,3,4} + \phi_{4,5,6} - \phi_{5,6,1}$
16	$\phi_{7,1,2} - \phi_{7,1,6} + \phi_{8,2,3} - \phi_{8,2,1} + \phi_{9,3,4} - \phi_{9,3,2} + \phi_{10,4,5} - \phi_{10,4,3} + \phi_{11,5,6} - \phi_{11,5,4}$ $+\phi_{12,6,1} - \phi_{12,6,5}$
17	$\phi_{7,1,2} - \phi_{7,1,6} - \phi_{8,2,3} + \phi_{8,2,1} + \phi_{9,3,4} - \phi_{9,3,2} - \phi_{10,4,5} + \phi_{10,4,3} + \phi_{11,5,6} - \phi_{11,5,4}$ $-\phi_{12,6,1} + \phi_{12,6,5}$
18	$2\phi_{7,1,2} - 2\phi_{7,1,6} + \phi_{8,2,3} - \phi_{8,2,1} - \phi_{9,3,4} + \phi_{9,3,2} - 2\phi_{10,4,5} + 2\phi_{10,4,3} - \phi_{11,5,6} + \phi_{11,5,4}$ $+\phi_{12,6,1} - \phi_{12,6,5}$
19	$\phi_{8,2,3} - \phi_{8,2,1} + \phi_{9,3,4} - \phi_{9,3,2} - \phi_{11,5,6} + \phi_{11,5,4} - \phi_{12,6,1} + \phi_{12,6,5}$
20	$2\phi_{7,1,2} - 2\phi_{7,1,6} - \phi_{8,2,3} + \phi_{8,2,1} - \phi_{9,3,4} + \phi_{9,3,2} + 2\phi_{10,4,5} - 2\phi_{10,4,3} - \phi_{11,5,6} + \phi_{11,5,4}$ $-\phi_{12,6,1} + \phi_{12,6,5}$
21	$\phi_{8,2,3} - \phi_{8,2,1} - \phi_{9,3,4} + \phi_{9,3,2} + \phi_{11,5,6} - \phi_{11,5,4} - \phi_{12,6,1} + \phi_{12,6,5}$
22	$\tau_{1,2,3,4} - \tau_{2,3,4,5} + \tau_{3,4,5,6} - \tau_{4,5,6,1} + \tau_{5,6,1,2} - \tau_{6,1,2,3}$
23	$\tau_{1,2,3,4} - \tau_{3,4,5,6} + \tau_{4,5,6,1} - \tau_{6,1,2,3}$
24	$-\tau_{1,2,3,4} + 2\tau_{2,3,4,5} - \tau_{3,4,5,6} - \tau_{4,5,6,1} + 2\tau_{5,6,1,2} - \tau_{6,1,2,3}$
25	$\gamma_{7,1,2,6} + \gamma_{8,2,3,1} + \gamma_{9,3,4,2} + \gamma_{10,4,5,3} + \gamma_{11,5,6,4} + \gamma_{12,6,1,5}$
26	$\gamma_{7,1,2,6} - \gamma_{8,2,3,1} + \gamma_{9,3,4,2} - \gamma_{10,4,5,3} + \gamma_{11,5,6,4} - \gamma_{12,6,1,5}$
27	$2\gamma_{7,1,2,6} + \gamma_{8,2,3,1} - \gamma_{9,3,4,2} - 2\gamma_{10,4,5,3} - \gamma_{11,5,6,4} + \gamma_{12,6,1,5}$
28	$\gamma_{8,2,3,1} + \gamma_{9,3,4,2} - \gamma_{11,5,6,4} - \gamma_{12,6,1,5}$
29	$2\gamma_{7,1,2,6} - \gamma_{8,2,3,1} - \gamma_{9,3,4,2} + 2\gamma_{10,4,5,3} - \gamma_{11,5,6,4} - \gamma_{12,6,1,5}$
30	$\gamma_{8,2,3,1} - \gamma_{9,3,4,2} + \gamma_{11,5,6,4} - \gamma_{12,6,1,5}$

S4.6 cyclopropene

Table S21: CCSD(T)/cc-pVTZ Optimum Cartesian coordinates (bohr)

1	C	−0.00000000	−0.00000000	1.67420069
2	H	0.00001614	−1.72642240	2.78915742
3	H	−0.00001614	1.72642240	2.78915742
4	C	1.22917808	0.00000291	−0.90945117
5	C	−1.22917808	−0.00000291	−0.90945117
6	H	2.98721276	−0.00000494	−1.92768856
7	H	−2.98721276	0.00000494	−1.92768856

Table S22: Reference [CCSD(T)/cc-pVTZ] CMA-0A and CMA-2A (ξ = 0.02) harmonic frequencies for Level B = MP2/cc-pVTZ and CCSD(T)/cc-pVDZ.

	Reference CCSD(T)/ cc-pVTZ	Pure MP2/ cc-pVTZ	CMA-0A MP2/ cc-pVTZ	CMA-2A MP2/ cc-pVTZ	Pure CCSD(T)/ cc-pVDZ	CMA-0A CCSD(T)/ cc-pVDZ	CMA-2A CCSD(T)/ cc-pVDZ
$\omega_1(a_1)$	3310.75	3326.62	3310.74	3310.74	3455.70	3310.74	3310.74
$\omega_2(a_1)$	3071.86	3088.42	3071.85	3071.85	3215.34	3071.83	3071.83
$\omega_3(a_1)$	1682.96	1677.34	1682.94	1682.96	1762.81	1682.20	1682.95
$\omega_4(a_1)$	1524.77	1526.62	1524.75	1524.72	1542.79	1525.33	1524.51
$\omega_5(a_1)$	1158.16	1153.88	1158.16	1158.23	1204.62	1158.19	1158.55
$\omega_6(a_1)$	927.46	929.75	927.54	927.46	941.05	927.98	927.53
$\omega_7(a_2)$	1020.59	1023.63	1020.28	1020.59	1028.28	1020.58	1020.59
$\omega_8(a_2)$	824.74	838.84	825.12	824.74	834.90	824.74	824.74
$\omega_9(b_1)$	3143.03	3167.07	3143.02	3143.02	3299.00	3143.02	3143.02
$\omega_{10}(b_1)$	1108.96	1111.63	1108.97	1108.97	1119.07	1108.96	1108.96
$\omega_{11}(b_1)$	575.68	584.69	575.69	575.68	588.05	575.70	575.70
$\omega_{12}(b_2)$	3264.57	3280.37	3264.57	3264.57	3410.21	3264.57	3264.57
$\omega_{13}(b_2)$	1074.23	1064.44	1073.27	1073.31	1087.58	1072.83	1072.83
$\omega_{14}(b_2)$	1042.08	1033.50	1042.89	1042.89	1057.50	1042.77	1043.19
$\omega_{15}(b_2)$	791.69	790.47	791.95	791.89	813.75	792.70	792.13

Table S23: CMA-0A and CMA-2A (ξ = 0.02) harmonic frequencies targeting CCSD(T)/cc-pVTZ for Level B = B3LYP/6-31G(2*df*,*p*).

	Pure B3LYP/ 6-31G(2*df*,*p*)	CMA-0A B3LYP/ 6-31G(2*df*,*p*)	CMA-2A B3LYP/ 6-31G(2*df*,*p*)
$\omega_1(a_1)$	3322.40	3310.74	3310.74
$\omega_2(a_1)$	3079.98	3071.82	3071.82
$\omega_3(a_1)$	1697.21	1682.89	1682.93
$\omega_4(a_1)$	1520.62	1524.73	1524.69
$\omega_5(a_1)$	1149.08	1158.35	1158.36
$\omega_6(a_1)$	935.81	927.54	927.52
$\omega_7(a_2)$	1019.44	1020.59	1020.59
$\omega_8(a_2)$	867.26	824.74	824.74
$\omega_9(b_1)$	3153.06	3143.03	3143.03
$\omega_{10}(b_1)$	1116.51	1108.91	1108.91
$\omega_{11}(b_1)$	622.99	575.79	575.79
$\omega_{12}(b_2)$	3277.11	3264.56	3264.56
$\omega_{13}(b_2)$	1080.52	1073.70	1073.70
$\omega_{14}(b_2)$	1035.81	1042.62	1042.62
$\omega_{15}(b_2)$	785.27	791.74	791.74

Table S24: Symmetrized, unnormalized natural internal coordinates for cyclopropene.

1	$r_{1,4} + r_{1,5} + r_{4,5}$
2	$r_{1,4} - r_{1,5}$
3	$-r_{1,4} - r_{1,5} + 2r_{4,5}$
4	$r_{1,2} + r_{1,3}$
5	$r_{1,2} - r_{1,3}$
6	$r_{4,6} + r_{5,7}$
7	$r_{4,6} - r_{5,7}$
8	$4\phi_{2,1,3} - \phi_{3,1,4} - \phi_{3,1,5} - \phi_{2,1,4} - \phi_{2,1,5}$
9	$\phi_{3,1,4} + \phi_{3,1,5} - \phi_{2,1,4} - \phi_{2,1,5}$
10	$\phi_{3,1,4} - \phi_{3,1,5} + \phi_{2,1,4} - \phi_{2,1,5}$
11	$\phi_{3,1,4} - \phi_{3,1,5} - \phi_{2,1,4} + \phi_{2,1,5}$
12	$\phi_{6,4,1} - \phi_{6,4,5} + \phi_{7,5,1} - \phi_{7,5,4}$
13	$\phi_{6,4,1} - \phi_{6,4,5} - \phi_{7,5,1} + \phi_{7,5,4}$
14	$\tau_{7,5,4,6}$
15	$\gamma_{6,4,1,5} - \gamma_{7,5,1,4}$

S4.7 allene

Table S25: CCSD(T)/cc-pVTZ Optimum Cartesian coordinates (bohr)

1	C	0.00000000	0.00000000	−0.00000001
2	C	0.00000000	0.00000000	−2.48178976
3	C	0.00000000	0.00000000	2.48178971
4	H	−1.75641355	0.00000000	−3.53278972
5	H	1.75641355	0.00000000	−3.53278972
6	H	0.00000000	−1.75641338	3.53279007
7	H	0.00000000	1.75641338	3.53279007

Table S26: Reference [CCSD(T)/cc-pVTZ] CMA-0A and CMA-2A (ξ = 0.02) harmonic frequencies for Level B = MP2/cc-pVTZ and CCSD(T)/cc-pVDZ.

	Reference CCSD(T)/ cc-pVTZ	Pure MP2/ cc-pVTZ	CMA-0A MP2/ cc-pVTZ	CMA-2A MP2/ cc-pVTZ	Pure CCSD(T)/ cc-pVDZ	CMA-0A CCSD(T)/ cc-pVDZ	CMA-2A CCSD(T)/ cc-pVDZ
$\omega_1(a_1)$	3144.28	3159.70	3144.28	3144.28	3283.15	3144.27	3144.27
$\omega_2(a_1)$	1488.52	1481.35	1488.52	1488.52	1516.96	1487.82	1488.53
$\omega_3(a_1)$	1080.75	1082.06	1080.76	1080.76	1122.49	1081.75	1080.77
$\omega_4(b_1)$	870.31	892.05	870.31	870.31	875.85	870.31	870.31
$\omega_5(b_2)$	3142.75	3159.37	3142.75	3142.75	3282.49	3142.72	3142.72
$\omega_6(b_2)$	2012.15	2009.82	2012.12	2012.15	2109.08	2012.20	2012.20
$\omega_7(b_2)$	1438.38	1433.30	1438.41	1438.38	1455.27	1438.38	1438.38
$\omega_{8\mathrm{a}}(e)$	3226.41	3247.74	3226.41	3226.41	3382.45	3226.40	3226.40
$\omega_{8\mathrm{b}}(e)$	3226.41	3247.56	3226.41	3226.41	3382.27	3226.40	3226.40
$\omega_{9\mathrm{a}}(e)$	1017.78	1011.11	1017.70	1017.70	1036.54	1017.83	1017.83
$\omega_{9\mathrm{b}}(e)$	1017.66	1011.09	1017.57	1017.57	1036.54	1017.70	1017.70
$\omega_{10\mathrm{a}}(e)$	856.73	855.14	856.73	856.73	864.54	856.74	856.74
$\omega_{10\mathrm{b}}(e)$	856.73	855.11	856.73	856.73	864.50	856.74	856.74
$\omega_{11\mathrm{a}}(e)$	347.95	356.61	348.21	348.20	356.67	347.96	347.95
$\omega_{11\mathrm{b}}(e)$	347.95	356.60	348.20	348.20	356.66	347.96	347.95

Table S27: CMA-0A and CMA-2A (ξ = 0.02) harmonic frequencies targeting CCSD(T)/cc-pVTZ for Level B = B3LYP/6-31G(2df,p).

	Pure B3LYP/ 6-31G(2df,p)	CMA-0A B3LYP/ 6-31G(2df,p)	CMA-2A B3LYP/ 6-31G(2df,p)
$\omega_1(a_1)$	3155.65	3144.28	3144.28
$\omega_2(a_1)$	1471.11	1488.46	1488.46
$\omega_3(a_1)$	1087.83	1080.85	1080.85
$\omega_4(b_1)$	880.58	870.31	870.31
$\omega_5(b_2)$	3151.46	3142.66	3142.66
$\omega_6(b_2)$	2010.71	2012.19	2012.29
$\omega_7(b_2)$	1422.88	1438.51	1438.38
$\omega_{8\mathrm{a}}(e)$	3239.52	3226.41	3226.41
$\omega_{8\mathrm{b}}(e)$	3239.38	3226.41	3226.41
$\omega_{9\mathrm{a}}(e)$	1019.56	1017.52	1017.52
$\omega_{9\mathrm{b}}(e)$	1019.49	1017.40	1017.40
$\omega_{10\mathrm{a}}(e)$	863.17	856.86	856.86
$\omega_{10\mathrm{b}}(e)$	863.07	856.85	856.85
$\omega_{11\mathrm{a}}(e)$	368.94	348.42	348.42
$\omega_{11\mathrm{b}}(e)$	368.91	348.41	348.41

Table S28: Symmetrized, unnormalized natural internal coordinates for allene.

1	$r_{1,2} + r_{1,3}$
2	$r_{1,2} - r_{1,3}$
3	$r_{2,4} + r_{2,5} + r_{3,6} + r_{3,7}$
4	$r_{2,4} + r_{2,5} - r_{3,6} - r_{3,7}$
5	$r_{2,4} - r_{2,5} + r_{3,6} - r_{3,7}$
6	$r_{2,4} - r_{2,5} - r_{3,6} + r_{3,7}$
7	$2\phi_{4,2,5} - \phi_{1,2,4} - \phi_{1,2,5} + 2\phi_{6,3,7} - \phi_{1,3,6} - \phi_{1,3,7}$
8	$2\phi_{4,2,5} - \phi_{1,2,4} - \phi_{1,2,5} - 2\phi_{6,3,7} + \phi_{1,3,6} + \phi_{1,3,7}$
9	$\phi_{1,2,4} - \phi_{1,2,5} + \phi_{1,3,6} - \phi_{1,3,7}$
10	$\phi_{1,2,4} - \phi_{1,2,5} - \phi_{1,3,6} + \phi_{1,3,7}$
11	$\tau_{4,2,3,6} + \tau_{4,2,3,7} + \tau_{5,2,3,6} + \tau_{5,2,3,7}$
12	$\gamma_{1,2,4,5} + \gamma_{1,3,7,6}$
13	$\gamma_{1,2,4,5} - \gamma_{1,3,7,6}$
14	$\alpha^x_{4,2,1,3} - \alpha^x_{5,2,1,3} + \alpha^x_{6,3,1,2} - \alpha^x_{7,3,1,2}$
15	$\alpha^x_{4,2,1,3} - \alpha^x_{5,2,1,3} - \alpha^x_{6,3,1,2} + \alpha^x_{7,3,1,2}$

S4.8 spiropentane

Table S29: CCSD(T)/cc-pVTZ Optimum Cartesian coordinates (bohr)

1	C	0.00000000	−0.00000000	0.00000000
2	C	−1.02524750	−1.02524750	−2.39918091
3	C	1.02524750	1.02524750	−2.39918091
4	C	1.02524750	−1.02524750	2.39918091
5	C	−1.02524750	1.02524750	2.39918091
6	H	−0.46577708	−2.91033012	−2.96611806
7	H	−2.91033012	−0.46577708	−2.96611806
8	H	0.46577708	2.91033012	−2.96611806
9	H	2.91033012	0.46577708	−2.96611806
10	H	0.46577708	−2.91033012	2.96611806
11	H	2.91033012	−0.46577708	2.96611806
12	H	−0.46577708	2.91033012	2.96611806
13	H	−2.91033012	0.46577708	2.96611806

Table S30: Reference [CCSD(T)/cc-pVTZ] CMA-0A and CMA-2A (ξ = 0.02) harmonic frequencies for Level B = MP2/cc-pVTZ and CCSD(T)/cc-pVDZ.

	Reference CCSD(T)/ cc-pVTZ	Pure MP2/ cc-pVTZ	CMA-0A MP2/ cc-pVTZ	CMA-2A MP2/ cc-pVTZ	Pure CCSD(T)/ cc-pVDZ	CMA-0A CCSD(T)/ cc-pVDZ	CMA-2A CCSD(T)/ cc-pVDZ
$\omega_1(a_1)$	3134.85	3148.68	3134.85	3134.85	3271.74	3134.84	3134.84
$\omega_2(a_1)$	1501.43	1497.87	1501.40	1501.40	1520.29	1501.04	1501.04
$\omega_3(a_1)$	1076.02	1067.65	1072.42	1076.03	1111.28	1074.20	1076.31
$\omega_4(a_1)$	1054.41	1050.78	1058.08	1054.44	1074.83	1056.75	1054.60
$\omega_5(a_1)$	598.43	599.03	598.50	598.44	619.67	598.62	598.62
$\omega_6(a_2)$	3213.22	3236.81	3213.22	3213.22	3362.25	3213.22	3213.22
$\omega_7(a_2)$	1173.46	1171.62	1173.46	1173.46	1183.96	1173.45	1173.45
$\omega_8(a_2)$	840.13	838.36	840.14	840.14	846.55	840.17	840.17
$\omega_9(b_1)$	3212.32	3235.55	3212.32	3212.32	3361.43	3212.31	3212.31
$\omega_{10}(b_1)$	1183.50	1180.66	1183.45	1183.45	1193.46	1183.49	1183.49
$\omega_{11}(b_1)$	1027.32	1027.54	1027.39	1027.39	1036.53	1027.35	1027.35
$\omega_{12}(b_1)$	293.06	292.70	293.07	293.07	295.76	293.08	293.08
$\omega_{13}(b_2)$	3135.93	3151.11	3135.93	3135.93	3272.51	3135.91	3135.91
$\omega_{14}(b_2)$	1596.21	1586.54	1596.15	1596.16	1659.32	1595.02	1596.23
$\omega_{15}(b_2)$	1439.26	1439.83	1439.24	1439.23	1457.83	1440.45	1439.11
$\omega_{16}(b_2)$	1019.25	1006.35	1018.43	1019.33	1035.37	1018.33	1018.33
$\omega_{17}(b_2)$	900.83	898.59	901.91	900.88	935.80	902.14	902.14
$\omega_{18a}(e)$	3225.32	3247.93	3225.32	3225.32	3374.18	3225.32	3225.32
$\omega_{18b}(e)$	3225.32	3247.83	3225.32	3225.32	3374.09	3225.31	3225.31
$\omega_{19a}(e)$	3130.04	3146.14	3130.04	3130.04	3265.46	3130.02	3130.02
$\omega_{19b}(e)$	3130.04	3146.05	3130.04	3130.04	3265.38	3130.02	3130.02
$\omega_{20a}(e)$	1468.81	1467.34	1468.78	1468.78	1480.45	1468.73	1468.73
$\omega_{20b}(e)$	1468.81	1467.34	1468.78	1468.78	1480.45	1468.73	1468.73
$\omega_{21a}(e)$	1189.88	1183.01	1189.86	1189.87	1211.60	1188.84	1189.47
$\omega_{21b}(e)$	1189.88	1183.00	1189.86	1189.86	1211.59	1188.84	1189.47
$\omega_{22a}(e)$	1077.33	1064.21	1077.35	1077.35	1091.73	1077.58	1077.58
$\omega_{22b}(e)$	1077.33	1064.14	1077.35	1077.35	1091.65	1077.58	1077.58
$\omega_{23a}(e)$	897.42	896.84	897.50	897.50	925.98	898.68	897.85
$\omega_{23b}(e)$	897.42	896.82	897.50	897.49	925.96	898.68	897.85
$\omega_{24a}(e)$	786.27	787.58	786.27	786.27	797.89	786.36	786.36
$\omega_{24b}(e)$	786.27	787.57	786.27	786.27	797.88	786.36	786.36
$\omega_{25a}(e)$	297.15	293.94	297.18	297.17	304.44	297.16	297.16
$\omega_{25b}(e)$	297.15	293.91	297.18	297.17	304.39	297.16	297.16

Table S31: CMA-0A and CMA-2A (ξ = 0.02) harmonic frequencies targeting CCSD(T)/cc-pVTZ for Level B = B3LYP/6-31G(2*df*,*p*).

	Pure B3LYP/ 6-31G(2*df*,*p*)	CMA-0A B3LYP/ 6-31G(2*df*,*p*)	CMA-2A B3LYP/ 6-31G(2*df*,*p*)
$\omega_1(a_1)$	3144.84	3134.82	3134.82
$\omega_2(a_1)$	1491.71	1501.40	1501.40
$\omega_3(a_1)$	1079.37	1075.75	1076.06
$\omega_4(a_1)$	1053.65	1054.78	1054.47
$\omega_5(a_1)$	601.29	598.48	598.48
$\omega_6(a_2)$	3224.73	3213.22	3213.22
$\omega_7(a_2)$	1171.84	1173.45	1173.45
$\omega_8(a_2)$	841.71	840.15	840.15
$\omega_9(b_1)$	3223.17	3212.32	3212.32
$\omega_{10}(b_1)$	1181.33	1183.49	1183.49
$\omega_{11}(b_1)$	1026.79	1027.33	1027.33
$\omega_{12}(b_1)$	291.53	293.08	293.08
$\omega_{13}(b_2)$	3143.75	3135.87	3135.87
$\omega_{14}(b_2)$	1570.28	1596.25	1596.28
$\omega_{15}(b_2)$	1432.97	1439.24	1439.21
$\omega_{16}(b_2)$	1024.15	1019.28	1019.29
$\omega_{17}(b_2)$	896.49	900.98	900.96
$\omega_{18\mathrm{a}}(e)$	3238.44	3225.33	3225.32
$\omega_{18\mathrm{b}}(e)$	3237.91	3225.29	3225.32
$\omega_{19\mathrm{a}}(e)$	3140.14	3130.04	3130.04
$\omega_{19\mathrm{b}}(e)$	3139.60	3130.03	3130.04
$\omega_{20\mathrm{a}}(e)$	1463.30	1468.72	1468.81
$\omega_{20\mathrm{b}}(e)$	1462.26	1468.71	1468.81
$\omega_{21\mathrm{a}}(e)$	1182.41	1189.82	1189.88
$\omega_{21\mathrm{b}}(e)$	1181.22	1189.81	1189.88
$\omega_{22\mathrm{a}}(e)$	1080.64	1077.39	1077.33
$\omega_{22\mathrm{b}}(e)$	1080.43	1077.34	1077.33
$\omega_{23\mathrm{a}}(e)$	888.36	897.65	897.42
$\omega_{23\mathrm{b}}(e)$	887.91	897.60	897.42
$\omega_{24\mathrm{a}}(e)$	786.42	786.35	786.27
$\omega_{24\mathrm{b}}(e)$	785.22	786.34	786.27
$\omega_{25\mathrm{a}}(e)$	308.71	297.18	297.15
$\omega_{25\mathrm{b}}(e)$	308.44	297.17	297.15

Table S32: Symmetrized, unnormalized natural internal coordinates for spiropentane.

1	$r_{1,2} + r_{1,3} + r_{2,3} + r_{1,4} + r_{1,5} + r_{4,5}$
2	$r_{1,2} + r_{1,3} + r_{2,3} - r_{1,4} - r_{1,5} - r_{4,5}$
3	$-r_{1,2} - r_{1,3} + 2r_{2,3} - r_{1,4} - r_{1,5} + 2r_{4,5}$
4	$-r_{1,2} - r_{1,3} + 2r_{2,3} + r_{1,4} + r_{1,5} - 2r_{4,5}$
5	$r_{1,2} - r_{1,3} + r_{1,4} - r_{1,5}$
6	$r_{1,2} - r_{1,3} - r_{1,4} + r_{1,5}$
7	$r_{2,6} + r_{2,7} + r_{3,8} + r_{3,9} + r_{4,10} + r_{4,11} + r_{5,12} + r_{5,13}$
8	$r_{2,6} + r_{2,7} + r_{3,8} + r_{3,9} - r_{4,10} - r_{4,11} - r_{5,12} - r_{5,13}$
9	$r_{2,6} + r_{2,7} - r_{3,8} - r_{3,9} + r_{4,10} + r_{4,11} - r_{5,12} - r_{5,13}$
10	$r_{2,6} + r_{2,7} - r_{3,8} - r_{3,9} - r_{4,10} - r_{4,11} + r_{5,12} + r_{5,13}$
11	$r_{2,6} - r_{2,7} + r_{3,8} - r_{3,9} + r_{4,10} - r_{4,11} + r_{5,12} - r_{5,13}$
12	$r_{2,6} - r_{2,7} + r_{3,8} - r_{3,9} - r_{4,10} + r_{4,11} - r_{5,12} + r_{5,13}$
13	$r_{2,6} - r_{2,7} - r_{3,8} + r_{3,9} + r_{4,10} - r_{4,11} - r_{5,12} + r_{5,13}$
14	$r_{2,6} - r_{2,7} - r_{3,8} + r_{3,9} - r_{4,10} + r_{4,11} + r_{5,12} - r_{5,13}$
15	$\phi_{2,1,4} + \phi_{3,1,4} - \phi_{2,1,5} - \phi_{3,1,5}$
16	$\phi_{2,1,4} - \phi_{3,1,4} + \phi_{2,1,5} - \phi_{3,1,5}$
17	$\phi_{2,1,4} - \phi_{3,1,4} - \phi_{2,1,5} + \phi_{3,1,5}$
18	$4\phi_{6,2,7} - \phi_{6,2,1} - \phi_{6,2,3} - \phi_{7,2,1} - \phi_{7,2,3} + 4\phi_{8,3,9} - \phi_{8,3,1} - \phi_{8,3,2} - \phi_{9,3,1} - \phi_{9,3,2}$ $+4\phi_{10,4,11} - \phi_{10,4,1} - \phi_{10,4,5} - \phi_{11,4,1} - \phi_{11,4,5} + 4\phi_{12,5,13} - \phi_{12,5,1} - \phi_{12,5,4} - \phi_{13,5,1} - \phi_{13,5,4}$
19	$4\phi_{6,2,7} - \phi_{6,2,1} - \phi_{6,2,3} - \phi_{7,2,1} - \phi_{7,2,3} + 4\phi_{8,3,9} - \phi_{8,3,1} - \phi_{8,3,2} - \phi_{9,3,1} - \phi_{9,3,2}$ $-4\phi_{10,4,11} + \phi_{10,4,1} + \phi_{10,4,5} + \phi_{11,4,1} + \phi_{11,4,5} - 4\phi_{12,5,13} + \phi_{12,5,1} + \phi_{12,5,4} + \phi_{13,5,1} + \phi_{13,5,4}$
20	$4\phi_{6,2,7} - \phi_{6,2,1} - \phi_{6,2,3} - \phi_{7,2,1} - \phi_{7,2,3} - 4\phi_{8,3,9} + \phi_{8,3,1} + \phi_{8,3,2} + \phi_{9,3,1} + \phi_{9,3,2}$ $+4\phi_{10,4,11} - \phi_{10,4,1} - \phi_{10,4,5} - \phi_{11,4,1} - \phi_{11,4,5} - 4\phi_{12,5,13} + \phi_{12,5,1} + \phi_{12,5,4} + \phi_{13,5,1} + \phi_{13,5,4}$
21	$4\phi_{6,2,7} - \phi_{6,2,1} - \phi_{6,2,3} - \phi_{7,2,1} - \phi_{7,2,3} - 4\phi_{8,3,9} + \phi_{8,3,1} + \phi_{8,3,2} + \phi_{9,3,1} + \phi_{9,3,2}$ $-4\phi_{10,4,11} + \phi_{10,4,1} + \phi_{10,4,5} + \phi_{11,4,1} + \phi_{11,4,5} + 4\phi_{12,5,13} - \phi_{12,5,1} - \phi_{12,5,4} - \phi_{13,5,1} - \phi_{13,5,4}$
22	$\phi_{6,2,1} + \phi_{6,2,3} - \phi_{7,2,1} - \phi_{7,2,3} + \phi_{8,3,1} + \phi_{8,3,2} - \phi_{9,3,1} - \phi_{9,3,2} + \phi_{10,4,1} + \phi_{10,4,5}$ $-\phi_{11,4,1} - \phi_{11,4,5} + \phi_{12,5,1} + \phi_{12,5,4} - \phi_{13,5,1} - \phi_{13,5,4}$
23	$\phi_{6,2,1} + \phi_{6,2,3} - \phi_{7,2,1} - \phi_{7,2,3} + \phi_{8,3,1} + \phi_{8,3,2} - \phi_{9,3,1} - \phi_{9,3,2} - \phi_{10,4,1} - \phi_{10,4,5}$ $+\phi_{11,4,1} + \phi_{11,4,5} - \phi_{12,5,1} - \phi_{12,5,4} + \phi_{13,5,1} + \phi_{13,5,4}$
24	$\phi_{6,2,1} + \phi_{6,2,3} - \phi_{7,2,1} - \phi_{7,2,3} - \phi_{8,3,1} - \phi_{8,3,2} + \phi_{9,3,1} + \phi_{9,3,2} + \phi_{10,4,1} + \phi_{10,4,5}$ $-\phi_{11,4,1} - \phi_{11,4,5} - \phi_{12,5,1} - \phi_{12,5,4} + \phi_{13,5,1} + \phi_{13,5,4}$
25	$\phi_{6,2,1} + \phi_{6,2,3} - \phi_{7,2,1} - \phi_{7,2,3} - \phi_{8,3,1} - \phi_{8,3,2} + \phi_{9,3,1} + \phi_{9,3,2} - \phi_{10,4,1} - \phi_{10,4,5}$ $+\phi_{11,4,1} + \phi_{11,4,5} + \phi_{12,5,1} + \phi_{12,5,4} - \phi_{13,5,1} - \phi_{13,5,4}$
26	$\phi_{6,2,1} - \phi_{6,2,3} + \phi_{7,2,1} - \phi_{7,2,3} + \phi_{8,3,1} - \phi_{8,3,2} + \phi_{9,3,1} - \phi_{9,3,2} + \phi_{10,4,1} - \phi_{10,4,5}$ $+\phi_{11,4,1} - \phi_{11,4,5} + \phi_{12,5,1} - \phi_{12,5,4} + \phi_{13,5,1} - \phi_{13,5,4}$
27	$\phi_{6,2,1} - \phi_{6,2,3} + \phi_{7,2,1} - \phi_{7,2,3} + \phi_{8,3,1} - \phi_{8,3,2} + \phi_{9,3,1} - \phi_{9,3,2} - \phi_{10,4,1} + \phi_{10,4,5}$ $-\phi_{11,4,1} + \phi_{11,4,5} - \phi_{12,5,1} + \phi_{12,5,4} - \phi_{13,5,1} + \phi_{13,5,4}$
28	$\phi_{6,2,1} - \phi_{6,2,3} + \phi_{7,2,1} - \phi_{7,2,3} - \phi_{8,3,1} + \phi_{8,3,2} - \phi_{9,3,1} + \phi_{9,3,2} + \phi_{10,4,1} - \phi_{10,4,5}$ $+\phi_{11,4,1} - \phi_{11,4,5} - \phi_{12,5,1} + \phi_{12,5,4} - \phi_{13,5,1} + \phi_{13,5,4}$
29	$\phi_{6,2,1} - \phi_{6,2,3} + \phi_{7,2,1} - \phi_{7,2,3} - \phi_{8,3,1} + \phi_{8,3,2} - \phi_{9,3,1} + \phi_{9,3,2} - \phi_{10,4,1} + \phi_{10,4,5}$ $-\phi_{11,4,1} + \phi_{11,4,5} + \phi_{12,5,1} - \phi_{12,5,4} + \phi_{13,5,1} - \phi_{13,5,4}$
30	$\phi_{6,2,1} - \phi_{6,2,3} - \phi_{7,2,1} + \phi_{7,2,3} + \phi_{8,3,1} - \phi_{8,3,2} - \phi_{9,3,1} + \phi_{9,3,2} + \phi_{10,4,1} - \phi_{10,4,5}$ $-\phi_{11,4,1} + \phi_{11,4,5} + \phi_{12,5,1} - \phi_{12,5,4} - \phi_{13,5,1} + \phi_{13,5,4}$
31	$\phi_{6,2,1} - \phi_{6,2,3} - \phi_{7,2,1} + \phi_{7,2,3} + \phi_{8,3,1} - \phi_{8,3,2} - \phi_{9,3,1} + \phi_{9,3,2} - \phi_{10,4,1} + \phi_{10,4,5}$ $+\phi_{11,4,1} - \phi_{11,4,5} - \phi_{12,5,1} + \phi_{12,5,4} + \phi_{13,5,1} - \phi_{13,5,4}$
32	$\phi_{6,2,1} - \phi_{6,2,3} - \phi_{7,2,1} + \phi_{7,2,3} - \phi_{8,3,1} + \phi_{8,3,2} + \phi_{9,3,1} - \phi_{9,3,2} + \phi_{10,4,1} - \phi_{10,4,5}$ $-\phi_{11,4,1} + \phi_{11,4,5} - \phi_{12,5,1} + \phi_{12,5,4} + \phi_{13,5,1} - \phi_{13,5,4}$
33	$\phi_{6,2,1} - \phi_{6,2,3} - \phi_{7,2,1} + \phi_{7,2,3} - \phi_{8,3,1} + \phi_{8,3,2} + \phi_{9,3,1} - \phi_{9,3,2} - \phi_{10,4,1} + \phi_{10,4,5}$ $+\phi_{11,4,1} - \phi_{11,4,5} + \phi_{12,5,1} - \phi_{12,5,4} - \phi_{13,5,1} + \phi_{13,5,4}$

S4.9 aluminum trichloride

Table S33: CCSD(T)/cc-pVTZ Optimum Cartesian coordinates (bohr)

1	Al	0.00000000	0.00000000	0.00000069
2	Cl	0.00000000	0.00000000	−3.92579486
3	Cl	0.00000000	3.39983903	1.96289716
4	Cl	0.00000000	−3.39983903	1.96289716

Table S34: Reference [CCSD(T)/cc-pVTZ] CMA-0A and CMA-2A (ξ = 0.02) harmonic frequencies for Level B = MP2/cc-pVTZ and CCSD(T)/cc-pVDZ.

	Reference CCSD(T)/ cc-pVTZ	Pure MP2/ cc-pVTZ	CMA-0A MP2/ cc-pVTZ	CMA-2A MP2/ cc-pVTZ	Pure CCSD(T)/ cc-pVDZ	CMA-0A CCSD(T)/ cc-pVDZ	CMA-2A CCSD(T)/ cc-pVDZ
$\omega_1(a_1^{'})$	388.75	393.29	388.75	388.75	391.81	388.75	388.75
$\omega_{2a}(e^{'})$	628.44	636.71	628.44	628.44	639.39	628.44	628.44
$\omega_{2b}(e^{'})$	628.44	636.70	628.44	628.44	639.38	628.44	628.44
$\omega_{3a}(e^{'})$	147.29	146.42	147.29	147.29	152.03	147.29	147.29
$\omega_{3b}(e^{'})$	147.27	146.41	147.27	147.27	152.02	147.27	147.27
$\omega_4(a_2^{''})$	207.49	207.81	207.49	207.49	213.88	207.49	207.49

Table S35: CMA-0A and CMA-2A (ξ = 0.02) harmonic frequencies targeting CCSD(T)/cc-pVTZ for Level B = B3LYP/6-31G(2*df*,*p*).

	Pure B3LYP/ 6-31G(2*df*,*p*)	CMA-0A B3LYP/ 6-31G(2*df*,*p*)	CMA-2A B3LYP/ 6-31G(2*df*,*p*)
$\omega_1(a_1^{'})$	381.99	388.75	388.75
$\omega_{2a}(e^{'})$	631.04	628.42	628.46
$\omega_{2b}(e^{'})$	621.41	628.40	628.41
$\omega_{3a}(e^{'})$	153.90	147.50	147.30
$\omega_{3b}(e^{'})$	147.70	147.30	147.28
$\omega_4(a_2^{''})$	204.50	207.49	207.49

Table S36: Symmetrized, unnormalized natural internal coordinates for aluminum trichloride.

1	$r_{1,2} + r_{1,3} + r_{1,4}$
2	$2r_{1,2} - r_{1,3} - r_{1,4}$
3	$r_{1,3} - r_{1,4}$
4	$2\phi_{2,1,3} - \phi_{2,1,4} - \phi_{3,1,4}$
5	$\phi_{2,1,4} - \phi_{3,1,4}$
6	$\gamma_{2,1,3,4} + \gamma_{3,1,4,2} + \gamma_{4,1,2,3}$

S4.10 aluminum trifluoride

Table S37: CCSD(T)/cc-pVTZ Optimum Cartesian coordinates (bohr)

1	Al	0.00000000	0.00000000	0.00000081
2	F	0.00000000	0.00000000	−3.08479068
3	F	0.00000000	2.67150833	1.54239476
4	F	0.00000000	−2.67150833	1.54239476

Table S38: Reference [CCSD(T)/cc-pVTZ] CMA-0A and CMA-2A ($\xi = 0.02$) harmonic frequencies for Level B = MP2/cc-pVTZ and CCSD(T)/cc-pVDZ.

	Reference CCSD(T)/ cc-pVTZ	Pure MP2/ cc-pVTZ	CMA-0A MP2/ cc-pVTZ	CMA-2A MP2/ cc-pVTZ	Pure CCSD(T)/ cc-pVDZ	CMA-0A CCSD(T)/ cc-pVDZ	CMA-2A CCSD(T)/ cc-pVDZ
$\omega_1(a_1^{'})$	695.29	712.22	695.29	695.29	704.32	695.29	695.29
$\omega_{2\mathrm{a}}(e^{'})$	962.29	989.94	962.28	962.28	992.33	962.27	962.27
$\omega_{2\mathrm{b}}(e^{'})$	962.28	989.91	962.28	962.28	992.30	962.27	962.27
$\omega_{3\mathrm{a}}(e^{'})$	244.99	244.91	245.00	245.00	249.63	245.04	245.04
$\omega_{3\mathrm{b}}(e^{'})$	244.84	244.91	244.86	244.86	249.63	244.91	244.91
$\omega_4(a_2^{''})$	301.32	305.56	301.32	301.32	301.15	301.32	301.32

Table S39: CMA-0A and CMA-2A ($\xi = 0.02$) harmonic frequencies targeting CCSD(T)/cc-pVTZ for Level B = B3LYP/6-31G(2*df*,*p*).

	Pure B3LYP/ 6-31G(2*df*,*p*)	CMA-0A B3LYP/ 6-31G(2*df*,*p*)	CMA-2A B3LYP/ 6-31G(2*df*,*p*)
$\omega_1(a_1^{'})$	703.87	695.29	695.29
$\omega_{2\mathrm{a}}(e^{'})$	980.79	962.28	962.28
$\omega_{2\mathrm{b}}(e^{'})$	973.75	962.23	962.28
$\omega_{3\mathrm{a}}(e^{'})$	243.75	245.13	244.94
$\omega_{3\mathrm{b}}(e^{'})$	231.33	244.94	244.94
$\omega_4(a_2^{''})$	297.95	301.32	301.32

Table S40: Symmetrized, unnormalized natural internal coordinates for aluminum trifluoride.

1	$r_{1,2} + r_{1,3} + r_{1,4}$
2	$2r_{1,2} - r_{1,3} - r_{1,4}$
3	$r_{1,3} - r_{1,4}$
4	$2\phi_{2,1,3} - \phi_{2,1,4} - \phi_{3,1,4}$
5	$\phi_{2,1,4} - \phi_{3,1,4}$
6	$\gamma_{2,1,3,4} + \gamma_{3,1,4,2} + \gamma_{4,1,2,3}$

S4.11 boron trichloride

Table S41: CCSD(T)/cc-pVTZ Optimum Cartesian coordinates (bohr)

1	B	0.00000000	0.00000000	0.00000105
2	Cl	0.00000000	0.00000000	−3.29682188
3	Cl	0.00000000	2.85513240	1.64841078
4	Cl	0.00000000	−2.85513240	1.64841078

Table S42: Reference [CCSD(T)/cc-pVTZ] CMA-0A and CMA-2A (ξ = 0.02) harmonic frequencies for Level B = MP2/cc-pVTZ and CCSD(T)/cc-pVDZ.

	Reference CCSD(T)/ cc-pVTZ	Pure MP2/ cc-pVTZ	CMA-0A MP2/ cc-pVTZ	CMA-2A MP2/ cc-pVTZ	Pure CCSD(T)/ cc-pVDZ	CMA-0A CCSD(T)/ cc-pVDZ	CMA-2A CCSD(T)/ cc-pVDZ
$\omega_1(a_1^{'})$	476.79	478.43	476.79	476.79	492.48	476.79	476.79
$\omega_{2a}(e^{'})$	972.62	972.37	972.61	972.61	1020.71	972.61	972.61
$\omega_{2b}(e^{'})$	972.61	972.33	972.61	972.61	1020.68	972.61	972.61
$\omega_{3a}(e^{'})$	257.22	257.14	257.22	257.22	263.30	257.22	257.22
$\omega_{3b}(e^{'})$	257.16	257.14	257.17	257.17	263.29	257.17	257.17
$\omega_4(a_2^{''})$	462.52	461.94	462.52	462.52	470.84	462.52	462.52

Table S43: CMA-0A and CMA-2A (ξ = 0.02) harmonic frequencies targeting CCSD(T)/cc-pVTZ for Level B = B3LYP/6-31G(2*df*,*p*).

	Pure B3LYP/ 6-31G(2*df*,*p*)	CMA-0A B3LYP/ 6-31G(2*df*,*p*)	CMA-2A B3LYP/ 6-31G(2*df*,*p*)
$\omega_1(a_1^{'})$	474.80	476.79	476.79
$\omega_{2a}(e^{'})$	952.45	972.60	972.61
$\omega_{2b}(e^{'})$	950.35	972.59	972.60
$\omega_{3a}(e^{'})$	254.15	257.32	257.25
$\omega_{3b}(e^{'})$	250.13	257.20	257.20
$\omega_4(a_2^{''})$	457.50	462.52	462.52

Table S44: Symmetrized, unnormalized natural internal coordinates for boron trichloride.

1	$r_{1,2} + r_{1,3} + r_{1,4}$
2	$2r_{1,2} - r_{1,3} - r_{1,4}$
3	$r_{1,3} - r_{1,4}$
4	$2\phi_{2,1,3} - \phi_{2,1,4} - \phi_{3,1,4}$
5	$\phi_{2,1,4} - \phi_{3,1,4}$
6	$\gamma_{2,1,3,4} + \gamma_{3,1,4,2} + \gamma_{4,1,2,3}$

S4.12 boron trifluoride

Table S45: CCSD(T)/cc-pVTZ Optimum Cartesian coordinates (bohr)

1	B	0.00000000	0.00000000	−0.00000000
2	F	0.00000000	−2.14848994	1.24043124
3	F	0.00000000	−0.00000000	−2.48086249
4	F	−0.00000000	2.14848994	1.24043124

Table S46: Reference [CCSD(T)/cc-pVTZ] CMA-0A and CMA-2A (ξ = 0.02) harmonic frequencies for Level B = MP2/cc-pVTZ and CCSD(T)/cc-pVDZ.

	Reference CCSD(T)/ cc-pVTZ	Pure MP2/ cc-pVTZ	CMA-0A MP2/ cc-pVTZ	CMA-2A MP2/ cc-pVTZ	Pure CCSD(T)/ cc-pVDZ	CMA-0A CCSD(T)/ cc-pVDZ	CMA-2A CCSD(T)/ cc-pVDZ
$\omega_1(a_1^{'})$	899.30	900.36	899.30	899.30	905.94	899.30	899.30
$\omega_{2\mathrm{a}}(e^{'})$	1493.14	1493.05	1493.14	1493.14	1538.20	1493.14	1493.14
$\omega_{2\mathrm{b}}(e^{'})$	1493.14	1492.98	1493.14	1493.14	1538.14	1493.14	1493.14
$\omega_{3\mathrm{a}}(e^{'})$	483.70	483.55	483.71	483.71	498.44	483.71	483.71
$\omega_{3\mathrm{b}}(e^{'})$	483.70	483.54	483.71	483.71	498.43	483.71	483.71
$\omega_4(a_2^{''})$	700.10	700.36	700.10	700.10	732.21	700.10	700.10

Table S47: CMA-0A and CMA-2A (ξ = 0.02) harmonic frequencies targeting CCSD(T)/cc-pVTZ for Level B = B3LYP/6-31G(2*df*,*p*).

	Pure B3LYP/ 6-31G(2*df*,*p*)	CMA-0A B3LYP/ 6-31G(2*df*,*p*)	CMA-2A B3LYP/ 6-31G(2*df*,*p*)
$\omega_1(a_1^{'})$	897.08	899.30	899.30
$\omega_{2\mathrm{a}}(e^{'})$	1481.97	1493.08	1493.08
$\omega_{2\mathrm{b}}(e^{'})$	1481.64	1493.08	1493.08
$\omega_{3\mathrm{a}}(e^{'})$	468.24	483.89	483.89
$\omega_{3\mathrm{b}}(e^{'})$	467.65	483.88	483.88
$\omega_4(a_2^{''})$	695.80	700.10	700.10

Table S48: Symmetrized, unnormalized natural internal coordinates for boron trifluoride.

1	$r_{1,2} + r_{1,3} + r_{1,4}$
2	$2r_{1,2} - r_{1,3} - r_{1,4}$
3	$r_{1,3} - r_{1,4}$
4	$2\phi_{2,1,3} - \phi_{2,1,4} - \phi_{3,1,4}$
5	$\phi_{2,1,4} - \phi_{3,1,4}$
6	$\gamma_{2,1,3,4} + \gamma_{3,1,4,2} + \gamma_{4,1,2,3}$

S4.13 tetrachloromethane

Table S49: CCSD(T)/cc-pVTZ Optimum Cartesian coordinates (bohr)

1	C	0.00000000	0.00000000	0.00000006
2	Cl	0.00000000	−2.73683182	1.93523230
3	Cl	0.00000000	2.73683182	1.93523230
4	Cl	2.73683173	0.00000000	−1.93523231
5	Cl	−2.73683173	0.00000000	−1.93523231

Table S50: Reference [CCSD(T)/cc-pVTZ] CMA-0A and CMA-2A (ξ = 0.02) harmonic frequencies for Level B = MP2/cc-pVTZ and CCSD(T)/cc-pVDZ.

	Reference CCSD(T)/ cc-pVTZ	Pure MP2/ cc-pVTZ	CMA-0A MP2/ cc-pVTZ	CMA-2A MP2/ cc-pVTZ	Pure CCSD(T)/ cc-pVDZ	CMA-0A CCSD(T)/ cc-pVDZ	CMA-2A CCSD(T)/ cc-pVDZ
$\omega_1(a_1)$	463.23	464.14	463.23	463.23	472.90	463.23	463.23
$\omega_{2a}(e)$	218.44	218.37	218.43	218.43	227.50	218.42	218.42
$\omega_{2b}(e)$	218.41	218.37	218.42	218.42	227.49	218.42	218.42
$\omega_{3a}(t_2)$	802.57	796.10	802.57	802.57	823.73	802.57	802.57
$\omega_{3b}(t_2)$	802.56	796.03	802.55	802.55	823.65	802.55	802.55
$\omega_{3c}(t_2)$	802.53	796.03	802.54	802.54	823.65	802.54	802.54
$\omega_{4a}(t_2)$	316.79	317.04	316.76	316.76	327.40	316.77	316.77
$\omega_{4b}(t_2)$	316.70	317.02	316.73	316.73	327.38	316.74	316.74
$\omega_{4c}(t_2)$	316.69	317.02	316.69	316.69	327.38	316.71	316.71

Table S51: CMA-0A and CMA-2A (ξ = 0.02) harmonic frequencies targeting CCSD(T)/cc-pVTZ for Level B = B3LYP/6-31G(2df,p).

	Pure B3LYP/ 6-31G(2df,p)	CMA-0A B3LYP/ 6-31G(2df,p)	CMA-2A B3LYP/ 6-31G(2df,p)
$\omega_1(a_1)$	464.91	463.23	463.23
$\omega_{2a}(e)$	219.43	218.44	218.44
$\omega_{2b}(e)$	218.27	218.41	218.41
$\omega_{3a}(t_2)$	778.30	802.49	802.57
$\omega_{3b}(t_2)$	777.58	802.49	802.55
$\omega_{3c}(t_2)$	777.58	802.47	802.54
$\omega_{4a}(t_2)$	319.03	316.93	316.76
$\omega_{4b}(t_2)$	317.44	316.90	316.74
$\omega_{4c}(t_2)$	317.44	316.89	316.70

Table S52: Symmetrized, unnormalized natural internal coordinates for tetrachloromethane.

1	$r_{1,2} + r_{1,3} + r_{1,4} + r_{1,5}$
2	$-r_{1,2} - r_{1,3} + r_{1,4} + r_{1,5}$
3	$-r_{1,2} + r_{1,3} - r_{1,4} + r_{1,5}$
4	$r_{1,2} - r_{1,3} - r_{1,4} + r_{1,5}$
5	$2\phi_{2,1,3} + 2\phi_{4,1,5} - \phi_{2,1,4} - \phi_{2,1,5} - \phi_{3,1,4} - \phi_{3,1,5}$
6	$\phi_{2,1,4} - \phi_{2,1,5} - \phi_{3,1,4} + \phi_{3,1,5}$
7	$-\phi_{2,1,3} + \phi_{4,1,5}$
8	$-\phi_{2,1,4} + \phi_{3,1,5}$
9	$\phi_{2,1,5} - \phi_{3,1,4}$

S4.14 tetrafluoromethane

Table S53: CCSD(T)/cc-pVTZ Optimum Cartesian coordinates (bohr)

1	C	−0.00000000	0.00000000	0.00000000
2	F	0.00000000	−2.03544624	1.43927784
3	F	−0.00000000	2.03544624	1.43927784
4	F	2.03544624	0.00000000	−1.43927784
5	F	−2.03544624	−0.00000000	−1.43927784

Table S54: Reference [CCSD(T)/cc-pVTZ] CMA-0A and CMA-2A (ξ = 0.02) harmonic frequencies for Level B = MP2/cc-pVTZ and CCSD(T)/cc-pVDZ.

	Reference CCSD(T)/ cc-pVTZ	Pure MP2/ cc-pVTZ	CMA-0A MP2/ cc-pVTZ	CMA-2A MP2/ cc-pVTZ	Pure CCSD(T)/ cc-pVDZ	CMA-0A CCSD(T)/ cc-pVDZ	CMA-2A CCSD(T)/ cc-pVDZ
$\omega_1(a_1)$	923.21	925.21	923.21	923.21	932.56	923.21	923.21
$\omega_{2\text{a}}(e)$	440.04	440.49	440.04	440.04	445.39	440.04	440.04
$\omega_{2\text{b}}(e)$	440.04	440.48	440.04	440.04	445.39	440.04	440.04
$\omega_{3\text{a}}(t_2)$	1323.00	1312.79	1323.00	1323.00	1368.72	1322.98	1322.98
$\omega_{3\text{b}}(t_2)$	1323.00	1312.61	1323.00	1323.00	1368.55	1322.98	1322.98
$\omega_{3\text{c}}(t_2)$	1323.00	1312.61	1323.00	1323.00	1368.55	1322.98	1322.98
$\omega_{4\text{a}}(t_2)$	638.99	640.37	639.00	639.00	645.95	639.05	639.05
$\omega_{4\text{b}}(t_2)$	638.99	640.34	639.00	639.00	645.92	639.05	639.05
$\omega_{4\text{c}}(t_2)$	638.99	640.34	639.00	639.00	645.92	639.05	639.05

Table S55: CMA-0A and CMA-2A (ξ = 0.02) harmonic frequencies targeting CCSD(T)/cc-pVTZ for Level B = B3LYP/6-31G(2*df*,*p*).

	Pure B3LYP/ 6-31G(2*df*,*p*)	CMA-0A B3LYP/ 6-31G(2*df*,*p*)	CMA-2A B3LYP/ 6-31G(2*df*,*p*)
$\omega_1(a_1)$	929.65	923.21	923.21
$\omega_{2\text{a}}(e)$	434.21	440.04	440.04
$\omega_{2\text{b}}(e)$	433.63	440.04	440.04
$\omega_{3\text{a}}(t_2)$	1320.23	1323.00	1323.00
$\omega_{3\text{b}}(t_2)$	1320.23	1323.00	1323.00
$\omega_{3\text{c}}(t_2)$	1318.71	1323.00	1323.00
$\omega_{4\text{a}}(t_2)$	633.97	639.00	639.00
$\omega_{4\text{b}}(t_2)$	633.46	638.99	638.99
$\omega_{4\text{c}}(t_2)$	633.46	638.99	638.99

Table S56: Symmetrized, unnormalized natural internal coordinates for tetrafluoromethane.

1	$r_{1,2} + r_{1,3} + r_{1,4} + r_{1,5}$
2	$-r_{1,2} - r_{1,3} + r_{1,4} + r_{1,5}$
3	$-r_{1,2} + r_{1,3} - r_{1,4} + r_{1,5}$
4	$r_{1,2} - r_{1,3} - r_{1,4} + r_{1,5}$
5	$2\phi_{2,1,3} + 2\phi_{4,1,5} - \phi_{2,1,4} - \phi_{2,1,5} - \phi_{3,1,4} - \phi_{3,1,5}$
6	$\phi_{2,1,4} - \phi_{2,1,5} - \phi_{3,1,4} + \phi_{3,1,5}$
7	$-\phi_{2,1,3} + \phi_{4,1,5}$
8	$-\phi_{2,1,4} + \phi_{3,1,5}$
9	$\phi_{2,1,5} - \phi_{3,1,4}$

S4.15 dichloromethane

Table S57: CCSD(T)/cc-pVTZ Optimum Cartesian coordinates (bohr)

1	C	−0.00000000	1.52259374	0.00000000
2	Cl	−2.78829097	−0.33831409	0.00000000
3	Cl	2.78829098	−0.33831426	0.00000000
4	H	−0.00000018	2.67397215	−1.69354225
5	H	−0.00000018	2.67397215	1.69354225

Table S58: Reference [CCSD(T)/cc-pVTZ] CMA-0A and CMA-2A (ξ = 0.02) harmonic frequencies for Level B = MP2/cc-pVTZ and CCSD(T)/cc-pVDZ.

	Reference CCSD(T)/ cc-pVTZ	Pure MP2/ cc-pVTZ	CMA-0A MP2/ cc-pVTZ	CMA-2A MP2/ cc-pVTZ	Pure CCSD(T)/ cc-pVDZ	CMA-0A CCSD(T)/ cc-pVDZ	CMA-2A CCSD(T)/ cc-pVDZ
$\omega_1(a_1)$	3127.54	3181.24	3127.54	3127.54	3285.55	3127.50	3127.50
$\omega_2(a_1)$	1474.48	1570.52	1474.48	1474.48	1565.32	1474.54	1474.54
$\omega_3(a_1)$	723.57	753.43	723.57	723.57	751.44	723.63	723.63
$\omega_4(a_1)$	284.60	301.28	284.63	284.63	304.57	284.65	284.65
$\omega_5(a_2)$	1180.95	1264.75	1180.95	1180.95	1262.27	1180.95	1180.95
$\omega_6(b_1)$	3202.53	3255.44	3202.51	3202.51	3375.46	3202.48	3202.48
$\omega_7(b_1)$	906.97	968.21	907.04	907.04	962.13	907.15	907.15
$\omega_8(b_2)$	1293.06	1391.40	1293.01	1293.01	1388.69	1292.98	1292.98
$\omega_9(b_2)$	776.83	812.87	776.92	776.92	808.65	776.97	776.97

Table S59: CMA-0A and CMA-2A (ξ = 0.02) harmonic frequencies targeting CCSD(T)/cc-pVTZ for Level B = B3LYP/6-31G(2*df*,*p*).

	Pure B3LYP/ 6-31G(2*df*,*p*)	CMA-0A B3LYP/ 6-31G(2*df*,*p*)	CMA-2A B3LYP/ 6-31G(2*df*,*p*)
$\omega_1(a_1)$	3146.93	3127.53	3127.53
$\omega_2(a_1)$	1467.53	1474.50	1474.50
$\omega_3(a_1)$	724.41	723.58	723.58
$\omega_4(a_1)$	285.95	284.63	284.63
$\omega_5(a_2)$	1172.10	1180.95	1180.95
$\omega_6(b_1)$	3226.63	3202.52	3202.52
$\omega_7(b_1)$	907.13	907.01	907.01
$\omega_8(b_2)$	1293.52	1292.99	1293.06
$\omega_9(b_2)$	755.30	776.95	776.83

Table S60: Symmetrized, unnormalized natural internal coordinates for dichloromethane.

1	$r_{1,2} + r_{1,3}$
2	$r_{1,2} - r_{1,3}$
3	$r_{1,4} + r_{1,5}$
4	$r_{1,4} - r_{1,5}$
5	$4\phi_{2,1,3} - \phi_{2,1,4} - \phi_{2,1,5} - \phi_{3,1,4} - \phi_{3,1,5}$
6	$\phi_{2,1,4} + \phi_{2,1,5} - \phi_{3,1,4} - \phi_{3,1,5}$
7	$\phi_{2,1,4} - \phi_{2,1,5} + \phi_{3,1,4} - \phi_{3,1,5}$
8	$\phi_{2,1,4} - \phi_{2,1,5} - \phi_{3,1,4} + \phi_{3,1,5}$
9	$-\phi_{2,1,3} - \phi_{2,1,4} - \phi_{2,1,5} - \phi_{3,1,4} - \phi_{3,1,5} + 5\phi_{4,1,5}$

S4.16 difluoromethane

Table S61: CCSD(T)/cc-pVTZ Optimum Cartesian coordinates (bohr)

1	C	0.00000000	1.04690947	−0.00000000
2	F	−2.07913590	−0.44653109	0.00000000
3	F	2.07913592	−0.44653108	0.00000000
4	H	−0.00000010	2.18482447	−1.71602833
5	H	−0.00000010	2.18482447	1.71602833

Table S62: Reference [CCSD(T)/cc-pVTZ] CMA-0A and CMA-2A (ξ = 0.02) harmonic frequencies for Level B = MP2/cc-pVTZ and CCSD(T)/cc-pVDZ.

	Reference CCSD(T)/ cc-pVTZ	Pure MP2/ cc-pVTZ	CMA-0A MP2/ cc-pVTZ	CMA-2A MP2/ cc-pVTZ	Pure CCSD(T)/ cc-pVDZ	CMA-0A CCSD(T)/ cc-pVDZ	CMA-2A CCSD(T)/ cc-pVDZ
$\omega_1(a_1)$	3075.76	3138.20	3075.74	3075.74	3237.24	3075.69	3075.69
$\omega_2(a_1)$	1556.46	1640.94	1556.44	1556.44	1635.56	1556.53	1556.53
$\omega_3(a_1)$	1141.59	1155.76	1141.60	1141.60	1166.89	1141.65	1141.65
$\omega_4(a_1)$	536.99	551.26	537.12	537.12	554.42	537.04	537.04
$\omega_5(a_2)$	1292.33	1364.16	1292.33	1292.33	1366.10	1292.33	1292.33
$\omega_6(b_1)$	3148.01	3207.64	3148.00	3148.00	3318.85	3147.96	3147.96
$\omega_7(b_1)$	1202.57	1257.62	1202.59	1202.59	1250.29	1202.67	1202.67
$\omega_8(b_2)$	1482.31	1554.38	1482.11	1482.11	1565.56	1482.29	1482.29
$\omega_9(b_1)$	1142.17	1141.27	1142.42	1142.42	1169.57	1142.20	1142.20

Table S63: CMA-0A and CMA-2A (ξ = 0.02) harmonic frequencies targeting CCSD(T)/cc-pVTZ for Level B = B3LYP/6-31G(2*df*,*p*).

	Pure B3LYP/ 6-31G(2*df*,*p*)	CMA-0A B3LYP/ 6-31G(2*df*,*p*)	CMA-2A B3LYP/ 6-31G(2*df*,*p*)
$\omega_1(a_1)$	3091.25	3075.70	3075.70
$\omega_2(a_1)$	1546.91	1556.46	1556.46
$\omega_3(a_1)$	1141.12	1141.72	1141.72
$\omega_4(a_1)$	534.30	537.02	537.02
$\omega_5(a_2)$	1269.14	1292.33	1292.33
$\omega_6(b_1)$	3160.81	3148.00	3148.00
$\omega_7(b_1)$	1192.41	1202.57	1202.57
$\omega_8(b_2)$	1479.96	1482.30	1482.30
$\omega_9(b_2)$	1134.95	1142.17	1142.17

Table S64: Symmetrized, unnormalized natural internal coordinates for difluoromethane.

1	$r_{1,2} + r_{1,3}$
2	$r_{1,2} - r_{1,3}$
3	$r_{1,4} + r_{1,5}$
4	$r_{1,4} - r_{1,5}$
5	$4\phi_{2,1,3} - \phi_{2,1,4} - \phi_{2,1,5} - \phi_{3,1,4} - \phi_{3,1,5}$
6	$\phi_{2,1,4} + \phi_{2,1,5} - \phi_{3,1,4} - \phi_{3,1,5}$
7	$\phi_{2,1,4} - \phi_{2,1,5} + \phi_{3,1,4} - \phi_{3,1,5}$
8	$\phi_{2,1,4} - \phi_{2,1,5} - \phi_{3,1,4} + \phi_{3,1,5}$
9	$-\phi_{2,1,3} - \phi_{2,1,4} - \phi_{2,1,5} - \phi_{3,1,4} - \phi_{3,1,5} + 5\phi_{4,1,5}$

S4.17 formic acid

Table S65: CCSD(T)/cc-pVTZ Optimum Cartesian coordinates (bohr)

1	H	0.05468955	2.84555164	−0.00000000
2	C	0.18775950	0.78180753	−0.00000000
3	O	2.12426566	−0.40608921	−0.00000000
4	O	−2.14789723	−0.23081035	0.00000000
5	H	−1.91525954	−2.04634183	0.00000000

Table S66: Reference [CCSD(T)/cc-pVTZ] CMA-0A and CMA-2A (ξ = 0.02) harmonic frequencies for Level B = MP2/cc-pVTZ and CCSD(T)/cc-pVDZ.

	Reference CCSD(T)/ cc-pVTZ	Pure MP2/ cc-pVTZ	CMA-0A MP2/ cc-pVTZ	CMA-2A MP2/ cc-pVTZ	Pure CCSD(T)/ cc-pVDZ	CMA-0A CCSD(T)/ cc-pVDZ	CMA-2A CCSD(T)/ cc-pVDZ
$\omega_1(a^{'})$	3764.00	3775.33	3764.00	3764.00	3837.11	3763.97	3763.97
$\omega_2(a^{'})$	3089.21	3106.69	3089.21	3089.21	3232.78	3089.20	3089.20
$\omega_3(a^{'})$	1824.55	1822.20	1824.47	1824.53	1886.00	1824.18	1824.50
$\omega_4(a^{'})$	1415.80	1415.99	1415.64	1415.57	1441.54	1415.51	1415.50
$\omega_5(a^{'})$	1326.19	1315.23	1326.02	1326.25	1344.92	1326.73	1326.41
$\omega_6(a^{'})$	1137.12	1132.56	1137.64	1137.37	1167.34	1137.51	1137.39
$\omega_7(a^{'})$	629.41	629.42	629.42	629.42	638.26	629.44	629.44
$\omega_8(a^{''})$	1061.21	1067.48	1061.18	1061.21	1076.40	1061.14	1061.21
$\omega_9(a^{''})$	675.64	684.33	675.68	675.64	698.30	675.74	675.64

Table S67: CMA-0A and CMA-2A (ξ = 0.02) harmonic frequencies targeting CCSD(T)/cc-pVTZ for Level B = B3LYP/6-31G(2*df*,*p*).

	Pure B3LYP/ 6-31G(2*df*,*p*)	CMA-0A B3LYP/ 6-31G(2*df*,*p*)	CMA-2A B3LYP/ 6-31G(2*df*,*p*)
$\omega_1(a^{'})$	3760.05	3763.99	3763.99
$\omega_2(a^{'})$	3107.60	3089.18	3089.18
$\omega_3(a^{'})$	1831.55	1824.42	1824.49
$\omega_4(a^{'})$	1407.34	1415.88	1415.80
$\omega_5(a^{'})$	1318.58	1326.25	1326.25
$\omega_6(a^{'})$	1130.52	1137.27	1137.27
$\omega_7(a^{'})$	624.72	629.44	629.44
$\omega_8(a^{''})$	1059.65	1061.20	1061.21
$\omega_9(a^{''})$	688.89	675.64	675.64

Table S68: Symmetrized, unnormalized natural internal coordinates for formic acid.

1	$r_{2,3}$
2	$r_{2,4}$
3	$r_{2,1}$
4	$r_{4,5}$
5	$\phi_{2,4,5}$
6	$2\phi_{3,2,4} - \phi_{3,2,1} - \phi_{4,2,1}$
7	$\phi_{3,2,1} - \phi_{4,2,1}$
8	$\tau_{1,2,4,5} + \tau_{3,2,4,5}$
9	$\gamma_{1,2,3,4}$

S4.18 formaldehyde

Table S69: CCSD(T)/cc-pVTZ Optimum Cartesian coordinates (bohr)

1	C	0.00000000	0.00000000	1.14423616
2	H	0.00000000	−1.76987484	2.24620597
3	H	0.00000000	1.76987484	2.24620597
4	O	−0.00000000	0.00000000	−1.14151276

Table S70: Reference [CCSD(T)/cc-pVTZ] CMA-0A and CMA-2A (ξ = 0.02) harmonic frequencies for Level B = MP2/cc-pVTZ and CCSD(T)/cc-pVDZ.

	Reference CCSD(T)/ cc-pVTZ	Pure MP2/ cc-pVTZ	CMA-0A MP2/ cc-pVTZ	CMA-2A MP2/ cc-pVTZ	Pure CCSD(T)/ cc-pVDZ	CMA-0A CCSD(T)/ cc-pVDZ	CMA-2A CCSD(T)/ cc-pVDZ
$\omega_1(a_1)$	2929.23	2946.14	2929.22	2929.22	3068.95	2929.20	2929.20
$\omega_2(a_1)$	1780.76	1775.33	1780.75	1780.75	1827.20	1780.74	1780.74
$\omega_3(a_1)$	1543.21	1550.10	1543.24	1543.24	1566.35	1543.28	1543.28
$\omega_4(b_1)$	1192.19	1206.14	1192.19	1192.19	1206.38	1192.19	1192.19
$\omega_5(b_2)$	2995.85	3017.92	2995.85	2995.85	3143.13	2995.85	2995.85
$\omega_6(b_2)$	1274.88	1278.44	1274.88	1274.88	1296.37	1274.88	1274.88

Table S71: CMA-0A and CMA-2A (ξ = 0.02) harmonic frequencies targeting CCSD(T)/cc-pVTZ for Level B = B3LYP/6-31G(2*df*,*p*).

	Pure B3LYP/ 6-31G(2*df*,*p*)	CMA-0A B3LYP/ 6-31G(2*df*,*p*)	CMA-2A B3LYP/ 6-31G(2*df*,*p*)
$\omega_1(a_1)$	2949.27	2929.19	2929.19
$\omega_2(a_1)$	1797.21	1780.50	1780.50
$\omega_3(a_1)$	1536.30	1543.59	1543.59
$\omega_4(b_1)$	1208.00	1192.19	1192.19
$\omega_5(b_2)$	3013.06	2995.84	2995.84
$\omega_6(b_2)$	1266.40	1274.89	1274.89

Table S72: Symmetrized, unnormalized natural internal coordinates for formaldehyde.

1	$r_{1,2} + r_{1,3}$
2	$r_{1,2} - r_{1,3}$
3	$r_{1,4}$
4	$2\phi_{2,1,3} - \phi_{2,1,4} - \phi_{3,1,4}$
5	$\phi_{2,1,4} - \phi_{3,1,4}$
6	$\gamma_{4,1,2,3}$

S4.19 singlet methylene

Table S73: CCSD(T)/cc-pVTZ Optimum Cartesian coordinates (bohr)

1	C	0.00000000	−0.00000000	0.19071799
2	H	0.00000000	−1.62640634	−1.13542319
3	H	0.00000000	1.62640634	−1.13542319

Table S74: Reference [CCSD(T)/cc-pVTZ] CMA-0A and CMA-2A (ξ = 0.02) harmonic frequencies for Level B = MP2/cc-pVTZ and CCSD(T)/cc-pVDZ.

	Reference CCSD(T)/ cc-pVTZ	Pure MP2/ cc-pVTZ	CMA-0A MP2/ cc-pVTZ	CMA-2A MP2/ cc-pVTZ	Pure CCSD(T)/ cc-pVDZ	CMA-0A CCSD(T)/ cc-pVDZ	CMA-2A CCSD(T)/ cc-pVDZ
$\omega_1(a_1)$	2912.05	2939.49	2912.05	2912.05	3057.57	2911.92	2911.92
$\omega_2(a_1)$	1406.59	1421.07	1406.59	1406.59	1427.87	1406.85	1406.85
$\omega_3(b_2)$	2983.12	3014.35	2983.12	2983.12	3144.66	2983.12	2983.12

Table S75: CMA-0A and CMA-2A (ξ = 0.02) harmonic frequencies targeting CCSD(T)/cc-pVTZ for Level B = B3LYP/6-31G(2*df*,*p*).

	Pure B3LYP/ 6-31G(2*df*,*p*)	CMA-0A B3LYP/ 6-31G(2*df*,*p*)	CMA-2A B3LYP/ 6-31G(2*df*,*p*)
$\omega_1(a_1)$	2939.19	2912.05	2912.05
$\omega_2(a_1)$	1428.38	1406.59	1406.59
$\omega_3(b_2)$	3012.46	2983.12	2983.12

Table S76: Symmetrized, unnormalized natural internal coordinates for singlet methylene.

1	$r_{1,2} + r_{1,3}$
2	$r_{1,2} - r_{1,3}$
3	$\phi_{2,1,3}$

S4.20 chloromethane

Table S77: CCSD(T)/cc-pVTZ Optimum Cartesian coordinates (bohr)

1	C	2.32154417	−0.00000390	0.00000000
2	Cl	−1.05377924	0.00000143	0.00000000
3	H	2.97370152	1.94601045	0.00000000
4	H	2.97370577	−0.97300677	1.68529621
5	H	2.97370577	−0.97300677	−1.68529621

Table S78: Reference [CCSD(T)/cc-pVTZ] CMA-0A and CMA-2A (ξ = 0.02) harmonic frequencies for Level B = MP2/cc-pVTZ and CCSD(T)/cc-pVDZ.

	Reference CCSD(T)/ cc-pVTZ	Pure MP2/ cc-pVTZ	CMA-0A MP2/ cc-pVTZ	CMA-2A MP2/ cc-pVTZ	Pure CCSD(T)/ cc-pVDZ	CMA-0A CCSD(T)/ cc-pVDZ	CMA-2A CCSD(T)/ cc-pVDZ
$\omega_1(a_1)$	3078.66	3092.05	3078.66	3078.66	3216.56	3078.65	3078.65
$\omega_2(a_1)$	1385.57	1386.90	1385.57	1385.57	1407.78	1385.57	1385.57
$\omega_3(a_1)$	746.24	755.63	746.25	746.25	756.37	746.29	746.29
$\omega_{4\text{a}}(e)$	3179.27	3202.47	3179.26	3179.26	3336.72	3179.23	3179.23
$\omega_{4\text{b}}(e)$	3179.26	3202.36	3179.26	3179.26	3336.60	3179.23	3179.23
$\omega_{5\text{a}}(e)$	1493.13	1498.75	1493.12	1493.12	1505.91	1493.18	1493.18
$\omega_{5\text{b}}(e)$	1493.13	1498.74	1493.12	1493.12	1505.90	1493.18	1493.18
$\omega_{6\text{a}}(e)$	1031.75	1036.55	1031.76	1031.76	1045.57	1031.78	1031.78
$\omega_{6\text{b}}(e)$	1031.74	1036.54	1031.76	1031.76	1045.57	1031.78	1031.78

Table S79: CMA-0A and CMA-2A (ξ = 0.02) harmonic frequencies targeting CCSD(T)/cc-pVTZ for Level B = B3LYP/6-31G(2*df*,*p*).

	Pure B3LYP/ 6-31G(2*df*,*p*)	CMA-0A B3LYP/ 6-31G(2*df*,*p*)	CMA-2A B3LYP/ 6-31G(2*df*,*p*)
$\omega_1(a_1)$	3101.77	3078.65	3078.65
$\omega_2(a_1)$	1386.98	1385.56	1385.56
$\omega_3(a_1)$	743.40	746.33	746.33
$\omega_{4\text{a}}(e)$	3202.48	3179.26	3179.26
$\omega_{4\text{b}}(e)$	3202.31	3179.25	3179.25
$\omega_{5\text{a}}(e)$	1488.50	1493.11	1493.11
$\omega_{5\text{b}}(e)$	1488.42	1493.11	1493.11
$\omega_{6\text{a}}(e)$	1032.51	1031.81	1031.81
$\omega_{6\text{b}}(e)$	1032.48	1031.80	1031.80

Table S80: Symmetrized, unnormalized natural internal coordinates for chloromethane.

1	$r_{1,2}$
2	$r_{1,3} + r_{1,4} + r_{1,5}$
3	$2r_{1,3} - r_{1,4} - r_{1,5}$
4	$r_{1,4} - r_{1,5}$
5	$2\phi_{3,1,2} - \phi_{4,1,2} - \phi_{5,1,2}$
6	$\phi_{4,1,2} - \phi_{5,1,2}$
7	$\phi_{3,1,2} + \phi_{4,1,2} + \phi_{5,1,2} - \phi_{3,1,4} - \phi_{4,1,5} - \phi_{5,1,3}$
8	$2\phi_{3,1,4} - \phi_{4,1,5} - \phi_{5,1,3}$
9	$\phi_{4,1,5} - \phi_{5,1,3}$

S4.21 thiomethanol

Table S81: CCSD(T)/cc-pVTZ Optimum Cartesian coordinates (bohr)

1	C	−2.32717838	−0.02121580	0.00000000
2	S	1.11216012	0.08308952	0.00000000
3	H	−2.96268843	1.93642244	0.00000000
4	H	−3.04525758	−0.95063598	1.68762632
5	H	−3.04525758	−0.95063598	−1.68762632
6	H	1.48053809	−2.41845552	0.00000000

Table S82: Reference [CCSD(T)/cc-pVTZ] CMA-0A and CMA-2A (ξ = 0.02) harmonic frequencies for Level B = MP2/cc-pVTZ and CCSD(T)/cc-pVDZ.

	Reference CCSD(T)/ cc-pVTZ	Pure MP2/ cc-pVTZ	CMA-0A MP2/ cc-pVTZ	CMA-2A MP2/ cc-pVTZ	Pure CCSD(T)/ cc-pVDZ	CMA-0A CCSD(T)/ cc-pVDZ	CMA-2A CCSD(T)/ cc-pVDZ
$\omega_1(a^{'})$	3149.99	3173.76	3149.99	3149.99	3305.38	3149.95	3149.95
$\omega_2(a^{'})$	3059.94	3073.17	3059.94	3059.94	3196.60	3059.93	3059.93
$\omega_3(a^{'})$	2712.63	2755.14	2712.63	2712.63	2800.49	2712.63	2712.63
$\omega_4(a^{'})$	1496.98	1501.04	1496.96	1496.96	1510.51	1496.99	1496.99
$\omega_5(a^{'})$	1362.12	1360.13	1362.12	1362.12	1382.53	1362.12	1362.12
$\omega_6(a^{'})$	1099.79	1097.61	1099.75	1099.75	1117.42	1099.83	1099.83
$\omega_7(a^{'})$	799.87	804.38	799.83	799.93	810.49	799.93	799.93
$\omega_8(a^{'})$	720.90	727.67	721.07	720.95	732.70	721.01	721.01
$\omega_9(a^{''})$	3151.32	3175.11	3151.31	3151.31	3306.92	3151.28	3151.28
$\omega_{10}(a^{''})$	1482.93	1487.09	1482.91	1482.91	1496.52	1482.98	1482.98
$\omega_{11}(a^{''})$	976.26	980.38	976.29	976.29	991.86	976.31	976.31
$\omega_{12}(a^{''})$	237.65	240.26	237.66	237.66	257.94	237.66	237.66

Table S83: CMA-0A and CMA-2A (ξ = 0.02) harmonic frequencies targeting CCSD(T)/cc-pVTZ for Level B = B3LYP/6-31G(2*df*,*p*).

	Pure B3LYP/ 6-31G(2*df*,*p*)	CMA-0A B3LYP/ 6-31G(2*df*,*p*)	CMA-2A B3LYP/ 6-31G(2*df*,*p*)
$\omega_1(a^{'})$	3170.51	3149.97	3149.97
$\omega_2(a^{'})$	3081.64	3059.93	3059.93
$\omega_3(a^{'})$	2736.03	2712.62	2712.62
$\omega_4(a^{'})$	1494.87	1496.94	1496.94
$\omega_5(a^{'})$	1366.09	1362.12	1362.17
$\omega_6(a^{'})$	1105.64	1099.86	1099.86
$\omega_7(a^{'})$	805.48	799.90	799.91
$\omega_8(a^{'})$	714.49	721.07	720.95
$\omega_9(a^{''})$	3171.98	3151.30	3151.30
$\omega_{10}(a^{''})$	1482.11	1482.91	1482.91
$\omega_{11}(a^{''})$	977.83	976.33	976.33
$\omega_{12}(a^{''})$	239.49	237.67	237.67

Table S84: Symmetrized, unnormalized natural internal coordinates for thiomethanol.

1	$r_{1,2}$
2	$r_{2,6}$
3	$r_{1,3} + r_{1,4} + r_{1,5}$
4	$r_{1,4} - r_{1,5}$
5	$2r_{1,3} - r_{1,4} - r_{1,5}$
6	$\phi_{1,2,6}$
7	$\phi_{3,1,2} + \phi_{4,1,2} + \phi_{5,1,2} - \phi_{4,1,5} - \phi_{3,1,4} - \phi_{3,1,5}$
8	$2\phi_{3,1,2} - \phi_{4,1,2} - \phi_{5,1,2}$
9	$\phi_{4,1,2} - \phi_{5,1,2}$
10	$2\phi_{4,1,5} - \phi_{3,1,4} - \phi_{3,1,5}$
11	$\phi_{3,1,4} - \phi_{3,1,5}$
12	$\tau_{3,1,2,6} + \tau_{4,1,2,6} + \tau_{5,1,2,6}$

S4.22 trichloromethane

Table S85: CCSD(T)/cc-pVTZ Optimum Cartesian coordinates (bohr)

1	C	0.00000070	0.89489730	0.00000000
2	H	0.00000088	2.93924961	0.00000000
3	Cl	−3.18361737	−0.13060258	0.00000000
4	Cl	1.59180855	−0.13060186	−2.75709409
5	Cl	1.59180855	−0.13060186	2.75709409

Table S86: Reference [CCSD(T)/cc-pVTZ] CMA-0A and CMA-2A (ξ = 0.02) harmonic frequencies for Level B = MP2/cc-pVTZ and CCSD(T)/cc-pVDZ.

	Reference CCSD(T)/ cc-pVTZ	Pure MP2/ cc-pVTZ	CMA-0A MP2/ cc-pVTZ	CMA-2A MP2/ cc-pVTZ	Pure CCSD(T)/ cc-pVDZ	CMA-0A CCSD(T)/ cc-pVDZ	CMA-2A CCSD(T)/ cc-pVDZ
$\omega_1(a_1)$	3181.88	3198.61	3181.88	3181.88	3327.89	3181.88	3181.88
$\omega_2(a_1)$	678.57	682.46	678.58	678.58	689.02	678.60	678.60
$\omega_3(a_1)$	368.48	369.43	368.48	368.48	377.26	368.48	368.48
$\omega_{4\mathrm{a}}(e)$	1244.75	1245.10	1244.72	1244.75	1271.34	1244.72	1244.72
$\omega_{4\mathrm{b}}(e)$	1244.75	1245.09	1244.72	1244.75	1271.32	1244.72	1244.72
$\omega_{5\mathrm{a}}(e)$	789.90	789.49	789.94	789.89	807.75	789.94	789.94
$\omega_{5\mathrm{b}}(e)$	789.88	789.47	789.94	789.89	807.73	789.94	789.94
$\omega_{6\mathrm{a}}(e)$	261.72	261.91	261.69	261.69	270.52	261.70	261.70
$\omega_{6\mathrm{b}}(e)$	261.66	261.91	261.69	261.69	270.51	261.70	261.70

Table S87: CMA-0A and CMA-2A (ξ = 0.02) harmonic frequencies targeting CCSD(T)/cc-pVTZ for Level B = B3LYP/6-31G(2*df*,*p*).

	Pure B3LYP/ 6-31G(2*df*,*p*)	CMA-0A B3LYP/ 6-31G(2*df*,*p*)	CMA-2A B3LYP/ 6-31G(2*df*,*p*)
$\omega_1(a_1)$	3203.72	3181.88	3181.88
$\omega_2(a_1)$	680.55	678.57	678.57
$\omega_3(a_1)$	370.51	368.49	368.49
$\omega_{4\mathrm{a}}(e)$	1239.37	1244.72	1244.75
$\omega_{4\mathrm{b}}(e)$	1237.99	1244.70	1244.73
$\omega_{5\mathrm{a}}(e)$	777.17	789.93	789.88
$\omega_{5\mathrm{b}}(e)$	765.32	789.90	789.87
$\omega_{6\mathrm{a}}(e)$	263.11	261.81	261.80
$\omega_{6\mathrm{b}}(e)$	261.74	261.80	261.78

Table S88: Symmetrized, unnormalized natural internal coordinates for trichloromethane.

1	$r_{1,2}$
2	$r_{1,3} + r_{1,4} + r_{1,5}$
3	$2r_{1,3} - r_{1,4} - r_{1,5}$
4	$r_{1,4} - r_{1,5}$
5	$2\phi_{3,1,2} - \phi_{4,1,2} - \phi_{5,1,2}$
6	$\phi_{4,1,2} - \phi_{5,1,2}$
7	$\phi_{3,1,2} + \phi_{4,1,2} + \phi_{5,1,2} - \phi_{3,1,4} - \phi_{3,1,5} - \phi_{4,1,5}$
8	$-\phi_{3,1,4} - \phi_{3,1,5} + 2\phi_{4,1,5}$
9	$\phi_{3,1,4} - \phi_{3,1,5}$

S4.23 trifluoromethane

Table S89: CCSD(T)/cc-pVTZ Optimum Cartesian coordinates (bohr)

1	C	0.68502199	−0.00000000	0.00000000
2	H	2.73949866	−0.00000000	0.00000000
3	F	−0.19266881	1.18093464	−2.04543880
4	F	−0.19266881	−2.36186929	−0.00000000
5	F	−0.19266881	1.18093464	2.04543880

Table S90: Reference [CCSD(T)/cc-pVTZ] CMA-0A and CMA-2A (ξ = 0.02) harmonic frequencies for Level B = MP2/cc-pVTZ and CCSD(T)/cc-pVDZ.

	Reference CCSD(T)/ cc-pVTZ	Pure MP2/ cc-pVTZ	CMA-0A MP2/ cc-pVTZ	CMA-2A MP2/ cc-pVTZ	Pure CCSD(T)/ cc-pVDZ	CMA-0A CCSD(T)/ cc-pVDZ	CMA-2A CCSD(T)/ cc-pVDZ
$\omega_1(a_1)$	3160.87	3181.06	3160.86	3160.86	3298.35	3160.86	3160.86
$\omega_2(a_1)$	1161.90	1164.54	1161.91	1161.91	1173.52	1161.89	1161.89
$\omega_3(a_1)$	710.23	711.57	710.23	710.23	715.61	710.28	710.28
$\omega_{4\text{a}}(e)$	1424.32	1427.23	1424.17	1424.17	1455.48	1423.08	1424.32
$\omega_{4\text{b}}(e)$	1424.32	1427.20	1424.17	1424.17	1455.46	1423.08	1424.32
$\omega_{5\text{a}}(e)$	1203.17	1197.13	1203.35	1203.35	1238.14	1204.62	1203.16
$\omega_{5\text{b}}(e)$	1203.17	1197.08	1203.35	1203.35	1238.09	1204.62	1203.16
$\omega_{6\text{a}}(e)$	514.80	515.48	514.81	514.81	521.10	514.82	514.82
$\omega_{6\text{b}}(e)$	514.80	515.46	514.81	514.81	521.08	514.82	514.82

Table S91: CMA-0A and CMA-2A (ξ = 0.02) harmonic frequencies targeting CCSD(T)/cc-pVTZ for Level B = B3LYP/6-31G(2*df*,*p*).

	Pure B3LYP/ 6-31G(2*df*,*p*)	CMA-0A B3LYP/ 6-31G(2*df*,*p*)	CMA-2A B3LYP/ 6-31G(2*df*,*p*)
$\omega_1(a_1)$	3176.61	3160.83	3160.83
$\omega_2(a_1)$	1162.93	1161.96	1162.00
$\omega_3(a_1)$	705.86	710.30	710.23
$\omega_{4\text{a}}(e)$	1410.21	1424.26	1424.26
$\omega_{4\text{b}}(e)$	1408.93	1424.25	1424.25
$\omega_{5\text{a}}(e)$	1198.89	1203.23	1203.23
$\omega_{5\text{b}}(e)$	1198.07	1203.22	1203.22
$\omega_{6\text{a}}(e)$	509.31	514.84	514.84
$\omega_{6\text{b}}(e)$	508.99	514.84	514.84

Table S92: Symmetrized, unnormalized natural internal coordinates for trifluoromethane.

1	$r_{1,2}$
2	$r_{1,3} + r_{1,4} + r_{1,5}$
3	$2r_{1,3} - r_{1,4} - r_{1,5}$
4	$r_{1,4} - r_{1,5}$
5	$2\phi_{3,1,2} - \phi_{4,1,2} - \phi_{5,1,2}$
6	$\phi_{4,1,2} - \phi_{5,1,2}$
7	$\phi_{3,1,2} + \phi_{4,1,2} + \phi_{5,1,2} - \phi_{3,1,4} - \phi_{3,1,5} - \phi_{4,1,5}$
8	$-\phi_{3,1,4} - \phi_{3,1,5} + 2\phi_{4,1,5}$
9	$\phi_{3,1,4} - \phi_{3,1,5}$

S4.24 methyl nitrite

Table S93: CCSD(T)/cc-pVTZ Optimum Cartesian coordinates (bohr)

1	C	0.34164099	3.25928704	0.00000000
2	O	−0.95847922	0.88669596	0.00000000
3	N	0.84560637	−1.10908148	0.00000000
4	O	−0.16294508	−3.08906870	0.00000000
5	H	2.37626442	2.93275940	0.00000000
6	H	−0.19772995	4.31132740	−1.68539213
7	H	−0.19772995	4.31132740	1.68539213

Table S94: Reference [CCSD(T)/cc-pVTZ] CMA-0A and CMA-2A (ξ = 0.02) harmonic frequencies for Level B = MP2/cc-pVTZ and CCSD(T)/cc-pVDZ.

	Reference CCSD(T)/ cc-pVTZ	Pure MP2/ cc-pVTZ	CMA-0A MP2/ cc-pVTZ	CMA-2A MP2/ cc-pVTZ	Pure CCSD(T)/ cc-pVDZ	CMA-0A CCSD(T)/ cc-pVDZ	CMA-2A CCSD(T)/ cc-pVDZ
$\omega_1(a^{'})$	3138.47	3161.13	3138.46	3138.46	3290.60	3138.43	3138.43
$\omega_2(a^{'})$	3048.99	3060.44	3048.99	3048.99	3183.03	3048.98	3048.98
$\omega_3(a^{'})$	1710.30	1670.95	1709.54	1709.80	1778.03	1710.10	1710.23
$\omega_4(a^{'})$	1518.60	1524.66	1518.59	1518.59	1528.88	1518.59	1518.59
$\omega_5(a^{'})$	1462.96	1462.45	1462.90	1462.91	1476.91	1463.00	1463.00
$\omega_6(a^{'})$	1209.15	1204.42	1209.10	1209.15	1215.38	1209.19	1209.21
$\omega_7(a^{'})$	1086.78	1082.36	1086.81	1086.82	1102.98	1086.81	1086.82
$\omega_8(a^{'})$	847.80	827.78	846.95	848.94	864.73	848.14	847.86
$\omega_9(a^{'})$	595.03	575.14	598.58	594.91	598.64	595.16	595.10
$\omega_{10}(a^{'})$	380.32	375.76	380.49	380.37	383.73	380.39	380.39
$\omega_{11}(a^{''})$	3137.29	3160.78	3137.28	3137.28	3289.54	3137.25	3137.25
$\omega_{12}(a^{''})$	1494.32	1499.64	1494.32	1494.32	1506.22	1494.34	1494.35
$\omega_{13}(a^{''})$	1176.76	1179.53	1176.78	1176.78	1186.35	1176.81	1176.81
$\omega_{14}(a^{''})$	220.91	229.51	220.87	220.91	223.22	220.85	220.92
$\omega_{15}(a^{''})$	79.92	99.69	80.11	79.94	87.01	80.35	79.94

Table S95: CMA-0A and CMA-2A (ξ = 0.02) harmonic frequencies targeting CCSD(T)/cc-pVTZ for Level B = B3LYP/6-31G(2*df*,*p*).

	Pure B3LYP/ 6-31G(2*df*,*p*)	CMA-0A B3LYP/ 6-31G(2*df*,*p*)	CMA-2A B3LYP/ 6-31G(2*df*,*p*)
$\omega_1(a^{'})$	3157.94	3138.46	3138.46
$\omega_2(a^{'})$	3070.64	3048.96	3048.96
$\omega_3(a^{'})$	1748.62	1710.29	1710.29
$\omega_4(a^{'})$	1511.85	1518.57	1518.57
$\omega_5(a^{'})$	1454.53	1462.89	1462.91
$\omega_6(a^{'})$	1201.26	1209.10	1209.10
$\omega_7(a^{'})$	1069.13	1086.20	1087.00
$\omega_8(a^{'})$	847.81	848.81	847.84
$\omega_9(a^{'})$	597.91	595.18	595.07
$\omega_{10}(a^{'})$	379.26	380.41	380.38
$\omega_{11}(a^{''})$	3157.31	3137.28	3137.28
$\omega_{12}(a^{''})$	1491.94	1494.22	1494.23
$\omega_{13}(a^{''})$	1166.79	1176.86	1176.88
$\omega_{14}(a^{''})$	230.65	220.46	220.91
$\omega_{15}(a^{''})$	121.24	81.57	79.93

Table S96: Symmetrized, unnormalized natural internal coordinates for methyl nitrite.

1	$r_{1,2}$
2	$r_{2,3}$
3	$r_{3,4}$
4	$r_{1,5} + r_{1,6} + r_{1,7}$
5	$2r_{1,5} - r_{1,6} - r_{1,7}$
6	$r_{1,6} - r_{1,7}$
7	$\phi_{2,3,4}$
8	$\phi_{1,2,3}$
9	$\phi_{2,1,5} + \phi_{2,1,6} + \phi_{2,1,7} - \phi_{6,1,7} - \phi_{5,1,7} - \phi_{5,1,6}$
10	$2\phi_{2,1,5} - \phi_{2,1,6} - \phi_{2,1,7}$
11	$\phi_{2,1,6} - \phi_{2,1,7}$
12	$2\phi_{6,1,7} - \phi_{5,1,7} - \phi_{5,1,6}$
13	$\phi_{5,1,7} - \phi_{5,1,6}$
14	$\tau_{1,2,3,4}$
15	$\tau_{3,2,1,5} + \tau_{3,2,1,6} + \tau_{3,2,1,7}$

S4.25 nitromethane

Table S97: CCSD(T)/cc-pVTZ Optimum Cartesian coordinates (bohr)

1	C	2.64676257	−0.00234129	0.00000000
2	N	−0.17451343	−0.01675275	0.00000000
3	O	−1.22490380	0.00548208	2.06093220
4	O	−1.22490380	0.00548208	−2.06093220
5	H	3.28001151	−0.94121976	1.70856088
6	H	3.28001151	−0.94121976	−1.70856088
7	H	3.23040361	1.96907619	0.00000000

Table S98: Reference [CCSD(T)/cc-pVTZ] CMA-0A and CMA-2A (ξ = 0.02) harmonic frequencies for Level B = MP2/cc-pVTZ and CCSD(T)/cc-pVDZ.

	Reference CCSD(T)/ cc-pVTZ	Pure MP2/ cc-pVTZ	CMA-0A MP2/ cc-pVTZ	CMA-2A MP2/ cc-pVTZ	Pure CCSD(T)/ cc-pVDZ	CMA-0A CCSD(T)/ cc-pVDZ	CMA-2A CCSD(T)/ cc-pVDZ
$\omega_1(a^{'})$	3186.71	3207.17	3186.69	3186.69	3338.74	3186.62	3186.62
$\omega_2(a^{'})$	3088.78	3099.93	3088.81	3088.81	3222.07	3088.83	3088.83
$\omega_3(a^{'})$	1487.78	1493.03	1487.76	1487.76	1500.32	1487.74	1487.74
$\omega_4(a^{'})$	1425.54	1427.95	1425.42	1425.46	1456.26	1424.60	1424.62
$\omega_5(a^{'})$	1409.53	1409.25	1409.61	1409.61	1426.97	1410.48	1410.48
$\omega_6(a^{'})$	1142.44	1141.72	1142.51	1142.47	1146.44	1142.49	1142.51
$\omega_7(a^{'})$	935.42	940.68	935.43	935.41	955.35	935.40	935.37
$\omega_8(a^{'})$	669.99	674.13	670.01	670.02	683.70	670.13	670.20
$\omega_9(a^{'})$	608.72	609.44	608.74	608.73	609.20	608.84	608.73
$\omega_{10}(a^{''})$	3215.03	3235.07	3215.03	3215.03	3367.17	3214.99	3214.99
$\omega_{11}(a^{''})$	1642.57	1787.82	1639.69	1642.61	1704.87	1642.16	1642.57
$\omega_{12}(a^{''})$	1475.65	1482.97	1476.77	1475.52	1487.51	1475.99	1475.71
$\omega_{13}(a^{''})$	1110.73	1117.67	1113.44	1110.84	1121.32	1110.98	1110.77
$\omega_{14}(a^{''})$	476.92	478.52	477.01	476.93	485.32	476.87	476.93
$\omega_{15}(a^{''})$	22.46	25.03	23.17	22.46	-22.67	23.80	22.46

Table S99: CMA-0A and CMA-2A (ξ = 0.02) harmonic frequencies targeting CCSD(T)/cc-pVTZ for Level B = B3LYP/6-31G(2*df*,*p*).

	Pure B3LYP/ 6-31G(2*df*,*p*)	CMA-0A B3LYP/ 6-31G(2*df*,*p*)	CMA-2A B3LYP/ 6-31G(2*df*,*p*)
$\omega_1(a^{'})$	3205.45	3186.71	3186.71
$\omega_2(a^{'})$	3108.21	3088.74	3088.74
$\omega_3(a^{'})$	1479.86	1487.33	1487.33
$\omega_4(a^{'})$	1429.47	1425.30	1425.70
$\omega_5(a^{'})$	1404.41	1409.44	1409.73
$\omega_6(a^{'})$	1138.09	1142.39	1142.39
$\omega_7(a^{'})$	934.31	936.42	935.47
$\omega_8(a^{'})$	668.71	670.15	670.01
$\omega_9(a^{'})$	613.87	609.26	609.26
$\omega_{10}(a^{''})$	3234.60	3215.03	3215.03
$\omega_{11}(a^{''})$	1647.59	1642.55	1642.57
$\omega_{12}(a^{''})$	1466.37	1475.64	1475.65
$\omega_{13}(a^{''})$	1113.99	1110.74	1110.74
$\omega_{14}(a^{''})$	480.32	476.93	476.93
$\omega_{15}(a^{''})$	-35.41	23.82	22.46

Table S100: Symmetrized, unnormalized natural internal coordinates for nitromethane.

1	$r_{1,2}$
2	$r_{2,3} + r_{2,4}$
3	$r_{2,3} - r_{2,4}$
4	$r_{1,5} + r_{1,6} + r_{1,7}$
5	$-r_{1,5} - r_{1,6} + 2r_{1,7}$
6	$r_{1,5} - r_{1,6}$
7	$2\phi_{3,2,4} - \phi_{1,2,3} - \phi_{1,2,4}$
8	$\phi_{1,2,3} - \phi_{1,2,4}$
9	$\phi_{2,1,7} + \phi_{2,1,5} + \phi_{2,1,6} - \phi_{5,1,6} - \phi_{5,1,7} - \phi_{6,1,7}$
10	$2\phi_{2,1,7} - \phi_{2,1,5} - \phi_{2,1,6}$
11	$\phi_{2,1,5} - \phi_{2,1,6}$
12	$2\phi_{5,1,6} - \phi_{5,1,7} - \phi_{6,1,7}$
13	$\phi_{5,1,7} - \phi_{6,1,7}$
14	$\tau_{3,2,1,5} + \tau_{3,2,1,6} + \tau_{3,2,1,7} + \tau_{4,2,1,5} + \tau_{4,2,1,6} + \tau_{4,2,1,7}$
15	$\gamma_{1,2,3,4}$

S4.26 methylamine

Table S101: CCSD(T)/cc-pVTZ Optimum Cartesian coordinates (bohr)

1	C	−1.40168824	−0.00000000	0.02605239
2	N	1.36882488	−0.00000000	−0.14039430
3	H	2.05503952	1.52392893	0.79571578
4	H	2.05503953	−1.52392892	0.79571579
5	H	−2.13608556	1.65964124	−0.95180826
6	H	−2.13608556	−1.65964127	−0.95180822
7	H	−2.16717853	0.00000003	1.95267120

Table S102: Reference [CCSD(T)/cc-pVTZ] CMA-0A and CMA-2A ($\xi = 0.02$) harmonic frequencies for Level B = MP2/cc-pVTZ and CCSD(T)/cc-pVDZ.

	Reference CCSD(T)/ cc-pVTZ	Pure MP2/ cc-pVTZ	CMA-0A MP2/ cc-pVTZ	CMA-2A MP2/ cc-pVTZ	Pure CCSD(T)/ cc-pVDZ	CMA-0A CCSD(T)/ cc-pVDZ	CMA-2A CCSD(T)/ cc-pVDZ
$\omega_1(a^{'})$	3498.27	3513.80	3498.26	3498.26	3607.43	3498.19	3498.19
$\omega_2(a^{'})$	3079.94	3102.88	3079.58	3079.58	3227.00	3079.49	3079.49
$\omega_3(a^{'})$	2996.98	3013.70	2997.35	2997.35	3131.95	2997.37	2997.37
$\omega_4(a^{'})$	1667.46	1658.61	1667.45	1667.45	1688.68	1667.39	1667.39
$\omega_5(a^{'})$	1508.16	1512.94	1508.13	1508.13	1520.55	1508.07	1508.07
$\omega_6(a^{'})$	1458.06	1456.33	1458.08	1458.08	1481.11	1458.27	1458.27
$\omega_7(a^{'})$	1188.02	1185.97	1187.95	1188.04	1200.23	1188.04	1188.11
$\omega_8(a^{'})$	1065.57	1066.66	1065.60	1065.60	1089.75	1065.55	1065.53
$\omega_9(a^{'})$	878.35	872.18	878.51	878.39	886.06	878.91	878.83
$\omega_{10}(a^{''})$	3579.12	3605.78	3579.12	3579.12	3697.95	3579.12	3579.12
$\omega_{11}(a^{''})$	3115.36	3141.51	3115.36	3115.36	3268.68	3115.33	3115.33
$\omega_{12}(a^{''})$	1527.65	1532.76	1527.65	1527.65	1540.08	1527.69	1527.69
$\omega_{13}(a^{''})$	1359.39	1355.66	1359.40	1359.40	1366.58	1359.29	1359.29
$\omega_{14}(a^{''})$	976.31	977.96	976.32	976.32	984.61	976.50	976.50
$\omega_{15}(a^{''})$	306.35	309.07	306.36	306.35	337.92	306.39	306.38

Table S103: CMA-0A and CMA-2A ($\xi = 0.02$) harmonic frequencies targeting CCSD(T)/cc-pVTZ for Level B = B3LYP/6-31G(2*df*,*p*).

	Pure B3LYP/ 6-31G(2*df*,*p*)	CMA-0A B3LYP/ 6-31G(2*df*,*p*)	CMA-2A B3LYP/ 6-31G(2*df*,*p*)
$\omega_1(a^{'})$	3515.53	3498.26	3498.26
$\omega_2(a^{'})$	3097.85	3079.76	3079.76
$\omega_3(a^{'})$	3012.70	2997.11	2997.11
$\omega_4(a^{'})$	1663.51	1667.42	1667.42
$\omega_5(a^{'})$	1506.01	1507.95	1507.95
$\omega_6(a^{'})$	1462.93	1458.17	1458.17
$\omega_7(a^{'})$	1188.01	1188.05	1188.07
$\omega_8(a^{'})$	1059.75	1065.20	1065.72
$\omega_9(a^{'})$	873.65	879.28	878.62
$\omega_{10}(a^{''})$	3590.81	3579.12	3579.12
$\omega_{11}(a^{''})$	3134.22	3115.35	3115.35
$\omega_{12}(a^{''})$	1523.99	1527.64	1527.64
$\omega_{13}(a^{''})$	1368.69	1359.40	1359.40
$\omega_{14}(a^{''})$	983.01	976.36	976.36
$\omega_{15}(a^{''})$	308.68	306.56	306.56

Table S104: Symmetrized, unnormalized natural internal coordinates for methylamine.

1	$r_{1,2}$
2	$r_{2,3} + r_{2,4}$
3	$r_{2,3} - r_{2,4}$
4	$r_{1,5} + r_{1,6} + r_{1,7}$
5	$-r_{1,5} - r_{1,6} + 2r_{1,7}$
6	$r_{1,5} - r_{1,6}$
7	$2\phi_{3,2,4} - \phi_{1,2,3} - \phi_{1,2,4}$
8	$\phi_{1,2,3} - \phi_{1,2,4}$
9	$\phi_{2,1,7} + \phi_{2,1,5} + \phi_{2,1,6} - \phi_{5,1,6} - \phi_{5,1,7} - \phi_{6,1,7}$
10	$2\phi_{2,1,7} - \phi_{2,1,5} - \phi_{2,1,6}$
11	$\phi_{2,1,5} - \phi_{2,1,6}$
12	$2\phi_{5,1,6} - \phi_{5,1,7} - \phi_{6,1,7}$
13	$\phi_{5,1,7} - \phi_{6,1,7}$
14	$\tau_{3,2,1,5} + \tau_{3,2,1,6} + \tau_{3,2,1,7} + \tau_{4,2,1,5} + \tau_{4,2,1,6} + \tau_{4,2,1,7}$
15	$\gamma_{1,2,3,4}$

S4.27 ethylene

Table S105: CCSD(T)/cc-pVTZ Optimum Cartesian coordinates (bohr)

1	C	1.26340450	0.00000000	0.00000000
2	H	2.33147619	−1.74616425	0.00000000
3	H	2.33147619	1.74616425	0.00000000
4	C	−1.26340450	0.00000000	−0.00000000
5	H	−2.33147619	−1.74616425	−0.00000000
6	H	−2.33147619	1.74616425	−0.00000000

Table S106: Reference [CCSD(T)/cc-pVTZ] CMA-0A and CMA-2A ($\xi = 0.02$) harmonic frequencies for Level B = MP2/cc-pVTZ and CCSD(T)/cc-pVDZ.

	Reference CCSD(T)/ cc-pVTZ	Pure MP2/ cc-pVTZ	CMA-0A MP2/ cc-pVTZ	CMA-2A MP2/ cc-pVTZ	Pure CCSD(T)/ cc-pVDZ	CMA-0A CCSD(T)/ cc-pVDZ	CMA-2A CCSD(T)/ cc-pVDZ
$\omega_1(a_g)$	3157.09	3173.05	3157.09	3157.09	3302.91	3157.07	3157.07
$\omega_2(a_g)$	1671.73	1666.28	1671.73	1671.73	1730.97	1670.53	1671.76
$\omega_3(a_g)$	1368.98	1373.21	1368.99	1368.99	1397.13	1370.49	1368.99
$\omega_4(b_{1g})$	3219.26	3241.74	3219.26	3219.26	3378.07	3219.25	3219.25
$\omega_5(b_{1g})$	1242.16	1238.75	1242.16	1242.16	1256.86	1242.16	1242.16
$\omega_6(a_u)$	1046.96	1070.99	1046.96	1046.96	1057.24	1046.96	1046.96
$\omega_7(b_{1u})$	966.67	976.13	966.67	966.67	976.39	966.67	966.67
$\omega_8(b_{1g})$	941.84	950.10	941.84	941.84	946.31	941.84	941.84
$\omega_9(b_{2u})$	3246.14	3268.43	3246.13	3246.13	3403.30	3246.13	3246.13
$\omega_{10}(b_{2u})$	823.04	820.93	823.05	823.05	842.59	823.06	823.06
$\omega_{11}(b_{3g})$	3139.11	3155.69	3139.11	3139.11	3282.57	3139.11	3139.11
$\omega_{12}(b_{3g})$	1479.06	1479.21	1479.06	1479.06	1494.46	1479.06	1479.06

Table S107: CMA-0A and CMA-2A ($\xi = 0.02$) harmonic frequencies targeting CCSD(T)/cc-pVTZ for Level B = B3LYP/6-31G(2*df*,*p*).

	Pure B3LYP/ 6-31G(2*df*,*p*)	CMA-0A B3LYP/ 6-31G(2*df*,*p*)	CMA-2A B3LYP/ 6-31G(2*df*,*p*)
$\omega_1(a_g)$	3169.38	3157.08	3157.08
$\omega_2(a_g)$	1668.68	1671.30	1671.76
$\omega_3(a_g)$	1376.90	1369.54	1368.98
$\omega_4(b_{1g})$	3235.08	3219.25	3219.25
$\omega_5(b_{1g})$	1241.70	1242.17	1242.17
$\omega_6(a_u)$	1063.67	1046.96	1046.96
$\omega_7(b_{1u})$	985.19	966.67	966.67
$\omega_8(b_{1g})$	968.89	941.84	941.84
$\omega_9(b_{2u})$	3263.59	3246.13	3246.13
$\omega_{10}(b_{2u})$	825.77	823.06	823.06
$\omega_{11}(b_{3g})$	3154.08	3139.11	3139.11
$\omega_{12}(b_{3g})$	1470.17	1479.06	1479.06

Table S108: Symmetrized, unnormalized natural internal coordinates for ethylene.

1	$r_{1,4}$
2	$r_{1,2} + r_{1,3} + r_{4,5} + r_{4,6}$
3	$r_{1,2} + r_{1,3} - r_{4,5} - r_{4,6}$
4	$r_{1,2} - r_{1,3} + r_{4,5} - r_{4,6}$
5	$r_{1,2} - r_{1,3} - r_{4,5} + r_{4,6}$
6	$2\phi_{2,1,3} - \phi_{2,1,4} - \phi_{3,1,4} + 2\phi_{6,4,5} - \phi_{5,4,1} - \phi_{6,4,1}$
7	$\phi_{2,1,4} - \phi_{3,1,4} + \phi_{5,4,1} - \phi_{6,4,1}$
8	$2\phi_{2,1,3} - \phi_{2,1,4} - \phi_{3,1,4} - 2\phi_{6,4,5} + \phi_{5,4,1} + \phi_{6,4,1}$
9	$\phi_{2,1,4} - \phi_{3,1,4} - \phi_{5,4,1} + \phi_{6,4,1}$
10	$\tau_{2,1,4,5} + \tau_{3,1,4,6}$
11	$\gamma_{1,4,5,6} + \gamma_{4,1,2,3}$
12	$\gamma_{1,4,5,6} - \gamma_{4,1,2,3}$

S4.28 tetrafluoroethylene

Table S109: CCSD(T)/cc-pVTZ Optimum Cartesian coordinates (bohr)

1	C	−0.00000000	1.25411723	−0.00000000
2	F	−0.00000000	2.61971410	−2.07863349
3	F	−0.00000000	2.61971408	2.07863350
4	C	0.00000000	−1.25411723	−0.00000000
5	F	0.00000000	−2.61971410	−2.07863349
6	F	0.00000000	−2.61971408	2.07863350

Table S110: Reference [CCSD(T)/cc-pVTZ] CMA-0A and CMA-2A ($\xi = 0.02$) harmonic frequencies for Level B = MP2/cc-pVTZ and CCSD(T)/cc-pVDZ.

	Reference CCSD(T)/ cc-pVTZ	Pure MP2/ cc-pVTZ	CMA-0A MP2/ cc-pVTZ	CMA-2A MP2/ cc-pVTZ	Pure CCSD(T)/ cc-pVDZ	CMA-0A CCSD(T)/ cc-pVDZ	CMA-2A CCSD(T)/ cc-pVDZ
$\omega_1(a_g)$	1921.15	1919.42	1921.15	1921.15	1984.36	1921.11	1921.13
$\omega_2(a_g)$	797.32	798.27	797.32	797.32	811.70	797.37	797.32
$\omega_3(a_g)$	401.04	401.71	401.04	401.04	405.23	401.15	401.15
$\omega_4(b_{1g})$	1385.81	1374.39	1385.81	1385.81	1428.34	1385.81	1385.81
$\omega_5(b_{1g})$	556.41	557.58	556.41	556.41	561.04	556.42	556.41
$\omega_6(a_u)$	201.37	204.23	201.37	201.37	203.00	201.37	201.37
$\omega_7(b_{1u})$	418.44	428.50	418.44	418.44	434.47	418.44	418.44
$\omega_8(b_{1g})$	511.82	540.66	511.82	511.82	530.08	511.82	511.82
$\omega_9(b_{2u})$	1376.54	1370.04	1376.54	1376.54	1412.56	1376.54	1376.54
$\omega_{10}(b_{2u})$	210.88	210.73	210.88	210.88	209.16	210.89	210.89
$\omega_{11}(b_{3g})$	1208.55	1209.08	1208.55	1208.55	1229.18	1208.54	1208.55
$\omega_{12}(b_{3g})$	559.79	560.96	559.79	559.79	567.54	559.81	559.79

Table S111: CMA-0A and CMA-2A ($\xi = 0.02$) harmonic frequencies targeting CCSD(T)/cc-pVTZ for Level B = B3LYP/6-31G(2*df*,*p*).

	Pure B3LYP/ 6-31G(2*df*,*p*)	CMA-0A B3LYP/ 6-31G(2*df*,*p*)	CMA-2A B3LYP/ 6-31G(2*df*,*p*)
$\omega_1(a_g)$	1917.45	1921.15	1921.15
$\omega_2(a_g)$	804.42	797.32	797.31
$\omega_3(a_g)$	398.54	401.09	401.09
$\omega_4(b_{1g})$	1361.88	1385.77	1385.81
$\omega_5(b_{1g})$	550.83	556.51	556.41
$\omega_6(a_u)$	198.84	201.37	201.37
$\omega_7(b_{1u})$	435.47	418.44	418.44
$\omega_8(b_{1g})$	549.15	511.82	511.82
$\omega_9(b_{2u})$	1368.62	1376.53	1376.53
$\omega_{10}(b_{2u})$	204.70	210.92	210.92
$\omega_{11}(b_{3g})$	1212.22	1208.55	1208.55
$\omega_{12}(b_{3g})$	556.45	559.79	559.79

Table S112: Symmetrized, unnormalized natural internal coordinates for tetrafluoroethylene.

1	$r_{1,4}$
2	$r_{1,2} + r_{1,3} + r_{4,5} + r_{4,6}$
3	$r_{1,2} + r_{1,3} - r_{4,5} - r_{4,6}$
4	$r_{1,2} - r_{1,3} + r_{4,5} - r_{4,6}$
5	$r_{1,2} - r_{1,3} - r_{4,5} + r_{4,6}$
6	$2\phi_{2,1,3} - \phi_{2,1,4} - \phi_{3,1,4} + 2\phi_{6,4,5} - \phi_{5,4,1} - \phi_{6,4,1}$
7	$\phi_{2,1,4} - \phi_{3,1,4} + \phi_{5,4,1} - \phi_{6,4,1}$
8	$2\phi_{2,1,3} - \phi_{2,1,4} - \phi_{3,1,4} - 2\phi_{6,4,5} + \phi_{5,4,1} + \phi_{6,4,1}$
9	$\phi_{2,1,4} - \phi_{3,1,4} - \phi_{5,4,1} + \phi_{6,4,1}$
10	$\tau_{2,1,4,5} + \tau_{3,1,4,6}$
11	$\gamma_{1,4,5,6} + \gamma_{4,1,2,3}$
12	$\gamma_{1,4,5,6} - \gamma_{4,1,2,3}$

S4.29 tetrachloroethylene

Table S113: CCSD(T)/cc-pVTZ Optimum Cartesian coordinates (bohr)

1	C	1.27393262	0.00000000	0.00000000
2	Cl	3.00539606	−2.74614238	0.00000000
3	Cl	3.00539606	2.74614238	0.00000000
4	C	−1.27393262	0.00000000	0.00000000
5	Cl	−3.00539606	−2.74614238	0.00000000
6	Cl	−3.00539606	2.74614238	0.00000000

Table S114: Reference [CCSD(T)/cc-pVTZ] CMA-0A and CMA-2A ($\xi = 0.02$) harmonic frequencies for Level B = MP2/cc-pVTZ and CCSD(T)/cc-pVDZ.

	Reference CCSD(T)/ cc-pVTZ	Pure MP2/ cc-pVTZ	CMA-0A MP2/ cc-pVTZ	CMA-2A MP2/ cc-pVTZ	Pure CCSD(T)/ cc-pVDZ	CMA-0A CCSD(T)/ cc-pVDZ	CMA-2A CCSD(T)/ cc-pVDZ
$\omega_1(a_g)$	1614.67	1603.94	1614.67	1614.67	1685.77	1614.67	1614.67
$\omega_2(a_g)$	452.08	453.45	452.09	452.09	464.25	452.08	452.08
$\omega_3(a_g)$	236.38	235.06	236.38	236.38	245.04	236.39	236.39
$\omega_4(b_{1g})$	1009.16	996.91	1009.15	1009.16	1043.09	1009.16	1009.16
$\omega_5(b_{1g})$	348.10	348.41	348.12	348.10	356.04	348.10	348.10
$\omega_6(a_u)$	99.15	101.12	99.15	99.15	98.52	99.15	99.15
$\omega_7(b_{1u})$	288.16	287.43	288.16	288.16	296.42	288.16	288.16
$\omega_8(b_{1g})$	517.55	517.15	517.55	517.55	518.52	517.55	517.55
$\omega_9(b_{2u})$	928.96	928.16	928.96	928.96	958.21	928.96	928.96
$\omega_{10}(b_{2u})$	176.42	173.74	176.42	176.42	184.05	176.42	176.42
$\omega_{11}(b_{3g})$	785.28	785.62	785.28	785.28	801.60	785.28	785.28
$\omega_{12}(b_{3g})$	312.17	312.52	312.18	312.17	322.08	312.17	312.17

Table S115: CMA-0A and CMA-2A ($\xi = 0.02$) harmonic frequencies targeting CCSD(T)/cc-pVTZ for Level B = B3LYP/6-31G(2*df*,*p*).

	Pure B3LYP/ 6-31G(2*df*,*p*)	CMA-0A B3LYP/ 6-31G(2*df*,*p*)	CMA-2A B3LYP/ 6-31G(2*df*,*p*)
$\omega_1(a_g)$	1612.45	1614.65	1614.65
$\omega_2(a_g)$	453.76	452.15	452.15
$\omega_3(a_g)$	238.72	236.38	236.38
$\omega_4(b_{1g})$	987.18	1009.09	1009.09
$\omega_5(b_{1g})$	350.25	348.30	348.30
$\omega_6(a_u)$	98.53	99.15	99.15
$\omega_7(b_{1u})$	295.64	288.16	288.16
$\omega_8(b_{1g})$	542.52	517.55	517.55
$\omega_9(b_{2u})$	909.02	928.96	928.96
$\omega_{10}(b_{2u})$	178.93	176.43	176.43
$\omega_{11}(b_{3g})$	784.12	785.27	785.27
$\omega_{12}(b_{3g})$	314.42	312.19	312.19

Table S116: Symmetrized, unnormalized natural internal coordinates for tetrachloroethylene.

1	$r_{1,4}$
2	$r_{1,2} + r_{1,3} + r_{4,5} + r_{4,6}$
3	$r_{1,2} + r_{1,3} - r_{4,5} - r_{4,6}$
4	$r_{1,2} - r_{1,3} + r_{4,5} - r_{4,6}$
5	$r_{1,2} - r_{1,3} - r_{4,5} + r_{4,6}$
6	$2\phi_{2,1,3} - \phi_{2,1,4} - \phi_{3,1,4} + 2\phi_{6,4,5} - \phi_{5,4,1} - \phi_{6,4,1}$
7	$\phi_{2,1,4} - \phi_{3,1,4} + \phi_{5,4,1} - \phi_{6,4,1}$
8	$2\phi_{2,1,3} - \phi_{2,1,4} - \phi_{3,1,4} - 2\phi_{6,4,5} + \phi_{5,4,1} + \phi_{6,4,1}$
9	$\phi_{2,1,4} - \phi_{3,1,4} - \phi_{5,4,1} + \phi_{6,4,1}$
10	$\tau_{2,1,4,5} + \tau_{3,1,4,6}$
11	$\gamma_{1,4,5,6} + \gamma_{4,1,2,3}$
12	$\gamma_{1,4,5,6} - \gamma_{4,1,2,3}$

S4.30 acetylene

Table S117: CCSD(T)/cc-pVTZ Optimum Cartesian coordinates (bohr)

1	X	1.00000000	0.00000000	1.14300021
2	C	−0.00000000	0.00000000	1.14300021
3	X	−0.00000000	1.00000000	1.14300021
4	X	1.00000000	0.00000000	−1.14300021
5	C	−0.00000000	0.00000000	−1.14300021
6	X	−0.00000000	1.00000000	−1.14300021
7	H	−0.00000000	0.00000000	3.15309138
8	H	−0.00000000	−0.00000000	−3.15309138

Table S118: Reference [CCSD(T)/cc-pVTZ] CMA-0A and CMA-2A ($\xi = 0.02$) harmonic frequencies for Level B = MP2/cc-pVTZ and CCSD(T)/cc-pVDZ.

	Reference CCSD(T)/ cc-pVTZ	Pure MP2/ cc-pVTZ	CMA-0A MP2/ cc-pVTZ	CMA-2A MP2/ cc-pVTZ	Pure CCSD(T)/ cc-pVDZ	CMA-0A CCSD(T)/ cc-pVDZ	CMA-2A CCSD(T)/ cc-pVDZ
$\omega_1(\sigma_g^+)$	3510.94	3522.34	3510.91	3510.91	3655.38	3510.94	3510.94
$\omega_2(\sigma_g^+)$	2000.86	1983.61	2000.91	2000.91	2096.53	2000.87	2000.87
$\omega_3(\sigma_u^+)$	3409.95	3424.96	3409.95	3409.95	3558.13	3409.95	3409.95
$\omega_{4a}(\pi_u)$	746.28	752.05	746.28	746.28	760.96	746.28	746.28
$\omega_{4b}(\pi_u)$	746.28	752.11	746.28	746.28	761.00	746.28	746.28
$\omega_{5a}(\pi_g)$	577.56	591.06	577.56	577.56	589.88	577.56	577.56
$\omega_{5b}(\pi_g)$	577.56	591.08	577.56	577.56	589.89	577.56	577.56

Table S119: CMA-0A and CMA-2A ($\xi = 0.02$) harmonic frequencies targeting CCSD(T)/cc-pVTZ for Level B = B3LYP/6-31G(2*df*,*p*).

	Pure B3LYP/ 6-31G(2*df*,*p*)	CMA-0A B3LYP/ 6-31G(2*df*,*p*)	CMA-2A B3LYP/ 6-31G(2*df*,*p*)
$\omega_1(\sigma_g^+)$	3530.91	3510.93	3510.93
$\omega_2(\sigma_g^+)$	2020.94	2000.89	2000.89
$\omega_3(\sigma_u^+)$	3433.71	3409.95	3409.95
$\omega_{4a}(\pi_u)$	781.02	746.28	746.28
$\omega_{4b}(\pi_u)$	781.08	746.28	746.28
$\omega_{5a}(\pi_g)$	561.27	577.56	577.56
$\omega_{5b}(\pi_g)$	561.29	577.56	577.56

Table S120: Symmetrized, unnormalized natural internal coordinates for acetylene.

1	$r_{2,5}$
2	$r_{2,7} + r_{5,8}$
3	$r_{2,7} - r_{5,8}$
4	$\theta_{7,2,5,1} + \theta_{7,2,5,3}$
5	$\theta_{7,2,5,1} - \theta_{7,2,5,3}$
6	$\theta_{8,5,2,4} + \theta_{8,5,2,6}$
7	$\theta_{8,5,2,4} - \theta_{8,5,2,6}$

S4.31 glyoxal

Table S121: CCSD(T)/cc-pVTZ Optimum Cartesian coordinates (bohr)

1	C	1.21889603	−0.76024287	−0.00000000
2	H	0.96585913	−2.82956991	−0.00000000
3	O	3.26409381	0.26574464	−0.00000000
4	C	−1.21889603	0.76024286	0.00000000
5	H	−0.96585912	2.82956990	0.00000000
6	O	−3.26409381	−0.26574463	0.00000000

Table S122: Reference [CCSD(T)/cc-pVTZ] CMA-0A and CMA-2A ($\xi = 0.02$) harmonic frequencies for Level B = MP2/cc-pVTZ and CCSD(T)/cc-pVDZ.

	Reference CCSD(T)/ cc-pVTZ	Pure MP2/ cc-pVTZ	CMA-0A MP2/ cc-pVTZ	CMA-2A MP2/ cc-pVTZ	Pure CCSD(T)/ cc-pVDZ	CMA-0A CCSD(T)/ cc-pVDZ	CMA-2A CCSD(T)/ cc-pVDZ
$\omega_1(a_\mathrm{g})$	2987.10	3002.93	2987.09	2987.09	3125.99	2987.09	2987.09
$\omega_2(a_\mathrm{g})$	1779.77	1763.00	1779.71	1779.77	1833.16	1779.61	1779.61
$\omega_3(a_\mathrm{g})$	1384.84	1387.70	1384.84	1384.83	1405.09	1384.74	1384.74
$\omega_4(a_\mathrm{g})$	1095.40	1095.62	1095.49	1095.41	1114.23	1095.64	1095.64
$\omega_5(a_\mathrm{g})$	560.06	557.99	560.08	560.07	572.68	560.35	560.35
$\omega_6(b_\mathrm{g})$	1068.80	1079.34	1068.80	1068.80	1084.11	1068.80	1068.80
$\omega_7(a_\mathrm{u})$	823.86	832.67	823.85	823.86	840.46	823.85	823.86
$\omega_8(a_\mathrm{u})$	136.26	135.79	136.32	136.26	151.02	136.32	136.26
$\omega_9(b_\mathrm{u})$	2982.13	2998.22	2982.13	2982.13	3118.06	2982.12	2982.12
$\omega_{10}(b_\mathrm{u})$	1757.89	1750.67	1757.89	1757.89	1807.47	1757.88	1757.88
$\omega_{11}(b_\mathrm{u})$	1342.00	1343.96	1342.01	1342.01	1356.82	1342.04	1342.04
$\omega_{12}(b_\mathrm{u})$	330.98	329.78	330.98	330.98	331.01	331.00	330.98

Table S123: CMA-0A and CMA-2A ($\xi = 0.02$) harmonic frequencies targeting CCSD(T)/cc-pVTZ for Level B = B3LYP/6-31G(2*df*,*p*).

	Pure B3LYP/ 6-31G(2*df*,*p*)	CMA-0A B3LYP/ 6-31G(2*df*,*p*)	CMA-2A B3LYP/ 6-31G(2*df*,*p*)
$\omega_1(a_\mathrm{g})$	3002.37	2987.04	2987.04
$\omega_2(a_\mathrm{g})$	1785.75	1779.39	1779.76
$\omega_3(a_\mathrm{g})$	1385.83	1384.73	1384.69
$\omega_4(a_\mathrm{g})$	1076.50	1096.18	1095.75
$\omega_5(a_\mathrm{g})$	555.99	560.31	560.07
$\omega_6(b_\mathrm{g})$	1087.79	1068.80	1068.80
$\omega_7(a_\mathrm{u})$	827.78	823.81	823.86
$\omega_8(a_\mathrm{u})$	138.09	136.59	136.26
$\omega_9(b_\mathrm{u})$	2997.19	2982.11	2982.11
$\omega_{10}(b_\mathrm{u})$	1780.03	1757.87	1757.87
$\omega_{11}(b_\mathrm{u})$	1340.17	1342.07	1342.07
$\omega_{12}(b_\mathrm{u})$	330.09	330.98	330.98

Table S124: Symmetrized, unnormalized natural internal coordinates for glyoxal.

1	$r_{1,4}$
2	$r_{1,2} + r_{4,5}$
3	$r_{1,2} - r_{4,5}$
4	$r_{1,3} + r_{4,6}$
5	$r_{1,3} - r_{4,6}$
6	$\phi_{2,1,4} - \phi_{2,1,3} + \phi_{5,4,1} - \phi_{5,4,6}$
7	$\phi_{2,1,4} - \phi_{2,1,3} - \phi_{5,4,1} + \phi_{5,4,6}$
8	$-\phi_{2,1,4} - \phi_{2,1,3} + 2\phi_{4,1,3} - \phi_{5,4,1} - \phi_{5,4,6} + 2\phi_{1,4,6}$
9	$-\phi_{2,1,4} - \phi_{2,1,3} + 2\phi_{4,1,3} + \phi_{5,4,1} + \phi_{5,4,6} - 2\phi_{1,4,6}$
10	$\tau_{6,4,1,3}$
11	$\gamma_{5,4,6,1} + \gamma_{2,1,3,4}$
12	$\gamma_{5,4,6,1} - \gamma_{2,1,3,4}$

S4.32 ketene

Table S125: CCSD(T)/cc-pVTZ Optimum Cartesian coordinates (bohr)

1	C	−0.00000000	0.00000000	−2.45258708
2	C	−0.00000000	0.00000000	0.03916157
3	H	1.77986810	0.00000000	−3.44221703
4	H	−1.77986810	−0.00000000	−3.44221703
5	O	0.00000000	−0.00000000	2.24442655

Table S126: Reference [CCSD(T)/cc-pVTZ] CMA-0A and CMA-2A ($\xi = 0.02$) harmonic frequencies for Level B = MP2/cc-pVTZ and CCSD(T)/cc-pVDZ.

	Reference CCSD(T)/ cc-pVTZ	Pure MP2/ cc-pVTZ	CMA-0A MP2/ cc-pVTZ	CMA-2A MP2/ cc-pVTZ	Pure CCSD(T)/ cc-pVDZ	CMA-0A CCSD(T)/ cc-pVDZ	CMA-2A CCSD(T)/ cc-pVDZ
$\omega_1(a_1)$	3201.09	3215.62	3201.09	3201.09	3342.90	3201.07	3201.07
$\omega_2(a_1)$	2196.66	2215.50	2196.65	2196.65	2279.64	2196.45	2196.45
$\omega_3(a_1)$	1419.96	1412.28	1419.97	1419.97	1446.54	1418.82	1420.12
$\omega_4(a_1)$	1151.97	1152.32	1151.98	1151.98	1189.88	1153.82	1152.23
$\omega_5(b_1)$	590.76	583.94	584.48	590.76	594.23	590.15	590.76
$\omega_6(b_1)$	514.86	504.86	521.98	514.86	522.51	515.56	514.86
$\omega_7(b_2)$	3305.76	3323.30	3305.76	3305.76	3464.40	3305.74	3305.74
$\omega_8(b_2)$	993.60	986.64	993.51	993.51	1008.27	993.65	993.65
$\omega_9(b_2)$	434.17	432.90	434.38	434.38	443.74	434.20	434.20

Table S127: CMA-0A and CMA-2A ($\xi = 0.02$) harmonic frequencies targeting CCSD(T)/cc-pVTZ for Level B = B3LYP/6-31G(2df,p).

	Pure B3LYP/ 6-31G(2df,p)	CMA-0A B3LYP/ 6-31G(2df,p)	CMA-2A B3LYP/ 6-31G(2df,p)
$\omega_1(a_1)$	3213.56	3201.06	3201.06
$\omega_2(a_1)$	2211.41	2196.57	2196.57
$\omega_3(a_1)$	1403.07	1419.93	1420.05
$\omega_4(a_1)$	1156.46	1152.28	1152.12
$\omega_5(b_1)$	591.58	590.47	590.76
$\omega_6(b_1)$	536.30	515.19	514.86
$\omega_7(b_2)$	3320.40	3305.75	3305.75
$\omega_8(b_2)$	992.25	993.42	993.42
$\omega_9(b_2)$	444.31	434.65	434.65

Table S128: Symmetrized, unnormalized natural internal coordinates for ketene.

1	$r_{1,2}$
2	$r_{2,5}$
3	$r_{1,3} + r_{1,4}$
4	$r_{1,3} - r_{1,4}$
5	$2\phi_{3,1,4} - \phi_{3,1,2} - \phi_{4,1,2}$
6	$\phi_{3,1,2} - \phi_{4,1,2}$
7	$\gamma_{2,1,3,4}$
8	$\alpha^{x}_{3,1,2,5} - \alpha^{x}_{4,1,2,5}$
9	$\alpha^{y}_{3,1,2,5} - \alpha^{y}_{4,1,2,5}$

S4.33 vinyl fluoride

Table S129: CCSD(T)/cc-pVTZ Optimum Cartesian coordinates (bohr)

1	C	−0.23062727	0.84927217	0.00000000
2	F	2.01489057	−0.33348284	−0.00000000
3	H	−0.03883537	2.88455505	0.00000000
4	C	−2.40187281	−0.41086779	−0.00000000
5	H	−2.44489109	−2.45275480	−0.00000000
6	H	−4.15403542	0.63464361	−0.00000000

Table S130: Reference [CCSD(T)/cc-pVTZ] CMA-0A and CMA-2A ($\xi = 0.02$) harmonic frequencies for Level B = MP2/cc-pVTZ and CCSD(T)/cc-pVDZ.

	Reference CCSD(T)/ cc-pVTZ	Pure MP2/ cc-pVTZ	CMA-0A MP2/ cc-pVTZ	CMA-2A MP2/ cc-pVTZ	Pure CCSD(T)/ cc-pVDZ	CMA-0A CCSD(T)/ cc-pVDZ	CMA-2A CCSD(T)/ cc-pVDZ
$\omega_1(a^{'})$	3280.45	3301.10	3280.45	3280.45	3436.28	3280.40	3280.40
$\omega_2(a^{'})$	3216.73	3235.18	3216.71	3216.71	3365.94	3216.75	3216.75
$\omega_3(a^{'})$	3178.28	3194.10	3178.29	3178.29	3320.33	3178.29	3178.29
$\omega_4(a^{'})$	1703.22	1696.19	1703.21	1703.21	1773.90	1702.57	1702.99
$\omega_5(a^{'})$	1424.87	1422.88	1424.85	1424.85	1444.35	1424.97	1424.97
$\omega_6(a^{'})$	1335.38	1337.10	1335.38	1335.37	1355.00	1335.85	1335.32
$\omega_7(a^{'})$	1186.16	1183.93	1186.17	1186.20	1205.74	1186.31	1186.37
$\omega_8(a^{'})$	945.55	942.22	945.59	945.56	964.54	945.73	945.65
$\omega_9(a^{'})$	481.38	481.34	481.41	481.38	485.72	481.48	481.48
$\omega_{10}(a^{''})$	956.17	972.47	956.08	956.08	975.60	956.07	956.07
$\omega_{11}(a^{''})$	871.03	870.90	871.08	871.08	876.41	871.04	871.04
$\omega_{12}(a^{''})$	725.01	737.59	725.08	725.07	736.40	725.14	725.14

Table S131: CMA-0A and CMA-2A ($\xi = 0.02$) harmonic frequencies targeting CCSD(T)/cc-pVTZ for Level B = B3LYP/6-31G(2*df*,*p*).

	Pure B3LYP/ 6-31G(2*df*,*p*)	CMA-0A B3LYP/ 6-31G(2*df*,*p*)	CMA-2A B3LYP/ 6-31G(2*df*,*p*)
$\omega_1(a^{'})$	3295.43	3280.44	3280.44
$\omega_2(a^{'})$	3232.78	3216.65	3216.65
$\omega_3(a^{'})$	3190.55	3178.33	3178.33
$\omega_4(a^{'})$	1699.95	1702.90	1703.10
$\omega_5(a^{'})$	1412.57	1424.92	1424.92
$\omega_6(a^{'})$	1335.34	1335.63	1335.37
$\omega_7(a^{'})$	1178.16	1186.20	1186.23
$\omega_8(a^{'})$	943.19	945.73	945.70
$\omega_9(a^{'})$	479.06	481.47	481.47
$\omega_{10}(a^{''})$	974.58	955.96	955.96
$\omega_{11}(a^{''})$	886.14	871.00	871.00
$\omega_{12}(a^{''})$	732.93	725.32	725.32

Table S132: Symmetrized, unnormalized natural internal coordinates for vinyl fluoride.

1	$r_{1,4}$
2	$r_{1,2}$
3	$r_{1,3}$
4	$r_{4,5} + r_{4,6}$
5	$r_{4,5} - r_{4,6}$
6	$\phi_{2,1,4}$
7	$\phi_{2,1,3} - \phi_{4,1,3}$
8	$2\phi_{5,4,6} - \phi_{5,4,1} - \phi_{6,4,1}$
9	$\phi_{5,4,1} - \phi_{6,4,1}$
10	$\tau_{6,4,1,2} + \tau_{5,4,1,2}$
11	$\gamma_{1,4,5,6}$
12	$\gamma_{3,1,2,4}$

S4.34 vinyl chloride

Table S133: CCSD(T)/cc-pVTZ Optimum Cartesian coordinates (bohr)

1	C	−1.25457203	0.96043344	0.00000000
2	Cl	1.82672009	−0.15681495	0.00000000
3	H	−1.37832434	2.99846821	0.00000000
4	C	−3.26305888	−0.55916494	0.00000000
5	H	−3.07637332	−2.59407651	0.00000000
6	H	−5.13698193	0.25883536	0.00000000

Table S134: Reference [CCSD(T)/cc-pVTZ] CMA-0A and CMA-2A ($\xi = 0.02$) harmonic frequencies for Level B = MP2/cc-pVTZ and CCSD(T)/cc-pVDZ.

	Reference CCSD(T)/ cc-pVTZ	Pure MP2/ cc-pVTZ	CMA-0A MP2/ cc-pVTZ	CMA-2A MP2/ cc-pVTZ	Pure CCSD(T)/ cc-pVDZ	CMA-0A CCSD(T)/ cc-pVDZ	CMA-2A CCSD(T)/ cc-pVDZ
$\omega_1(a^{'})$	3261.58	3282.14	3261.58	3261.58	3416.72	3261.54	3261.54
$\omega_2(a^{'})$	3219.69	3237.30	3219.69	3219.69	3369.32	3219.72	3219.72
$\omega_3(a^{'})$	3163.14	3178.42	3163.14	3163.14	3305.00	3163.14	3163.14
$\omega_4(a^{'})$	1648.92	1641.17	1648.91	1648.91	1716.38	1647.85	1648.82
$\omega_5(a^{'})$	1409.03	1408.23	1409.00	1409.00	1430.65	1410.12	1408.98
$\omega_6(a^{'})$	1303.91	1303.03	1303.92	1303.92	1324.34	1304.05	1304.05
$\omega_7(a^{'})$	1043.79	1039.71	1043.81	1043.84	1061.50	1043.84	1043.84
$\omega_8(a^{'})$	728.91	732.93	728.95	728.91	747.79	728.94	728.94
$\omega_9(a^{'})$	393.16	393.28	393.17	393.17	402.36	393.21	393.21
$\omega_{10}(a^{''})$	967.57	982.66	967.47	967.47	979.77	967.56	967.56
$\omega_{11}(a^{''})$	906.85	907.33	906.95	906.95	913.38	906.84	906.85
$\omega_{12}(a^{''})$	624.92	636.66	624.93	624.92	630.19	624.94	624.93

Table S135: CMA-0A and CMA-2A ($\xi = 0.02$) harmonic frequencies targeting CCSD(T)/cc-pVTZ for Level B = B3LYP/6-31G(2*df*,*p*).

	Pure B3LYP/ 6-31G(2*df*,*p*)	CMA-0A B3LYP/ 6-31G(2*df*,*p*)	CMA-2A B3LYP/ 6-31G(2*df*,*p*)
$\omega_1(a^{'})$	3279.03	3261.57	3261.57
$\omega_2(a^{'})$	3236.46	3219.65	3219.65
$\omega_3(a^{'})$	3177.27	3163.16	3163.16
$\omega_4(a^{'})$	1645.15	1648.45	1648.69
$\omega_5(a^{'})$	1403.09	1408.88	1408.59
$\omega_6(a^{'})$	1301.18	1304.62	1304.62
$\omega_7(a^{'})$	1040.95	1043.78	1043.78
$\omega_8(a^{'})$	724.68	729.13	729.13
$\omega_9(a^{'})$	398.62	393.17	393.17
$\omega_{10}(a^{''})$	977.28	967.53	967.53
$\omega_{11}(a^{''})$	926.18	906.80	906.80
$\omega_{12}(a^{''})$	634.75	625.04	625.04

Table S136: Symmetrized, unnormalized natural internal coordinates for vinyl chloride.

1	$r_{1,4}$
2	$r_{1,2}$
3	$r_{1,3}$
4	$r_{4,5} + r_{4,6}$
5	$r_{4,5} - r_{4,6}$
6	$\phi_{2,1,4}$
7	$\phi_{2,1,3} - \phi_{3,1,4}$
8	$2\phi_{5,4,6} - \phi_{5,4,1} - \phi_{6,4,1}$
9	$\phi_{5,4,1} - \phi_{6,4,1}$
10	$\tau_{2,1,4,6} + \tau_{3,1,4,5}$
11	$\gamma_{1,4,5,6}$
12	$\gamma_{4,1,2,3}$

S4.35 acetyl chloride

Table S137: CCSD(T)/cc-pVTZ Optimum Cartesian coordinates (bohr)

1	C	2.37756991	2.26850483	−0.00055361
2	C	1.05905955	−0.24683567	0.00014473
3	O	2.00061832	−2.28726397	0.00084009
4	Cl	−2.32530065	0.10341395	−0.00018125
5	H	4.41124345	1.96733004	−0.00032789
6	H	1.80005376	3.33615707	−1.66508115
7	H	1.79983852	3.33719391	1.66323370

Table S138: Reference [CCSD(T)/cc-pVTZ] CMA-0A and CMA-2A ($\xi = 0.02$) harmonic frequencies for Level B = MP2/cc-pVTZ and CCSD(T)/cc-pVDZ.

	Reference CCSD(T)/ cc-pVTZ	Pure MP2/ cc-pVTZ	CMA-0A MP2/ cc-pVTZ	CMA-2A MP2/ cc-pVTZ	Pure CCSD(T)/ cc-pVDZ	CMA-0A CCSD(T)/ cc-pVDZ	CMA-2A CCSD(T)/ cc-pVDZ
$\omega_1(a^{'})$	3169.14	3190.54	3169.13	3169.13	3319.54	3169.09	3169.09
$\omega_2(a^{'})$	3062.21	3073.65	3062.22	3062.22	3193.58	3062.20	3062.20
$\omega_3(a^{'})$	1859.03	1852.22	1859.01	1859.01	1917.69	1858.97	1858.99
$\omega_4(a^{'})$	1472.26	1473.34	1472.22	1472.22	1483.82	1472.29	1472.29
$\omega_5(a^{'})$	1394.09	1384.82	1394.11	1394.11	1412.92	1393.84	1393.84
$\omega_6(a^{'})$	1127.04	1120.31	1127.03	1127.06	1150.19	1126.46	1127.23
$\omega_7(a^{'})$	969.93	965.32	969.97	969.95	997.22	971.16	970.28
$\omega_8(a^{'})$	613.58	610.48	613.57	613.54	626.63	613.66	613.59
$\omega_9(a^{'})$	448.70	447.58	448.83	448.83	457.00	448.73	448.72
$\omega_{10}(a^{'})$	343.07	343.30	343.09	343.09	351.72	343.11	343.11
$\omega_{11}(a^{''})$	3142.85	3165.11	3142.85	3142.85	3293.35	3142.81	3142.81
$\omega_{12}(a^{''})$	1478.20	1479.93	1478.19	1478.19	1491.48	1478.20	1478.20
$\omega_{13}(a^{''})$	1047.49	1043.66	1047.49	1047.49	1057.82	1047.60	1047.60
$\omega_{14}(a^{''})$	518.05	518.41	518.07	518.07	527.21	518.06	518.06
$\omega_{15}(a^{''})$	143.29	141.37	143.38	143.38	161.90	143.36	143.35

Table S139: CMA-0A and CMA-2A ($\xi = 0.02$) harmonic frequencies targeting CCSD(T)/cc-pVTZ for Level B = B3LYP/6-31G(2*df*,*p*).

	Pure B3LYP/ 6-31G(2*df*,*p*)	CMA-0A B3LYP/ 6-31G(2*df*,*p*)	CMA-2A B3LYP/ 6-31G(2*df*,*p*)
$\omega_1(a^{'})$	3186.50	3169.13	3169.13
$\omega_2(a^{'})$	3081.54	3062.17	3062.17
$\omega_3(a^{'})$	1872.28	1858.87	1858.93
$\omega_4(a^{'})$	1466.62	1472.04	1472.04
$\omega_5(a^{'})$	1393.53	1394.13	1394.13
$\omega_6(a^{'})$	1123.63	1126.79	1126.79
$\omega_7(a^{'})$	963.13	970.94	970.81
$\omega_8(a^{'})$	616.26	613.58	613.58
$\omega_9(a^{'})$	448.19	448.74	448.74
$\omega_{10}(a^{'})$	347.29	343.12	343.12
$\omega_{11}(a^{''})$	3158.72	3142.85	3142.85
$\omega_{12}(a^{''})$	1470.88	1478.16	1478.16
$\omega_{13}(a^{''})$	1047.55	1047.51	1047.51
$\omega_{14}(a^{''})$	525.03	518.08	518.08
$\omega_{15}(a^{''})$	138.91	143.50	143.47

Table S140: Symmetrized, unnormalized natural internal coordinates for acetyl chloride.

1	$r_{1,2}$
2	$r_{2,3}$
3	$r_{2,4}$
4	$r_{1,5} + r_{1,6} + r_{1,7}$
5	$2r_{1,5} - r_{1,6} - r_{1,7}$
6	$r_{1,6} - r_{1,7}$
7	$2\phi_{3,2,4} - \phi_{1,2,3} - \phi_{1,2,4}$
8	$\phi_{1,2,3} - \phi_{1,2,4}$
9	$\phi_{2,1,5} + \phi_{2,1,7} + \phi_{2,1,6} - \phi_{6,1,7} - \phi_{5,1,6} - \phi_{5,1,7}$
10	$2\phi_{2,1,5} - \phi_{2,1,7} - \phi_{2,1,6}$
11	$\phi_{2,1,7} - \phi_{2,1,6}$
12	$2\phi_{6,1,7} - \phi_{5,1,6} - \phi_{5,1,7}$
13	$\phi_{5,1,6} - \phi_{5,1,7}$
14	$\tau_{5,1,2,3} + \tau_{6,1,2,3} + \tau_{7,1,2,3} + \tau_{5,1,2,4} + \tau_{6,1,2,4} + \tau_{7,1,2,4}$
15	$\gamma_{4,2,3,1}$

S4.36 acetyl fluoride

Table S141: CCSD(T)/cc-pVTZ Optimum Cartesian coordinates (bohr)

1	C	2.76454271	0.01457545	0.00000000
2	C	−0.04920230	−0.29403538	−0.00000002
3	O	−1.26541246	−2.18009943	0.00000001
4	F	−1.19736497	1.99333285	0.00000000
5	H	3.66400828	−1.82995283	0.00000007
6	H	3.32965655	1.09054359	−1.66356692
7	H	3.32965649	1.09054368	1.66356688

Table S142: Reference [CCSD(T)/cc-pVTZ] CMA-0A and CMA-2A ($\xi = 0.02$) harmonic frequencies for Level B = MP2/cc-pVTZ and CCSD(T)/cc-pVDZ.

	Reference CCSD(T)/ cc-pVTZ	Pure MP2/ cc-pVTZ	CMA-0A MP2/ cc-pVTZ	CMA-2A MP2/ cc-pVTZ	Pure CCSD(T)/ cc-pVDZ	CMA-0A CCSD(T)/ cc-pVDZ	CMA-2A CCSD(T)/ cc-pVDZ
$\omega_1(a^{'})$	3183.94	3205.21	3183.91	3183.91	3333.43	3183.85	3183.85
$\omega_2(a^{'})$	3067.89	3079.93	3067.91	3067.91	3199.43	3067.91	3067.91
$\omega_3(a^{'})$	1905.64	1901.44	1905.63	1905.63	1966.97	1905.52	1905.61
$\omega_4(a^{'})$	1478.05	1480.41	1478.01	1478.01	1489.11	1478.11	1478.11
$\omega_5(a^{'})$	1409.07	1402.51	1409.08	1409.08	1430.07	1408.21	1408.91
$\omega_6(a^{'})$	1229.96	1221.96	1229.93	1229.97	1265.32	1230.37	1229.90
$\omega_7(a^{'})$	1017.14	1013.95	1017.18	1017.15	1032.58	1017.91	1017.43
$\omega_8(a^{'})$	854.81	852.70	854.85	854.82	876.23	854.94	854.85
$\omega_9(a^{'})$	605.07	605.59	605.08	605.08	613.51	605.24	605.24
$\omega_{10}(a^{'})$	412.95	414.29	412.96	412.96	414.39	412.98	412.98
$\omega_{11}(a^{''})$	3141.37	3163.75	3141.37	3141.37	3291.51	3141.33	3141.33
$\omega_{12}(a^{''})$	1486.58	1489.91	1486.58	1486.58	1497.91	1486.55	1486.55
$\omega_{13}(a^{''})$	1073.07	1071.01	1073.06	1073.06	1082.65	1073.19	1073.20
$\omega_{14}(a^{''})$	572.45	574.75	572.47	572.48	579.82	572.47	572.47
$\omega_{15}(a^{''})$	135.38	132.54	135.45	135.43	143.19	135.46	135.43

Table S143: CMA-0A and CMA-2A ($\xi = 0.02$) harmonic frequencies targeting CCSD(T)/cc-pVTZ for Level B = B3LYP/6-31G(2*df*,*p*).

	Pure B3LYP/ 6-31G(2*df*,*p*)	CMA-0A B3LYP/ 6-31G(2*df*,*p*)	CMA-2A B3LYP/ 6-31G(2*df*,*p*)
$\omega_1(a^{'})$	3201.34	3183.93	3183.93
$\omega_2(a^{'})$	3086.58	3067.86	3067.86
$\omega_3(a^{'})$	1916.58	1905.45	1905.62
$\omega_4(a^{'})$	1473.99	1477.97	1477.97
$\omega_5(a^{'})$	1405.92	1408.84	1408.72
$\omega_6(a^{'})$	1225.98	1230.21	1230.40
$\omega_7(a^{'})$	1013.61	1017.40	1017.14
$\omega_8(a^{'})$	857.45	855.16	855.01
$\omega_9(a^{'})$	601.39	605.11	605.11
$\omega_{10}(a^{'})$	409.83	412.99	412.99
$\omega_{11}(a^{''})$	3155.91	3141.37	3141.37
$\omega_{12}(a^{''})$	1480.10	1486.49	1486.50
$\omega_{13}(a^{''})$	1073.13	1073.12	1073.12
$\omega_{14}(a^{''})$	574.15	572.53	572.53
$\omega_{15}(a^{''})$	129.18	135.67	135.57

Table S144: Symmetrized, unnormalized natural internal coordinates for acetyl fluoride.

1	$r_{1,2}$
2	$r_{2,3}$
3	$r_{2,4}$
4	$r_{1,5} + r_{1,6} + r_{1,7}$
5	$2r_{1,5} - r_{1,6} - r_{1,7}$
6	$r_{1,6} - r_{1,7}$
7	$2\phi_{3,2,4} - \phi_{1,2,3} - \phi_{1,2,4}$
8	$\phi_{1,2,3} - \phi_{1,2,4}$
9	$\phi_{2,1,5} + \phi_{2,1,7} + \phi_{2,1,6} - \phi_{6,1,7} - \phi_{5,1,6} - \phi_{5,1,7}$
10	$2\phi_{2,1,5} - \phi_{2,1,7} - \phi_{2,1,6}$
11	$\phi_{2,1,7} - \phi_{2,1,6}$
12	$2\phi_{6,1,7} - \phi_{5,1,6} - \phi_{5,1,7}$
13	$\phi_{5,1,6} - \phi_{5,1,7}$
14	$\tau_{5,1,2,3} + \tau_{6,1,2,3} + \tau_{7,1,2,3} + \tau_{5,1,2,4} + \tau_{6,1,2,4} + \tau_{7,1,2,4}$
15	$\gamma_{4,2,3,1}$

S4.37 acetic acid

Table S145: CCSD(T)/cc-pVTZ Optimum Cartesian coordinates (bohr)

1	C	−2.75458669	−0.07584462	0.00000006
2	C	0.07515133	0.19163584	0.00000005
3	O	1.24072442	2.15236111	0.00000001
4	O	1.22880289	−2.09877025	−0.00000003
5	H	−3.61644252	1.78677173	0.00000005
6	H	−3.34756391	−1.13717476	−1.66377116
7	H	−3.34756393	−1.13717626	1.66377031
8	H	3.02195767	−1.74165265	−0.00000015

Table S146: Reference [CCSD(T)/cc-pVTZ] CMA-0A and CMA-2A (ξ = 0.02) harmonic frequencies for Level B = MP2/cc-pVTZ and CCSD(T)/cc-pVDZ.

	Reference CCSD(T)/ cc-pVTZ	Pure MP2/ cc-pVTZ	CMA-0A MP2/ cc-pVTZ	CMA-2A MP2/ cc-pVTZ	Pure CCSD(T)/ cc-pVDZ	CMA-0A CCSD(T)/ cc-pVDZ	CMA-2A CCSD(T)/ cc-pVDZ
$\omega_1(a^{'})$	3776.29	3786.74	3776.29	3776.29	3846.91	3776.27	3776.27
$\omega_2(a^{'})$	3180.57	3202.28	3180.54	3180.54	3329.67	3180.48	3180.48
$\omega_3(a^{'})$	3064.96	3077.09	3064.98	3064.98	3196.34	3064.99	3064.99
$\omega_4(a^{'})$	1837.11	1834.76	1837.05	1837.08	1896.78	1836.80	1836.91
$\omega_5(a^{'})$	1481.23	1482.91	1481.15	1481.15	1492.14	1481.10	1481.10
$\omega_6(a^{'})$	1422.27	1414.33	1422.33	1422.34	1450.47	1421.31	1422.28
$\omega_7(a^{'})$	1355.18	1345.92	1355.10	1355.18	1372.73	1355.81	1355.44
$\omega_8(a^{'})$	1220.34	1207.39	1220.48	1220.34	1249.10	1220.82	1220.41
$\omega_9(a^{'})$	1005.01	1001.34	1005.10	1005.09	1018.69	1005.70	1005.13
$\omega_{10}(a^{'})$	869.68	868.69	869.70	869.70	893.64	869.92	869.92
$\omega_{11}(a^{'})$	584.13	584.54	584.13	584.13	594.56	584.22	584.22
$\omega_{12}(a^{'})$	418.78	418.93	418.79	418.79	422.28	418.79	418.79
$\omega_{13}(a^{''})$	3137.39	3160.27	3137.39	3137.39	3286.99	3137.36	3137.36
$\omega_{14}(a^{''})$	1487.62	1490.51	1487.61	1487.61	1498.00	1487.62	1487.62
$\omega_{15}(a^{''})$	1071.35	1068.94	1071.36	1071.36	1080.95	1071.45	1071.45
$\omega_{16}(a^{''})$	664.47	669.23	664.36	664.47	679.91	664.35	664.49
$\omega_{17}(a^{''})$	545.73	550.36	545.87	545.73	551.78	545.90	545.73
$\omega_{18}(a^{''})$	78.18	74.64	78.24	78.24	90.34	78.28	78.23

Table S147: CMA-0A and CMA-2A (ξ = 0.02) harmonic frequencies targeting CCSD(T)/cc-pVTZ for Level B = B3LYP/6-31G(2df,p).

	Pure B3LYP/ 6-31G(2df,p)	CMA-0A B3LYP/ 6-31G(2df,p)	CMA-2A B3LYP/ 6-31G(2df,p)
$\omega_1(a^{'})$	3772.47	3776.28	3776.28
$\omega_2(a^{'})$	3198.23	3180.56	3180.56
$\omega_3(a^{'})$	3084.23	3064.92	3064.92
$\omega_4(a^{'})$	1839.38	1836.95	1837.05
$\omega_5(a^{'})$	1476.41	1480.97	1480.97
$\omega_6(a^{'})$	1416.17	1421.69	1422.28
$\omega_7(a^{'})$	1353.00	1355.94	1355.18
$\omega_8(a^{'})$	1212.72	1220.30	1220.37
$\omega_9(a^{'})$	1002.42	1005.35	1005.34
$\omega_{10}(a^{'})$	866.07	870.06	869.95
$\omega_{11}(a^{'})$	581.26	584.15	584.15
$\omega_{12}(a^{'})$	418.92	418.81	418.81
$\omega_{13}(a^{''})$	3152.31	3137.39	3137.39
$\omega_{14}(a^{''})$	1482.19	1487.58	1487.58
$\omega_{15}(a^{''})$	1070.43	1071.40	1071.40
$\omega_{16}(a^{''})$	676.14	664.44	664.48
$\omega_{17}(a^{''})$	547.06	545.77	545.72
$\omega_{18}(a^{''})$	83.46	78.47	78.43

Table S148: Symmetrized, unnormalized natural internal coordinates for acetic acid.

1	$r_{1,2}$
2	$r_{2,3}$
3	$r_{2,4}$
4	$r_{4,8}$
5	$r_{1,5} + r_{1,6} + r_{1,7}$
6	$2r_{1,5} - r_{1,6} - r_{1,7}$
7	$r_{1,6} - r_{1,7}$
8	$\phi_{1,2,3} - \phi_{3,2,4}$
9	$-\phi_{1,2,3} - \phi_{3,2,4} + 2\phi_{4,2,1}$
10	$\phi_{2,4,8}$
11	$\phi_{5,1,2} + \phi_{6,1,2} + \phi_{7,1,2} - \phi_{6,1,7} - \phi_{5,1,6} - \phi_{5,1,7}$
12	$2\phi_{5,1,2} - \phi_{6,1,2} - \phi_{7,1,2}$
13	$\phi_{6,1,2} - \phi_{7,1,2}$
14	$2\phi_{6,1,7} - \phi_{5,1,6} - \phi_{5,1,7}$
15	$\phi_{5,1,6} - \phi_{5,1,7}$
16	$\tau_{1,2,4,8} + \tau_{3,2,4,8}$
17	$\tau_{5,1,2,3} + \tau_{6,1,2,3} + \tau_{7,1,2,3} + \tau_{5,1,2,4} + \tau_{6,1,2,4} + \tau_{7,1,2,4}$
18	$\gamma_{3,2,1,4}$

S4.38 methyl formate

Table S149: CCSD(T)/cc-pVTZ Optimum Cartesian coordinates (bohr)

1	C	−2.56474627	1.03805801	−0.00000001
2	O	−1.13832898	−1.27719414	0.00000001
3	C	1.37333384	−0.93569129	−0.00000000
4	O	2.44023024	1.07500163	0.00000000
5	H	−2.12605991	2.14890436	−1.67647724
6	H	−4.53723889	0.47158333	0.00000002
7	H	−2.12605989	2.14890440	1.67647720
8	H	2.31318467	−2.77931310	−0.00000007

Table S150: Reference [CCSD(T)/cc-pVTZ] CMA-0A and CMA-2A ($\xi = 0.02$) harmonic frequencies for Level B = MP2/cc-pVTZ and CCSD(T)/cc-pVDZ.

	Reference CCSD(T)/ cc-pVTZ	Pure MP2/ cc-pVTZ	CMA-0A MP2/ cc-pVTZ	CMA-2A MP2/ cc-pVTZ	Pure CCSD(T)/ cc-pVDZ	CMA-0A CCSD(T)/ cc-pVDZ	CMA-2A CCSD(T)/ cc-pVDZ
$\omega_1(a^{'})$	3176.95	3200.92	3176.94	3176.94	3328.50	3176.89	3176.89
$\omega_2(a^{'})$	3078.26	3094.68	3078.25	3078.25	3219.59	3078.26	3078.26
$\omega_3(a^{'})$	3063.30	3075.57	3063.32	3063.32	3196.88	3063.31	3063.31
$\omega_4(a^{'})$	1799.60	1796.87	1799.54	1799.60	1860.41	1799.34	1799.54
$\omega_5(a^{'})$	1509.79	1514.42	1509.73	1509.73	1519.61	1509.46	1509.46
$\omega_6(a^{'})$	1472.50	1472.53	1472.54	1472.54	1491.50	1472.85	1472.85
$\omega_7(a^{'})$	1404.55	1405.23	1404.51	1404.44	1427.52	1404.44	1404.44
$\omega_8(a^{'})$	1243.84	1244.63	1243.95	1243.95	1268.72	1243.10	1243.98
$\omega_9(a^{'})$	1195.91	1192.66	1195.90	1195.89	1210.76	1197.15	1196.01
$\omega_{10}(a^{'})$	957.60	956.35	957.66	957.66	974.07	957.66	957.66
$\omega_{11}(a^{'})$	776.79	775.66	776.84	776.84	787.32	777.03	776.91
$\omega_{12}(a^{'})$	310.98	307.68	311.00	311.00	317.99	311.02	311.02
$\omega_{13}(a^{''})$	3143.88	3167.12	3143.88	3143.88	3294.96	3143.86	3143.86
$\omega_{14}(a^{''})$	1495.32	1501.53	1495.32	1495.32	1507.39	1495.36	1495.36
$\omega_{15}(a^{''})$	1185.12	1186.88	1185.11	1185.11	1196.02	1185.13	1185.13
$\omega_{16}(a^{''})$	1049.47	1051.92	1049.50	1049.50	1061.39	1049.47	1049.47
$\omega_{17}(a^{''})$	339.98	345.54	339.98	339.98	344.65	339.99	339.98
$\omega_{18}(a^{''})$	144.91	146.02	144.93	144.91	156.56	144.98	144.97

Table S151: CMA-0A and CMA-2A (ξ = 0.02) harmonic frequencies targeting CCSD(T)/cc-pVTZ for Level B = B3LYP/6-31G(2*df*,*p*).

	Pure B3LYP/ 6-31G(2*df*,*p*)	CMA-0A B3LYP/ 6-31G(2*df*,*p*)	CMA-2A B3LYP/ 6-31G(2*df*,*p*)
$\omega_1(a^{'})$	3197.71	3176.95	3176.95
$\omega_2(a^{'})$	3097.10	3078.13	3078.13
$\omega_3(a^{'})$	3086.15	3063.38	3063.38
$\omega_4(a^{'})$	1806.09	1799.50	1799.52
$\omega_5(a^{'})$	1501.32	1509.60	1509.60
$\omega_6(a^{'})$	1471.91	1472.67	1472.67
$\omega_7(a^{'})$	1398.93	1404.61	1404.58
$\omega_8(a^{'})$	1231.23	1242.77	1243.86
$\omega_9(a^{'})$	1185.72	1196.95	1195.99
$\omega_{10}(a^{'})$	948.56	957.99	957.76
$\omega_{11}(a^{'})$	775.81	776.83	776.83
$\omega_{12}(a^{'})$	314.58	311.04	311.04
$\omega_{13}(a^{''})$	3165.11	3143.88	3143.88
$\omega_{14}(a^{''})$	1490.19	1495.32	1495.32
$\omega_{15}(a^{''})$	1179.88	1185.09	1185.09
$\omega_{16}(a^{''})$	1047.10	1049.48	1049.48
$\omega_{17}(a^{''})$	341.67	339.99	339.98
$\omega_{18}(a^{''})$	143.24	145.13	145.09

Table S152: Symmetrized, unnormalized natural internal coordinates for methyl formate.

1	$r_{1,2}$
2	$r_{2,3}$
3	$r_{3,4}$
4	$r_{3,8}$
5	$r_{1,6} + r_{1,5} + r_{1,7}$
6	$2r_{1,6} - r_{1,5} - r_{1,7}$
7	$r_{1,5} - r_{1,7}$
8	$\phi_{1,2,3}$
9	$2\phi_{2,3,4} - \phi_{4,3,8} - \phi_{2,3,8}$
10	$\phi_{4,3,8} - \phi_{2,3,8}$
11	$\phi_{5,1,7} + \phi_{6,1,5} + \phi_{6,1,7} - \phi_{6,1,2} - \phi_{5,1,2} - \phi_{7,1,2}$
12	$2\phi_{5,1,7} - \phi_{6,1,5} - \phi_{6,1,7}$
13	$\phi_{6,1,5} - \phi_{6,1,7}$
14	$2\phi_{6,1,2} - \phi_{5,1,2} - \phi_{7,1,2}$
15	$\phi_{5,1,2} - \phi_{7,1,2}$
16	$\tau_{5,1,2,3} + \tau_{6,1,2,3} + \tau_{7,1,2,3}$
17	$\tau_{1,2,3,4} + \tau_{1,2,3,8}$
18	$\gamma_{8,3,2,4}$

S4.39 acetaldehyde

Table S153: CCSD(T)/cc-pVTZ Optimum Cartesian coordinates (bohr)

1	C	−2.38336780	0.31344831	−0.00000000
2	C	0.23682939	−0.79607621	0.00000000
3	O	2.16461069	0.43923199	−0.00000000
4	H	0.32309962	−2.88723239	0.00000000
5	H	−2.29051382	2.36809272	−0.00000000
6	H	−3.41403157	−0.35261154	1.66196703
7	H	−3.41403157	−0.35261154	−1.66196703

Table S154: Reference [CCSD(T)/cc-pVTZ] CMA-0A and CMA-2A (ξ = 0.02) harmonic frequencies for Level B = MP2/cc-pVTZ and CCSD(T)/cc-pVDZ.

	Reference CCSD(T)/ cc-pVTZ	Pure MP2/ cc-pVTZ	CMA-0A MP2/ cc-pVTZ	CMA-2A MP2/ cc-pVTZ	Pure CCSD(T)/ cc-pVDZ	CMA-0A CCSD(T)/ cc-pVDZ	CMA-2A CCSD(T)/ cc-pVDZ
$\omega_1(a^{'})$	3156.22	3178.16	3156.18	3156.18	3307.38	3156.15	3156.15
$\omega_2(a^{'})$	3037.72	3050.22	3037.74	3037.74	3169.36	3037.69	3037.69
$\omega_3(a^{'})$	2918.88	2935.71	2918.89	2918.89	3058.61	2918.90	2918.90
$\omega_4(a^{'})$	1793.14	1785.54	1793.10	1793.12	1845.78	1793.00	1793.00
$\omega_5(a^{'})$	1469.93	1471.25	1469.87	1469.87	1481.19	1469.89	1469.89
$\omega_6(a^{'})$	1431.32	1433.08	1431.24	1431.21	1454.51	1431.31	1431.31
$\omega_7(a^{'})$	1383.16	1376.13	1383.33	1383.33	1400.04	1383.06	1383.06
$\omega_8(a^{'})$	1134.44	1132.22	1134.47	1134.48	1149.46	1134.39	1134.78
$\omega_9(a^{'})$	895.54	892.85	895.58	895.58	921.21	896.33	895.84
$\omega_{10}(a^{'})$	503.56	504.01	503.57	503.56	509.29	503.59	503.57
$\omega_{11}(a^{''})$	3105.97	3130.03	3105.96	3105.96	3254.84	3105.92	3105.92
$\omega_{12}(a^{''})$	1481.19	1483.82	1481.18	1481.18	1493.93	1481.19	1481.19
$\omega_{13}(a^{''})$	1132.41	1134.44	1132.34	1132.34	1143.94	1132.49	1132.49
$\omega_{14}(a^{''})$	777.41	776.76	777.53	777.54	790.18	777.45	777.45
$\omega_{15}(a^{''})$	156.12	156.62	156.22	156.15	168.38	156.20	156.19

Table S155: CMA-0A and CMA-2A (ξ = 0.02) harmonic frequencies targeting CCSD(T)/cc-pVTZ for Level B = B3LYP/6-31G(2*df*,*p*).

	Pure B3LYP/ 6-31G(2*df*,*p*)	CMA-0A B3LYP/ 6-31G(2*df*,*p*)	CMA-2A B3LYP/ 6-31G(2*df*,*p*)
$\omega_1(a^{'})$	3174.37	3156.20	3156.20
$\omega_2(a^{'})$	3056.92	3037.67	3037.67
$\omega_3(a^{'})$	2932.94	2918.85	2918.85
$\omega_4(a^{'})$	1803.85	1792.94	1793.07
$\omega_5(a^{'})$	1466.35	1469.40	1469.40
$\omega_6(a^{'})$	1429.03	1431.47	1431.42
$\omega_7(a^{'})$	1382.20	1383.48	1383.48
$\omega_8(a^{'})$	1133.20	1134.53	1134.54
$\omega_9(a^{'})$	890.38	896.24	896.07
$\omega_{10}(a^{'})$	504.87	503.61	503.58
$\omega_{11}(a^{''})$	3120.06	3105.97	3105.97
$\omega_{12}(a^{''})$	1477.29	1481.10	1481.11
$\omega_{13}(a^{''})$	1138.14	1132.23	1132.26
$\omega_{14}(a^{''})$	777.65	777.78	777.78
$\omega_{15}(a^{''})$	163.78	156.45	156.12

Table S156: Symmetrized, unnormalized natural internal coordinates for acetaldehyde.

1	$r_{1,2}$
2	$r_{2,3}$
3	$r_{2,4}$
4	$r_{1,5} + r_{1,6} + r_{1,7}$
5	$2r_{1,5} - r_{1,6} - r_{1,7}$
6	$r_{1,6} - r_{1,7}$
7	$2\phi_{3,2,4} - \phi_{1,2,3} - \phi_{1,2,4}$
8	$\phi_{1,2,3} - \phi_{1,2,4}$
9	$\phi_{2,1,5} + \phi_{2,1,7} + \phi_{2,1,6} - \phi_{6,1,7} - \phi_{5,1,6} - \phi_{5,1,7}$
10	$2\phi_{2,1,5} - \phi_{2,1,7} - \phi_{2,1,6}$
11	$\phi_{2,1,7} - \phi_{2,1,6}$
12	$2\phi_{6,1,7} - \phi_{5,1,6} - \phi_{5,1,7}$
13	$\phi_{5,1,6} - \phi_{5,1,7}$
14	$\tau_{5,1,2,3} + \tau_{6,1,2,3} + \tau_{7,1,2,3} + \tau_{5,1,2,4} + \tau_{6,1,2,4} + \tau_{7,1,2,4}$
15	$\gamma_{4,2,3,1}$

S4.40 ethyl chloride

Table S157: CCSD(T)/cc-pVTZ Optimum Cartesian coordinates (bohr)

1	C	0.71138750	−3.24270819	0.00000000
2	C	−1.25342727	−1.15447677	0.00000000
3	H	1.90901920	−3.11721977	1.67181496
4	H	−0.23045169	−5.08087833	0.00000000
5	H	1.90901920	−3.11721977	−1.67181496
6	H	−2.44503705	−1.22529373	1.67390274
7	H	−2.44503705	−1.22529373	−1.67390274
8	CL	0.22354627	1.90569145	0.00000000

Table S158: Reference [CCSD(T)/cc-pVTZ] CMA-0A and CMA-2A ($\xi = 0.02$) harmonic frequencies for Level B = MP2/cc-pVTZ and CCSD(T)/cc-pVDZ.

	Reference CCSD(T)/ cc-pVTZ	Pure MP2/ cc-pVTZ	CMA-0A MP2/ cc-pVTZ	CMA-2A MP2/ cc-pVTZ	Pure CCSD(T)/ cc-pVDZ	CMA-0A CCSD(T)/ cc-pVDZ	CMA-2A CCSD(T)/ cc-pVDZ
$\omega_1(a^{'})$	3119.73	3143.57	3119.61	3119.61	3270.53	3119.45	3119.45
$\omega_2(a^{'})$	3091.33	3107.33	3091.40	3091.40	3232.04	3091.51	3091.51
$\omega_3(a^{'})$	3042.96	3055.67	3043.02	3043.02	3177.06	3042.99	3042.99
$\omega_4(a^{'})$	1507.53	1510.16	1507.49	1507.49	1520.26	1507.50	1507.50
$\omega_5(a^{'})$	1498.23	1499.97	1498.21	1498.21	1514.51	1498.14	1498.14
$\omega_6(a^{'})$	1414.26	1406.69	1414.27	1414.27	1434.47	1413.97	1413.97
$\omega_7(a^{'})$	1321.71	1319.41	1321.72	1321.72	1344.67	1321.86	1321.86
$\omega_8(a^{'})$	1094.10	1092.85	1094.13	1094.13	1118.18	1093.69	1094.51
$\omega_9(a^{'})$	993.24	992.01	993.26	993.28	1017.35	994.34	993.44
$\omega_{10}(a^{'})$	688.50	696.31	688.55	688.52	699.43	688.55	688.54
$\omega_{11}(a^{'})$	331.55	330.59	331.56	331.56	339.66	331.56	331.56
$\omega_{12}(a^{''})$	3154.40	3177.65	3154.40	3154.40	3306.32	3154.38	3154.38
$\omega_{13}(a^{''})$	3127.71	3152.17	3127.71	3127.71	3282.50	3127.69	3127.69
$\omega_{14}(a^{''})$	1492.92	1495.18	1492.91	1492.91	1505.40	1492.95	1492.95
$\omega_{15}(a^{''})$	1280.41	1281.33	1280.35	1280.35	1294.17	1280.41	1280.41
$\omega_{16}(a^{''})$	1080.78	1081.98	1080.87	1080.87	1096.39	1080.81	1080.81
$\omega_{17}(a^{''})$	787.66	788.60	787.67	787.67	800.30	787.70	787.71
$\omega_{18}(a^{''})$	261.83	265.98	261.85	261.84	280.16	261.88	261.85

Table S159: CMA-0A and CMA-2A (ξ = 0.02) harmonic frequencies targeting CCSD(T)/cc-pVTZ for Level B = B3LYP/6-31G(2*df*,*p*).

	Pure B3LYP/ 6-31G(2*df*,*p*)	CMA-0A B3LYP/ 6-31G(2*df*,*p*)	CMA-2A B3LYP/ 6-31G(2*df*,*p*)
$\omega_1(a^{'})$	3136.21	3119.67	3119.67
$\omega_2(a^{'})$	3107.47	3091.33	3091.33
$\omega_3(a^{'})$	3062.56	3043.00	3043.00
$\omega_4(a^{'})$	1507.81	1507.46	1507.46
$\omega_5(a^{'})$	1494.60	1498.22	1498.22
$\omega_6(a^{'})$	1420.85	1414.04	1414.04
$\omega_7(a^{'})$	1322.18	1321.70	1321.70
$\omega_8(a^{'})$	1093.19	1093.73	1093.73
$\omega_9(a^{'})$	987.87	994.06	994.06
$\omega_{10}(a^{'})$	685.91	688.71	688.71
$\omega_{11}(a^{'})$	336.58	331.59	331.59
$\omega_{12}(a^{''})$	3172.92	3154.38	3154.38
$\omega_{13}(a^{''})$	3144.87	3127.71	3127.71
$\omega_{14}(a^{''})$	1490.94	1492.91	1492.91
$\omega_{15}(a^{''})$	1279.83	1280.29	1280.29
$\omega_{16}(a^{''})$	1080.31	1080.93	1080.93
$\omega_{17}(a^{''})$	793.22	787.74	787.74
$\omega_{18}(a^{''})$	259.16	261.85	261.85

Table S160: Symmetrized, unnormalized natural internal coordinates for ethyl chloride.

1	$r_{1,2}$
2	$r_{2,8}$
3	$r_{1,4} + r_{1,3} + r_{1,5}$
4	$2r_{1,4} - r_{1,3} - r_{1,5}$
5	$r_{1,3} - r_{1,5}$
6	$r_{2,6} + r_{2,7}$
7	$r_{2,6} - r_{2,7}$
8	$\phi_{1,2,8}$
9	$\phi_{6,2,1} + \phi_{6,2,8} - \phi_{7,2,1} - \phi_{7,2,8}$
10	$\phi_{6,2,1} - \phi_{6,2,8} + \phi_{7,2,1} - \phi_{7,2,8}$
11	$\phi_{6,2,1} - \phi_{6,2,8} - \phi_{7,2,1} + \phi_{7,2,8}$
12	$-\phi_{6,2,1} - \phi_{6,2,8} - \phi_{7,2,1} - \phi_{7,2,8} + 4\phi_{6,2,7}$
13	$\phi_{4,1,2} + \phi_{3,1,2} + \phi_{5,1,2} - \phi_{3,1,5} - \phi_{4,1,3} - \phi_{4,1,5}$
14	$2\phi_{4,1,2} - \phi_{3,1,2} - \phi_{5,1,2}$
15	$\phi_{3,1,2} - \phi_{5,1,2}$
16	$2\phi_{3,1,5} - \phi_{4,1,3} - \phi_{4,1,5}$
17	$\phi_{4,1,3} - \phi_{4,1,5}$
18	$\tau_{3,1,2,8} + \tau_{4,1,2,8} + \tau_{5,1,2,8}$

S4.41 ethane

Table S161: CCSD(T)/cc-pVTZ Optimum Cartesian coordinates (bohr)

1	C	−1.44464665	−0.00000002	0.00000001
2	C	1.44464665	0.00000008	−0.00000008
3	H	−2.19072560	−1.85534039	0.50798051
4	H	−2.19072568	1.36759417	1.35278165
5	H	−2.19072574	0.48774615	−1.86076207
6	H	2.19072576	−0.48774633	1.86076194
7	H	2.19072558	1.85534053	−0.50798031
8	H	2.19072568	−1.36759487	−1.35278094

Table S162: Reference [CCSD(T)/cc-pVTZ] CMA-0A and CMA-2A ($\xi = 0.02$) harmonic frequencies for Level B = MP2/cc-pVTZ and CCSD(T)/cc-pVDZ.

	Reference CCSD(T)/ cc-pVTZ	Pure MP2/ cc-pVTZ	CMA-0A MP2/ cc-pVTZ	CMA-2A MP2/ cc-pVTZ	Pure CCSD(T)/ cc-pVDZ	CMA-0A CCSD(T)/ cc-pVDZ	CMA-2A CCSD(T)/ cc-pVDZ
$\omega_1(a_{1g})$	3039.53	3051.30	3039.53	3039.53	3177.06	3039.48	3039.48
$\omega_2(a_{1g})$	1427.46	1421.34	1427.46	1427.46	1452.19	1427.28	1427.28
$\omega_3(a_{1g})$	1013.93	1015.05	1013.95	1013.95	1045.36	1014.33	1014.33
$\omega_{4a}(e_g)$	3096.88	3123.50	3096.88	3096.88	3252.34	3096.85	3096.85
$\omega_{4b}(e_g)$	3096.88	3123.44	3096.88	3096.88	3252.29	3096.85	3096.85
$\omega_{5a}(e_g)$	1510.83	1515.62	1510.81	1510.81	1522.33	1510.89	1510.89
$\omega_{5b}(e_g)$	1510.83	1515.61	1510.81	1510.81	1522.32	1510.89	1510.89
$\omega_{6a}(e_g)$	1224.79	1222.45	1224.82	1224.82	1237.99	1224.81	1224.81
$\omega_{6b}(e_g)$	1224.79	1222.45	1224.82	1224.82	1237.99	1224.81	1224.81
$\omega_7(a_{1u})$	310.01	314.19	310.01	310.01	331.48	310.01	310.01
$\omega_8(a_{2u})$	3038.00	3052.91	3038.00	3038.00	3171.45	3037.99	3037.99
$\omega_9(a_{2u})$	1406.53	1402.31	1406.53	1406.53	1421.61	1406.56	1406.56
$\omega_{10a}(e_u)$	3120.06	3145.60	3120.06	3120.06	3272.82	3120.03	3120.03
$\omega_{10b}(e_u)$	3120.06	3145.54	3120.06	3120.06	3272.76	3120.03	3120.03
$\omega_{11a}(e_u)$	1512.52	1515.39	1512.52	1512.52	1524.60	1512.57	1512.57
$\omega_{11b}(e_u)$	1512.52	1515.38	1512.52	1512.52	1524.58	1512.57	1512.57
$\omega_{12a}(e_u)$	820.85	821.77	820.87	820.86	832.79	820.90	820.90
$\omega_{12b}(e_u)$	820.85	821.75	820.87	820.86	832.77	820.90	820.90

Table S163: CMA-0A and CMA-2A (ξ = 0.02) harmonic frequencies targeting CCSD(T)/cc-pVTZ for Level B = B3LYP/6-31G(2*df*,*p*).

	Pure B3LYP/ 6-31G(2*df*,*p*)	CMA-0A B3LYP/ 6-31G(2*df*,*p*)	CMA-2A B3LYP/ 6-31G(2*df*,*p*)
$\omega_1(a_{1g})$	3059.32	3039.50	3039.50
$\omega_2(a_{1g})$	1430.23	1427.34	1427.34
$\omega_3(a_{1g})$	1008.47	1014.20	1014.20
$\omega_{4a}(e_g)$	3112.60	3096.88	3096.88
$\omega_{4b}(e_g)$	3112.52	3096.88	3096.88
$\omega_{5a}(e_g)$	1508.97	1510.81	1510.81
$\omega_{5b}(e_g)$	1508.84	1510.81	1510.81
$\omega_{6a}(e_g)$	1230.68	1224.82	1224.82
$\omega_{6b}(e_g)$	1230.64	1224.82	1224.82
$\omega_7(a_{1u})$	308.52	310.01	310.01
$\omega_8(a_{2u})$	3059.86	3038.00	3038.00
$\omega_9(a_{2u})$	1418.10	1406.53	1406.53
$\omega_{10a}(e_u)$	3137.77	3120.06	3120.06
$\omega_{10b}(e_u)$	3137.68	3120.06	3120.06
$\omega_{11a}(e_u)$	1514.69	1512.50	1512.50
$\omega_{11b}(e_u)$	1514.62	1512.50	1512.50
$\omega_{12a}(e_u)$	827.77	820.91	820.91
$\omega_{12b}(e_u)$	827.73	820.91	820.91

Table S164: Symmetrized, unnormalized natural internal coordinates for ethane.

1	$r_{1,2}$
2	$r_{1,3} + r_{1,4} + r_{1,5} + r_{2,7} + r_{2,8} + r_{2,6}$
3	$r_{1,3} + r_{1,4} + r_{1,5} - r_{2,7} - r_{2,8} - r_{2,6}$
4	$2r_{1,3} - r_{1,4} - r_{1,5} + 2r_{2,7} - r_{2,8} - r_{2,6}$
5	$r_{1,4} - r_{1,5} + r_{2,8} - r_{2,6}$
6	$2r_{1,3} - r_{1,4} - r_{1,5} - 2r_{2,7} + r_{2,8} + r_{2,6}$
7	$r_{1,4} - r_{1,5} - r_{2,8} + r_{2,6}$
8	$\phi_{4,1,5} + \phi_{3,1,4} + \phi_{3,1,5} - \phi_{3,1,2} - \phi_{4,1,2} - \phi_{5,1,2} + \phi_{6,2,8} + \phi_{7,2,8} + \phi_{7,2,6} - \phi_{7,2,1}$ $-\phi_{8,2,1} - \phi_{6,2,1}$
9	$\phi_{4,1,5} + \phi_{3,1,4} + \phi_{3,1,5} - \phi_{3,1,2} - \phi_{4,1,2} - \phi_{5,1,2} - \phi_{6,2,8} - \phi_{7,2,8} - \phi_{7,2,6} + \phi_{7,2,1}$ $+\phi_{8,2,1} + \phi_{6,2,1}$
10	$2\phi_{4,1,5} - \phi_{3,1,4} - \phi_{3,1,5} + 2\phi_{6,2,8} - \phi_{7,2,8} - \phi_{7,2,6}$
11	$\phi_{3,1,4} - \phi_{3,1,5} + \phi_{7,2,8} - \phi_{7,2,6}$
12	$2\phi_{4,1,5} - \phi_{3,1,4} - \phi_{3,1,5} - 2\phi_{6,2,8} + \phi_{7,2,8} + \phi_{7,2,6}$
13	$\phi_{3,1,4} - \phi_{3,1,5} - \phi_{7,2,8} + \phi_{7,2,6}$
14	$2\phi_{3,1,2} - \phi_{4,1,2} - \phi_{5,1,2} + 2\phi_{7,2,1} - \phi_{8,2,1} - \phi_{6,2,1}$
15	$\phi_{4,1,2} - \phi_{5,1,2} + \phi_{8,2,1} - \phi_{6,2,1}$
16	$2\phi_{3,1,2} - \phi_{4,1,2} - \phi_{5,1,2} - 2\phi_{7,2,1} + \phi_{8,2,1} + \phi_{6,2,1}$
17	$\phi_{4,1,2} - \phi_{5,1,2} - \phi_{8,2,1} + \phi_{6,2,1}$
18	$\tau_{3,1,2,7} + \tau_{7,2,1,3} + \tau_{4,1,2,8} + \tau_{8,2,1,4} + \tau_{5,1,2,6} + \tau_{6,2,1,5}$

S4.42 dimethyl ether

Table S165: CCSD(T)/cc-pVTZ Optimum Cartesian coordinates (bohr)

1	C	−2.19409540	0.47534797	−0.00000000
2	O	0.00000000	−1.03943917	0.00000000
3	C	2.19409540	0.47534797	−0.00000000
4	H	−3.82041225	−0.78516753	0.00000000
5	H	−2.27639290	1.68680381	−1.68214609
6	H	−2.27639290	1.68680381	1.68214609
7	H	2.27639289	1.68680383	1.68214608
8	H	2.27639289	1.68680383	−1.68214608
9	H	3.82041225	−0.78516754	0.00000000

Table S166: Reference [CCSD(T)/cc-pVTZ] CMA-0A and CMA-2A ($\xi = 0.02$) harmonic frequencies for Level B = MP2/cc-pVTZ and CCSD(T)/cc-pVDZ.

	Reference CCSD(T)/ cc-pVTZ	Pure MP2/ cc-pVTZ	CMA-0A MP2/ cc-pVTZ	CMA-2A MP2/ cc-pVTZ	Pure CCSD(T)/ cc-pVDZ	CMA-0A CCSD(T)/ cc-pVDZ	CMA-2A CCSD(T)/ cc-pVDZ
$\omega_1(a_1)$	3130.94	3154.26	3130.85	3130.85	3280.33	3130.83	3130.83
$\omega_2(a_1)$	2987.43	2999.99	2987.53	2987.53	3120.19	2987.46	2987.46
$\omega_3(a_1)$	1526.82	1530.64	1526.66	1526.66	1541.94	1525.21	1525.21
$\omega_4(a_1)$	1494.98	1494.20	1495.11	1495.11	1512.74	1496.67	1496.67
$\omega_5(a_1)$	1277.41	1272.63	1277.38	1277.38	1290.69	1277.43	1277.44
$\omega_6(a_1)$	964.06	961.48	964.15	964.15	981.28	964.20	964.20
$\omega_7(a_1)$	418.84	414.76	418.85	418.85	427.22	418.91	418.88
$\omega_8(a_2)$	3036.25	3062.58	3036.25	3036.25	3182.66	3036.21	3036.21
$\omega_9(a_2)$	1490.45	1495.82	1490.45	1490.45	1502.18	1490.52	1490.52
$\omega_{10}(a_2)$	1169.37	1171.81	1169.38	1169.38	1181.68	1169.40	1169.40
$\omega_{11}(a_2)$	203.09	207.76	203.10	203.09	213.42	203.10	203.10
$\omega_{12}(b_1)$	3030.84	3055.53	3030.84	3030.84	3177.66	3030.81	3030.81
$\omega_{13}(b_1)$	1500.07	1505.72	1500.07	1500.07	1510.53	1500.12	1500.12
$\omega_{14}(b_1)$	1202.41	1202.56	1202.42	1202.42	1216.12	1202.44	1202.44
$\omega_{15}(b_1)$	255.06	258.31	255.06	255.06	265.55	255.06	255.06
$\omega_{16}(b_2)$	3129.14	3153.26	3129.06	3129.06	3278.65	3129.01	3129.01
$\omega_{17}(b_2)$	2978.19	2992.86	2978.28	2978.28	3108.59	2978.26	2978.26
$\omega_{18}(b_2)$	1508.70	1513.11	1508.68	1508.68	1519.95	1508.73	1508.73
$\omega_{19}(b_2)$	1460.11	1459.71	1460.08	1460.08	1481.44	1460.05	1460.05
$\omega_{20}(b_2)$	1212.11	1211.19	1212.15	1212.15	1229.81	1211.99	1212.27
$\omega_{21}(b_2)$	1128.79	1130.95	1128.83	1128.83	1145.47	1129.14	1128.83

Table S167: CMA-0A and CMA-2A (ξ = 0.02) harmonic frequencies targeting CCSD(T)/cc-pVTZ for Level B = B3LYP/6-31G(2df,p).

	Pure B3LYP/ 6-31G(2df,p)	CMA-0A B3LYP/ 6-31G(2df,p)	CMA-2A B3LYP/ 6-31G(2df,p)
$\omega_1(a_1)$	3151.06	3130.93	3130.93
$\omega_2(a_1)$	3009.08	2987.41	2987.41
$\omega_3(a_1)$	1524.24	1526.40	1526.40
$\omega_4(a_1)$	1497.08	1495.38	1495.38
$\omega_5(a_1)$	1274.80	1277.41	1277.41
$\omega_6(a_1)$	955.42	964.16	964.16
$\omega_7(a_1)$	427.26	418.90	418.89
$\omega_8(a_2)$	3051.50	3036.24	3036.24
$\omega_9(a_2)$	1490.47	1490.40	1490.40
$\omega_{10}(a_2)$	1169.58	1169.43	1169.43
$\omega_{11}(a_2)$	216.73	203.32	203.32
$\omega_{12}(b_1)$	3046.86	3030.84	3030.84
$\omega_{13}(b_1)$	1500.41	1500.05	1500.05
$\omega_{14}(b_1)$	1200.87	1202.43	1202.45
$\omega_{15}(b_1)$	257.39	255.15	255.08
$\omega_{16}(b_2)$	3149.23	3129.13	3129.13
$\omega_{17}(b_2)$	2996.13	2978.14	2978.14
$\omega_{18}(b_2)$	1504.83	1508.52	1508.52
$\omega_{19}(b_2)$	1463.53	1460.15	1460.15
$\omega_{20}(b_2)$	1194.51	1211.07	1212.35
$\omega_{21}(b_2)$	1126.39	1130.27	1128.90

Table S168: Symmetrized, unnormalized natural internal coordinates for dimethyl ether.

1	$r_{1,2} + r_{2,3}$
2	$r_{1,2} - r_{2,3}$
3	$r_{1,4} + r_{1,5} + r_{1,6} + r_{3,9} + r_{3,7} + r_{3,8}$
4	$r_{1,4} + r_{1,5} + r_{1,6} - r_{3,9} - r_{3,7} - r_{3,8}$
5	$2r_{1,4} - r_{1,5} - r_{1,6} + 2r_{3,9} - r_{3,7} - r_{3,8}$
6	$2r_{1,4} - r_{1,5} - r_{1,6} - 2r_{3,9} + r_{3,7} + r_{3,8}$
7	$r_{1,5} - r_{1,6} + r_{3,7} - r_{3,8}$
8	$r_{1,5} - r_{1,6} - r_{3,7} + r_{3,8}$
9	$\phi_{1,2,3}$
10	$\phi_{4,1,2} + \phi_{5,1,2} + \phi_{6,1,2} - \phi_{5,1,6} - \phi_{4,1,5} - \phi_{4,1,6} + \phi_{9,3,2} + \phi_{7,3,2} + \phi_{8,3,2} - \phi_{7,3,8} - \phi_{9,3,7} - \phi_{9,3,8}$
11	$\phi_{4,1,2} + \phi_{5,1,2} + \phi_{6,1,2} - \phi_{5,1,6} - \phi_{4,1,5} - \phi_{4,1,6} - \phi_{9,3,2} - \phi_{7,3,2} - \phi_{8,3,2} + \phi_{7,3,8} + \phi_{9,3,7} + \phi_{9,3,8}$
12	$2\phi_{4,1,2} - \phi_{5,1,2} - \phi_{6,1,2} + 2\phi_{9,3,2} - \phi_{7,3,2} - \phi_{8,3,2}$
13	$2\phi_{4,1,2} - \phi_{5,1,2} - \phi_{6,1,2} - 2\phi_{9,3,2} + \phi_{7,3,2} + \phi_{8,3,2}$
14	$\phi_{5,1,2} - \phi_{6,1,2} + \phi_{7,3,2} - \phi_{8,3,2}$
15	$\phi_{5,1,2} - \phi_{6,1,2} - \phi_{7,3,2} + \phi_{8,3,2}$
16	$2\phi_{5,1,6} - \phi_{4,1,5} - \phi_{4,1,6} + 2\phi_{7,3,8} - \phi_{9,3,7} - \phi_{9,3,8}$
17	$2\phi_{5,1,6} - \phi_{4,1,5} - \phi_{4,1,6} - 2\phi_{7,3,8} + \phi_{9,3,7} + \phi_{9,3,8}$
18	$\phi_{4,1,5} - \phi_{4,1,6} + \phi_{9,3,7} - \phi_{9,3,8}$
19	$\phi_{4,1,5} - \phi_{4,1,6} - \phi_{9,3,7} + \phi_{9,3,8}$
20	$\tau_{4,1,2,3} + \tau_{5,1,2,3} + \tau_{6,1,2,3} + \tau_{7,3,2,1} + \tau_{8,3,2,1} + \tau_{9,3,2,1}$
21	$\tau_{4,1,2,3} + \tau_{5,1,2,3} + \tau_{6,1,2,3} - \tau_{7,3,2,1} - \tau_{8,3,2,1} - \tau_{9,3,2,1}$

S4.43 ethanol

Table S169: CCSD(T)/cc-pVTZ Optimum Cartesian coordinates (bohr)

1	C	−2.38160558	0.47396349	0.00000002
2	C	0.01654024	−1.09258611	−0.00000002
3	H	−2.44560526	1.67800747	1.67220551
4	H	−2.44560533	1.67800746	−1.67220550
5	H	−4.04445451	−0.74741713	0.00000006
6	H	0.06559877	−2.31232195	1.67426817
7	H	0.06559875	−2.31232193	−1.67426821
8	O	2.10079664	0.61455797	−0.00000002
9	H	3.62372825	−0.37160124	0.00000035

Table S170: Reference [CCSD(T)/cc-pVTZ] CMA-0A and CMA-2A ($\xi = 0.02$) harmonic frequencies for Level B = MP2/cc-pVTZ and CCSD(T)/cc-pVDZ.

	Reference CCSD(T)/ cc-pVTZ	Pure MP2/ cc-pVTZ	CMA-0A MP2/ cc-pVTZ	CMA-2A MP2/ cc-pVTZ	Pure CCSD(T)/ cc-pVDZ	CMA-0A CCSD(T)/ cc-pVDZ	CMA-2A CCSD(T)/ cc-pVDZ
$\omega_1(a^{'})$	3857.30	3874.39	3857.30	3857.30	3923.64	3857.29	3857.29
$\omega_2(a^{'})$	3123.33	3148.58	3123.32	3123.32	3273.46	3123.26	3123.26
$\omega_3(a^{'})$	3045.11	3057.80	3045.08	3045.08	3178.80	3045.03	3045.03
$\omega_4(a^{'})$	3000.32	3016.78	3000.36	3000.36	3131.80	3000.39	3000.39
$\omega_5(a^{'})$	1538.40	1541.11	1538.39	1538.39	1553.27	1538.27	1538.27
$\omega_6(a^{'})$	1508.41	1511.48	1508.33	1508.33	1523.03	1508.08	1508.08
$\omega_7(a^{'})$	1468.04	1460.91	1467.98	1467.98	1495.35	1468.12	1468.12
$\omega_8(a^{'})$	1403.22	1397.49	1403.22	1403.22	1419.47	1403.35	1403.35
$\omega_9(a^{'})$	1285.13	1276.06	1285.05	1285.16	1301.11	1285.01	1285.16
$\omega_{10}(a^{'})$	1121.48	1120.55	1121.59	1121.48	1139.37	1120.55	1120.42
$\omega_{11}(a^{'})$	1050.78	1047.14	1050.93	1050.95	1073.87	1052.19	1052.34
$\omega_{12}(a^{'})$	909.14	908.42	909.19	909.16	927.06	909.56	909.35
$\omega_{13}(a^{'})$	413.74	412.36	413.75	413.75	415.61	413.78	413.78
$\omega_{14}(a^{''})$	3128.35	3153.31	3128.35	3128.35	3281.05	3128.26	3128.26
$\omega_{15}(a^{''})$	3032.33	3058.83	3032.33	3032.33	3175.97	3032.39	3032.39
$\omega_{16}(a^{''})$	1489.67	1492.61	1489.65	1489.65	1501.15	1489.66	1489.66
$\omega_{17}(a^{''})$	1306.08	1308.61	1306.06	1306.06	1320.51	1306.07	1306.07
$\omega_{18}(a^{''})$	1187.04	1185.89	1187.09	1187.09	1202.02	1187.10	1187.10
$\omega_{19}(a^{''})$	818.70	819.61	818.71	818.71	832.11	818.68	818.69
$\omega_{20}(a^{''})$	280.63	285.48	280.63	280.64	296.69	280.72	280.89
$\omega_{21}(a^{''})$	235.41	239.14	235.45	235.42	255.16	235.70	235.46

Table S171: CMA-0A and CMA-2A (ξ = 0.02) harmonic frequencies targeting CCSD(T)/cc-pVTZ for Level B = B3LYP/6-31G(2*df*,*p*).

	Pure B3LYP/ 6-31G(2*df*,*p*)	CMA-0A B3LYP/ 6-31G(2*df*,*p*)	CMA-2A B3LYP/ 6-31G(2*df*,*p*)
$\omega_1(a^{'})$	3855.85	3857.30	3857.30
$\omega_2(a^{'})$	3138.73	3123.32	3123.32
$\omega_3(a^{'})$	3065.12	3045.09	3045.09
$\omega_4(a^{'})$	3011.85	3000.29	3000.29
$\omega_5(a^{'})$	1534.37	1538.09	1538.09
$\omega_6(a^{'})$	1508.15	1508.48	1508.48
$\omega_7(a^{'})$	1465.45	1467.95	1467.95
$\omega_8(a^{'})$	1411.27	1403.28	1403.28
$\omega_9(a^{'})$	1282.14	1285.00	1285.04
$\omega_{10}(a^{'})$	1117.60	1120.50	1120.60
$\omega_{11}(a^{'})$	1039.70	1052.28	1052.29
$\omega_{12}(a^{'})$	907.47	909.44	909.26
$\omega_{13}(a^{'})$	415.02	413.80	413.80
$\omega_{14}(a^{''})$	3144.96	3128.35	3128.35
$\omega_{15}(a^{''})$	3040.95	3032.33	3032.33
$\omega_{16}(a^{''})$	1490.46	1489.66	1489.66
$\omega_{17}(a^{''})$	1302.88	1305.43	1305.43
$\omega_{18}(a^{''})$	1185.30	1187.71	1187.71
$\omega_{19}(a^{''})$	825.02	818.79	818.79
$\omega_{20}(a^{''})$	276.15	280.54	280.64
$\omega_{21}(a^{''})$	242.11	235.60	235.45

Table S172: Symmetrized, unnormalized natural internal coordinates for ethanol.

1	$r_{1,2}$
2	$r_{2,8}$
3	$r_{8,9}$
4	$r_{1,5} + r_{1,3} + r_{1,4}$
5	$2r_{1,5} - r_{1,3} - r_{1,4}$
6	$r_{1,3} - r_{1,4}$
7	$r_{2,6} + r_{2,7}$
8	$r_{2,6} - r_{2,7}$
9	$\phi_{1,2,8}$
10	$\phi_{2,8,9}$
11	$\phi_{5,1,2} + \phi_{3,1,2} + \phi_{4,1,2} - \phi_{3,1,4} - \phi_{5,1,3} - \phi_{5,1,4}$
12	$2\phi_{5,1,2} - \phi_{3,1,2} - \phi_{4,1,2}$
13	$\phi_{3,1,2} - \phi_{4,1,2}$
14	$2\phi_{3,1,4} - \phi_{5,1,3} - \phi_{5,1,4}$
15	$\phi_{5,1,3} - \phi_{5,1,4}$
16	$4\phi_{6,2,7} - \phi_{6,2,1} - \phi_{6,2,8} - \phi_{7,2,1} - \phi_{7,2,8}$
17	$\phi_{6,2,1} + \phi_{6,2,8} - \phi_{7,2,1} - \phi_{7,2,8}$
18	$\phi_{6,2,1} - \phi_{6,2,8} + \phi_{7,2,1} - \phi_{7,2,8}$
19	$\phi_{6,2,1} - \phi_{6,2,8} - \phi_{7,2,1} + \phi_{7,2,8}$
20	$\tau_{5,1,2,8} + \tau_{3,1,2,8} + \tau_{4,1,2,8}$
21	$\tau_{9,8,2,1}$

S4.44 acetonitrile

Table S173: CCSD(T)/cc-pVTZ Optimum Cartesian coordinates (bohr)

1	N	2.51255949	−0.00000000	0.00000000
2	C	0.31660733	−0.00000000	0.00000000
3	C	−2.45420572	0.00000000	−0.00000000
4	H	−3.15278789	0.96811431	−1.67682317
5	H	−3.15278789	−1.93622862	−0.00000000
6	H	−3.15278789	0.96811431	1.67682317

Table S174: Reference [CCSD(T)/cc-pVTZ] CMA-0A and CMA-2A (ξ = 0.02) harmonic frequencies for Level B = MP2/cc-pVTZ and CCSD(T)/cc-pVDZ.

	Reference CCSD(T)/ cc-pVTZ	Pure MP2/ cc-pVTZ	CMA-0A MP2/ cc-pVTZ	CMA-2A MP2/ cc-pVTZ	Pure CCSD(T)/ cc-pVDZ	CMA-0A CCSD(T)/ cc-pVDZ	CMA-2A CCSD(T)/ cc-pVDZ
$\omega_1(a_1)$	3066.03	3078.30	3066.02	3066.02	3197.66	3065.99	3065.99
$\omega_2(a_1)$	2298.73	2261.22	2298.70	2298.70	2398.96	2298.75	2298.75
$\omega_3(a_1)$	1414.02	1409.05	1414.03	1414.03	1434.36	1413.83	1413.83
$\omega_4(a_1)$	921.28	919.61	921.35	921.35	960.51	921.63	921.63
$\omega_{5\mathrm{a}}(e)$	3149.91	3171.98	3149.91	3149.91	3300.78	3149.87	3149.87
$\omega_{5\mathrm{b}}(e)$	3149.91	3171.86	3149.91	3149.91	3300.66	3149.87	3149.87
$\omega_{6\mathrm{a}}(e)$	1487.57	1490.61	1487.56	1487.56	1497.22	1487.60	1487.60
$\omega_{6\mathrm{b}}(e)$	1487.57	1490.60	1487.56	1487.56	1497.21	1487.60	1487.60
$\omega_{7\mathrm{a}}(e)$	1062.13	1062.26	1062.14	1062.14	1072.97	1062.21	1062.21
$\omega_{7\mathrm{b}}(e)$	1062.13	1062.25	1062.14	1062.14	1072.96	1062.21	1062.21
$\omega_{8\mathrm{a}}(e)$	361.14	362.28	361.14	361.14	369.49	361.14	361.14
$\omega_{8\mathrm{b}}(e)$	361.14	362.27	361.14	361.14	369.48	361.14	361.14

Table S175: CMA-0A and CMA-2A (ξ = 0.02) harmonic frequencies targeting CCSD(T)/cc-pVTZ for Level B = B3LYP/6-31G(2*df*,*p*).

	Pure B3LYP/ 6-31G(2*df*,*p*)	CMA-0A B3LYP/ 6-31G(2*df*,*p*)	CMA-2A B3LYP/ 6-31G(2*df*,*p*)
$\omega_1(a_1)$	3086.02	3065.94	3065.94
$\omega_2(a_1)$	2318.48	2298.57	2298.57
$\omega_3(a_1)$	1409.68	1414.01	1414.01
$\omega_4(a_1)$	909.55	921.96	921.96
$\omega_{5\mathrm{a}}(e)$	3165.43	3149.91	3149.91
$\omega_{5\mathrm{b}}(e)$	3165.32	3149.91	3149.91
$\omega_{6\mathrm{a}}(e)$	1476.65	1487.52	1487.52
$\omega_{6\mathrm{b}}(e)$	1476.61	1487.52	1487.52
$\omega_{7\mathrm{a}}(e)$	1058.48	1062.15	1062.18
$\omega_{7\mathrm{b}}(e)$	1058.47	1062.15	1062.18
$\omega_{8\mathrm{a}}(e)$	377.06	361.26	361.16
$\omega_{8\mathrm{b}}(e)$	377.05	361.26	361.16

Table S176: Symmetrized, unnormalized natural internal coordinates for acetonitrile.

1	$r_{1,2}$
2	$r_{2,3}$
3	$r_{3,4} + r_{3,5} + r_{3,6}$
4	$2r_{3,4} - r_{3,5} - r_{3,6}$
5	$r_{3,5} - r_{3,6}$
6	$\phi_{4,3,2} + \phi_{5,3,2} + \phi_{6,3,2} - \phi_{5,3,6} - \phi_{4,3,5} - \phi_{4,3,6}$
7	$2\phi_{4,3,2} - \phi_{5,3,2} - \phi_{6,3,2}$
8	$\phi_{5,3,2} - \phi_{6,3,2}$
9	$2\phi_{5,3,6} - \phi_{4,3,5} - \phi_{4,3,6}$
10	$\phi_{4,3,5} - \phi_{4,3,6}$
11	$2\alpha^x_{4,3,2,1} - \alpha^x_{5,3,2,1} - \alpha^x_{6,3,2,1}$
12	$2\alpha^y_{4,3,2,1} - \alpha^y_{5,3,2,1} - \alpha^y_{6,3,2,1}$

S4.45 propyne

Table S177: CCSD(T)/cc-pVTZ Optimum Cartesian coordinates (bohr)

1	H	4.59758364	−0.00000001	0.00000000
2	C	2.58924928	−0.00000000	−0.00000000
3	C	0.30051907	0.00000001	−0.00000000
4	C	−2.47081506	−0.00000000	−0.00000000
5	H	−3.19532958	1.92992565	0.00000000
6	H	−3.19532957	−0.96496284	−1.67136465
7	H	−3.19532957	−0.96496284	1.67136465

Table S178: Reference [CCSD(T)/cc-pVTZ] CMA-0A and CMA-2A (ξ = 0.02) harmonic frequencies for Level B = MP2/cc-pVTZ and CCSD(T)/cc-pVDZ.

	Reference CCSD(T)/ cc-pVTZ	Pure MP2/ cc-pVTZ	CMA-0A MP2/ cc-pVTZ	CMA-2A MP2/ cc-pVTZ	Pure CCSD(T)/ cc-pVDZ	CMA-0A CCSD(T)/ cc-pVDZ	CMA-2A CCSD(T)/ cc-pVDZ
$\omega_1(a_1)$	3470.25	3483.45	3470.23	3470.23	3616.64	3470.24	3470.24
$\omega_2(a_1)$	3048.35	3060.93	3048.34	3048.34	3177.87	3048.30	3048.30
$\omega_3(a_1)$	2177.61	2158.84	2177.64	2177.64	2277.60	2177.64	2177.64
$\omega_4(a_1)$	1417.12	1410.48	1417.13	1417.13	1437.88	1417.01	1417.01
$\omega_5(a_1)$	935.50	934.18	935.51	935.51	972.78	935.80	935.80
$\omega_{6a}(e)$	3122.33	3145.99	3122.33	3122.33	3271.47	3122.29	3122.29
$\omega_{6b}(e)$	3122.33	3145.88	3122.33	3122.33	3271.35	3122.29	3122.29
$\omega_{7a}(e)$	1491.36	1493.73	1491.35	1491.35	1501.21	1491.41	1491.41
$\omega_{7b}(e)$	1491.36	1493.71	1491.35	1491.35	1501.20	1491.41	1491.41
$\omega_{8a}(e)$	1059.94	1059.07	1059.96	1059.96	1071.71	1059.99	1059.99
$\omega_{8b}(e)$	1059.94	1059.06	1059.95	1059.96	1071.70	1059.99	1059.99
$\omega_{9a}(e)$	619.78	627.61	619.76	619.78	635.25	619.77	619.79
$\omega_{9b}(e)$	619.78	627.58	619.76	619.78	635.24	619.77	619.79
$\omega_{10a}(e)$	322.43	325.91	322.49	322.43	331.70	322.48	322.44
$\omega_{10b}(e)$	322.43	325.90	322.49	322.43	331.68	322.48	322.44

Table S179: CMA-0A and CMA-2A (ξ = 0.02) harmonic frequencies targeting CCSD(T)/cc-pVTZ for Level B = B3LYP/6-31G(2*df*,*p*).

	Pure B3LYP/ 6-31G(2*df*,*p*)	CMA-0A B3LYP/ 6-31G(2*df*,*p*)	CMA-2A B3LYP/ 6-31G(2*df*,*p*)
$\omega_1(a_1)$	3492.04	3470.25	3470.25
$\omega_2(a_1)$	3066.06	3048.27	3048.27
$\omega_3(a_1)$	2186.88	2177.48	2177.48
$\omega_4(a_1)$	1413.65	1417.09	1417.09
$\omega_5(a_1)$	919.56	936.12	936.12
$\omega_{6a}(e)$	3135.14	3122.33	3122.33
$\omega_{6b}(e)$	3135.04	3122.33	3122.33
$\omega_{7a}(e)$	1480.31	1491.29	1491.29
$\omega_{7b}(e)$	1480.27	1491.29	1491.29
$\omega_{8a}(e)$	1057.64	1060.01	1060.02
$\omega_{8b}(e)$	1057.62	1060.01	1060.02
$\omega_{9a}(e)$	630.72	619.79	619.79
$\omega_{9b}(e)$	630.69	619.79	619.79
$\omega_{10a}(e)$	331.29	322.51	322.48
$\omega_{10b}(e)$	331.28	322.50	322.48

Table S180: Symmetrized, unnormalized natural internal coordinates for propyne.

1	$r_{1,2}$
2	$r_{2,3}$
3	$r_{3,4}$
4	$r_{4,5} + r_{4,6} + r_{4,7}$
5	$2r_{4,5} - r_{4,6} - r_{4,7}$
6	$r_{4,6} - r_{4,7}$
7	$\phi_{6,4,7} + \phi_{5,4,6} + \phi_{5,4,7} - \phi_{5,4,3} - \phi_{6,4,3} - \phi_{7,4,3}$
8	$2\phi_{6,4,7} - \phi_{5,4,6} - \phi_{5,4,7}$
9	$\phi_{5,4,6} - \phi_{5,4,7}$
10	$2\phi_{5,4,3} - \phi_{6,4,3} - \phi_{7,4,3}$
11	$\phi_{6,4,3} - \phi_{7,4,3}$
12	$2\alpha^x_{5,4,3,2} - \alpha^x_{6,4,3,2} - \alpha^x_{7,4,3,2}$
13	$\alpha^x_{6,4,3,2} - \alpha^x_{7,4,3,2}$
14	$2\alpha^x_{5,4,2,1} - \alpha^x_{6,4,2,1} - \alpha^x_{7,4,2,1}$
15	$\alpha^x_{6,4,2,1} - \alpha^x_{7,4,2,1}$

S4.46 trifluoroacetonitrile

Table S181: CCSD(T)/cc-pVTZ Optimum Cartesian coordinates (bohr)

1	N	4.43122755	−0.00000000	0.00000000
2	C	2.23806724	−0.00000000	0.00000000
3	C	−0.57616305	−0.00000000	0.00000000
4	F	−1.43860612	1.17812158	−2.04056644
5	F	−1.43860612	−2.35624316	−0.00000000
6	F	−1.43860612	1.17812158	2.04056644

Table S182: Reference [CCSD(T)/cc-pVTZ] CMA-0A and CMA-2A (ξ = 0.02) harmonic frequencies for Level B = MP2/cc-pVTZ and CCSD(T)/cc-pVDZ.

	Reference CCSD(T)/ cc-pVTZ	Pure MP2/ cc-pVTZ	CMA-0A MP2/ cc-pVTZ	CMA-2A MP2/ cc-pVTZ	Pure CCSD(T)/ cc-pVDZ	CMA-0A CCSD(T)/ cc-pVDZ	CMA-2A CCSD(T)/ cc-pVDZ
$\omega_1(a_1)$	2304.40	2254.12	2304.38	2304.39	2400.16	2304.39	2304.39
$\omega_2(a_1)$	1258.85	1254.43	1258.85	1258.85	1289.04	1258.73	1258.76
$\omega_3(a_1)$	821.09	821.56	821.11	821.10	831.38	821.12	821.08
$\omega_4(a_1)$	527.15	526.30	527.20	527.20	535.76	527.43	527.43
$\omega_{5\text{a}}(e)$	1258.53	1246.99	1258.53	1258.53	1303.61	1258.52	1258.52
$\omega_{5\text{b}}(e)$	1258.53	1246.94	1258.53	1258.53	1303.56	1258.52	1258.52
$\omega_{6\text{a}}(e)$	629.30	631.93	629.30	629.30	634.70	629.28	629.30
$\omega_{6\text{b}}(e)$	629.30	631.92	629.30	629.30	634.70	629.28	629.30
$\omega_{7\text{a}}(e)$	469.33	470.42	469.34	469.33	474.17	469.37	469.34
$\omega_{7\text{b}}(e)$	469.33	470.41	469.34	469.33	474.16	469.37	469.34
$\omega_{8\text{a}}(e)$	189.86	190.44	189.88	189.87	189.09	189.87	189.86
$\omega_{8\text{b}}(e)$	189.86	190.44	189.88	189.87	189.08	189.87	189.86

Table S183: CMA-0A and CMA-2A (ξ = 0.02) harmonic frequencies targeting CCSD(T)/cc-pVTZ for Level B = B3LYP/6-31G(2*df*,*p*).

	Pure B3LYP/ 6-31G(2*df*,*p*)	CMA-0A B3LYP/ 6-31G(2*df*,*p*)	CMA-2A B3LYP/ 6-31G(2*df*,*p*)
$\omega_1(a_1)$	2329.48	2304.10	2304.36
$\omega_2(a_1)$	1235.01	1258.97	1258.86
$\omega_3(a_1)$	820.59	821.60	821.16
$\omega_4(a_1)$	519.56	527.38	527.16
$\omega_{5\text{a}}(e)$	1251.58	1258.52	1258.52
$\omega_{5\text{b}}(e)$	1250.94	1258.52	1258.52
$\omega_{6\text{a}}(e)$	627.96	629.16	629.23
$\omega_{6\text{b}}(e)$	627.84	629.14	629.22
$\omega_{7\text{a}}(e)$	466.93	469.33	469.23
$\omega_{7\text{b}}(e)$	466.86	469.32	469.21
$\omega_{8\text{a}}(e)$	191.61	190.47	190.47
$\omega_{8\text{b}}(e)$	191.32	190.43	190.43

Table S184: Symmetrized, unnormalized natural internal coordinates for trifluoroacetonitrile.

1	$r_{1,2}$
2	$r_{2,3}$
3	$r_{3,4} + r_{3,5} + r_{3,6}$
4	$2r_{3,4} - r_{3,5} - r_{3,6}$
5	$r_{3,5} - r_{3,6}$
6	$\phi_{4,3,2} + \phi_{5,3,2} + \phi_{6,3,2} - \phi_{5,3,6} - \phi_{4,3,5} - \phi_{4,3,6}$
7	$2\phi_{4,3,2} - \phi_{5,3,2} - \phi_{6,3,2}$
8	$\phi_{5,3,2} - \phi_{6,3,2}$
9	$2\phi_{5,3,6} - \phi_{4,3,5} - \phi_{4,3,6}$
10	$\phi_{4,3,5} - \phi_{4,3,6}$
11	$2\alpha^x_{4,3,2,1} - \alpha^x_{5,3,2,1} - \alpha^x_{6,3,2,1}$
12	$2\alpha^y_{4,3,2,1} - \alpha^y_{5,3,2,1} - \alpha^y_{6,3,2,1}$

S4.47 silicon tetrachloride

Table S185: CCSD(T)/cc-pVTZ Optimum Cartesian coordinates (bohr)

1	Si	0.00000000	0.00000000	−0.00000000
2	Cl	0.00000000	−3.13063739	2.21369401
3	Cl	0.00000000	3.13063739	2.21369401
4	Cl	3.13063738	0.00000000	−2.21369401
5	Cl	−3.13063738	0.00000000	−2.21369401

Table S186: Reference [CCSD(T)/cc-pVTZ] CMA-0A and CMA-2A (ξ = 0.02) harmonic frequencies for Level B = MP2/cc-pVTZ and CCSD(T)/cc-pVDZ.

	Reference CCSD(T)/ cc-pVTZ	Pure MP2/ cc-pVTZ	CMA-0A MP2/ cc-pVTZ	CMA-2A MP2/ cc-pVTZ	Pure CCSD(T)/ cc-pVDZ	CMA-0A CCSD(T)/ cc-pVDZ	CMA-2A CCSD(T)/ cc-pVDZ
$\omega_1(a_1)$	424.70	429.07	424.70	424.70	430.21	424.70	424.70
$\omega_{2\mathrm{a}}(e)$	146.57	146.22	146.57	146.57	149.21	146.57	146.57
$\omega_{2\mathrm{b}}(e)$	146.56	146.22	146.57	146.57	149.21	146.57	146.57
$\omega_{3\mathrm{a}}(t_2)$	627.23	633.58	627.21	627.21	641.76	627.21	627.21
$\omega_{3\mathrm{b}}(t_2)$	627.19	633.54	627.20	627.20	641.72	627.20	627.20
$\omega_{3\mathrm{c}}(t_2)$	627.16	633.54	627.17	627.17	641.72	627.16	627.16
$\omega_{4\mathrm{a}}(t_2)$	221.78	221.01	221.71	221.71	224.57	221.71	221.71
$\omega_{4\mathrm{b}}(t_2)$	221.56	220.99	221.60	221.60	224.55	221.63	221.63
$\omega_{4\mathrm{c}}(t_2)$	221.54	220.99	221.58	221.58	224.55	221.54	221.54

Table S187: CMA-0A and CMA-2A (ξ = 0.02) harmonic frequencies targeting CCSD(T)/cc-pVTZ for Level B = B3LYP/6-31G(2*df*,*p*).

	Pure B3LYP/ 6-31G(2*df*,*p*)	CMA-0A B3LYP/ 6-31G(2*df*,*p*)	CMA-2A B3LYP/ 6-31G(2*df*,*p*)
$\omega_1(a_1)$	419.86	424.70	424.70
$\omega_{2\mathrm{a}}(e)$	146.10	146.57	146.57
$\omega_{2\mathrm{b}}(e)$	145.60	146.56	146.56
$\omega_{3\mathrm{a}}(t_2)$	624.70	627.20	627.20
$\omega_{3\mathrm{b}}(t_2)$	622.53	627.17	627.17
$\omega_{3\mathrm{c}}(t_2)$	622.53	627.16	627.16
$\omega_{4\mathrm{a}}(t_2)$	220.71	221.84	221.84
$\omega_{4\mathrm{b}}(t_2)$	220.71	221.63	221.63
$\omega_{4\mathrm{c}}(t_2)$	217.52	221.54	221.54

Table S188: Symmetrized, unnormalized natural internal coordinates for silicon tetrachloride.

1	$r_{1,2} + r_{1,3} + r_{1,4} + r_{1,5}$
2	$-r_{1,2} - r_{1,3} + r_{1,4} + r_{1,5}$
3	$-r_{1,2} + r_{1,3} - r_{1,4} + r_{1,5}$
4	$r_{1,2} - r_{1,3} - r_{1,4} + r_{1,5}$
5	$2\phi_{2,1,3} + 2\phi_{4,1,5} - \phi_{2,1,4} - \phi_{2,1,5} - \phi_{3,1,4} - \phi_{3,1,5}$
6	$\phi_{2,1,4} - \phi_{2,1,5} - \phi_{3,1,4} + \phi_{3,1,5}$
7	$-\phi_{2,1,3} + \phi_{4,1,5}$
8	$-\phi_{2,1,4} + \phi_{3,1,5}$
9	$\phi_{2,1,5} - \phi_{3,1,4}$

S4.48 silicon tetrafluoride

Table S189: CCSD(T)/cc-pVTZ Optimum Cartesian coordinates (bohr)

1	Si	0.00000000	0.00000000	−0.00000003
2	F	0.00000000	−2.40960515	1.70384729
3	F	0.00000000	2.40960515	1.70384729
4	F	2.40960519	0.00000000	−1.70384727
5	F	−2.40960519	0.00000000	−1.70384727

Table S190: Reference [CCSD(T)/cc-pVTZ] CMA-0A and CMA-2A (ξ = 0.02) harmonic frequencies for Level B = MP2/cc-pVTZ and CCSD(T)/cc-pVDZ.

	Reference CCSD(T)/ cc-pVTZ	Pure MP2/ cc-pVTZ	CMA-0A MP2/ cc-pVTZ	CMA-2A MP2/ cc-pVTZ	Pure CCSD(T)/ cc-pVDZ	CMA-0A CCSD(T)/ cc-pVDZ	CMA-2A CCSD(T)/ cc-pVDZ
$\omega_1(a_1)$	798.06	813.90	798.06	798.06	817.53	798.06	798.06
$\omega_{2\mathrm{a}}(e)$	263.27	263.80	263.25	263.25	263.91	263.25	263.25
$\omega_{2\mathrm{b}}(e)$	263.22	263.80	263.24	263.24	263.91	263.24	263.24
$\omega_{3\mathrm{a}}(t_2)$	1038.07	1062.82	1038.06	1038.06	1088.54	1038.05	1038.05
$\omega_{3\mathrm{b}}(t_2)$	1038.05	1062.73	1038.05	1038.05	1088.46	1038.05	1038.05
$\omega_{3\mathrm{c}}(t_2)$	1038.05	1062.73	1038.05	1038.05	1088.46	1038.05	1038.05
$\omega_{4\mathrm{a}}(t_2)$	387.78	389.58	387.79	387.79	389.85	387.80	387.80
$\omega_{4\mathrm{b}}(t_2)$	387.76	389.55	387.76	387.76	389.82	387.77	387.77
$\omega_{4\mathrm{c}}(t_2)$	387.72	389.55	387.75	387.75	389.82	387.76	387.76

Table S191: CMA-0A and CMA-2A (ξ = 0.02) harmonic frequencies targeting CCSD(T)/cc-pVTZ for Level B = B3LYP/6-31G(2*df*,*p*).

	Pure B3LYP/ 6-31G(2*df*,*p*)	CMA-0A B3LYP/ 6-31G(2*df*,*p*)	CMA-2A B3LYP/ 6-31G(2*df*,*p*)
$\omega_1(a_1)$	799.97	798.06	798.06
$\omega_{2\mathrm{a}}(e)$	266.79	263.27	263.27
$\omega_{2\mathrm{b}}(e)$	263.83	263.22	263.22
$\omega_{3\mathrm{a}}(t_2)$	1055.74	1038.06	1038.06
$\omega_{3\mathrm{b}}(t_2)$	1055.73	1038.05	1038.05
$\omega_{3\mathrm{c}}(t_2)$	1054.40	1038.05	1038.05
$\omega_{4\mathrm{a}}(t_2)$	387.53	387.79	387.79
$\omega_{4\mathrm{b}}(t_2)$	387.53	387.77	387.77
$\omega_{4\mathrm{c}}(t_2)$	385.46	387.75	387.75

Table S192: Symmetrized, unnormalized natural internal coordinates for silicon tetrafluoride.

1	$r_{1,2} + r_{1,3} + r_{1,4} + r_{1,5}$
2	$-r_{1,2} - r_{1,3} + r_{1,4} + r_{1,5}$
3	$-r_{1,2} + r_{1,3} - r_{1,4} + r_{1,5}$
4	$r_{1,2} - r_{1,3} - r_{1,4} + r_{1,5}$
5	$2\phi_{2,1,3} + 2\phi_{4,1,5} - \phi_{2,1,4} - \phi_{2,1,5} - \phi_{3,1,4} - \phi_{3,1,5}$
6	$\phi_{2,1,4} - \phi_{2,1,5} - \phi_{3,1,4} + \phi_{3,1,5}$
7	$-\phi_{2,1,3} + \phi_{4,1,5}$
8	$-\phi_{2,1,4} + \phi_{3,1,5}$
9	$\phi_{2,1,5} - \phi_{3,1,4}$

S4.49 disilane

Table S193: CCSD(T)/cc-pVTZ Optimum Cartesian coordinates (bohr)

1	Si	0.00000209	2.21895084	0.00000000
2	Si	−0.00000209	−2.21895084	0.00000000
3	H	−1.31404950	3.19278368	−2.27600840
4	H	−1.31404950	3.19278368	2.27600840
5	H	2.62810651	3.19279459	0.00000000
6	H	−2.62810651	−3.19279459	0.00000000
7	H	1.31404950	−3.19278368	2.27600840
8	H	1.31404950	−3.19278368	−2.27600840

Table S194: Reference [CCSD(T)/cc-pVTZ] CMA-0A and CMA-2A (ξ = 0.02) harmonic frequencies for Level B = MP2/cc-pVTZ and CCSD(T)/cc-pVDZ.

	Reference CCSD(T)/ cc-pVTZ	Pure MP2/ cc-pVTZ	CMA-0A MP2/ cc-pVTZ	CMA-2A MP2/ cc-pVTZ	Pure CCSD(T)/ cc-pVDZ	CMA-0A CCSD(T)/ cc-pVDZ	CMA-2A CCSD(T)/ cc-pVDZ
$\omega_1(a_{1g})$	2230.82	2264.41	2230.82	2230.82	2260.63	2230.82	2230.82
$\omega_2(a_{1g})$	935.18	946.05	935.18	935.18	941.86	935.18	935.18
$\omega_3(a_{1g})$	437.94	442.97	437.94	437.94	443.16	437.95	437.95
$\omega_{4a}(e_g)$	2238.14	2274.92	2238.14	2238.14	2271.38	2238.14	2238.14
$\omega_{4b}(e_u)$	2238.14	2274.92	2238.14	2238.14	2271.38	2238.14	2238.14
$\omega_{5a}(e_g)$	2229.68	2266.19	2229.68	2229.68	2263.83	2229.68	2229.68
$\omega_{5b}(e_u)$	2229.68	2266.19	2229.68	2229.68	2263.83	2229.68	2229.68
$\omega_{6a}(e_g)$	966.39	981.33	966.39	966.39	970.53	966.39	966.39
$\omega_{6b}(e_u)$	966.37	981.33	966.37	966.37	970.53	966.37	966.37
$\omega_{7a}(e_g)$	952.28	967.07	952.27	952.27	956.00	952.28	952.28
$\omega_{7b}(e_u)$	952.27	967.07	952.27	952.27	956.00	952.27	952.27
$\omega_{8a}(e_g)$	636.90	643.05	636.91	636.91	641.48	636.91	636.91
$\omega_{8b}(e_u)$	636.90	643.05	636.91	636.91	641.48	636.90	636.90
$\omega_{9a}(e_g)$	371.94	372.82	371.94	371.94	374.81	371.95	371.94
$\omega_{9b}(e_u)$	371.94	372.83	371.94	371.94	374.81	371.94	371.94
$\omega_{10}(a_{1u})$	137.39	139.62	137.39	137.39	137.70	137.39	137.39
$\omega_{11}(a_{2u})$	2221.82	2256.35	2221.82	2221.82	2251.82	2221.82	2221.82
$\omega_{12}(a_{2u})$	860.79	869.26	860.79	860.79	869.71	860.79	860.79

Table S195: CMA-0A and CMA-2A (ξ = 0.02) harmonic frequencies targeting CCSD(T)/cc-pVTZ for Level B = B3LYP/6-31G(2*df*,*p*).

	Pure B3LYP/ 6-31G(2*df*,*p*)	CMA-0A B3LYP/ 6-31G(2*df*,*p*)	CMA-2A B3LYP/ 6-31G(2*df*,*p*)
$\omega_1(a_{1g})$	2240.94	2230.82	2230.82
$\omega_2(a_{1g})$	927.59	935.17	935.17
$\omega_3(a_{1g})$	427.61	437.98	437.98
$\omega_{4a}(e_g)$	2253.27	2238.13	2238.13
$\omega_{4b}(e_u)$	2243.60	2229.68	2229.68
$\omega_{5a}(e_g)$	959.00	966.39	966.39
$\omega_{5b}(e_u)$	945.55	952.25	952.28
$\omega_{6a}(e_g)$	635.78	636.96	636.91
$\omega_{6b}(e_u)$	375.07	371.97	371.96
$\omega_{7a}(e_g)$	130.33	137.39	137.39
$\omega_{7b}(e_u)$	2232.94	2221.82	2221.82
$\omega_{8a}(e_g)$	854.58	860.79	860.79
$\omega_{8b}(e_u)$	2253.22	2238.14	2238.14
$\omega_{9a}(e_g)$	2243.33	2229.68	2229.68
$\omega_{9b}(e_u)$	958.91	966.37	966.37
$\omega_{10}(a_{1u})$	945.29	952.25	952.27
$\omega_{11}(a_{2u})$	635.80	636.95	636.91
$\omega_{12}(a_{2u})$	375.08	371.96	371.96

Table S196: Symmetrized, unnormalized natural internal coordinates for disilane.

1	$r_{1,2}$
2	$r_{1,3} + r_{1,4} + r_{1,5} + r_{2,7} + r_{2,6} + r_{2,8}$
3	$r_{1,3} + r_{1,4} + r_{1,5} - r_{2,7} - r_{2,6} - r_{2,8}$
4	$2r_{1,3} - r_{1,4} - r_{1,5} + 2r_{2,7} - r_{2,6} - r_{2,8}$
5	$2r_{1,3} - r_{1,4} - r_{1,5} - 2r_{2,7} + r_{2,6} + r_{2,8}$
6	$r_{1,4} - r_{1,5} + r_{2,6} - r_{2,8}$
7	$r_{1,4} - r_{1,5} - r_{2,6} + r_{2,8}$
8	$\phi_{4,1,5} + \phi_{3,1,4} + \phi_{3,1,5} - \phi_{3,1,2} - \phi_{4,1,2} - \phi_{5,1,2} + \phi_{6,2,8} + \phi_{7,2,6} + \phi_{7,2,8} - \phi_{7,2,1} - \phi_{6,2,1} - \phi_{8,2,1}$
9	$\phi_{4,1,5} + \phi_{3,1,4} + \phi_{3,1,5} - \phi_{3,1,2} - \phi_{4,1,2} - \phi_{5,1,2} - \phi_{6,2,8} - \phi_{7,2,6} - \phi_{7,2,8} + \phi_{7,2,1} + \phi_{6,2,1} + \phi_{8,2,1}$
10	$2\phi_{4,1,5} - \phi_{3,1,4} - \phi_{3,1,5} + 2\phi_{6,2,8} - \phi_{7,2,6} - \phi_{7,2,8}$
11	$2\phi_{4,1,5} - \phi_{3,1,4} - \phi_{3,1,5} - 2\phi_{6,2,8} + \phi_{7,2,6} + \phi_{7,2,8}$
12	$\phi_{3,1,4} - \phi_{3,1,5} + \phi_{7,2,6} - \phi_{7,2,8}$
13	$\phi_{3,1,4} - \phi_{3,1,5} - \phi_{7,2,6} + \phi_{7,2,8}$
14	$2\phi_{3,1,2} - \phi_{4,1,2} - \phi_{5,1,2} + 2\phi_{7,2,1} - \phi_{6,2,1} - \phi_{8,2,1}$
15	$2\phi_{3,1,2} - \phi_{4,1,2} - \phi_{5,1,2} - 2\phi_{7,2,1} + \phi_{6,2,1} + \phi_{8,2,1}$
16	$\phi_{4,1,2} - \phi_{5,1,2} + \phi_{6,2,1} - \phi_{8,2,1}$
17	$\phi_{4,1,2} - \phi_{5,1,2} - \phi_{6,2,1} + \phi_{8,2,1}$
18	$\tau_{3,1,2,7} + \tau_{7,2,1,3} + \tau_{4,1,2,8} + \tau_{8,2,1,4} + \tau_{5,1,2,6} + \tau_{6,2,1,5}$

S4.50 methyl silane

Table S197: CCSD(T)/cc-pVTZ Optimum Cartesian coordinates (bohr)

1	Si	1.14265735	−0.00000106	0.00000000
2	C	−2.40853504	0.00000395	0.00000000
3	H	2.12904008	−1.31148904	−2.27156471
4	H	2.12905165	2.62297024	0.00000000
5	H	2.12904008	−1.31148904	2.27156471
6	H	−3.14298917	0.96423858	−1.67011742
7	H	−3.14298917	0.96423858	1.67011742
8	H	−3.14297141	−1.92848698	0.00000000

Table S198: Reference [CCSD(T)/cc-pVTZ] CMA-0A and CMA-2A ($\xi = 0.02$) harmonic frequencies for Level B = MP2/cc-pVTZ and CCSD(T)/cc-pVDZ.

	Reference CCSD(T)/ cc-pVTZ	Pure MP2/ cc-pVTZ	CMA-0A MP2/ cc-pVTZ	CMA-2A MP2/ cc-pVTZ	Pure CCSD(T)/ cc-pVDZ	CMA-0A CCSD(T)/ cc-pVDZ	CMA-2A CCSD(T)/ cc-pVDZ
$\omega_1(a_1)$	3038.03	3051.73	3038.02	3038.02	3173.56	3038.02	3038.02
$\omega_2(a_1)$	2235.39	2268.92	2235.39	2235.39	2264.26	2235.38	2235.38
$\omega_3(a_1)$	1294.07	1287.64	1294.06	1294.06	1309.30	1294.06	1294.06
$\omega_4(a_1)$	957.95	968.61	957.94	957.94	965.71	957.95	957.95
$\omega_5(a_1)$	705.49	711.99	705.53	705.53	710.98	705.58	705.58
$\omega_6(a_2)$	204.58	206.67	204.58	204.58	211.55	204.58	204.58
$\omega_{7\mathrm{a}}(e)$	3122.34	3147.18	3122.34	3122.34	3274.29	3122.30	3122.30
$\omega_{7\mathrm{b}}(e)$	3122.33	3147.07	3122.34	3122.34	3274.18	3122.30	3122.30
$\omega_{8\mathrm{a}}(e)$	2233.66	2269.68	2233.65	2233.65	2267.22	2233.66	2233.66
$\omega_{8\mathrm{b}}(e)$	2233.65	2269.63	2233.65	2233.65	2267.18	2233.66	2233.66
$\omega_{9\mathrm{a}}(e)$	1469.44	1470.86	1469.43	1469.43	1481.15	1469.46	1469.46
$\omega_{9\mathrm{b}}(e)$	1469.42	1470.84	1469.41	1469.41	1481.13	1469.44	1469.44
$\omega_{10\mathrm{a}}(e)$	973.70	987.95	973.69	973.69	977.06	973.68	973.68
$\omega_{10\mathrm{b}}(e)$	973.68	987.93	973.68	973.68	977.04	973.67	973.67
$\omega_{11\mathrm{a}}(e)$	887.60	889.84	887.61	887.61	900.18	887.66	887.66
$\omega_{11\mathrm{b}}(e)$	887.60	889.84	887.61	887.61	900.17	887.66	887.66
$\omega_{12\mathrm{a}}(e)$	517.82	520.26	517.85	517.85	520.03	517.93	517.93
$\omega_{12\mathrm{b}}(e)$	517.82	520.25	517.84	517.84	520.01	517.92	517.92

Table S199: CMA-0A and CMA-2A (ξ = 0.02) harmonic frequencies targeting CCSD(T)/cc-pVTZ for Level B = B3LYP/6-31G(2*df*,*p*).

	Pure B3LYP/ 6-31G(2*df*,*p*)	CMA-0A B3LYP/ 6-31G(2*df*,*p*)	CMA-2A B3LYP/ 6-31G(2*df*,*p*)
$\omega_1(a_1)$	3058.56	3038.00	3038.00
$\omega_2(a_1)$	2243.71	2235.39	2235.39
$\omega_3(a_1)$	1299.14	1294.01	1294.13
$\omega_4(a_1)$	953.61	957.95	957.95
$\omega_5(a_1)$	698.78	705.73	705.51
$\omega_6(a_2)$	3138.41	3122.32	3122.32
$\omega_{7\mathrm{a}}(e)$	199.25	204.58	204.58
$\omega_{7\mathrm{b}}(e)$	3138.01	3122.32	3122.32
$\omega_{8\mathrm{a}}(e)$	2246.47	2233.66	2233.66
$\omega_{8\mathrm{b}}(e)$	2246.07	2233.65	2233.65
$\omega_{9\mathrm{a}}(e)$	1471.57	1469.42	1469.42
$\omega_{9\mathrm{b}}(e)$	1471.21	1469.40	1469.40
$\omega_{10\mathrm{a}}(e)$	964.66	973.56	973.56
$\omega_{10\mathrm{b}}(e)$	964.38	973.55	973.55
$\omega_{11\mathrm{a}}(e)$	893.39	887.68	887.78
$\omega_{11\mathrm{b}}(e)$	893.35	887.67	887.78
$\omega_{12\mathrm{a}}(e)$	524.28	518.10	517.93
$\omega_{12\mathrm{b}}(e)$	524.23	518.10	517.92

Table S200: Symmetrized, unnormalized natural internal coordinates for methyl silane.

1	$r_{1,2}$
2	$r_{1,4} + r_{1,3} + r_{1,5}$
3	$2r_{1,4} - r_{1,3} - r_{1,5}$
4	$r_{1,3} - r_{1,5}$
5	$r_{2,8} + r_{2,6} + r_{2,7}$
6	$2r_{2,8} - r_{2,6} - r_{2,7}$
7	$r_{2,6} - r_{2,7}$
8	$\phi_{4,1,2} + \phi_{3,1,2} + \phi_{5,1,2} - \phi_{3,1,5} - \phi_{4,1,3} - \phi_{4,1,5}$
9	$2\phi_{4,1,2} - \phi_{3,1,2} - \phi_{5,1,2}$
10	$\phi_{3,1,2} - \phi_{5,1,2}$
11	$2\phi_{3,1,5} - \phi_{4,1,3} - \phi_{4,1,5}$
12	$\phi_{4,1,3} - \phi_{4,1,5}$
13	$\phi_{8,2,1} + \phi_{6,2,1} + \phi_{7,2,1} - \phi_{6,2,7} - \phi_{8,2,6} - \phi_{8,2,7}$
14	$2\phi_{8,2,1} - \phi_{6,2,1} - \phi_{7,2,1}$
15	$\phi_{6,2,1} - \phi_{7,2,1}$
16	$2\phi_{6,2,7} - \phi_{8,2,6} - \phi_{8,2,7}$
17	$\phi_{8,2,6} - \phi_{8,2,7}$
18	$\tau_{8,2,1,4} + \tau_{6,2,1,5} + \tau_{7,2,1,3}$

S4.51 phosphane

Table S201: CCSD(T)/cc-pVTZ Optimum Cartesian coordinates (bohr)

1	P	0.12868063	0.00000031	0.00000000
2	H	−1.31825839	1.12497772	−1.94852696
3	H	−1.31826007	−2.24996482	0.00000000
4	H	−1.31825839	1.12497772	1.94852696

Table S202: Reference [CCSD(T)/cc-pVTZ] CMA-0A and CMA-2A (ξ = 0.02) harmonic frequencies for Level B = MP2/cc-pVTZ and CCSD(T)/cc-pVDZ.

	Reference CCSD(T)/ cc-pVTZ	Pure MP2/ cc-pVTZ	CMA-0A MP2/ cc-pVTZ	CMA-2A MP2/ cc-pVTZ	Pure CCSD(T)/ cc-pVDZ	CMA-0A CCSD(T)/ cc-pVDZ	CMA-2A CCSD(T)/ cc-pVDZ
$\omega_1(a_1)$	2415.52	2453.48	2415.52	2415.52	2480.05	2415.51	2415.51
$\omega_2(a_1)$	1021.69	1018.46	1021.69	1021.69	1048.83	1021.70	1021.70
$\omega_{3\mathrm{a}}(e)$	2422.82	2465.50	2422.82	2422.82	2491.33	2422.82	2422.82
$\omega_{3\mathrm{b}}(e)$	2422.82	2465.44	2422.82	2422.82	2491.27	2422.82	2422.82
$\omega_{4\mathrm{a}}(e)$	1145.25	1164.78	1145.25	1145.25	1162.13	1145.25	1145.25
$\omega_{4\mathrm{b}}(e)$	1145.25	1163.77	1145.25	1145.25	1161.09	1145.25	1145.25

Table S203: CMA-0A and CMA-2A (ξ = 0.02) harmonic frequencies targeting CCSD(T)/cc-pVTZ for Level B = B3LYP/6-31G(2*df*,*p*).

	Pure B3LYP/ 6-31G(2*df*,*p*)	CMA-0A B3LYP/ 6-31G(2*df*,*p*)	CMA-2A B3LYP/ 6-31G(2*df*,*p*)
$\omega_1(a_1)$	2432.09	2415.52	2415.52
$\omega_2(a_1)$	1027.63	1021.69	1021.69
$\omega_{3\mathrm{a}}(e)$	2444.02	2422.82	2422.82
$\omega_{3\mathrm{b}}(e)$	2443.84	2422.82	2422.82
$\omega_{4\mathrm{a}}(e)$	1142.91	1145.25	1145.25
$\omega_{4\mathrm{b}}(e)$	1141.89	1145.25	1145.25

Table S204: Symmetrized, unnormalized natural internal coordinates for phosphane.

1	$r_{1,2} + r_{1,3} + r_{1,4}$
2	$2r_{1,2} - r_{1,3} - r_{1,4}$
3	$r_{1,3} - r_{1,4}$
4	$2\phi_{2,1,3} - \phi_{2,1,4} - \phi_{3,1,4}$
5	$\phi_{2,1,4} - \phi_{3,1,4}$
6	$\gamma_{2,1,3,4} + \gamma_{3,1,4,2} + \gamma_{4,1,2,3}$

S4.52 phosphorus trifluoride

Table S205: CCSD(T)/cc-pVTZ Optimum Cartesian coordinates (bohr)

1	P	−0.00000092	0.94258107	0.00000000
2	F	−1.28867646	−0.51224165	2.23205457
3	F	−1.28867646	−0.51224165	−2.23205457
4	F	2.57735441	−0.51223963	0.00000000

Table S206: Reference [CCSD(T)/cc-pVTZ] CMA-0A and CMA-2A ($\xi = 0.02$) harmonic frequencies for Level B = MP2/cc-pVTZ and CCSD(T)/cc-pVDZ.

	Reference CCSD(T)/ cc-pVTZ	Pure MP2/ cc-pVTZ	CMA-0A MP2/ cc-pVTZ	CMA-2A MP2/ cc-pVTZ	Pure CCSD(T)/ cc-pVDZ	CMA-0A CCSD(T)/ cc-pVDZ	CMA-2A CCSD(T)/ cc-pVDZ
$\omega_1(a_1)$	915.76	929.73	915.74	915.74	962.94	915.60	915.60
$\omega_2(a_1)$	501.47	492.25	501.51	501.51	499.37	501.76	501.76
$\omega_{3\mathrm{a}}(e)$	897.81	907.21	897.81	897.81	960.93	897.79	897.79
$\omega_{3\mathrm{b}}(e)$	897.80	907.17	897.80	897.80	960.89	897.77	897.77
$\omega_{4\mathrm{a}}(e)$	359.14	348.44	359.13	359.13	352.90	359.20	359.20
$\omega_{4\mathrm{b}}(e)$	359.12	348.30	359.13	359.13	352.77	359.20	359.20

Table S207: CMA-0A and CMA-2A ($\xi = 0.02$) harmonic frequencies targeting CCSD(T)/cc-pVTZ for Level B = B3LYP/6-31G(2*df*,*p*).

	Pure B3LYP/ 6-31G(2*df*,*p*)	CMA-0A B3LYP/ 6-31G(2*df*,*p*)	CMA-2A B3LYP/ 6-31G(2*df*,*p*)
$\omega_1(a_1)$	915.63	915.75	915.75
$\omega_2(a_1)$	485.94	501.49	501.49
$\omega_{3\mathrm{a}}(e)$	904.38	897.81	897.81
$\omega_{3\mathrm{b}}(e)$	903.97	897.80	897.80
$\omega_{4\mathrm{a}}(e)$	346.01	359.13	359.13
$\omega_{4\mathrm{b}}(e)$	345.84	359.13	359.13

Table S208: Symmetrized, unnormalized natural internal coordinates for phosphorus trifluoride.

1	$r_{1,2} + r_{1,3} + r_{1,4}$
2	$2r_{1,2} - r_{1,3} - r_{1,4}$
3	$r_{1,3} - r_{1,4}$
4	$2\phi_{2,1,3} - \phi_{2,1,4} - \phi_{3,1,4}$
5	$\phi_{2,1,4} - \phi_{3,1,4}$
6	$\gamma_{2,1,3,4} + \gamma_{3,1,4,2} + \gamma_{4,1,2,3}$

S4.53 hypochlorous acid

Table S209: CCSD(T)/cc-pVTZ Optimum Cartesian coordinates (bohr)

1	H	−2.59688895	1.66013000	0.00000000
2	O	−2.15400490	−0.10905861	0.00000000
3	Cl	1.06009581	0.00203789	0.00000000

Table S210: Reference [CCSD(T)/cc-pVTZ] CMA-0A and CMA-2A (ξ = 0.02) harmonic frequencies for Level B = MP2/cc-pVTZ and CCSD(T)/cc-pVDZ.

	Reference CCSD(T)/ cc-pVTZ	Pure MP2/ cc-pVTZ	CMA-0A MP2/ cc-pVTZ	CMA-2A MP2/ cc-pVTZ	Pure CCSD(T)/ cc-pVDZ	CMA-0A CCSD(T)/ cc-pVDZ	CMA-2A CCSD(T)/ cc-pVDZ
$\omega_1(a^{'})$	3809.62	3822.33	3809.60	3809.60	3880.50	3809.53	3809.53
$\omega_2(a^{'})$	1281.33	1274.49	1281.35	1281.35	1274.06	1281.46	1281.46
$\omega_3(a^{'})$	732.53	758.45	732.58	732.58	746.45	732.76	732.76

Table S211: CMA-0A and CMA-2A (ξ = 0.02) harmonic frequencies targeting CCSD(T)/cc-pVTZ for Level B = B3LYP/6-31G(2*df*,*p*).

	Pure B3LYP/ 6-31G(2*df*,*p*)	CMA-0A B3LYP/ 6-31G(2*df*,*p*)	CMA-2A B3LYP/ 6-31G(2*df*,*p*)
$\omega_1(a^{'})$	3812.98	3809.59	3809.59
$\omega_2(a^{'})$	1287.50	1281.39	1281.39
$\omega_3(a^{'})$	749.15	732.54	732.54

Table S212: Symmetrized, unnormalized natural internal coordinates for hypochlorous acid.

1	$r_{1,2}$
2	$r_{2,3}$
3	$\phi_{1,2,3}$

S4.54 nitrosyl chloride

Table S213: CCSD(T)/cc-pVTZ Optimum Cartesian coordinates (bohr)

1	O	−2.97939346	0.63940304	0.00000000
2	N	−1.57525854	−0.99888693	0.00000000
3	Cl	1.99359145	0.10753257	0.00000000

Table S214: Reference [CCSD(T)/cc-pVTZ] CMA-0A and CMA-2A (ξ = 0.02) harmonic frequencies for Level B = MP2/cc-pVTZ and CCSD(T)/cc-pVDZ.

	Reference CCSD(T)/ cc-pVTZ	Pure MP2/ cc-pVTZ	CMA-0A MP2/ cc-pVTZ	CMA-2A MP2/ cc-pVTZ	Pure CCSD(T)/ cc-pVDZ	CMA-0A CCSD(T)/ cc-pVDZ	CMA-2A CCSD(T)/ cc-pVDZ
$\omega_1(a^{'})$	1828.88	1828.37	1828.84	1828.88	1914.93	1828.80	1828.88
$\omega_2(a^{'})$	609.86	618.02	609.86	609.86	625.61	609.88	609.86
$\omega_3(a^{'})$	342.84	350.17	343.05	342.84	353.67	343.25	342.84

Table S215: CMA-0A and CMA-2A (ξ = 0.02) harmonic frequencies targeting CCSD(T)/cc-pVTZ for Level B = B3LYP/6-31G(2*df*,*p*).

	Pure B3LYP/ 6-31G(2*df*,*p*)	CMA-0A B3LYP/ 6-31G(2*df*,*p*)	CMA-2A B3LYP/ 6-31G(2*df*,*p*)
$\omega_1(a^{'})$	1873.93	1828.87	1828.88
$\omega_2(a^{'})$	618.87	609.85	609.86
$\omega_3(a^{'})$	344.79	342.90	342.84

Table S216: Symmetrized, unnormalized natural internal coordinates for nitrosyl chloride.

1	$r_{1,2}$
2	$r_{2,3}$
3	$\phi_{1,2,3}$

S4.55 ozone

Table S217: CCSD(T)/cc-pVTZ Optimum Cartesian coordinates (bohr)

1	O	0.00000000	−2.05457709	0.42011174
2	O	0.00000000	0.00000000	−0.84022349
3	O	0.00000000	2.05457709	0.42011174

Table S218: Reference [CCSD(T)/cc-pVTZ] CMA-0A and CMA-2A ($\xi = 0.02$) harmonic frequencies for Level B = MP2/cc-pVTZ and CCSD(T)/cc-pVDZ.

	Reference CCSD(T)/ cc-pVTZ	Pure MP2/ cc-pVTZ	CMA-0A MP2/ cc-pVTZ	CMA-2A MP2/ cc-pVTZ	Pure CCSD(T)/ cc-pVDZ	CMA-0A CCSD(T)/ cc-pVDZ	CMA-2A CCSD(T)/ cc-pVDZ
$\omega_1(a_1)$	1153.11	1150.21	1153.11	1153.11	1137.09	1153.09	1153.09
$\omega_2(a_1)$	715.68	712.93	715.69	715.69	712.60	715.72	715.72
$\omega_3(b_2)$	1054.32	517.33	1054.32	1054.32	503.40	1054.32	1054.32

Table S219: CMA-0A and CMA-2A ($\xi = 0.02$) harmonic frequencies targeting CCSD(T)/cc-pVTZ for Level B = B3LYP/6-31G(2df,p).

	Pure B3LYP/ 6-31G(2df,p)	CMA-0A B3LYP/ 6-31G(2df,p)	CMA-2A B3LYP/ 6-31G(2df,p)
$\omega_1(a_1)$	1196.62	1153.06	1153.06
$\omega_2(a_1)$	721.26	715.76	715.76
$\omega_3(b_2)$	1124.68	1054.32	1054.32

Table S220: Symmetrized, unnormalized natural internal coordinates for ozone.

1	$r_{1,2} + r_{2,3}$
2	$r_{1,2} - r_{2,3}$
3	$\phi_{1,2,3}$

S4.56 oxygen difluoride

Table S221: CCSD(T)/cc-pVTZ Optimum Cartesian coordinates (bohr)

1	O	0.00000000	0.00000000	1.16502871
2	F	0.00000000	−2.08708826	−0.49042371
3	F	0.00000000	2.08708826	−0.49042371

Table S222: Reference [CCSD(T)/cc-pVTZ] CMA-0A and CMA-2A ($\xi = 0.02$) harmonic frequencies for Level B = MP2/cc-pVTZ and CCSD(T)/cc-pVDZ.

	Reference CCSD(T)/ cc-pVTZ	Pure MP2/ cc-pVTZ	CMA-0A MP2/ cc-pVTZ	CMA-2A MP2/ cc-pVTZ	Pure CCSD(T)/ cc-pVDZ	CMA-0A CCSD(T)/ cc-pVDZ	CMA-2A CCSD(T)/ cc-pVDZ
$\omega_1(a_1)$	950.11	959.11	950.08	950.11	932.97	950.10	950.10
$\omega_2(a_1)$	469.29	474.29	469.34	469.29	468.82	469.29	469.29
$\omega_3(b_2)$	867.42	888.05	867.42	867.42	844.25	867.42	867.42

Table S223: CMA-0A and CMA-2A ($\xi = 0.02$) harmonic frequencies targeting CCSD(T)/cc-pVTZ for Level B = B3LYP/6-31G(2*df*,*p*).

	Pure B3LYP/ 6-31G(2*df*,*p*)	CMA-0A B3LYP/ 6-31G(2*df*,*p*)	CMA-2A B3LYP/ 6-31G(2*df*,*p*)
$\omega_1(a_1)$	995.79	950.02	950.11
$\omega_2(a_1)$	473.56	469.46	469.29
$\omega_3(b_2)$	864.01	867.42	867.42

Table S224: Symmetrized, unnormalized natural internal coordinates for oxygen difluoride.

1	$r_{1,2} + r_{1,3}$
2	$r_{1,2} - r_{1,3}$
3	$\phi_{2,1,3}$

S4.57 water

Table S225: CCSD(T)/cc-pVTZ Optimum Cartesian coordinates (bohr)

1	O	0.00000000	0.00000000	0.12550454
2	H	0.00000000	−1.42462540	−0.99592409
3	H	0.00000000	1.42462540	−0.99592409

Table S226: Reference [CCSD(T)/cc-pVTZ] CMA-0A and CMA-2A ($\xi = 0.02$) harmonic frequencies for Level B = MP2/cc-pVTZ and CCSD(T)/cc-pVDZ.

	Reference CCSD(T)/ cc-pVTZ	Pure MP2/ cc-pVTZ	CMA-0A MP2/ cc-pVTZ	CMA-2A MP2/ cc-pVTZ	Pure CCSD(T)/ cc-pVDZ	CMA-0A CCSD(T)/ cc-pVDZ	CMA-2A CCSD(T)/ cc-pVDZ
$\omega_1(a_1)$	3840.92	3850.51	3840.92	3840.92	3907.61	3840.90	3840.90
$\omega_2(a_1)$	1668.87	1650.39	1668.88	1668.88	1684.91	1668.92	1668.92
$\omega_3(b_2)$	3945.53	3971.34	3945.53	3945.53	4027.33	3945.53	3945.53

Table S227: CMA-0A and CMA-2A ($\xi = 0.02$) harmonic frequencies targeting CCSD(T)/cc-pVTZ for Level B = B3LYP/6-31G(2*df*,*p*).

	Pure B3LYP/ 6-31G(2*df*,*p*)	CMA-0A B3LYP/ 6-31G(2*df*,*p*)	CMA-2A B3LYP/ 6-31G(2*df*,*p*)
$\omega_1(a_1)$	3846.06	3840.92	3840.92
$\omega_2(a_1)$	1662.84	1668.88	1668.88
$\omega_3(b_2)$	3951.91	3945.53	3945.53

Table S228: Symmetrized, unnormalized natural internal coordinates for water.

1	$r_{1,2} + r_{1,3}$
2	$r_{1,2} - r_{1,3}$
3	$\phi_{2,1,3}$

S4.58 trifluoroamine

Table S229: CCSD(T)/cc-pVTZ Optimum Cartesian coordinates (bohr)

1	N	0.92019321	0.00000000	−0.00000000
2	F	−0.22608099	1.16249337	−2.01349759
3	F	−0.22608099	−2.32498675	0.00000000
4	F	−0.22608099	1.16249337	2.01349759

Table S230: Reference [CCSD(T)/cc-pVTZ] CMA-0A and CMA-2A (ξ = 0.02) harmonic frequencies for Level B = MP2/cc-pVTZ and CCSD(T)/cc-pVDZ.

	Reference CCSD(T)/ cc-pVTZ	Pure MP2/ cc-pVTZ	CMA-0A MP2/ cc-pVTZ	CMA-2A MP2/ cc-pVTZ	Pure CCSD(T)/ cc-pVDZ	CMA-0A CCSD(T)/ cc-pVDZ	CMA-2A CCSD(T)/ cc-pVDZ
$\omega_1(a_1)$	1058.35	1059.49	1058.35	1058.35	1053.15	1058.26	1058.26
$\omega_2(a_1)$	659.91	666.00	659.91	659.91	659.84	660.05	660.05
$\omega_{3\mathrm{a}}(e)$	949.20	933.38	949.17	949.19	944.43	949.16	949.16
$\omega_{3\mathrm{b}}(e)$	949.20	933.31	949.17	949.19	944.36	949.16	949.16
$\omega_{4\mathrm{a}}(e)$	502.48	503.66	502.53	502.49	504.94	502.54	502.54
$\omega_{4\mathrm{b}}(e)$	502.48	503.58	502.53	502.49	504.86	502.54	502.54

Table S231: CMA-0A and CMA-2A (ξ = 0.02) harmonic frequencies targeting CCSD(T)/cc-pVTZ for Level B = B3LYP/6-31G(2*df*,*p*).

	Pure B3LYP/ 6-31G(2*df*,*p*)	CMA-0A B3LYP/ 6-31G(2*df*,*p*)	CMA-2A B3LYP/ 6-31G(2*df*,*p*)
$\omega_1(a_1)$	1068.00	1058.29	1058.29
$\omega_2(a_1)$	659.75	660.00	660.00
$\omega_{3\mathrm{a}}(e)$	934.15	949.16	949.19
$\omega_{3\mathrm{b}}(e)$	933.33	949.14	949.19
$\omega_{4\mathrm{a}}(e)$	497.64	502.59	502.50
$\omega_{4\mathrm{b}}(e)$	496.57	502.56	502.49

Table S232: Symmetrized, unnormalized natural internal coordinates for trifluoroamine.

1	$r_{1,2} + r_{1,3} + r_{1,4}$
2	$2r_{1,2} - r_{1,3} - r_{1,4}$
3	$r_{1,3} - r_{1,4}$
4	$2\phi_{2,1,3} - \phi_{2,1,4} - \phi_{3,1,4}$
5	$\phi_{2,1,4} - \phi_{3,1,4}$
6	$\gamma_{2,1,3,4} + \gamma_{3,1,4,2} + \gamma_{4,1,2,3}$

S4.59 chlorine trifluoride

Table S233: CCSD(T)/cc-pVTZ Optimum Cartesian coordinates (bohr)

1	Cl	0.00000000	0.00000000	0.69252140
2	F	0.00000000	0.00000000	−2.33980384
3	F	0.00000000	3.20469409	0.53256734
4	F	0.00000000	−3.20469409	0.53256734

Table S234: Reference [CCSD(T)/cc-pVTZ] CMA-0A and CMA-2A (ξ = 0.02) harmonic frequencies for Level B = MP2/cc-pVTZ and CCSD(T)/cc-pVDZ.

	Reference CCSD(T)/ cc-pVTZ	Pure MP2/ cc-pVTZ	CMA-0A MP2/ cc-pVTZ	CMA-2A MP2/ cc-pVTZ	Pure CCSD(T)/ cc-pVDZ	CMA-0A CCSD(T)/ cc-pVDZ	CMA-2A CCSD(T)/ cc-pVDZ
$\omega_1(a_1)$	765.58	786.73	765.43	765.46	809.77	765.12	765.12
$\omega_2(a_1)$	543.99	556.90	544.15	544.15	573.59	544.47	544.47
$\omega_3(a_1)$	338.43	336.01	338.51	338.45	346.02	338.69	338.69
$\omega_4(b_1)$	336.96	338.12	336.96	336.96	341.24	336.96	336.96
$\omega_5(b_2)$	735.59	770.63	735.59	735.59	806.50	735.54	735.54
$\omega_6(b_2)$	441.96	444.84	441.96	441.96	444.12	442.05	442.05

Table S235: CMA-0A and CMA-2A (ξ = 0.02) harmonic frequencies targeting CCSD(T)/cc-pVTZ for Level B = B3LYP/6-31G(2*df*,*p*).

	Pure B3LYP/ 6-31G(2*df*,*p*)	CMA-0A B3LYP/ 6-31G(2*df*,*p*)	CMA-2A B3LYP/ 6-31G(2*df*,*p*)
$\omega_1(a_1)$	789.56	765.57	765.57
$\omega_2(a_1)$	551.29	543.98	543.99
$\omega_3(a_1)$	330.68	338.46	338.44
$\omega_4(b_1)$	329.76	336.96	336.96
$\omega_5(b_2)$	756.84	735.59	735.59
$\omega_6(b_2)$	437.61	441.96	441.96

Table S236: Symmetrized, unnormalized natural internal coordinates for chlorine trifluoride.

1	$r_{1,2}$
2	$r_{1,3} + r_{1,4}$
3	$r_{1,3} - r_{1,4}$
4	$\phi_{3,1,2} + \phi_{4,1,2}$
5	$\phi_{3,1,2} - \phi_{4,1,2}$
6	$\gamma_{2,1,3,4}$

S4.60 hydrogen peroxide

Table S237: CCSD(T)/cc-pVTZ Optimum Cartesian coordinates (bohr)

1	H	−1.79701451	1.36358961	0.92174870
2	O	−1.37290862	−0.11245968	−0.05807855
3	O	1.37290862	0.11245968	−0.05807855
4	H	1.79701451	−1.36358961	0.92174870

Table S238: Reference [CCSD(T)/cc-pVTZ] CMA-0A and CMA-2A (ξ = 0.02) harmonic frequencies for Level B = MP2/cc-pVTZ and CCSD(T)/cc-pVDZ.

	Reference CCSD(T)/ cc-pVTZ	Pure MP2/ cc-pVTZ	CMA-0A MP2/ cc-pVTZ	CMA-2A MP2/ cc-pVTZ	Pure CCSD(T)/ cc-pVDZ	CMA-0A CCSD(T)/ cc-pVDZ	CMA-2A CCSD(T)/ cc-pVDZ
$\omega_1(a)$	3809.29	3826.73	3809.25	3809.28	3879.42	3809.27	3809.27
$\omega_2(a)$	1435.98	1426.62	1435.66	1436.00	1447.14	1435.88	1436.02
$\omega_3(a)$	911.79	924.21	912.43	911.79	891.84	911.86	911.80
$\omega_4(a)$	372.17	370.94	372.26	372.18	367.80	372.56	372.17
$\omega_5(b)$	3807.91	3825.46	3807.91	3807.91	3876.94	3807.87	3807.87
$\omega_6(b)$	1323.64	1319.01	1323.64	1323.64	1324.98	1323.74	1323.74

Table S239: CMA-0A and CMA-2A (ξ = 0.02) harmonic frequencies targeting CCSD(T)/cc-pVTZ for Level B = B3LYP/6-31G(2*df*,*p*).

	Pure B3LYP/ 6-31G(2*df*,*p*)	CMA-0A B3LYP/ 6-31G(2*df*,*p*)	CMA-2A B3LYP/ 6-31G(2*df*,*p*)
$\omega_1(a)$	3811.77	3809.26	3809.27
$\omega_2(a)$	1447.63	1435.97	1436.00
$\omega_3(a)$	931.55	911.89	911.85
$\omega_4(a)$	382.09	372.19	372.17
$\omega_5(b)$	3811.23	3807.90	3807.90
$\omega_6(b)$	1333.31	1323.65	1323.65

Table S240: Symmetrized, unnormalized natural internal coordinates for hydrogen peroxide.

1	$r_{2,3}$
2	$r_{1,2} + r_{3,4}$
3	$r_{1,2} - r_{3,4}$
4	$\phi_{1,2,3} + \phi_{2,3,4}$
5	$\phi_{1,2,3} - \phi_{2,3,4}$
6	$\tau_{1,2,3,4}$

S4.61 carbonyl fluoride

Table S241: CCSD(T)/cc-pVTZ Optimum Cartesian coordinates (bohr)

1	C	−0.30295787	−0.00000001	0.00000000
2	F	1.15873701	−2.00577068	0.00000000
3	F	1.15873683	2.00577078	0.00000000
4	O	−2.52535314	−0.00000011	−0.00000000

Table S242: Reference [CCSD(T)/cc-pVTZ] CMA-0A and CMA-2A (ξ = 0.02) harmonic frequencies for Level B = MP2/cc-pVTZ and CCSD(T)/cc-pVDZ.

	Reference CCSD(T)/ cc-pVTZ	Pure MP2/ cc-pVTZ	CMA-0A MP2/ cc-pVTZ	CMA-2A MP2/ cc-pVTZ	Pure CCSD(T)/ cc-pVDZ	CMA-0A CCSD(T)/ cc-pVDZ	CMA-2A CCSD(T)/ cc-pVDZ
$\omega_1(a_1)$	1978.03	1979.81	1978.03	1978.03	2043.85	1978.03	1978.03
$\omega_2(a_1)$	983.53	982.88	983.52	983.52	999.40	983.54	983.53
$\omega_3(a_1)$	588.74	590.43	588.74	588.74	594.82	588.74	588.74
$\omega_4(b_1)$	1293.99	1281.37	1293.99	1293.99	1341.32	1293.99	1293.99
$\omega_5(b_1)$	626.11	626.93	626.11	626.11	633.00	626.11	626.11
$\omega_6(b_2)$	786.02	791.18	786.02	786.02	793.61	786.02	786.02

Table S243: CMA-0A and CMA-2A (ξ = 0.02) harmonic frequencies targeting CCSD(T)/cc-pVTZ for Level B = B3LYP/6-31G(2*df*,*p*).

	Pure B3LYP/ 6-31G(2*df*,*p*)	CMA-0A B3LYP/ 6-31G(2*df*,*p*)	CMA-2A B3LYP/ 6-31G(2*df*,*p*)
$\omega_1(a_1)$	1992.05	1978.02	1978.02
$\omega_2(a_1)$	992.27	983.54	983.54
$\omega_3(a_1)$	584.92	588.74	588.74
$\omega_4(b_1)$	1290.74	1293.99	1293.99
$\omega_5(b_1)$	620.20	626.11	626.11
$\omega_6(b_2)$	789.97	786.02	786.02

Table S244: Symmetrized, unnormalized natural internal coordinates for carbonyl fluoride.

1	$r_{1,2} + r_{1,3}$
2	$r_{1,2} - r_{1,3}$
3	$r_{1,4}$
4	$2\phi_{2,1,3} - \phi_{2,1,4} - \phi_{3,1,4}$
5	$\phi_{2,1,4} - \phi_{3,1,4}$
6	$\gamma_{4,1,2,3}$

S4.62 singlet silylene

Table S245: CCSD(T)/cc-pVTZ Optimum Cartesian coordinates (bohr)

1	Si	0.00000000	0.00000000	0.13352287
2	H	0.00000000	−2.07118886	−1.85327784
3	H	0.00000000	2.07118886	−1.85327784

Table S246: Reference [CCSD(T)/cc-pVTZ] CMA-0A and CMA-2A ($\xi = 0.02$) harmonic frequencies for Level B = MP2/cc-pVTZ and CCSD(T)/cc-pVDZ.

	Reference CCSD(T)/ cc-pVTZ	Pure MP2/ cc-pVTZ	CMA-0A MP2/ cc-pVTZ	CMA-2A MP2/ cc-pVTZ	Pure CCSD(T)/ cc-pVDZ	CMA-0A CCSD(T)/ cc-pVDZ	CMA-2A CCSD(T)/ cc-pVDZ
$\omega_1(a_1)$	2063.17	2101.76	2063.17	2063.17	2100.30	2063.17	2063.17
$\omega_2(a_1)$	1025.33	1044.38	1025.33	1025.33	1043.32	1025.33	1025.33
$\omega_3(b_2)$	2060.52	2100.71	2060.52	2060.52	2097.94	2060.52	2060.52

Table S247: CMA-0A and CMA-2A ($\xi = 0.02$) harmonic frequencies targeting CCSD(T)/cc-pVTZ for Level B = B3LYP/6-31G(2*df*,*p*).

	Pure B3LYP/ 6-31G(2*df*,*p*)	CMA-0A B3LYP/ 6-31G(2*df*,*p*)	CMA-2A B3LYP/ 6-31G(2*df*,*p*)
$\omega_1(a_1)$	2076.64	2063.13	2063.13
$\omega_2(a_1)$	1027.10	1025.39	1025.39
$\omega_3(b_2)$	2083.14	2060.52	2060.52

Table S248: Symmetrized, unnormalized natural internal coordinates for singlet silylene.

1	$r_{1,2} + r_{1,3}$
2	$r_{1,2} - r_{1,3}$
3	$\phi_{2,1,3}$

S4.63 nitrous oxide

Table S249: CCSD(T)/cc-pVTZ Optimum Cartesian coordinates (bohr)

1	N	−0.00000000	−0.00000000	−2.27661556
2	X	1.00000000	−0.00000000	−0.13588985
3	N	−0.00000000	−0.00000000	−0.13588985
4	X	−0.00000000	1.00000000	−0.13588985
5	O	0.00000000	0.00000000	2.11207702

Table S250: Reference [CCSD(T)/cc-pVTZ] CMA-0A and CMA-2A (ξ = 0.02) harmonic frequencies for Level B = MP2/cc-pVTZ and CCSD(T)/cc-pVDZ.

	Reference CCSD(T)/ cc-pVTZ	Pure MP2/ cc-pVTZ	CMA-0A MP2/ cc-pVTZ	CMA-2A MP2/ cc-pVTZ	Pure CCSD(T)/ cc-pVDZ	CMA-0A CCSD(T)/ cc-pVDZ	CMA-2A CCSD(T)/ cc-pVDZ
$\omega_1(\sigma^+)$	2282.57	2307.73	2277.23	2282.57	2398.87	2282.51	2282.57
$\omega_{2\mathrm{a}}(\pi)$	601.18	607.31	601.18	601.18	601.03	601.18	601.18
$\omega_{2\mathrm{b}}(\pi)$	601.18	607.30	601.18	601.18	601.02	601.18	601.18
$\omega_3(\sigma^+)$	1297.09	1322.29	1306.43	1297.09	1349.83	1297.19	1297.09

Table S251: CMA-0A and CMA-2A (ξ = 0.02) harmonic frequencies targeting CCSD(T)/cc-pVTZ for Level B = B3LYP/6-31G(2df,p).

	Pure B3LYP/ 6-31G(2df,p)	CMA-0A B3LYP/ 6-31G(2df,p)	CMA-2A B3LYP/ 6-31G(2df,p)
$\omega_1(\sigma^+)$	2316.98	2282.42	2282.57
$\omega_{2\mathrm{a}}(\pi)$	617.71	601.18	601.18
$\omega_{2\mathrm{b}}(\pi)$	617.71	601.18	601.18
$\omega_3(\sigma^+)$	1316.53	1297.36	1297.09

Table S252: Symmetrized, unnormalized natural internal coordinates for nitrous oxide.

1	$r_{1,3}$
2	$r_{3,5}$
3	$\theta_{1,3,5,2}$
4	$\theta_{1,3,5,4}$

S4.64 hydrazine

Table S253: CCSD(T)/cc-pVTZ Optimum Cartesian coordinates (bohr)

1	N	−0.17939083	−1.39259049	0.00000000
2	N	0.17939083	1.39259049	0.00000000
3	H	0.87854704	−1.97545387	−1.49671921
4	H	0.87854704	−1.97545387	1.49671921
5	H	−0.87854704	1.97545387	−1.49671921
6	H	−0.87854704	1.97545387	1.49671921

Table S254: Reference [CCSD(T)/cc-pVTZ] CMA-0A and CMA-2A ($\xi = 0.02$) harmonic frequencies for Level B = MP2/cc-pVTZ and CCSD(T)/cc-pVDZ.

	Reference CCSD(T)/ cc-pVTZ	Pure MP2/ cc-pVTZ	CMA-0A MP2/ cc-pVTZ	CMA-2A MP2/ cc-pVTZ	Pure CCSD(T)/ cc-pVDZ	CMA-0A CCSD(T)/ cc-pVDZ	CMA-2A CCSD(T)/ cc-pVDZ
$\omega_1(a_g)$	3437.77	3454.45	3437.77	3437.77	3542.36	3437.64	3437.64
$\omega_2(a_g)$	1690.46	1684.69	1690.44	1690.44	1714.86	1690.44	1690.44
$\omega_3(a_g)$	1256.79	1243.43	1256.36	1256.80	1275.27	1256.91	1257.04
$\omega_4(a_g)$	943.78	942.12	944.42	943.82	956.33	944.14	943.97
$\omega_5(b_g)$	3512.91	3541.31	3512.91	3512.91	3630.15	3512.91	3512.91
$\omega_6(b_g)$	1487.98	1479.54	1487.98	1487.98	1484.81	1487.98	1487.98
$\omega_7(a_u)$	3533.97	3561.31	3533.97	3533.97	3649.00	3533.96	3533.96
$\omega_8(a_u)$	1108.51	1106.06	1108.50	1108.51	1100.74	1108.53	1108.53
$\omega_9(a_u)$	25.68	37.10	26.29	25.68	159.73	25.92	25.92
$\omega_{10}(b_u)$	3455.35	3472.41	3455.35	3455.35	3556.61	3455.25	3455.25
$\omega_{11}(b_u)$	1638.68	1630.83	1638.66	1638.68	1661.86	1638.74	1638.76
$\omega_{12}(b_u)$	1068.37	1059.20	1068.40	1068.37	1086.82	1068.61	1068.58

Table S255: CMA-0A and CMA-2A ($\xi = 0.02$) harmonic frequencies targeting CCSD(T)/cc-pVTZ for Level B = B3LYP/6-31G(2*df*,*p*).

	Pure B3LYP/ 6-31G(2*df*,*p*)	CMA-0A B3LYP/ 6-31G(2*df*,*p*)	CMA-2A B3LYP/ 6-31G(2*df*,*p*)
$\omega_1(a_g)$	3455.22	3437.75	3437.75
$\omega_2(a_g)$	1692.86	1690.41	1690.41
$\omega_3(a_g)$	1257.61	1256.86	1256.87
$\omega_4(a_g)$	950.45	943.87	943.86
$\omega_5(b_g)$	3525.66	3512.88	3512.88
$\omega_6(b_g)$	1501.96	1488.03	1488.03
$\omega_7(a_u)$	3546.71	3533.96	3533.96
$\omega_8(a_u)$	1126.46	1108.46	1108.53
$\omega_9(a_u)$	95.04	29.03	25.69
$\omega_{10}(b_u)$	3473.45	3455.35	3455.35
$\omega_{11}(b_u)$	1635.11	1638.64	1638.68
$\omega_{12}(b_u)$	1073.18	1068.44	1068.37

Table S256: Symmetrized, unnormalized natural internal coordinates for hydrazine.

1	$r_{1,2}$
2	$r_{1,3} + r_{1,4} + r_{2,5} + r_{2,6}$
3	$r_{1,3} + r_{1,4} - r_{2,5} - r_{2,6}$
4	$r_{1,3} - r_{1,4} + r_{2,5} - r_{2,6}$
5	$r_{1,3} - r_{1,4} - r_{2,5} + r_{2,6}$
6	$2\phi_{3,1,4} - \phi_{3,1,2} - \phi_{4,1,2} + 2\phi_{5,2,6} - \phi_{5,2,1} - \phi_{6,2,1}$
7	$2\phi_{3,1,4} - \phi_{3,1,2} - \phi_{4,1,2} - 2\phi_{5,2,6} + \phi_{5,2,1} + \phi_{6,2,1}$
8	$\phi_{3,1,2} - \phi_{4,1,2} + \phi_{5,2,1} - \phi_{6,2,1}$
9	$\phi_{3,1,2} - \phi_{4,1,2} - \phi_{5,2,1} + \phi_{6,2,1}$
10	$\tau_{3,1,2,5} + \tau_{3,1,2,6} + \tau_{4,1,2,5} + \tau_{4,1,2,6}$
11	$\gamma_{1,2,5,6} + \gamma_{2,1,3,4}$
12	$\gamma_{1,2,5,6} - \gamma_{2,1,3,4}$

S4.65 cyanogen

Table S257: CCSD(T)/cc-pVTZ Optimum Cartesian coordinates (bohr)

1	X	1.00000000	0.00000000	1.31251829
2	C	−0.00000000	0.00000000	1.31251829
3	X	−0.00000000	1.00000000	1.31251829
4	X	1.00000000	0.00000000	−1.31251829
5	C	0.00000000	0.00000000	−1.31251829
6	X	0.00000000	1.00000000	−1.31251829
7	N	0.00000000	0.00000000	3.51379022
8	N	−0.00000000	0.00000000	−3.51379022

Table S258: Reference [CCSD(T)/cc-pVTZ] CMA-0A and CMA-2A (ξ = 0.02) harmonic frequencies for Level B = MP2/cc-pVTZ and CCSD(T)/cc-pVDZ.

	Reference CCSD(T)/ cc-pVTZ	Pure MP2/ cc-pVTZ	CMA-0A MP2/ cc-pVTZ	CMA-2A MP2/ cc-pVTZ	Pure CCSD(T)/ cc-pVDZ	CMA-0A CCSD(T)/ cc-pVDZ	CMA-2A CCSD(T)/ cc-pVDZ
$\omega_1(\sigma_g^+)$	2367.74	2309.29	2367.72	2367.72	2477.71	2367.74	2367.74
$\omega_2(\sigma_g^+)$	855.82	848.47	855.89	855.89	897.23	855.83	855.83
$\omega_{3a}(\pi_u)$	236.26	240.70	236.26	236.26	241.41	236.26	236.26
$\omega_{3b}(\pi_u)$	236.26	240.69	236.26	236.26	241.41	236.26	236.26
$\omega_4(\sigma_u^+)$	2175.85	2131.38	2175.85	2175.85	2269.57	2175.85	2175.85
$\omega_{5a}(\pi_g)$	498.61	510.83	498.61	498.61	518.28	498.61	498.61
$\omega_{5b}(\pi_g)$	498.61	510.82	498.61	498.61	518.27	498.61	498.61

Table S259: CMA-0A and CMA-2A (ξ = 0.02) harmonic frequencies targeting CCSD(T)/cc-pVTZ for Level B = B3LYP/6-31G(2*df*,*p*).

	Pure B3LYP/ 6-31G(2*df*,*p*)	CMA-0A B3LYP/ 6-31G(2*df*,*p*)	CMA-2A B3LYP/ 6-31G(2*df*,*p*)
$\omega_1(\sigma_g^+)$	2375.39	2367.56	2367.74
$\omega_2(\sigma_g^+)$	856.77	856.32	855.82
$\omega_{3a}(\pi_u)$	247.05	236.26	236.26
$\omega_{3b}(\pi_u)$	2218.85	2175.85	2175.85
$\omega_4(\sigma_u^+)$	247.04	236.26	236.26
$\omega_{5a}(\pi_g)$	544.15	498.61	498.61
$\omega_{5b}(\pi_g)$	544.12	498.61	498.61

Table S260: Symmetrized, unnormalized natural internal coordinates for cyanogen.

1	$r_{2,5}$
2	$r_{2,7} + r_{5,8}$
3	$r_{2,7} - r_{5,8}$
4	$\theta_{7,2,5,1} + \theta_{8,5,2,4}$
5	$\theta_{7,2,5,1} - \theta_{8,5,2,4}$
6	$\theta_{7,2,5,3} + \theta_{8,5,2,6}$
7	$\theta_{7,2,5,3} - \theta_{8,5,2,6}$

S4.66 aziridine

Table S261: CCSD(T)/cc-pVTZ Optimum Cartesian coordinates (bohr)

1	N	1.59582703	0.17617984	0.00000000
2	C	−0.81575373	−0.02639125	1.40146541
3	C	−0.81575373	−0.02639125	−1.40146541
4	H	−1.14815063	−1.78855443	2.38563323
5	H	−1.43745953	1.65562797	2.38563323
6	H	−1.43745953	1.65562797	−2.38563323
7	H	−1.14815063	−1.78855443	−2.38563323
8	H	2.42432052	−1.55357917	0.00000000

Table S262: Reference [CCSD(T)/cc-pVTZ] CMA-0A and CMA-2A (ξ = 0.02) harmonic frequencies for Level B = MP2/cc-pVTZ and CCSD(T)/cc-pVDZ.

	Reference CCSD(T)/ cc-pVTZ	Pure MP2/ cc-pVTZ	CMA-0A MP2/ cc-pVTZ	CMA-2A MP2/ cc-pVTZ	Pure CCSD(T)/ cc-pVDZ	CMA-0A CCSD(T)/ cc-pVDZ	CMA-2A CCSD(T)/ cc-pVDZ
$\omega_1(a^{'})$	3515.02	3536.09	3515.01	3515.01	3625.84	3514.99	3514.99
$\omega_2(a^{'})$	3228.03	3250.96	3227.98	3227.98	3376.83	3227.98	3227.98
$\omega_3(a^{'})$	3137.27	3152.69	3137.24	3137.24	3274.70	3137.24	3137.24
$\omega_4(a^{'})$	1532.65	1530.55	1532.55	1532.55	1557.18	1531.84	1531.84
$\omega_5(a^{'})$	1307.58	1302.11	1306.88	1307.48	1321.75	1307.21	1307.52
$\omega_6(a^{'})$	1245.94	1244.31	1246.55	1246.00	1282.83	1246.89	1246.68
$\omega_7(a^{'})$	1120.20	1108.14	1119.96	1120.36	1139.43	1119.38	1119.70
$\omega_8(a^{'})$	1022.46	1025.72	1022.57	1022.67	1036.80	1023.01	1022.75
$\omega_9(a^{'})$	874.83	876.97	874.09	873.93	905.40	875.94	875.75
$\omega_{10}(a^{'})$	782.13	773.27	783.90	783.24	785.15	782.52	782.39
$\omega_{11}(a^{''})$	3214.74	3238.40	3214.69	3214.69	3363.33	3214.69	3214.69
$\omega_{12}(a^{''})$	3130.26	3146.66	3130.23	3130.23	3266.05	3130.22	3130.22
$\omega_{13}(a^{''})$	1501.76	1503.54	1501.67	1501.67	1515.71	1501.65	1501.65
$\omega_{14}(a^{''})$	1272.26	1260.66	1271.62	1271.82	1282.46	1271.91	1272.01
$\omega_{15}(a^{''})$	1162.60	1164.93	1162.56	1162.84	1178.15	1161.87	1161.87
$\omega_{16}(a^{''})$	1113.59	1101.91	1113.60	1113.60	1120.49	1114.33	1114.33
$\omega_{17}(a^{''})$	923.43	918.64	924.21	924.18	924.13	923.68	923.75
$\omega_{18}(a^{''})$	840.79	842.60	841.47	840.81	871.35	841.59	841.37

Table S263: CMA-0A and CMA-2A (ξ = 0.02) harmonic frequencies targeting CCSD(T)/cc-pVTZ for Level B = B3LYP/6-31G(2df,p).

	Pure B3LYP/ 6-31G(2df,p)	CMA-0A B3LYP/ 6-31G(2df,p)	CMA-2A B3LYP/ 6-31G(2df,p)
$\omega_1(a^{'})$	3527.86	3515.00	3515.00
$\omega_2(a^{'})$	3243.74	3227.98	3227.98
$\omega_3(a^{'})$	3149.07	3137.20	3137.20
$\omega_4(a^{'})$	1522.33	1532.46	1532.46
$\omega_5(a^{'})$	1310.14	1307.26	1307.44
$\omega_6(a^{'})$	1246.86	1246.14	1246.00
$\omega_7(a^{'})$	1115.72	1120.28	1120.62
$\omega_8(a^{'})$	1028.85	1022.80	1022.66
$\omega_9(a^{'})$	872.94	873.94	873.89
$\omega_{10}(a^{'})$	770.07	783.75	783.42
$\omega_{11}(a^{''})$	3228.51	3214.68	3214.68
$\omega_{12}(a^{''})$	3143.34	3130.21	3130.21
$\omega_{13}(a^{''})$	1495.66	1501.61	1501.61
$\omega_{14}(a^{''})$	1298.46	1271.02	1271.02
$\omega_{15}(a^{''})$	1165.59	1161.65	1161.65
$\omega_{16}(a^{''})$	1110.54	1114.97	1114.97
$\omega_{17}(a^{''})$	927.85	923.59	924.91
$\omega_{18}(a^{''})$	843.99	842.67	841.22

Table S264: Symmetrized, unnormalized natural internal coordinates for aziridine.

1	$r_{1,8}$
2	$r_{2,3} + r_{1,2} + r_{1,3}$
3	$2r_{2,3} - r_{1,2} - r_{1,3}$
4	$r_{1,2} - r_{1,3}$
5	$r_{2,4} + r_{2,5} + r_{3,6} + r_{3,7}$
6	$r_{2,4} + r_{2,5} - r_{3,6} - r_{3,7}$
7	$r_{2,4} - r_{2,5} - r_{3,6} + r_{3,7}$
8	$r_{2,4} - r_{2,5} + r_{3,6} - r_{3,7}$
9	$\phi_{8,1,2} - \phi_{8,1,3}$
10	$4\phi_{4,2,5} - \phi_{4,2,1} - \phi_{4,2,3} - \phi_{5,2,1} - \phi_{5,2,3} + 4\phi_{6,3,7} - \phi_{6,3,1} - \phi_{6,3,2} - \phi_{7,3,1} - \phi_{7,3,2}$
11	$4\phi_{4,2,5} - \phi_{4,2,1} - \phi_{4,2,3} - \phi_{5,2,1} - \phi_{5,2,3} - 4\phi_{6,3,7} + \phi_{6,3,1} + \phi_{6,3,2} + \phi_{7,3,1} + \phi_{7,3,2}$
12	$\phi_{4,2,1} - \phi_{4,2,3} + \phi_{5,2,1} - \phi_{5,2,3} + \phi_{6,3,1} - \phi_{6,3,2} + \phi_{7,3,1} - \phi_{7,3,2}$
13	$\phi_{4,2,1} - \phi_{4,2,3} + \phi_{5,2,1} - \phi_{5,2,3} - \phi_{6,3,1} + \phi_{6,3,2} - \phi_{7,3,1} + \phi_{7,3,2}$
14	$\phi_{4,2,1} + \phi_{4,2,3} - \phi_{5,2,1} - \phi_{5,2,3} + \phi_{6,3,1} + \phi_{6,3,2} - \phi_{7,3,1} - \phi_{7,3,2}$
15	$\phi_{4,2,1} + \phi_{4,2,3} - \phi_{5,2,1} - \phi_{5,2,3} - \phi_{6,3,1} - \phi_{6,3,2} + \phi_{7,3,1} + \phi_{7,3,2}$
16	$\phi_{4,2,1} - \phi_{4,2,3} - \phi_{5,2,1} + \phi_{5,2,3} + \phi_{6,3,1} - \phi_{6,3,2} - \phi_{7,3,1} + \phi_{7,3,2}$
17	$\phi_{4,2,1} - \phi_{4,2,3} - \phi_{5,2,1} + \phi_{5,2,3} - \phi_{6,3,1} + \phi_{6,3,2} + \phi_{7,3,1} - \phi_{7,3,2}$
18	$\gamma_{8,1,2,3}$

S4.67 acetamide

Table S265: CCSD(T)/cc-pVTZ Optimum Cartesian coordinates (bohr)

1	C	2.71257408	0.46659407	−0.00029896
2	C	−0.08279841	−0.16544287	0.00691252
3	O	−0.87869630	−2.32459739	−0.00775877
4	N	−1.66151859	1.89377014	0.05553476
5	H	3.75222131	−1.12020941	0.78819940
6	H	3.33318276	0.75461502	−1.94795633
7	H	3.11796536	2.17773791	1.07525063
8	H	−0.98387343	3.61876868	−0.34837186
9	H	−3.50051514	1.56367391	−0.29435040

Table S266: Reference [CCSD(T)/cc-pVTZ] CMA-0A and CMA-2A ($\xi = 0.02$) harmonic frequencies for Level B = MP2/cc-pVTZ and CCSD(T)/cc-pVDZ.

	Reference CCSD(T)/ cc-pVTZ	Pure MP2/ cc-pVTZ	CMA-0A MP2/ cc-pVTZ	CMA-2A MP2/ cc-pVTZ	Pure CCSD(T)/ cc-pVDZ	CMA-0A CCSD(T)/ cc-pVDZ	CMA-2A CCSD(T)/ cc-pVDZ
$\omega_1(a)$	3738.95	3755.73	3738.94	3738.94	3857.47	3738.86	3738.86
$\omega_2(a)$	3604.29	3612.65	3604.30	3604.30	3711.50	3604.37	3604.37
$\omega_3(a)$	3165.52	3187.23	3165.47	3165.47	3314.78	3165.43	3165.43
$\omega_4(a)$	3122.95	3146.22	3122.92	3122.92	3271.78	3122.83	3122.83
$\omega_5(a)$	3050.62	3063.10	3050.69	3050.69	3182.22	3050.75	3050.75
$\omega_6(a)$	1794.31	1797.06	1794.24	1794.30	1855.86	1793.90	1794.12
$\omega_7(a)$	1628.26	1622.06	1628.25	1628.25	1644.42	1627.37	1627.37
$\omega_8(a)$	1495.75	1498.57	1495.74	1495.74	1507.55	1495.53	1495.53
$\omega_9(a)$	1480.84	1482.47	1480.80	1480.80	1493.04	1480.63	1480.64
$\omega_{10}(a)$	1409.87	1401.90	1409.96	1409.92	1439.24	1409.04	1410.51
$\omega_{11}(a)$	1344.17	1338.81	1344.18	1344.18	1368.98	1346.33	1344.85
$\omega_{12}(a)$	1134.12	1130.72	1134.15	1134.12	1148.54	1134.28	1134.24
$\omega_{13}(a)$	1057.36	1053.87	1057.38	1057.38	1065.20	1057.37	1057.37
$\omega_{14}(a)$	981.94	978.59	981.96	981.97	997.79	982.61	982.29
$\omega_{15}(a)$	854.16	852.39	854.18	854.16	881.65	854.48	854.41
$\omega_{16}(a)$	638.47	642.37	638.42	638.48	649.31	638.46	638.52
$\omega_{17}(a)$	553.53	554.20	553.53	553.52	562.91	553.52	553.52
$\omega_{18}(a)$	508.36	510.84	508.41	508.35	513.64	508.46	508.43
$\omega_{19}(a)$	416.23	416.62	416.22	416.22	423.34	416.09	416.10
$\omega_{20}(a)$	300.01	321.99	300.20	300.11	230.71	300.83	300.89
$\omega_{21}(a)$	56.09	57.06	56.31	56.16	61.69	58.20	56.21

Table S267: CMA-0A and CMA-2A (ξ = 0.02) harmonic frequencies targeting CCSD(T)/cc-pVTZ for Level B = B3LYP/6-31G(2df,p).

	Pure B3LYP/ 6-31G(2df,p)	CMA-0A B3LYP/ 6-31G(2df,p)	CMA-2A B3LYP/ 6-31G(2df,p)
$\omega_1(a)$	3753.21	3738.93	3738.93
$\omega_2(a)$	3617.22	3604.30	3604.30
$\omega_3(a)$	3182.64	3165.48	3165.48
$\omega_4(a)$	3136.79	3122.94	3122.94
$\omega_5(a)$	3068.92	3050.62	3050.62
$\omega_6(a)$	1798.52	1794.17	1794.22
$\omega_7(a)$	1616.70	1628.24	1628.26
$\omega_8(a)$	1493.63	1495.67	1495.67
$\omega_9(a)$	1477.07	1480.60	1480.60
$\omega_{10}(a)$	1404.68	1408.73	1408.68
$\omega_{11}(a)$	1341.71	1345.68	1345.71
$\omega_{12}(a)$	1135.91	1134.11	1134.10
$\omega_{13}(a)$	1057.65	1057.39	1057.42
$\omega_{14}(a)$	985.46	982.06	982.02
$\omega_{15}(a)$	850.53	854.47	854.46
$\omega_{16}(a)$	647.69	638.08	638.11
$\omega_{17}(a)$	554.11	553.55	553.53
$\omega_{18}(a)$	508.38	508.10	508.39
$\omega_{19}(a)$	422.20	416.05	416.32
$\omega_{20}(a)$	365.62	301.97	300.98
$\omega_{21}(a)$	46.94	56.86	56.20

Table S268: Symmetrized, unnormalized natural internal coordinates for acetamide.

1	$r_{1,2}$
2	$r_{2,3}$
3	$r_{2,4}$
4	$r_{1,5} + r_{1,6} + r_{1,7}$
5	$2r_{1,5} - r_{1,6} - r_{1,7}$
6	$r_{1,6} - r_{1,7}$
7	$r_{4,8} + r_{4,9}$
8	$r_{4,8} - r_{4,9}$
9	$2\phi_{1,2,4} - \phi_{1,2,3} - \phi_{4,2,3}$
10	$\phi_{1,2,3} - \phi_{4,2,3}$
11	$\phi_{5,1,2} + \phi_{6,1,2} + \phi_{7,1,2} - \phi_{6,1,7} - \phi_{5,1,6} - \phi_{5,1,7}$
12	$2\phi_{5,1,2} - \phi_{6,1,2} - \phi_{7,1,2}$
13	$\phi_{6,1,2} - \phi_{7,1,2}$
14	$2\phi_{6,1,7} - \phi_{5,1,6} - \phi_{5,1,7}$
15	$\phi_{5,1,6} - \phi_{5,1,7}$
16	$2\phi_{8,4,9} - \phi_{8,4,2} - \phi_{9,4,2}$
17	$\phi_{8,4,2} - \phi_{9,4,2}$
18	$\tau_{5,1,2,3} + \tau_{5,1,2,4} + \tau_{6,1,2,3} + \tau_{6,1,2,4} + \tau_{7,1,2,3} + \tau_{7,1,2,4}$
19	$\tau_{8,4,2,1} + \tau_{8,4,2,3} + \tau_{9,4,2,1} + \tau_{9,4,2,3}$
20	$\gamma_{3,2,1,4}$
21	$\gamma_{2,4,8,9}$

S4.68 dimethylamine

Table S269: CCSD(T)/cc-pVTZ Optimum Cartesian coordinates (bohr)

1	N	1.05866364	−0.16314434	0.00000000
2	H	2.32232113	1.27592347	−0.00000000
3	C	−0.48856083	0.03001809	2.27606500
4	C	−0.48856083	0.03001809	−2.27606500
5	H	0.71957620	0.01962878	3.94620566
6	H	−1.73337996	−1.61370927	2.38661331
7	H	−1.68486837	1.73209079	2.33361602
8	H	0.71957620	0.01962878	−3.94620566
9	H	−1.73337996	−1.61370927	−2.38661331
10	H	−1.68486837	1.73209079	−2.33361602

Table S270: Reference [CCSD(T)/cc-pVTZ] CMA-0A and CMA-2A (ξ = 0.02) harmonic frequencies for Level B = MP2/cc-pVTZ and CCSD(T)/cc-pVDZ.

	Reference CCSD(T)/ cc-pVTZ	Pure MP2/ cc-pVTZ	CMA-0A MP2/ cc-pVTZ	CMA-2A MP2/ cc-pVTZ	Pure CCSD(T)/ cc-pVDZ	CMA-0A CCSD(T)/ cc-pVDZ	CMA-2A CCSD(T)/ cc-pVDZ
$\omega_1(a^{'})$	3524.72	3545.68	3524.71	3524.71	3634.19	3524.71	3524.71
$\omega_2(a^{'})$	3113.81	3138.71	3113.77	3113.77	3263.68	3113.70	3113.70
$\omega_3(a^{'})$	3065.27	3085.83	3064.95	3064.95	3209.46	3064.85	3064.85
$\omega_4(a^{'})$	2959.91	2975.76	2960.29	2960.29	3095.73	2960.35	2960.35
$\omega_5(a^{'})$	1524.85	1528.89	1524.82	1524.82	1537.34	1524.63	1524.63
$\omega_6(a^{'})$	1504.35	1507.95	1504.27	1504.27	1517.26	1503.95	1503.95
$\omega_7(a^{'})$	1467.77	1464.14	1467.85	1467.85	1490.30	1468.48	1468.48
$\omega_8(a^{'})$	1273.32	1269.73	1273.33	1273.33	1288.79	1273.35	1273.37
$\omega_9(a^{'})$	1198.28	1194.60	1198.24	1198.30	1213.83	1198.31	1198.31
$\omega_{10}(a^{'})$	953.41	952.91	953.44	953.43	974.25	953.44	953.42
$\omega_{11}(a^{'})$	812.70	806.34	812.80	812.72	839.15	812.87	812.88
$\omega_{12}(a^{'})$	385.55	382.31	385.57	385.57	394.92	385.61	385.61
$\omega_{13}(a^{'})$	268.98	272.36	269.00	268.98	283.93	269.08	269.03
$\omega_{14}(a^{''})$	3112.90	3138.55	3112.86	3112.86	3262.75	3112.79	3112.79
$\omega_{15}(a^{''})$	3066.32	3088.87	3066.02	3066.02	3210.46	3065.93	3065.93
$\omega_{16}(a^{''})$	2958.30	2976.09	2958.64	2958.64	3091.65	2958.70	2958.70
$\omega_{17}(a^{''})$	1522.52	1523.91	1522.02	1522.02	1538.75	1521.72	1521.72
$\omega_{18}(a^{''})$	1492.67	1495.80	1492.44	1492.44	1505.15	1491.93	1491.93
$\omega_{19}(a^{''})$	1478.41	1470.34	1479.07	1479.07	1496.46	1479.46	1479.46
$\omega_{20}(a^{''})$	1438.95	1435.42	1438.91	1438.91	1460.39	1439.28	1439.28
$\omega_{21}(a^{''})$	1177.66	1175.95	1177.77	1177.77	1207.28	1177.64	1177.93
$\omega_{22}(a^{''})$	1100.92	1102.63	1100.95	1100.95	1115.69	1101.29	1100.99
$\omega_{23}(a^{''})$	1028.85	1029.19	1028.87	1028.87	1043.26	1029.07	1029.07
$\omega_{24}(a^{''})$	223.19	227.54	223.19	223.19	238.22	223.24	223.24

Table S271: CMA-0A and CMA-2A (ξ = 0.02) harmonic frequencies targeting CCSD(T)/cc-pVTZ for Level B = B3LYP/6-31G(2*df*,*p*).

	Pure B3LYP/ 6-31G(2*df*,*p*)	CMA-0A B3LYP/ 6-31G(2*df*,*p*)	CMA-2A B3LYP/ 6-31G(2*df*,*p*)
$\omega_1(a^{'})$	3534.62	3524.70	3524.70
$\omega_2(a^{'})$	3131.28	3113.80	3113.80
$\omega_3(a^{'})$	3083.28	3065.14	3065.14
$\omega_4(a^{'})$	2975.97	2960.02	2960.02
$\omega_5(a^{'})$	1522.17	1524.78	1524.78
$\omega_6(a^{'})$	1504.31	1504.34	1504.34
$\omega_7(a^{'})$	1476.12	1467.81	1467.81
$\omega_8(a^{'})$	1277.66	1273.32	1273.33
$\omega_9(a^{'})$	1197.72	1198.14	1198.30
$\omega_{10}(a^{'})$	952.04	953.43	953.43
$\omega_{11}(a^{'})$	796.69	813.07	812.90
$\omega_{12}(a^{'})$	392.91	385.68	385.61
$\omega_{13}(a^{'})$	271.17	269.15	269.05
$\omega_{14}(a^{''})$	3130.24	3112.89	3112.89
$\omega_{15}(a^{''})$	3083.49	3066.21	3066.21
$\omega_{16}(a^{''})$	2971.19	2958.34	2958.34
$\omega_{17}(a^{''})$	1519.52	1522.38	1522.38
$\omega_{18}(a^{''})$	1491.55	1492.61	1492.61
$\omega_{19}(a^{''})$	1479.21	1478.17	1478.17
$\omega_{20}(a^{''})$	1445.67	1439.07	1439.07
$\omega_{21}(a^{''})$	1162.49	1177.59	1177.91
$\omega_{22}(a^{''})$	1103.77	1101.30	1100.96
$\omega_{23}(a^{''})$	1035.34	1029.20	1029.22
$\omega_{24}(a^{''})$	235.02	223.39	223.31

Table S272: Symmetrized, unnormalized natural internal coordinates for dimethylamine.

1	$r_{1,2}$
2	$r_{1,3} + r_{1,4}$
3	$r_{1,3} - r_{1,4}$
4	$r_{3,6} + r_{3,5} + r_{3,7} + r_{4,9} + r_{4,8} + r_{4,10}$
5	$r_{3,6} + r_{3,5} + r_{3,7} - r_{4,9} - r_{4,8} - r_{4,10}$
6	$2r_{3,6} - r_{3,5} - r_{3,7} + 2r_{4,9} - r_{4,8} - r_{4,10}$
7	$2r_{3,6} - r_{3,5} - r_{3,7} - 2r_{4,9} + r_{4,8} + r_{4,10}$
8	$r_{3,5} - r_{3,7} + r_{4,8} - r_{4,10}$
9	$r_{3,5} - r_{3,7} - r_{4,8} + r_{4,10}$
10	$\phi_{3,1,4}$
11	$\phi_{2,1,3} + \phi_{2,1,4}$
12	$\phi_{2,1,3} - \phi_{2,1,4}$
13	$\phi_{6,3,1} + \phi_{5,3,1} + \phi_{7,3,1} - \phi_{5,3,7} - \phi_{6,3,7} - \phi_{5,3,6} + \phi_{9,4,1} + \phi_{8,4,1} + \phi_{10,4,1} - \phi_{8,4,10}$ $-\phi_{9,4,10} - \phi_{8,4,9}$
14	$\phi_{6,3,1} + \phi_{5,3,1} + \phi_{7,3,1} - \phi_{5,3,7} - \phi_{6,3,7} - \phi_{5,3,6} - \phi_{9,4,1} - \phi_{8,4,1} - \phi_{10,4,1} + \phi_{8,4,10}$ $+\phi_{9,4,10} + \phi_{8,4,9}$
15	$2\phi_{6,3,1} - \phi_{5,3,1} - \phi_{7,3,1} + 2\phi_{9,4,1} - \phi_{8,4,1} - \phi_{10,4,1}$
16	$2\phi_{6,3,1} - \phi_{5,3,1} - \phi_{7,3,1} - 2\phi_{9,4,1} + \phi_{8,4,1} + \phi_{10,4,1}$
17	$\phi_{5,3,1} - \phi_{7,3,1} + \phi_{8,4,1} - \phi_{10,4,1}$
18	$\phi_{5,3,1} - \phi_{7,3,1} - \phi_{8,4,1} + \phi_{10,4,1}$
19	$2\phi_{5,3,7} - \phi_{6,3,7} - \phi_{5,3,6} + 2\phi_{8,4,10} - \phi_{9,4,10} - \phi_{8,4,9}$
20	$2\phi_{5,3,7} - \phi_{6,3,7} - \phi_{5,3,6} - 2\phi_{8,4,10} + \phi_{9,4,10} + \phi_{8,4,9}$
21	$\phi_{6,3,7} - \phi_{5,3,6} + \phi_{9,4,10} - \phi_{8,4,9}$
22	$\phi_{6,3,7} - \phi_{5,3,6} - \phi_{9,4,10} + \phi_{8,4,9}$
23	$\tau_{5,3,1,4} + \tau_{5,3,1,2} + \tau_{6,3,1,4} + \tau_{6,3,1,2} + \tau_{7,3,1,4} + \tau_{7,3,1,2} + \tau_{8,4,1,3} + \tau_{8,4,1,2} + \tau_{9,4,1,3} + \tau_{9,4,1,2}$ $+\tau_{10,4,1,3} + \tau_{10,4,1,2}$
24	$\tau_{5,3,1,4} + \tau_{5,3,1,2} + \tau_{6,3,1,4} + \tau_{6,3,1,2} + \tau_{7,3,1,4} + \tau_{7,3,1,2} - \tau_{8,4,1,3} - \tau_{8,4,1,2} - \tau_{9,4,1,3} - \tau_{9,4,1,2}$ $-\tau_{10,4,1,3} - \tau_{10,4,1,2}$

S4.69 ethylamine

Table S273: CCSD(T)/cc-pVTZ Optimum Cartesian coordinates (bohr)

1	C	−2.41599594	0.50146691	0.00000000
2	C	0.01027304	−1.06763773	−0.00000000
3	N	2.36790056	0.39595653	0.00000000
4	H	−4.09501177	−0.70240098	−0.00000000
5	H	−2.49875000	1.71532507	−1.67014001
6	H	−2.49875000	1.71532507	1.67014001
7	H	0.04170761	−2.30474842	1.65378697
8	H	0.04170761	−2.30474842	−1.65378697
9	H	2.37659366	1.56049390	1.52504554
10	H	2.37659366	1.56049390	−1.52504554

Table S274: Reference [CCSD(T)/cc-pVTZ] CMA-0A and CMA-2A (ξ = 0.02) harmonic frequencies for Level B = MP2/cc-pVTZ and CCSD(T)/cc-pVDZ.

	Reference CCSD(T)/ cc-pVTZ	Pure MP2/ cc-pVTZ	CMA-0A MP2/ cc-pVTZ	CMA-2A MP2/ cc-pVTZ	Pure CCSD(T)/ cc-pVDZ	CMA-0A CCSD(T)/ cc-pVDZ	CMA-2A CCSD(T)/ cc-pVDZ
$\omega_1(a^{'})$	3477.75	3492.65	3477.74	3477.74	3585.95	3477.65	3477.65
$\omega_2(a^{'})$	3100.09	3125.35	3100.05	3100.05	3249.82	3099.96	3099.96
$\omega_3(a^{'})$	3043.45	3060.46	3043.41	3043.41	3179.50	3043.38	3043.38
$\omega_4(a^{'})$	3022.37	3034.86	3022.44	3022.44	3155.37	3022.49	3022.49
$\omega_5(a^{'})$	1663.18	1653.06	1663.16	1663.16	1686.01	1663.13	1663.13
$\omega_6(a^{'})$	1511.30	1512.63	1511.25	1511.25	1524.53	1511.22	1511.22
$\omega_7(a^{'})$	1493.31	1495.51	1493.33	1493.33	1506.31	1493.29	1493.29
$\omega_8(a^{'})$	1408.58	1400.43	1408.50	1408.50	1432.91	1408.11	1408.11
$\omega_9(a^{'})$	1380.25	1373.92	1380.28	1380.29	1399.69	1380.64	1380.64
$\omega_{10}(a^{'})$	1160.25	1157.59	1160.24	1160.27	1177.35	1159.86	1160.36
$\omega_{11}(a^{'})$	1081.55	1079.90	1081.60	1081.61	1111.07	1082.36	1081.98
$\omega_{12}(a^{'})$	914.85	909.22	914.29	914.92	924.06	913.60	915.09
$\omega_{13}(a^{'})$	877.07	871.99	877.81	877.08	899.22	878.99	877.25
$\omega_{14}(a^{'})$	396.61	396.27	396.61	396.61	404.00	396.65	396.65
$\omega_{15}(a^{''})$	3559.16	3585.80	3559.16	3559.16	3676.93	3559.16	3559.16
$\omega_{16}(a^{''})$	3103.24	3128.83	3103.24	3103.24	3253.41	3103.05	3103.05
$\omega_{17}(a^{''})$	3074.11	3100.85	3074.11	3074.11	3225.31	3074.26	3074.26
$\omega_{18}(a^{''})$	1499.43	1502.17	1499.42	1499.42	1511.70	1499.45	1499.45
$\omega_{19}(a^{''})$	1396.10	1394.04	1396.03	1396.03	1407.95	1395.92	1395.92
$\omega_{20}(a^{''})$	1276.10	1273.22	1276.18	1276.18	1287.39	1276.18	1276.18
$\omega_{21}(a^{''})$	1008.36	1007.32	1008.38	1008.38	1017.12	1008.54	1008.54
$\omega_{22}(a^{''})$	777.12	778.19	777.12	777.13	789.30	777.16	777.17
$\omega_{23}(a^{''})$	288.51	290.76	288.45	288.44	325.92	287.72	288.53
$\omega_{24}(a^{''})$	254.09	257.07	254.18	254.18	270.79	255.12	254.14

Table S275: CMA-0A and CMA-2A (ξ = 0.02) harmonic frequencies targeting CCSD(T)/cc-pVTZ for Level B = B3LYP/6-31G(2df,p).

	Pure B3LYP/ 6-31G(2df,p)	CMA-0A B3LYP/ 6-31G(2df,p)	CMA-2A B3LYP/ 6-31G(2df,p)
$\omega_1(a^{'})$	3494.25	3477.73	3477.73
$\omega_2(a^{'})$	3114.33	3100.07	3100.07
$\omega_3(a^{'})$	3058.28	3043.44	3043.44
$\omega_4(a^{'})$	3040.82	3022.36	3022.36
$\omega_5(a^{'})$	1659.94	1663.11	1663.11
$\omega_6(a^{'})$	1512.35	1511.23	1511.23
$\omega_7(a^{'})$	1492.39	1493.31	1493.31
$\omega_8(a^{'})$	1413.89	1407.81	1407.81
$\omega_9(a^{'})$	1384.48	1380.75	1380.77
$\omega_{10}(a^{'})$	1161.91	1160.03	1160.26
$\omega_{11}(a^{'})$	1067.51	1081.95	1082.03
$\omega_{12}(a^{'})$	921.66	915.25	914.96
$\omega_{13}(a^{'})$	874.99	877.32	877.21
$\omega_{14}(a^{'})$	404.49	396.71	396.71
$\omega_{15}(a^{''})$	3570.08	3559.15	3559.15
$\omega_{16}(a^{''})$	3118.93	3103.23	3103.23
$\omega_{17}(a^{''})$	3087.45	3074.10	3074.10
$\omega_{18}(a^{''})$	1500.67	1499.40	1499.40
$\omega_{19}(a^{''})$	1400.24	1395.88	1395.88
$\omega_{20}(a^{''})$	1283.15	1276.28	1276.28
$\omega_{21}(a^{''})$	1015.95	1008.51	1008.51
$\omega_{22}(a^{''})$	783.12	777.17	777.17
$\omega_{23}(a^{''})$	289.98	288.52	288.52
$\omega_{24}(a^{''})$	249.54	254.17	254.17

Table S276: Symmetrized, unnormalized natural internal coordinates for ethylamine.

1	$r_{1,2}$
2	$r_{2,3}$
3	$r_{1,4} + r_{1,5} + r_{1,6}$
4	$2r_{1,4} - r_{1,5} - r_{1,6}$
5	$r_{1,5} - r_{1,6}$
6	$r_{2,7} + r_{2,8}$
7	$r_{2,7} - r_{2,8}$
8	$r_{3,9} + r_{3,10}$
9	$r_{3,9} - r_{3,10}$
10	$\phi_{1,2,3}$
11	$\phi_{4,1,2} + \phi_{5,1,2} + \phi_{6,1,2} - \phi_{5,1,6} - \phi_{4,1,5} - \phi_{4,1,6}$
12	$2\phi_{4,1,2} - \phi_{5,1,2} - \phi_{6,1,2}$
13	$\phi_{5,1,2} - \phi_{6,1,2}$
14	$2\phi_{5,1,6} - \phi_{4,1,5} - \phi_{4,1,6}$
15	$\phi_{4,1,5} - \phi_{4,1,6}$
16	$4\phi_{7,2,8} - \phi_{7,2,1} - \phi_{7,2,3} - \phi_{8,2,1} - \phi_{8,2,3}$
17	$\phi_{7,2,1} + \phi_{7,2,3} - \phi_{8,2,1} - \phi_{8,2,3}$
18	$\phi_{7,2,1} - \phi_{7,2,3} + \phi_{8,2,1} - \phi_{8,2,3}$
19	$\phi_{7,2,1} - \phi_{7,2,3} - \phi_{8,2,1} + \phi_{8,2,3}$
20	$2\phi_{9,3,10} - \phi_{9,3,2} - \phi_{10,3,2}$
21	$\phi_{9,3,2} - \phi_{10,3,2}$
22	$\tau_{4,1,2,3} + \tau_{5,1,2,3} + \tau_{6,1,2,3}$
23	$\tau_{9,3,2,1} + \tau_{10,3,2,1}$
24	$\gamma_{2,3,9,10}$

S4.70 acetone

Table S277: CCSD(T)/cc-pVTZ Optimum Cartesian coordinates (bohr)

1	C	−0.00000000	−2.43146449	1.32246298
2	C	−0.00000000	−0.00000000	−0.19161611
3	C	0.00000000	2.43146449	1.32246298
4	H	−0.00000000	−4.04696082	0.05128367
5	H	−1.66203278	−2.50063407	2.54685039
6	H	1.66203278	−2.50063407	2.54685039
7	H	0.00000000	4.04696082	0.05128367
8	H	1.66203278	2.50063407	2.54685039
9	H	−1.66203278	2.50063407	2.54685039
10	O	−0.00000000	0.00000000	−2.48892898

Table S278: Reference [CCSD(T)/cc-pVTZ] CMA-0A and CMA-2A ($\xi = 0.02$) harmonic frequencies for Level B = MP2/cc-pVTZ and CCSD(T)/cc-pVDZ.

	Reference CCSD(T)/ cc-pVTZ	Pure MP2/ cc-pVTZ	CMA-0A MP2/ cc-pVTZ	CMA-2A MP2/ cc-pVTZ	Pure CCSD(T)/ cc-pVDZ	CMA-0A CCSD(T)/ cc-pVDZ	CMA-2A CCSD(T)/ cc-pVDZ
$\omega_1(a_1)$	3159.26	3180.74	3159.20	3159.20	3308.05	3159.19	3159.19
$\omega_2(a_1)$	3042.41	3054.46	3042.47	3042.47	3172.76	3042.43	3042.43
$\omega_3(a_1)$	1786.22	1778.64	1786.17	1786.17	1840.41	1786.05	1786.05
$\omega_4(a_1)$	1476.54	1477.39	1476.52	1476.52	1487.37	1476.74	1476.74
$\omega_5(a_1)$	1386.96	1377.91	1387.01	1387.01	1404.26	1386.92	1386.92
$\omega_6(a_1)$	1081.17	1078.20	1081.21	1081.21	1094.08	1081.24	1081.24
$\omega_7(a_1)$	794.73	792.57	794.75	794.75	821.82	794.93	794.93
$\omega_8(a_1)$	371.56	371.71	371.56	371.56	379.62	371.57	371.57
$\omega_9(a_2)$	3104.03	3128.22	3104.02	3104.02	3252.26	3103.98	3103.98
$\omega_{10}(a_2)$	1474.27	1475.61	1474.27	1474.27	1486.86	1474.30	1474.30
$\omega_{11}(a_2)$	887.40	881.97	887.40	887.41	896.04	887.50	887.50
$\omega_{12}(a_2)$	20.78	-10.47	21.34	20.79	58.11	21.01	20.78
$\omega_{13}(b_1)$	3110.28	3134.05	3110.28	3110.28	3257.86	3110.24	3110.24
$\omega_{14}(b_1)$	1495.93	1498.64	1495.92	1495.92	1507.85	1495.92	1495.92
$\omega_{15}(b_1)$	1116.27	1112.83	1116.27	1116.28	1127.17	1116.37	1116.37
$\omega_{16}(b_1)$	480.47	481.32	480.48	480.50	490.57	480.48	480.49
$\omega_{17}(b_1)$	141.09	140.80	141.21	141.09	156.43	141.16	141.12
$\omega_{18}(b_2)$	3157.81	3179.75	3157.76	3157.76	3306.78	3157.73	3157.73
$\omega_{19}(b_2)$	3037.08	3049.81	3037.13	3037.13	3167.90	3037.10	3037.10
$\omega_{20}(b_2)$	1468.80	1469.12	1468.76	1468.76	1478.99	1468.82	1468.82
$\omega_{21}(b_2)$	1395.11	1385.60	1395.14	1395.14	1421.48	1394.02	1394.02
$\omega_{22}(b_2)$	1246.46	1240.19	1246.47	1246.47	1271.50	1247.22	1247.45
$\omega_{23}(b_2)$	894.52	888.87	894.55	894.55	914.67	895.31	894.99
$\omega_{24}(b_2)$	527.58	528.02	527.58	527.58	537.24	527.64	527.63

Table S279: CMA-0A and CMA-2A (ξ = 0.02) harmonic frequencies targeting CCSD(T)/cc-pVTZ for Level B = B3LYP/6-31G(2df,p).

	Pure B3LYP/ 6-31G(2df,p)	CMA-0A B3LYP/ 6-31G(2df,p)	CMA-2A B3LYP/ 6-31G(2df,p)
$\omega_1(a_1)$	3176.10	3159.25	3159.25
$\omega_2(a_1)$	3061.60	3042.37	3042.37
$\omega_3(a_1)$	1792.90	1786.07	1786.18
$\omega_4(a_1)$	1472.91	1476.21	1476.21
$\omega_5(a_1)$	1390.39	1387.35	1387.35
$\omega_6(a_1)$	1088.75	1081.21	1081.21
$\omega_7(a_1)$	789.52	795.12	794.88
$\omega_8(a_1)$	377.56	371.61	371.61
$\omega_9(a_2)$	3116.92	3104.02	3104.02
$\omega_{10}(a_2)$	1470.86	1474.22	1474.22
$\omega_{11}(a_2)$	887.00	887.44	887.49
$\omega_{12}(a_2)$	57.17	23.01	20.84
$\omega_{13}(b_1)$	3124.35	3110.27	3110.27
$\omega_{14}(b_1)$	1493.08	1495.89	1495.90
$\omega_{15}(b_1)$	1119.92	1116.28	1116.30
$\omega_{16}(b_1)$	491.62	480.50	480.50
$\omega_{17}(b_1)$	145.64	141.38	141.16
$\omega_{18}(b_2)$	3174.71	3157.79	3157.79
$\omega_{19}(b_2)$	3054.89	3037.05	3037.05
$\omega_{20}(b_2)$	1464.09	1468.43	1468.43
$\omega_{21}(b_2)$	1391.65	1394.45	1394.45
$\omega_{22}(b_2)$	1240.48	1247.15	1247.15
$\omega_{23}(b_2)$	892.94	895.31	895.31
$\omega_{24}(b_2)$	528.97	527.64	527.64

Table S280: Symmetrized, unnormalized natural internal coordinates for acetone.

1	$r_{2,10}$
2	$r_{1,2} + r_{2,3}$
3	$r_{1,2} - r_{2,3}$
4	$r_{1,4} + r_{1,5} + r_{1,6} + r_{3,7} + r_{3,9} + r_{3,8}$
5	$r_{1,4} + r_{1,5} + r_{1,6} - r_{3,7} - r_{3,9} - r_{3,8}$
6	$2r_{1,4} - r_{1,5} - r_{1,6} + 2r_{3,7} - r_{3,9} - r_{3,8}$
7	$2r_{1,4} - r_{1,5} - r_{1,6} - 2r_{3,7} + r_{3,9} + r_{3,8}$
8	$r_{1,5} - r_{1,6} + r_{3,9} - r_{3,8}$
9	$r_{1,5} - r_{1,6} - r_{3,9} + r_{3,8}$
10	$2\phi_{1,2,3} - \phi_{1,2,10} - \phi_{3,2,10}$
11	$\phi_{1,2,10} - \phi_{3,2,10}$
12	$\phi_{4,1,2} + \phi_{5,1,2} + \phi_{6,1,2} - \phi_{5,1,6} - \phi_{4,1,5} - \phi_{4,1,6} + \phi_{7,3,2} + \phi_{9,3,2} + \phi_{8,3,2} - \phi_{8,3,9}$ $-\phi_{7,3,9} - \phi_{7,3,8}$
13	$\phi_{4,1,2} + \phi_{5,1,2} + \phi_{6,1,2} - \phi_{5,1,6} - \phi_{4,1,5} - \phi_{4,1,6} - \phi_{7,3,2} - \phi_{9,3,2} - \phi_{8,3,2} + \phi_{8,3,9}$ $+\phi_{7,3,9} + \phi_{7,3,8}$
14	$2\phi_{4,1,2} - \phi_{5,1,2} - \phi_{6,1,2} + 2\phi_{7,3,2} - \phi_{9,3,2} - \phi_{8,3,2}$
15	$2\phi_{4,1,2} - \phi_{5,1,2} - \phi_{6,1,2} - 2\phi_{7,3,2} + \phi_{9,3,2} + \phi_{8,3,2}$
16	$\phi_{5,1,2} - \phi_{6,1,2} + \phi_{9,3,2} - \phi_{8,3,2}$
17	$\phi_{5,1,2} - \phi_{6,1,2} - \phi_{9,3,2} + \phi_{8,3,2}$
18	$2\phi_{5,1,6} - \phi_{4,1,5} - \phi_{4,1,6} + 2\phi_{8,3,9} - \phi_{7,3,9} - \phi_{7,3,8}$
19	$2\phi_{5,1,6} - \phi_{4,1,5} - \phi_{4,1,6} - 2\phi_{8,3,9} + \phi_{7,3,9} + \phi_{7,3,8}$
20	$\phi_{4,1,5} - \phi_{4,1,6} + \phi_{7,3,9} - \phi_{7,3,8}$
21	$\phi_{4,1,5} - \phi_{4,1,6} - \phi_{7,3,9} + \phi_{7,3,8}$
22	$\tau_{4,1,2,3} + \tau_{5,1,2,3} + \tau_{6,1,2,3} + \tau_{7,3,2,1} + \tau_{8,3,2,1} + \tau_{9,3,2,1}$
23	$\tau_{4,1,2,3} + \tau_{5,1,2,3} + \tau_{6,1,2,3} - \tau_{7,3,2,1} - \tau_{8,3,2,1} - \tau_{9,3,2,1}$
24	$\gamma_{10,2,1,3}$

S4.71 1-chloropropane

Table S281: CCSD(T)/cc-pVTZ Optimum Cartesian coordinates (bohr)

1	C	−4.78185816	0.13547591	0.00000000
2	C	−2.11109876	−0.97139330	0.00000000
3	C	−0.14244981	1.11919067	0.00000000
4	Cl	3.00981998	−0.14144508	0.00000000
5	H	−6.19879779	−1.36232962	0.00000000
6	H	−5.09986073	1.30896986	−1.66945794
7	H	−5.09986073	1.30896986	1.66945794
8	H	−1.82642436	−2.16506020	1.66021781
9	H	−1.82642436	−2.16506020	−1.66021781
10	H	−0.30600557	2.30469630	1.67497536
11	H	−0.30600557	2.30469630	−1.67497536

Table S282: Reference [CCSD(T)/cc-pVTZ] CMA-0A and CMA-2A (ξ = 0.02) harmonic frequencies for Level B = MP2/cc-pVTZ and CCSD(T)/cc-pVDZ.

	Reference CCSD(T)/ cc-pVTZ	Pure MP2/ cc-pVTZ	CMA-0A MP2/ cc-pVTZ	CMA-2A MP2/ cc-pVTZ	Pure CCSD(T)/ cc-pVDZ	CMA-0A CCSD(T)/ cc-pVDZ	CMA-2A CCSD(T)/ cc-pVDZ
$\omega_1(a^{'})$	3116.65	3141.29	3116.60	3116.60	3265.93	3116.51	3116.51
$\omega_2(a^{'})$	3079.76	3095.25	3079.72	3079.72	3217.49	3079.61	3079.61
$\omega_3(a^{'})$	3054.12	3069.69	3054.13	3054.13	3191.07	3054.14	3054.14
$\omega_4(a^{'})$	3033.76	3046.22	3033.84	3033.84	3167.00	3033.92	3033.92
$\omega_5(a^{'})$	1514.46	1515.23	1514.21	1514.21	1526.78	1514.41	1514.41
$\omega_6(a^{'})$	1499.57	1500.16	1499.71	1499.71	1513.02	1498.81	1498.81
$\omega_7(a^{'})$	1493.06	1493.03	1493.08	1493.08	1509.84	1493.63	1493.63
$\omega_8(a^{'})$	1414.57	1406.75	1414.41	1414.41	1436.22	1413.64	1413.64
$\omega_9(a^{'})$	1375.19	1363.98	1375.34	1375.34	1401.77	1375.63	1375.63
$\omega_{10}(a^{'})$	1284.71	1281.69	1284.75	1284.75	1303.28	1284.82	1284.82
$\omega_{11}(a^{'})$	1125.98	1123.94	1126.01	1126.02	1148.02	1125.71	1126.26
$\omega_{12}(a^{'})$	1055.55	1054.32	1055.61	1055.61	1086.89	1056.62	1056.29
$\omega_{13}(a^{'})$	915.17	913.94	915.20	915.20	937.51	915.61	915.32
$\omega_{14}(a^{'})$	757.78	764.72	757.78	757.80	769.96	757.82	757.82
$\omega_{15}(a^{'})$	363.85	363.54	363.88	363.86	372.13	363.89	363.89
$\omega_{16}(a^{'})$	233.16	231.58	233.18	233.18	240.01	233.18	233.18
$\omega_{17}(a^{''})$	3139.46	3162.42	3139.46	3139.46	3288.93	3139.37	3139.37
$\omega_{18}(a^{''})$	3108.28	3133.24	3108.27	3108.27	3258.37	3108.20	3108.20
$\omega_{19}(a^{''})$	3086.79	3112.35	3086.79	3086.79	3237.98	3086.91	3086.91
$\omega_{20}(a^{''})$	1506.44	1509.09	1506.43	1506.43	1517.28	1506.48	1506.48
$\omega_{21}(a^{''})$	1322.93	1322.61	1322.92	1322.92	1336.51	1322.91	1322.91
$\omega_{22}(a^{''})$	1251.33	1249.87	1251.26	1251.26	1266.03	1251.32	1251.32
$\omega_{23}(a^{''})$	1097.32	1098.42	1097.43	1097.43	1112.76	1097.34	1097.34
$\omega_{24}(a^{''})$	870.11	870.42	870.12	870.12	882.45	870.15	870.15
$\omega_{25}(a^{''})$	750.95	752.59	750.96	750.96	766.48	751.00	751.00
$\omega_{26}(a^{''})$	231.61	234.66	231.62	231.62	247.20	231.66	231.65
$\omega_{27}(a^{''})$	117.40	119.40	117.41	117.40	125.55	117.46	117.44

Table S283: CMA-0A and CMA-2A (ξ = 0.02) harmonic frequencies targeting CCSD(T)/cc-pVTZ for Level B = B3LYP/6-31G(2df,p).

	Pure B3LYP/ 6-31G(2df,p)	CMA-0A B3LYP/ 6-31G(2df,p)	CMA-2A B3LYP/ 6-31G(2df,p)
$\omega_1(a^{'})$	3132.15	3116.64	3116.64
$\omega_2(a^{'})$	3095.17	3079.73	3079.73
$\omega_3(a^{'})$	3067.39	3054.04	3054.04
$\omega_4(a^{'})$	3053.02	3033.83	3033.83
$\omega_5(a^{'})$	1516.76	1514.27	1514.27
$\omega_6(a^{'})$	1498.84	1499.64	1499.64
$\omega_7(a^{'})$	1490.71	1493.10	1493.10
$\omega_8(a^{'})$	1421.34	1414.01	1414.01
$\omega_9(a^{'})$	1378.32	1375.05	1375.05
$\omega_{10}(a^{'})$	1292.20	1284.81	1284.81
$\omega_{11}(a^{'})$	1126.03	1125.78	1125.78
$\omega_{12}(a^{'})$	1045.64	1056.38	1056.38
$\omega_{13}(a^{'})$	914.15	915.42	915.42
$\omega_{14}(a^{'})$	756.07	758.09	758.09
$\omega_{15}(a^{'})$	367.35	363.93	363.93
$\omega_{16}(a^{'})$	239.47	233.23	233.23
$\omega_{17}(a^{''})$	3155.94	3139.44	3139.44
$\omega_{18}(a^{''})$	3123.57	3108.22	3108.22
$\omega_{19}(a^{''})$	3099.53	3086.84	3086.84
$\omega_{20}(a^{''})$	1506.12	1506.43	1506.43
$\omega_{21}(a^{''})$	1325.29	1322.91	1322.91
$\omega_{22}(a^{''})$	1254.51	1251.26	1251.26
$\omega_{23}(a^{''})$	1098.13	1097.39	1097.39
$\omega_{24}(a^{''})$	875.22	870.17	870.17
$\omega_{25}(a^{''})$	757.04	751.00	751.00
$\omega_{26}(a^{''})$	235.16	231.69	231.72
$\omega_{27}(a^{''})$	119.63	117.49	117.44

Table S284: Symmetrized, unnormalized natural internal coordinates for 1-chloropropane.

1	$r_{1,2}$
2	$r_{2,3}$
3	$r_{3,4}$
4	$r_{1,5} + r_{1,6} + r_{1,7}$
5	$2r_{1,5} - r_{1,6} - r_{1,7}$
6	$r_{1,6} - r_{1,7}$
7	$r_{2,8} + r_{2,9}$
8	$r_{2,8} - r_{2,9}$
9	$r_{3,10} + r_{3,11}$
10	$r_{3,10} - r_{3,11}$
11	$\phi_{1,2,3}$
12	$\phi_{2,3,4}$
13	$\phi_{5,1,2} + \phi_{6,1,2} + \phi_{7,1,2} - \phi_{6,1,7} - \phi_{5,1,6} - \phi_{5,1,7}$
14	$2\phi_{5,1,2} - \phi_{6,1,2} - \phi_{7,1,2}$
15	$\phi_{6,1,2} - \phi_{7,1,2}$
16	$2\phi_{6,1,7} - \phi_{5,1,6} - \phi_{5,1,7}$
17	$\phi_{5,1,6} - \phi_{5,1,7}$
18	$4\phi_{8,2,9} - \phi_{8,2,1} - \phi_{8,2,3} - \phi_{9,2,1} - \phi_{9,2,3}$
19	$\phi_{8,2,1} + \phi_{8,2,3} - \phi_{9,2,1} - \phi_{9,2,3}$
20	$\phi_{8,2,1} - \phi_{8,2,3} + \phi_{9,2,1} - \phi_{9,2,3}$
21	$\phi_{8,2,1} - \phi_{8,2,3} - \phi_{9,2,1} + \phi_{9,2,3}$
22	$4\phi_{10,3,11} - \phi_{10,3,2} - \phi_{10,3,4} - \phi_{11,3,2} - \phi_{11,3,4}$
23	$\phi_{10,3,2} + \phi_{10,3,4} - \phi_{11,3,2} - \phi_{11,3,4}$
24	$\phi_{10,3,2} - \phi_{10,3,4} + \phi_{11,3,2} - \phi_{11,3,4}$
25	$\phi_{10,3,2} - \phi_{10,3,4} - \phi_{11,3,2} + \phi_{11,3,4}$
26	$\tau_{1,2,3,4}$
27	$\tau_{5,1,2,3} + \tau_{6,1,2,3} + \tau_{7,1,2,3}$

S4.72 methoxyethane

Table S285: CCSD(T)/cc-pVTZ Optimum Cartesian coordinates (bohr)

1	C	−3.51555612	0.29044205	−0.00000000
2	C	−0.95458299	−0.99195423	−0.00000000
3	O	0.93589970	0.90095503	−0.00000000
4	C	3.37683746	−0.17280447	0.00000000
5	H	−5.02516284	−1.11599211	−0.00000000
6	H	−3.71909489	1.47840980	−1.67234300
7	H	−3.71909489	1.47840980	1.67234300
8	H	−0.74126596	−2.20420926	1.67415920
9	H	−0.74126596	−2.20420926	−1.67415920
10	H	4.73626773	1.37209229	0.00000000
11	H	3.68698239	−1.34648639	−1.68213636
12	H	3.68698239	−1.34648639	1.68213636

Table S286: Reference [CCSD(T)/cc-pVTZ] CMA-0A and CMA-2A (ξ = 0.02) harmonic frequencies for Level B = MP2/cc-pVTZ and CCSD(T)/cc-pVDZ.

	Reference CCSD(T)/ cc-pVTZ	Pure MP2/ cc-pVTZ	CMA-0A MP2/ cc-pVTZ	CMA-2A MP2/ cc-pVTZ	Pure CCSD(T)/ cc-pVDZ	CMA-0A CCSD(T)/ cc-pVDZ	CMA-2A CCSD(T)/ cc-pVDZ
$\omega_1(a^{'})$	3128.05	3152.28	3127.97	3127.97	3277.30	3127.95	3127.95
$\omega_2(a^{'})$	3123.74	3148.85	3123.73	3123.73	3273.34	3123.67	3123.67
$\omega_3(a^{'})$	3045.76	3058.25	3045.74	3045.74	3178.71	3045.71	3045.71
$\omega_4(a^{'})$	2985.25	2998.08	2985.32	2985.32	3116.78	2985.26	2985.26
$\omega_5(a^{'})$	2971.39	2987.06	2971.43	2971.43	3100.89	2971.42	2971.42
$\omega_6(a^{'})$	1538.29	1539.62	1538.21	1538.21	1555.26	1537.79	1537.79
$\omega_7(a^{'})$	1515.64	1519.59	1515.63	1515.63	1527.00	1515.31	1515.31
$\omega_8(a^{'})$	1506.90	1509.56	1506.84	1506.84	1521.46	1506.62	1506.62
$\omega_9(a^{'})$	1480.96	1479.15	1480.99	1480.99	1500.66	1481.76	1481.76
$\omega_{10}(a^{'})$	1431.74	1424.41	1431.71	1431.71	1458.54	1431.67	1431.67
$\omega_{11}(a^{'})$	1396.67	1391.31	1396.71	1396.71	1413.05	1396.75	1396.75
$\omega_{12}(a^{'})$	1244.73	1239.93	1244.68	1244.68	1259.37	1244.72	1244.78
$\omega_{13}(a^{'})$	1174.17	1172.52	1174.22	1174.22	1197.42	1174.32	1174.24
$\omega_{14}(a^{'})$	1119.09	1117.88	1119.15	1119.16	1133.61	1118.94	1119.27
$\omega_{15}(a^{'})$	1048.43	1045.21	1048.53	1048.53	1073.78	1049.22	1048.92
$\omega_{16}(a^{'})$	874.88	872.53	874.93	874.92	892.64	875.12	875.05
$\omega_{17}(a^{'})$	468.22	465.34	468.23	468.22	474.65	468.26	468.26
$\omega_{18}(a^{'})$	288.79	286.76	288.80	288.80	291.86	288.87	288.87
$\omega_{19}(a^{''})$	3128.68	3153.69	3128.67	3128.67	3281.30	3128.61	3128.61
$\omega_{20}(a^{''})$	3034.72	3060.25	3034.69	3034.69	3180.90	3034.68	3034.68
$\omega_{21}(a^{''})$	3002.00	3027.67	3002.02	3002.02	3144.98	3002.03	3002.03
$\omega_{22}(a^{''})$	1495.37	1500.64	1495.36	1495.36	1505.87	1495.40	1495.40
$\omega_{23}(a^{''})$	1490.30	1493.01	1490.29	1490.29	1500.92	1490.33	1490.33
$\omega_{24}(a^{''})$	1301.19	1302.33	1301.19	1301.19	1316.47	1301.17	1301.17
$\omega_{25}(a^{''})$	1204.65	1203.26	1204.60	1204.60	1220.72	1204.55	1204.55
$\omega_{26}(a^{''})$	1171.74	1172.06	1171.84	1171.84	1185.04	1171.93	1171.93
$\omega_{27}(a^{''})$	820.71	821.83	820.73	820.73	834.66	820.74	820.76
$\omega_{28}(a^{''})$	259.12	262.98	259.13	259.13	274.71	259.10	259.16
$\omega_{29}(a^{''})$	209.79	213.19	209.80	209.80	218.56	210.00	209.85
$\omega_{30}(a^{''})$	112.85	115.09	112.87	112.86	112.96	112.92	112.86

Table S287: CMA-0A and CMA-2A (ξ = 0.02) harmonic frequencies targeting CCSD(T)/cc-pVTZ for Level B = B3LYP/6-31G(2df,p).

	Pure B3LYP/ 6-31G(2df,p)	CMA-0A B3LYP/ 6-31G(2df,p)	CMA-2A B3LYP/ 6-31G(2df,p)
$\omega_1(a^{'})$	3148.36	3128.04	3128.04
$\omega_2(a^{'})$	3139.02	3123.73	3123.73
$\omega_3(a^{'})$	3065.48	3045.74	3045.74
$\omega_4(a^{'})$	3005.07	2985.20	2985.20
$\omega_5(a^{'})$	2983.59	2971.39	2971.39
$\omega_6(a^{'})$	1536.74	1538.07	1538.07
$\omega_7(a^{'})$	1512.63	1515.28	1515.28
$\omega_8(a^{'})$	1504.97	1507.19	1507.19
$\omega_9(a^{'})$	1483.87	1481.01	1481.01
$\omega_{10}(a^{'})$	1432.46	1431.45	1431.45
$\omega_{11}(a^{'})$	1405.99	1396.73	1396.73
$\omega_{12}(a^{'})$	1240.24	1244.00	1244.00
$\omega_{13}(a^{'})$	1155.56	1175.24	1175.24
$\omega_{14}(a^{'})$	1121.08	1119.25	1119.29
$\omega_{15}(a^{'})$	1040.63	1048.58	1048.58
$\omega_{16}(a^{'})$	872.96	875.22	875.16
$\omega_{17}(a^{'})$	473.03	468.26	468.26
$\omega_{18}(a^{'})$	292.76	288.89	288.89
$\omega_{19}(a^{''})$	3145.30	3128.67	3128.67
$\omega_{20}(a^{''})$	3049.93	3034.70	3034.70
$\omega_{21}(a^{''})$	3011.83	3002.01	3002.01
$\omega_{22}(a^{''})$	1495.53	1495.32	1495.32
$\omega_{23}(a^{''})$	1489.84	1490.29	1490.29
$\omega_{24}(a^{''})$	1303.48	1301.03	1301.03
$\omega_{25}(a^{''})$	1205.46	1204.64	1204.64
$\omega_{26}(a^{''})$	1172.69	1171.95	1171.96
$\omega_{27}(a^{''})$	828.69	820.81	820.81
$\omega_{28}(a^{''})$	261.64	258.89	259.15
$\omega_{29}(a^{''})$	213.04	210.06	209.82
$\omega_{30}(a^{''})$	118.17	113.27	112.91

Table S288: Symmetrized, unnormalized natural internal coordinates for methoxyethane.

1	$r_{1,2}$
2	$r_{2,3}$
3	$r_{3,4}$
4	$r_{1,5} + r_{1,6} + r_{1,7}$
5	$2r_{1,5} - r_{1,6} - r_{1,7}$
6	$r_{1,6} - r_{1,7}$
7	$r_{2,8} + r_{2,9}$
8	$r_{2,8} - r_{2,9}$
9	$r_{4,10} + r_{4,11} + r_{4,12}$
10	$2r_{4,10} - r_{4,11} - r_{4,12}$
11	$r_{4,11} - r_{4,12}$
12	$\phi_{1,2,3}$
13	$\phi_{2,3,4}$
14	$\phi_{5,1,2} + \phi_{6,1,2} + \phi_{7,1,2} - \phi_{6,1,7} - \phi_{5,1,6} - \phi_{5,1,7}$
15	$2\phi_{5,1,2} - \phi_{6,1,2} - \phi_{7,1,2}$
16	$\phi_{6,1,2} - \phi_{7,1,2}$
17	$2\phi_{6,1,7} - \phi_{5,1,6} - \phi_{5,1,7}$
18	$\phi_{5,1,6} - \phi_{5,1,7}$
19	$4\phi_{8,2,9} - \phi_{8,2,1} - \phi_{8,2,3} - \phi_{9,2,1} - \phi_{9,2,3}$
20	$\phi_{8,2,1} + \phi_{8,2,3} - \phi_{9,2,1} - \phi_{9,2,3}$
21	$\phi_{8,2,1} - \phi_{8,2,3} + \phi_{9,2,1} - \phi_{9,2,3}$
22	$\phi_{8,2,1} - \phi_{8,2,3} - \phi_{9,2,1} + \phi_{9,2,3}$
23	$\phi_{10,4,3} + \phi_{11,4,3} + \phi_{12,4,3} - \phi_{11,4,12} - \phi_{10,4,11} - \phi_{10,4,12}$
24	$2\phi_{10,4,3} - \phi_{11,4,3} - \phi_{12,4,3}$
25	$\phi_{11,4,3} - \phi_{12,4,3}$
26	$2\phi_{11,4,12} - \phi_{10,4,11} - \phi_{10,4,12}$
27	$\phi_{10,4,11} - \phi_{10,4,12}$
28	$\tau_{1,2,3,4}$
29	$\tau_{5,1,2,3} + \tau_{6,1,2,3} + \tau_{7,1,2,3}$
30	$\tau_{10,4,3,2} + \tau_{11,4,3,2} + \tau_{12,4,3,2}$

S4.73 isopropyl alcohol

Table S289: CCSD(T)/cc-pVTZ Optimum Cartesian coordinates (bohr)

1	C	−2.38831701	−1.37281068	0.19751772
2	C	−0.00000002	−0.02316624	−0.68077558
3	C	2.38831634	−1.37281178	0.19751772
4	H	−4.06437603	−0.36748732	−0.45670502
5	H	−2.44637430	−1.47961484	2.26098859
6	H	−2.44637460	−3.30273182	−0.54053624
7	H	−0.00000003	0.07560930	−2.74320395
8	O	0.00000063	2.56018606	0.10662450
9	H	0.00000066	2.55954490	1.92575998
10	H	4.06437582	−0.36748919	−0.45670501
11	H	2.44637317	−3.30273286	−0.54053635
12	H	2.44637346	−1.47961611	2.26098862

Table S290: Reference [CCSD(T)/cc-pVTZ] CMA-0A and CMA-2A (ξ = 0.02) harmonic frequencies for Level B = MP2/cc-pVTZ and CCSD(T)/cc-pVDZ.

	Reference CCSD(T)/ cc-pVTZ	Pure MP2/ cc-pVTZ	CMA-0A MP2/ cc-pVTZ	CMA-2A MP2/ cc-pVTZ	Pure CCSD(T)/ cc-pVDZ	CMA-0A CCSD(T)/ cc-pVDZ	CMA-2A CCSD(T)/ cc-pVDZ
$\omega_1(a^{'})$	3819.49	3837.20	3819.49	3819.49	3888.71	3819.48	3819.48
$\omega_2(a^{'})$	3124.15	3147.94	3124.09	3124.09	3272.90	3124.02	3124.02
$\omega_3(a^{'})$	3103.92	3128.00	3103.87	3103.87	3250.64	3103.57	3103.57
$\omega_4(a^{'})$	3060.75	3082.51	3060.79	3060.79	3200.47	3060.92	3060.92
$\omega_5(a^{'})$	3028.61	3040.23	3028.66	3028.66	3160.47	3028.83	3028.83
$\omega_6(a^{'})$	1511.47	1514.78	1511.25	1511.25	1524.58	1510.55	1510.55
$\omega_7(a^{'})$	1503.80	1505.12	1503.90	1503.90	1515.13	1504.45	1504.45
$\omega_8(a^{'})$	1427.15	1417.55	1426.83	1426.94	1452.49	1427.09	1427.09
$\omega_9(a^{'})$	1410.33	1402.75	1410.32	1410.32	1429.61	1410.32	1410.32
$\omega_{10}(a^{'})$	1315.36	1308.10	1315.51	1315.53	1333.37	1315.50	1315.51
$\omega_{11}(a^{'})$	1196.43	1192.15	1196.56	1196.43	1216.89	1196.32	1196.42
$\omega_{12}(a^{'})$	1092.18	1084.99	1092.36	1092.36	1109.47	1092.37	1092.37
$\omega_{13}(a^{'})$	983.15	981.53	983.24	983.23	1001.22	983.40	983.28
$\omega_{14}(a^{'})$	825.99	824.93	826.02	826.00	847.12	826.33	826.32
$\omega_{15}(a^{'})$	464.61	464.43	464.64	464.64	475.73	464.64	464.64
$\omega_{16}(a^{'})$	357.74	356.46	357.74	357.74	363.72	357.77	357.77
$\omega_{17}(a^{'})$	270.92	274.89	271.02	271.02	286.69	271.17	271.17
$\omega_{18}(a^{''})$	3121.53	3146.06	3121.49	3121.49	3270.38	3121.42	3121.42
$\omega_{19}(a^{''})$	3092.77	3118.42	3092.76	3092.76	3241.58	3092.73	3092.73
$\omega_{20}(a^{''})$	3024.63	3037.59	3024.67	3024.67	3156.40	3024.69	3024.69
$\omega_{21}(a^{''})$	1491.04	1492.12	1489.63	1489.63	1503.31	1489.54	1489.54
$\omega_{22}(a^{''})$	1487.92	1490.44	1489.26	1489.26	1500.44	1489.20	1489.20
$\omega_{23}(a^{''})$	1403.42	1393.68	1403.41	1403.41	1429.49	1402.95	1402.95
$\omega_{24}(a^{''})$	1367.15	1362.97	1367.12	1367.12	1381.25	1367.29	1367.29
$\omega_{25}(a^{''})$	1159.76	1160.05	1159.84	1159.84	1185.09	1160.14	1160.38
$\omega_{26}(a^{''})$	939.40	936.87	939.44	939.44	957.82	939.41	939.11
$\omega_{27}(a^{''})$	922.41	920.54	922.43	922.46	934.66	923.02	923.07
$\omega_{28}(a^{''})$	425.28	423.88	425.26	425.28	432.39	424.44	425.30
$\omega_{29}(a^{''})$	272.99	272.29	273.01	273.01	314.28	272.61	272.97
$\omega_{30}(a^{''})$	224.49	227.34	224.71	224.58	244.14	226.96	224.70

Table S291: CMA-0A and CMA-2A (ξ = 0.02) harmonic frequencies targeting CCSD(T)/cc-pVTZ for Level B = B3LYP/6-31G(2*df*,*p*).

	Pure B3LYP/ 6-31G(2*df*,*p*)	CMA-0A B3LYP/ 6-31G(2*df*,*p*)	CMA-2A B3LYP/ 6-31G(2*df*,*p*)
$\omega_1(a^{'})$	3817.47	3819.48	3819.48
$\omega_2(a^{'})$	3138.85	3124.13	3124.13
$\omega_3(a^{'})$	3118.17	3103.92	3103.92
$\omega_4(a^{'})$	3072.74	3060.69	3060.69
$\omega_5(a^{'})$	3046.89	3028.64	3028.64
$\omega_6(a^{'})$	1512.15	1511.06	1511.06
$\omega_7(a^{'})$	1502.61	1504.05	1504.05
$\omega_8(a^{'})$	1424.37	1424.69	1424.80
$\omega_9(a^{'})$	1417.68	1412.74	1412.74
$\omega_{10}(a^{'})$	1317.39	1315.30	1315.30
$\omega_{11}(a^{'})$	1196.11	1195.82	1195.82
$\omega_{12}(a^{'})$	1091.05	1092.13	1092.13
$\omega_{13}(a^{'})$	975.75	984.41	984.24
$\omega_{14}(a^{'})$	826.19	826.09	826.09
$\omega_{15}(a^{'})$	473.78	464.70	464.70
$\omega_{16}(a^{'})$	362.49	357.77	357.77
$\omega_{17}(a^{'})$	266.28	271.13	271.13
$\omega_{18}(a^{''})$	3136.11	3121.50	3121.50
$\omega_{19}(a^{''})$	3105.80	3092.77	3092.77
$\omega_{20}(a^{''})$	3041.68	3024.61	3024.61
$\omega_{21}(a^{''})$	1488.93	1489.65	1489.65
$\omega_{22}(a^{''})$	1487.97	1489.14	1489.14
$\omega_{23}(a^{''})$	1405.76	1402.11	1402.11
$\omega_{24}(a^{''})$	1369.78	1368.19	1368.19
$\omega_{25}(a^{''})$	1156.58	1159.90	1159.90
$\omega_{26}(a^{''})$	944.59	938.75	938.75
$\omega_{27}(a^{''})$	928.56	923.67	923.69
$\omega_{28}(a^{''})$	424.52	425.22	425.33
$\omega_{29}(a^{''})$	263.64	272.55	273.10
$\omega_{30}(a^{''})$	226.40	225.62	224.66

Table S292: Symmetrized, unnormalized natural internal coordinates for isopropyl alcohol.

1	$r_{2,8}$
2	$r_{2,7}$
3	$r_{8,9}$
4	$r_{2,1} + r_{2,3}$
5	$r_{2,1} - r_{2,3}$
6	$r_{1,6} + r_{1,4} + r_{1,5} + r_{3,11} + r_{3,10} + r_{3,12}$
7	$r_{1,6} + r_{1,4} + r_{1,5} - r_{3,11} - r_{3,10} - r_{3,12}$
8	$2r_{1,6} - r_{1,4} - r_{1,5} + 2r_{3,11} - r_{3,10} - r_{3,12}$
9	$2r_{1,6} - r_{1,4} - r_{1,5} - 2r_{3,11} + r_{3,10} + r_{3,12}$
10	$r_{1,4} - r_{1,5} + r_{3,10} - r_{3,12}$
11	$r_{1,4} - r_{1,5} - r_{3,10} + r_{3,12}$
12	$\phi_{2,8,9}$
13	$\phi_{1,2,3}$
14	$\phi_{1,2,7} - \phi_{3,2,7}$
15	$\phi_{1,2,8} - \phi_{3,2,8}$
16	$\phi_{6,1,2} + \phi_{4,1,2} + \phi_{5,1,2} - \phi_{4,1,5} - \phi_{4,1,6} - \phi_{5,1,6} + \phi_{11,3,2} + \phi_{10,3,2} + \phi_{12,3,2} - \phi_{10,3,12}$ $-\phi_{10,3,11} - \phi_{12,3,11}$
17	$\phi_{6,1,2} + \phi_{4,1,2} + \phi_{5,1,2} - \phi_{4,1,5} - \phi_{4,1,6} - \phi_{5,1,6} - \phi_{11,3,2} - \phi_{10,3,2} - \phi_{12,3,2} + \phi_{10,3,12}$ $+\phi_{10,3,11} + \phi_{12,3,11}$
18	$2\phi_{6,1,2} - \phi_{4,1,2} - \phi_{5,1,2} + 2\phi_{11,3,2} - \phi_{10,3,2} - \phi_{12,3,2}$
19	$2\phi_{6,1,2} - \phi_{4,1,2} - \phi_{5,1,2} - 2\phi_{11,3,2} + \phi_{10,3,2} + \phi_{12,3,2}$
20	$\phi_{4,1,2} - \phi_{5,1,2} + \phi_{10,3,2} - \phi_{12,3,2}$
21	$\phi_{4,1,2} - \phi_{5,1,2} - \phi_{10,3,2} + \phi_{12,3,2}$
22	$2\phi_{4,1,5} - \phi_{4,1,6} - \phi_{5,1,6} + 2\phi_{10,3,12} - \phi_{10,3,11} - \phi_{12,3,11}$
23	$2\phi_{4,1,5} - \phi_{4,1,6} - \phi_{5,1,6} - 2\phi_{10,3,12} + \phi_{10,3,11} + \phi_{12,3,11}$
24	$\phi_{4,1,6} - \phi_{5,1,6} + \phi_{10,3,11} - \phi_{12,3,11}$
25	$\phi_{4,1,6} - \phi_{5,1,6} - \phi_{10,3,11} + \phi_{12,3,11}$
26	$\tau_{9,8,2,7}$
27	$\tau_{4,1,2,3} + \tau_{5,1,2,3} + \tau_{6,1,2,3} + \tau_{10,3,2,1} + \tau_{11,3,2,1} + \tau_{12,3,2,1}$
28	$\tau_{4,1,2,3} + \tau_{5,1,2,3} + \tau_{6,1,2,3} - \tau_{10,3,2,1} - \tau_{11,3,2,1} - \tau_{12,3,2,1}$
29	$\gamma_{7,2,1,3}$
30	$\gamma_{8,2,1,3}$

S4.74 propane

Table S293: CCSD(T)/cc-pVTZ Optimum Cartesian coordinates (bohr)

1	C	−2.39592864	0.51179667	0.00000000
2	C	0.00000000	−1.10217789	0.00000000
3	C	2.39592869	0.51179661	0.00000000
4	H	−4.09939241	−0.65278475	−0.00000000
5	H	−2.45820670	1.73037440	−1.66734556
6	H	−2.45820670	1.73037440	1.66734556
7	H	−0.00000041	−2.34011719	1.65598761
8	H	−0.00000041	−2.34011719	−1.65598761
9	H	4.09939243	−0.65278485	0.00000000
10	H	2.45820678	1.73037434	1.66734556
11	H	2.45820678	1.73037434	−1.66734556

Table S294: Reference [CCSD(T)/cc-pVTZ] CMA-0A and CMA-2A (ξ = 0.02) harmonic frequencies for Level B = MP2/cc-pVTZ and CCSD(T)/cc-pVDZ.

	Reference CCSD(T)/ cc-pVTZ	Pure MP2/ cc-pVTZ	CMA-0A MP2/ cc-pVTZ	CMA-2A MP2/ cc-pVTZ	Pure CCSD(T)/ cc-pVDZ	CMA-0A CCSD(T)/ cc-pVDZ	CMA-2A CCSD(T)/ cc-pVDZ
$\omega_1(a_1)$	3107.75	3132.57	3107.70	3107.70	3257.73	3107.61	3107.61
$\omega_2(a_1)$	3033.74	3051.22	3033.60	3033.60	3171.94	3033.46	3033.46
$\omega_3(a_1)$	3028.77	3040.48	3028.95	3028.95	3161.66	3029.09	3029.09
$\omega_4(a_1)$	1518.58	1519.97	1518.41	1518.41	1530.60	1518.52	1518.52
$\omega_5(a_1)$	1496.33	1497.26	1496.47	1496.47	1508.94	1496.34	1496.34
$\omega_6(a_1)$	1420.81	1414.04	1420.81	1420.81	1441.24	1420.87	1420.87
$\omega_7(a_1)$	1181.29	1178.69	1181.33	1181.33	1197.09	1181.28	1181.39
$\omega_8(a_1)$	885.78	885.83	885.79	885.79	911.77	886.10	885.96
$\omega_9(a_1)$	362.53	360.93	362.54	362.54	370.89	362.55	362.55
$\omega_{10}(a_2)$	3093.71	3120.11	3093.70	3093.70	3245.64	3093.68	3093.68
$\omega_{11}(a_2)$	1496.59	1499.09	1496.57	1496.57	1508.02	1496.64	1496.64
$\omega_{12}(a_2)$	1319.32	1319.47	1319.33	1319.33	1332.92	1319.31	1319.31
$\omega_{13}(a_2)$	906.61	906.42	906.63	906.63	919.24	906.63	906.63
$\omega_{14}(a_2)$	215.56	219.02	215.56	215.56	232.17	215.60	215.60
$\omega_{15}(b_1)$	3104.68	3129.58	3104.68	3104.68	3254.75	3104.54	3104.54
$\omega_{16}(b_1)$	3061.46	3088.50	3061.46	3061.46	3213.09	3061.57	3061.57
$\omega_{17}(b_1)$	1513.81	1517.04	1513.80	1513.80	1525.34	1513.83	1513.83
$\omega_{18}(b_1)$	1217.54	1213.24	1217.56	1217.56	1231.87	1217.56	1217.56
$\omega_{19}(b_1)$	748.63	749.35	748.63	748.64	763.57	748.67	748.67
$\omega_{20}(b_1)$	273.41	277.18	273.42	273.41	291.22	273.47	273.45
$\omega_{21}(b_2)$	3105.03	3130.85	3105.02	3105.02	3255.30	3104.95	3104.95
$\omega_{22}(b_2)$	3026.70	3040.09	3026.71	3026.71	3160.07	3026.71	3026.71
$\omega_{23}(b_2)$	1503.87	1506.01	1503.86	1503.86	1515.87	1503.75	1503.75
$\omega_{24}(b_2)$	1407.52	1398.58	1407.36	1407.36	1434.35	1406.58	1406.58
$\omega_{25}(b_2)$	1367.14	1359.66	1367.30	1367.30	1385.51	1367.78	1367.78
$\omega_{26}(b_2)$	1074.04	1074.70	1074.07	1074.07	1105.78	1074.72	1074.72
$\omega_{27}(b_2)$	928.98	928.28	929.00	929.00	942.62	929.13	929.13

Table S295: CMA-0A and CMA-2A (ξ = 0.02) harmonic frequencies targeting CCSD(T)/cc-pVTZ for Level B = B3LYP/6-31G(2df,p).

	Pure B3LYP/ 6-31G(2df,p)	CMA-0A B3LYP/ 6-31G(2df,p)	CMA-2A B3LYP/ 6-31G(2df,p)
$\omega_1(a_1)$	3122.61	3107.74	3107.74
$\omega_2(a_1)$	3048.81	3033.45	3033.45
$\omega_3(a_1)$	3046.05	3029.03	3029.03
$\omega_4(a_1)$	1520.67	1518.44	1518.44
$\omega_5(a_1)$	1495.76	1496.39	1496.39
$\omega_6(a_1)$	1429.91	1420.81	1420.81
$\omega_7(a_1)$	1186.12	1181.33	1181.33
$\omega_8(a_1)$	883.94	885.94	885.94
$\omega_9(a_1)$	371.25	362.59	362.59
$\omega_{10}(a_2)$	3108.70	3093.70	3093.70
$\omega_{11}(a_2)$	1496.76	1496.57	1496.57
$\omega_{12}(a_2)$	1322.24	1319.31	1319.31
$\omega_{13}(a_2)$	914.42	906.66	906.66
$\omega_{14}(a_2)$	221.96	215.66	215.66
$\omega_{15}(b_1)$	3120.55	3104.65	3104.65
$\omega_{16}(b_1)$	3072.39	3061.48	3061.48
$\omega_{17}(b_1)$	1514.65	1513.80	1513.80
$\omega_{18}(b_1)$	1225.27	1217.55	1217.55
$\omega_{19}(b_1)$	756.23	748.66	748.66
$\omega_{20}(b_1)$	273.92	273.48	273.48
$\omega_{21}(b_2)$	3119.65	3105.03	3105.03
$\omega_{22}(b_2)$	3045.26	3026.67	3026.67
$\omega_{23}(b_2)$	1503.14	1503.85	1503.85
$\omega_{24}(b_2)$	1412.38	1406.66	1406.66
$\omega_{25}(b_2)$	1375.82	1367.65	1367.65
$\omega_{26}(b_2)$	1066.21	1074.39	1074.39
$\omega_{27}(b_2)$	936.20	929.28	929.28

Table S296: Symmetrized, unnormalized natural internal coordinates for propane.

1	$r_{1,2} + r_{2,3}$
2	$r_{1,2} - r_{2,3}$
3	$r_{1,4} + r_{1,5} + r_{1,6} + r_{3,9} + r_{3,11} + r_{3,10}$
4	$r_{1,4} + r_{1,5} + r_{1,6} - r_{3,9} - r_{3,11} - r_{3,10}$
5	$2r_{1,4} - r_{1,5} - r_{1,6} + 2r_{3,9} - r_{3,11} - r_{3,10}$
6	$2r_{1,4} - r_{1,5} - r_{1,6} - 2r_{3,9} + r_{3,11} + r_{3,10}$
7	$r_{1,5} - r_{1,6} + r_{3,11} - r_{3,10}$
8	$r_{1,5} - r_{1,6} - r_{3,11} + r_{3,10}$
9	$r_{2,7} + r_{2,8}$
10	$r_{2,7} - r_{2,8}$
11	$\phi_{1,2,3}$
12	$\phi_{4,1,2} + \phi_{5,1,2} + \phi_{6,1,2} - \phi_{5,1,6} - \phi_{4,1,5} - \phi_{4,1,6} + \phi_{9,3,2} + \phi_{11,3,2} + \phi_{10,3,2} - \phi_{10,3,11}$ $-\phi_{9,3,11} - \phi_{9,3,10}$
13	$\phi_{4,1,2} + \phi_{5,1,2} + \phi_{6,1,2} - \phi_{5,1,6} - \phi_{4,1,5} - \phi_{4,1,6} - \phi_{9,3,2} - \phi_{11,3,2} - \phi_{10,3,2} + \phi_{10,3,11}$ $+\phi_{9,3,11} + \phi_{9,3,10}$
14	$2\phi_{4,1,2} - \phi_{5,1,2} - \phi_{6,1,2} + 2\phi_{9,3,2} - \phi_{11,3,2} - \phi_{10,3,2}$
15	$2\phi_{4,1,2} - \phi_{5,1,2} - \phi_{6,1,2} - 2\phi_{9,3,2} + \phi_{11,3,2} + \phi_{10,3,2}$
16	$\phi_{5,1,2} - \phi_{6,1,2} + \phi_{11,3,2} - \phi_{10,3,2}$
17	$\phi_{5,1,2} - \phi_{6,1,2} - \phi_{11,3,2} + \phi_{10,3,2}$
18	$2\phi_{5,1,6} - \phi_{4,1,5} - \phi_{4,1,6} + 2\phi_{10,3,11} - \phi_{9,3,11} - \phi_{9,3,10}$
19	$2\phi_{5,1,6} - \phi_{4,1,5} - \phi_{4,1,6} - 2\phi_{10,3,11} + \phi_{9,3,11} + \phi_{9,3,10}$
20	$\phi_{4,1,5} - \phi_{4,1,6} + \phi_{9,3,11} - \phi_{9,3,10}$
21	$\phi_{4,1,5} - \phi_{4,1,6} - \phi_{9,3,11} + \phi_{9,3,10}$
22	$4\phi_{7,2,8} - \phi_{7,2,1} - \phi_{7,2,3} - \phi_{8,2,1} - \phi_{8,2,3}$
23	$\phi_{7,2,1} + \phi_{7,2,3} - \phi_{8,2,1} - \phi_{8,2,3}$
24	$\phi_{7,2,1} - \phi_{7,2,3} + \phi_{8,2,1} - \phi_{8,2,3}$
25	$\phi_{7,2,1} - \phi_{7,2,3} - \phi_{8,2,1} + \phi_{8,2,3}$
26	$\tau_{4,1,2,3} + \tau_{5,1,2,3} + \tau_{6,1,2,3} + \tau_{9,3,2,1} + \tau_{10,3,2,1} + \tau_{11,3,2,1}$
27	$\tau_{4,1,2,3} + \tau_{5,1,2,3} + \tau_{6,1,2,3} - \tau_{9,3,2,1} - \tau_{10,3,2,1} - \tau_{11,3,2,1}$

S4.75 acrylonitrile

Table S297: CCSD(T)/cc-pVTZ Optimum Cartesian coordinates (bohr)

1	C	−3.19156925	0.72327464	−0.00000000
2	C	−1.30268767	−0.96690359	−0.00000000
3	C	1.30844710	−0.21134227	−0.00000000
4	N	3.42266953	0.40043186	0.00000000
5	H	−5.13781563	0.09913705	−0.00000000
6	H	−2.82071717	2.73416350	−0.00000000
7	H	−1.66434467	−2.97977677	0.00000000

Table S298: Reference [CCSD(T)/cc-pVTZ] CMA-0A and CMA-2A (ξ = 0.02) harmonic frequencies for Level B = MP2/cc-pVTZ and CCSD(T)/cc-pVDZ.

	Reference CCSD(T)/ cc-pVTZ	Pure MP2/ cc-pVTZ	CMA-0A MP2/ cc-pVTZ	CMA-2A MP2/ cc-pVTZ	Pure CCSD(T)/ cc-pVDZ	CMA-0A CCSD(T)/ cc-pVDZ	CMA-2A CCSD(T)/ cc-pVDZ
$\omega_1(a^{'})$	3262.58	3282.29	3262.58	3262.58	3416.48	3262.54	3262.54
$\omega_2(a^{'})$	3202.58	3219.72	3202.57	3202.57	3346.66	3202.59	3202.59
$\omega_3(a^{'})$	3164.32	3178.91	3164.33	3164.33	3305.45	3164.32	3164.32
$\omega_4(a^{'})$	2271.92	2235.15	2271.89	2271.89	2373.23	2271.91	2271.91
$\omega_5(a^{'})$	1659.64	1653.42	1659.63	1659.65	1724.80	1658.42	1658.79
$\omega_6(a^{'})$	1447.12	1447.66	1447.04	1447.04	1468.19	1447.91	1447.91
$\omega_7(a^{'})$	1314.40	1313.06	1314.51	1314.48	1334.60	1314.80	1314.33
$\omega_8(a^{'})$	1104.14	1101.66	1104.17	1104.17	1124.60	1103.99	1104.01
$\omega_9(a^{'})$	873.15	871.30	873.21	873.21	906.59	873.79	873.79
$\omega_{10}(a^{'})$	559.50	559.08	559.51	559.52	569.25	559.59	559.54
$\omega_{11}(a^{'})$	227.13	226.77	227.14	227.13	231.47	227.13	227.13
$\omega_{12}(a^{''})$	993.49	1012.85	993.35	993.38	1003.89	993.48	993.48
$\omega_{13}(a^{''})$	969.04	972.04	969.15	969.15	973.70	969.03	969.03
$\omega_{14}(a^{''})$	688.12	700.45	688.14	688.12	694.49	688.12	688.13
$\omega_{15}(a^{''})$	337.74	336.95	337.76	337.74	341.72	337.80	337.76

Table S299: CMA-0A and CMA-2A (ξ = 0.02) harmonic frequencies targeting CCSD(T)/cc-pVTZ for Level B = B3LYP/6-31G(2*df*,*p*).

	Pure B3LYP/ 6-31G(2*df*,*p*)	CMA-0A B3LYP/ 6-31G(2*df*,*p*)	CMA-2A B3LYP/ 6-31G(2*df*,*p*)
$\omega_1(a^{'})$	3279.72	3262.57	3262.57
$\omega_2(a^{'})$	3213.54	3202.54	3202.54
$\omega_3(a^{'})$	3178.33	3164.33	3164.33
$\omega_4(a^{'})$	2290.35	2271.42	2271.57
$\omega_5(a^{'})$	1653.73	1659.55	1659.84
$\omega_6(a^{'})$	1439.27	1447.18	1447.18
$\omega_7(a^{'})$	1314.01	1314.86	1314.49
$\omega_8(a^{'})$	1100.55	1104.16	1104.16
$\omega_9(a^{'})$	865.49	873.87	873.49
$\omega_{10}(a^{'})$	571.33	559.45	559.53
$\omega_{11}(a^{'})$	233.39	227.41	227.23
$\omega_{12}(a^{''})$	1009.03	992.73	992.75
$\omega_{13}(a^{''})$	988.72	969.56	969.56
$\omega_{14}(a^{''})$	711.82	688.29	688.46
$\omega_{15}(a^{''})$	349.30	338.14	337.73

Table S300: Symmetrized, unnormalized natural internal coordinates for acrylonitrile.

1	$r_{1,2}$
2	$r_{2,3}$
3	$r_{3,4}$
4	$r_{2,7}$
5	$r_{1,5} + r_{1,6}$
6	$r_{1,5} - r_{1,6}$
7	$2\phi_{1,2,3} - \phi_{1,2,7} - \phi_{3,2,7}$
8	$\phi_{1,2,7} - \phi_{3,2,7}$
9	$2\phi_{6,1,5} - \phi_{2,1,6} - \phi_{2,1,5}$
10	$\phi_{2,1,6} - \phi_{2,1,5}$
11	$\tau_{6,1,2,3} + \tau_{5,1,2,3} + \tau_{6,1,2,7} + \tau_{5,1,2,7}$
12	$\gamma_{2,1,6,5}$
13	$\gamma_{7,2,3,1}$
14	$\alpha^{x}_{1,2,3,4}$
15	$\alpha^{y}_{1,2,3,4}$

S4.76 trimethylamine

Table S301: CCSD(T)/cc-pVTZ Optimum Cartesian coordinates (bohr)

1	X	−2.60567339	0.00000000	0.00000000
2	N	−0.71594686	0.00000000	0.00000000
3	C	0.17737959	−1.30146773	−2.25420824
4	C	0.17737959	2.60293546	0.00000000
5	C	0.17737959	−1.30146773	2.25420824
6	H	2.26161442	−1.36371156	−2.36201770
7	H	−0.52888531	−0.34189560	−3.93805529
8	H	−0.52888531	−3.23950812	−2.26511792
9	H	2.26161442	2.72742311	0.00000000
10	H	−0.52888531	3.58140372	1.67293737
11	H	−0.52888531	3.58140372	−1.67293737
12	H	2.26161442	−1.36371156	2.36201770
13	H	−0.52888531	−3.23950812	2.26511792
14	H	−0.52888531	−0.34189560	3.93805529

Table S302: Reference [CCSD(T)/cc-pVTZ] CMA-0A and CMA-2A (ξ = 0.02) harmonic frequencies for Level B = MP2/cc-pVTZ and CCSD(T)/cc-pVDZ.

	Reference CCSD(T)/ cc-pVTZ	Pure MP2/ cc-pVTZ	CMA-0A MP2/ cc-pVTZ	CMA-2A MP2/ cc-pVTZ	Pure CCSD(T)/ cc-pVDZ	CMA-0A CCSD(T)/ cc-pVDZ	CMA-2A CCSD(T)/ cc-pVDZ
$\omega_1(a_1)$	3071.93	3089.56	3071.61	3071.61	3211.95	3071.51	3071.51
$\omega_2(a_1)$	2934.80	2949.46	2935.12	2935.12	3070.53	2935.15	2935.15
$\omega_3(a_1)$	1508.19	1511.16	1508.14	1508.14	1520.04	1507.59	1507.59
$\omega_4(a_1)$	1478.58	1472.09	1478.61	1478.61	1500.16	1479.20	1479.20
$\omega_5(a_1)$	1215.49	1209.14	1215.49	1215.50	1230.71	1215.59	1215.59
$\omega_6(a_1)$	847.53	843.27	847.58	847.56	871.22	847.65	847.65
$\omega_7(a_1)$	373.11	367.48	373.12	373.12	382.53	373.17	373.14
$\omega_8(a_2)$	3112.25	3137.91	3112.25	3112.25	3259.78	3112.22	3112.22
$\omega_9(a_2)$	1496.49	1498.35	1496.49	1496.49	1507.63	1496.55	1496.55
$\omega_{10}(a_2)$	1063.58	1062.40	1063.60	1063.60	1074.64	1063.61	1063.61
$\omega_{11}(a_2)$	240.77	247.43	240.78	240.78	247.88	240.80	240.78
$\omega_{12a}(e)$	3116.44	3140.74	3116.43	3116.43	3264.03	3116.40	3116.40
$\omega_{12b}(e)$	3116.44	3140.69	3116.43	3116.43	3263.96	3116.40	3116.40
$\omega_{13a}(e)$	3068.49	3088.66	3068.19	3068.19	3208.78	3068.07	3068.07
$\omega_{13b}(e)$	3068.49	3088.65	3068.19	3068.19	3208.77	3068.06	3068.06
$\omega_{14a}(e)$	2925.30	2943.19	2925.61	2925.61	3058.65	2925.67	2925.67
$\omega_{14b}(e)$	2925.30	2943.11	2925.61	2925.61	3058.58	2925.67	2925.67
$\omega_{15a}(e)$	1517.12	1519.91	1517.11	1517.11	1528.31	1517.10	1517.10
$\omega_{15b}(e)$	1517.12	1519.88	1517.11	1517.11	1528.28	1517.10	1517.10
$\omega_{16a}(e)$	1488.69	1491.20	1488.67	1488.67	1499.47	1488.74	1488.74
$\omega_{16b}(e)$	1488.68	1491.17	1488.67	1488.67	1499.45	1488.74	1488.74
$\omega_{17a}(e)$	1436.74	1430.50	1436.63	1436.63	1458.82	1436.45	1436.45
$\omega_{17b}(e)$	1436.74	1430.47	1436.63	1436.63	1458.80	1436.45	1436.45
$\omega_{18a}(e)$	1309.86	1301.46	1309.96	1309.96	1334.89	1309.74	1310.17
$\omega_{18b}(e)$	1309.86	1301.45	1309.96	1309.96	1334.88	1309.74	1310.17
$\omega_{19a}(e)$	1120.56	1119.47	1120.57	1120.57	1134.44	1120.59	1120.59
$\omega_{19b}(e)$	1120.56	1119.46	1120.57	1120.57	1134.43	1120.59	1120.59
$\omega_{20a}(e)$	1065.00	1064.38	1065.07	1065.07	1089.04	1065.80	1065.27
$\omega_{20b}(e)$	1065.00	1064.36	1065.07	1065.07	1089.02	1065.80	1065.27
$\omega_{21a}(e)$	417.26	414.46	417.24	417.27	425.26	417.24	417.28
$\omega_{21b}(e)$	417.26	414.43	417.23	417.27	425.24	417.24	417.28
$\omega_{22a}(e)$	279.99	283.96	280.06	280.00	289.31	280.07	280.00
$\omega_{22b}(e)$	279.99	283.94	280.06	280.00	289.30	280.07	280.00

Table S303: CMA-0A and CMA-2A (ξ = 0.02) harmonic frequencies targeting CCSD(T)/cc-pVTZ for Level B = B3LYP/6-31G($2df$,p).

	Pure B3LYP/ 6-31G($2df$,p)	CMA-0A B3LYP/ 6-31G($2df$,p)	CMA-2A B3LYP/ 6-31G($2df$,p)
$\omega_1(a_1)$	3088.27	3071.83	3071.83
$\omega_2(a_1)$	2951.59	2934.87	2934.87
$\omega_3(a_1)$	1508.70	1508.03	1508.03
$\omega_4(a_1)$	1489.86	1478.74	1478.74
$\omega_5(a_1)$	1220.82	1215.50	1215.50
$\omega_6(a_1)$	845.62	847.58	847.59
$\omega_7(a_1)$	384.44	373.23	373.19
$\omega_8(a_2)$	3127.82	3112.24	3112.24
$\omega_9(a_2)$	1492.46	1496.47	1496.47
$\omega_{10}(a_2)$	1072.35	1063.61	1063.64
$\omega_{11}(a_2)$	254.42	240.96	240.83
$\omega_{12\mathrm{a}}(e)$	3135.82	3116.38	3116.38
$\omega_{12\mathrm{b}}(e)$	3129.98	3116.30	3116.30
$\omega_{13\mathrm{a}}(e)$	3085.10	3068.49	3068.49
$\omega_{13\mathrm{b}}(e)$	3083.82	3068.44	3068.44
$\omega_{14\mathrm{a}}(e)$	2942.85	2925.35	2925.35
$\omega_{14\mathrm{b}}(e)$	2932.73	2925.27	2925.27
$\omega_{15\mathrm{a}}(e)$	1519.47	1516.64	1516.64
$\omega_{15\mathrm{b}}(e)$	1510.60	1516.28	1516.28
$\omega_{16\mathrm{a}}(e)$	1491.96	1489.20	1489.20
$\omega_{16\mathrm{b}}(e)$	1483.10	1488.61	1488.61
$\omega_{17\mathrm{a}}(e)$	1448.64	1437.37	1437.37
$\omega_{17\mathrm{b}}(e)$	1442.35	1436.68	1436.68
$\omega_{18\mathrm{a}}(e)$	1306.24	1309.44	1309.44
$\omega_{18\mathrm{b}}(e)$	1304.72	1309.31	1309.31
$\omega_{19\mathrm{a}}(e)$	1128.38	1120.37	1120.38
$\omega_{19\mathrm{b}}(e)$	1125.51	1120.08	1120.08
$\omega_{20\mathrm{a}}(e)$	1061.71	1066.47	1066.47
$\omega_{20\mathrm{b}}(e)$	1060.97	1066.46	1066.46
$\omega_{21\mathrm{a}}(e)$	429.55	417.32	417.32
$\omega_{21\mathrm{b}}(e)$	426.69	417.20	417.32
$\omega_{22\mathrm{a}}(e)$	293.03	280.26	280.10
$\omega_{22\mathrm{b}}(e)$	267.14	280.11	280.04

Table S304: Symmetrized, unnormalized natural internal coordinates for trimethylamine.

1	$r_{2,3} + r_{2,4} + r_{2,5}$
2	$2r_{2,3} - r_{2,4} - r_{2,5}$
3	$r_{2,4} - r_{2,5}$
4	$r_{3,6} + r_{3,7} + r_{3,8} + r_{4,9} + r_{4,10} + r_{4,11} + r_{5,12} + r_{5,13} + r_{5,14}$
5	$2r_{3,6} + 2r_{3,7} + 2r_{3,8} - r_{4,9} - r_{4,10} - r_{4,11} - r_{5,12} - r_{5,13} - r_{5,14}$
6	$r_{4,9} + r_{4,10} + r_{4,11} - r_{5,12} - r_{5,13} - r_{5,14}$
7	$2r_{3,6} - r_{3,7} - r_{3,8} + 2r_{4,9} - r_{4,10} - r_{4,11} + 2r_{5,12} - r_{5,13} - r_{5,14}$
8	$4r_{3,6} - 2r_{3,7} - 2r_{3,8} - 2r_{4,9} + r_{4,10} + r_{4,11} - 2r_{5,12} + r_{5,13} + r_{5,14}$
9	$2r_{4,9} - r_{4,10} - r_{4,11} - 2r_{5,12} + r_{5,13} + r_{5,14}$
10	$r_{3,7} - r_{3,8} + r_{4,10} - r_{4,11} + r_{5,13} - r_{5,14}$
11	$2r_{3,7} - 2r_{3,8} - r_{4,10} + r_{4,11} - r_{5,13} + r_{5,14}$
12	$r_{4,10} - r_{4,11} - r_{5,13} + r_{5,14}$
13	$2\phi_{4,2,5} - \phi_{3,2,4} - \phi_{3,2,5}$
14	$\phi_{3,2,4} - \phi_{3,2,5}$
15	$\phi_{4,2,5} + \phi_{3,2,4} + \phi_{3,2,5} - \gamma_{3,2,4,5} - \gamma_{4,2,5,3} - \gamma_{5,2,3,4}$
16	$\phi_{6,3,2} + \phi_{7,3,2} + \phi_{8,3,2} - \phi_{7,3,8} - \phi_{6,3,8} - \phi_{6,3,7} + \phi_{9,4,2} + \phi_{10,4,2} + \phi_{11,4,2} - \phi_{10,4,11}$ $-\phi_{9,4,11} - \phi_{9,4,10} + \phi_{12,5,2} + \phi_{13,5,2} + \phi_{14,5,2} - \phi_{13,5,14} - \phi_{12,5,14} - \phi_{12,5,13}$
17	$2\phi_{6,3,2} + 2\phi_{7,3,2} + 2\phi_{8,3,2} - 2\phi_{7,3,8} - 2\phi_{6,3,8} - 2\phi_{6,3,7} - \phi_{9,4,2} - \phi_{10,4,2} - \phi_{11,4,2} + \phi_{10,4,11}$ $+\phi_{9,4,11} + \phi_{9,4,10} - \phi_{12,5,2} - \phi_{13,5,2} - \phi_{14,5,2} + \phi_{13,5,14} + \phi_{12,5,14} + \phi_{12,5,13}$
18	$\phi_{9,4,2} + \phi_{10,4,2} + \phi_{11,4,2} - \phi_{10,4,11} - \phi_{9,4,11} - \phi_{9,4,10} - \phi_{12,5,2} - \phi_{13,5,2} - \phi_{14,5,2} + \phi_{13,5,14}$ $+\phi_{12,5,14} + \phi_{12,5,13}$
19	$2\phi_{6,3,2} - \phi_{7,3,2} - \phi_{8,3,2} + 2\phi_{9,4,2} - \phi_{10,4,2} - \phi_{11,4,2} + 2\phi_{12,5,2} - \phi_{13,5,2} - \phi_{14,5,2}$
20	$4\phi_{6,3,2} - 2\phi_{7,3,2} - 2\phi_{8,3,2} - 2\phi_{9,4,2} + \phi_{10,4,2} + \phi_{11,4,2} - 2\phi_{12,5,2} + \phi_{13,5,2} + \phi_{14,5,2}$
21	$2\phi_{9,4,2} - \phi_{10,4,2} - \phi_{11,4,2} - 2\phi_{12,5,2} + \phi_{13,5,2} + \phi_{14,5,2}$
22	$\phi_{7,3,2} - \phi_{8,3,2} + \phi_{10,4,2} - \phi_{11,4,2} + \phi_{13,5,2} - \phi_{14,5,2}$
23	$2\phi_{7,3,2} - 2\phi_{8,3,2} - \phi_{10,4,2} + \phi_{11,4,2} - \phi_{13,5,2} + \phi_{14,5,2}$
24	$\phi_{10,4,2} - \phi_{11,4,2} - \phi_{13,5,2} + \phi_{14,5,2}$
25	$2\phi_{7,3,8} - \phi_{6,3,8} - \phi_{6,3,7} + 2\phi_{10,4,11} - \phi_{9,4,11} - \phi_{9,4,10} + 2\phi_{13,5,14} - \phi_{12,5,14} - \phi_{12,5,13}$
26	$4\phi_{7,3,8} - 2\phi_{6,3,8} - 2\phi_{6,3,7} - 2\phi_{10,4,11} + \phi_{9,4,11} + \phi_{9,4,10} - 2\phi_{13,5,14} + \phi_{12,5,14} + \phi_{12,5,13}$
27	$2\phi_{10,4,11} - \phi_{9,4,11} - \phi_{9,4,10} - 2\phi_{13,5,14} + \phi_{12,5,14} + \phi_{12,5,13}$
28	$\phi_{6,3,8} - \phi_{6,3,7} + \phi_{9,4,11} - \phi_{9,4,10} + \phi_{12,5,14} - \phi_{12,5,13}$
29	$2\phi_{6,3,8} - 2\phi_{6,3,7} - \phi_{9,4,11} + \phi_{9,4,10} - \phi_{12,5,14} + \phi_{12,5,13}$
30	$\phi_{9,4,11} - \phi_{9,4,10} - \phi_{12,5,14} + \phi_{12,5,13}$
31	$\tau_{6,3,2,4} + \tau_{6,3,2,5} + \tau_{7,3,2,4} + \tau_{7,3,2,5} + \tau_{8,3,2,4} + \tau_{8,3,2,5} + \tau_{9,4,2,5} + \tau_{9,4,2,3} + \tau_{10,4,2,5} + \tau_{10,4,2,3}$ $+\tau_{11,4,2,5} + \tau_{11,4,2,3} + \tau_{12,5,2,3} + \tau_{12,5,2,4} + \tau_{13,5,2,3} + \tau_{13,5,2,4} + \tau_{14,5,2,3} + \tau_{14,5,2,4}$
32	$2\tau_{6,3,2,4} + 2\tau_{6,3,2,5} + 2\tau_{7,3,2,4} + 2\tau_{7,3,2,5} + 2\tau_{8,3,2,4} + 2\tau_{8,3,2,5} - \tau_{9,4,2,5} - \tau_{9,4,2,3} - \tau_{10,4,2,5} - \tau_{10,4,2,3}$ $-\tau_{11,4,2,5} - \tau_{11,4,2,3} - \tau_{12,5,2,3} - \tau_{12,5,2,4} - \tau_{13,5,2,3} - \tau_{13,5,2,4} - \tau_{14,5,2,3} - \tau_{14,5,2,4}$
33	$\tau_{9,4,2,5} + \tau_{9,4,2,3} + \tau_{10,4,2,5} + \tau_{10,4,2,3} + \tau_{11,4,2,5} + \tau_{11,4,2,3} - \tau_{12,5,2,3} - \tau_{12,5,2,4} - \tau_{13,5,2,3} - \tau_{13,5,2,4}$ $-\tau_{14,5,2,3} - \tau_{14,5,2,4}$

S4.77 isobutane

Table S305: CCSD(T)/cc-pVTZ Optimum Cartesian coordinates (bohr)

1	H	0.00000456	−2.77586961	0.00000000
2	C	0.00000456	−0.70455970	0.00000000
3	C	−1.37291926	0.20069470	−2.37795577
4	C	−1.37291926	0.20069470	2.37795577
5	C	2.74583331	0.20069470	0.00000000
6	H	−1.40906973	2.26831964	−2.44058129
7	H	−3.32653703	−0.46815497	−2.41690302
8	H	−0.42983254	−0.46815497	−4.08931064
9	H	−1.40906973	2.26831964	2.44058129
10	H	−0.42983254	−0.46815497	4.08931064
11	H	−3.32653703	−0.46815497	2.41690302
12	H	2.81815313	2.26831964	0.00000000
13	H	3.75636436	−0.46815497	−1.67240762
14	H	3.75636436	−0.46815497	1.67240762

Table S306: Reference [CCSD(T)/cc-pVTZ] CMA-0A and CMA-2A ($\xi = 0.02$) harmonic frequencies for Level B = MP2/cc-pVTZ and CCSD(T)/cc-pVDZ.

	Reference CCSD(T)/ cc-pVTZ	Pure MP2/ cc-pVTZ	CMA-0A MP2/ cc-pVTZ	CMA-2A MP2/ cc-pVTZ	Pure CCSD(T)/ cc-pVDZ	CMA-0A CCSD(T)/ cc-pVDZ	CMA-2A CCSD(T)/ cc-pVDZ
$\omega_1(a_1)$	3097.55	3121.02	3097.48	3097.48	3245.40	3097.31	3097.31
$\omega_2(a_1)$	3024.21	3043.14	3022.65	3022.65	3160.83	3024.19	3024.19
$\omega_3(a_1)$	3019.64	3033.52	3021.26	3021.26	3155.38	3019.85	3019.85
$\omega_4(a_1)$	1518.97	1521.58	1518.96	1518.96	1530.25	1518.99	1518.99
$\omega_5(a_1)$	1425.66	1417.32	1425.66	1425.66	1446.50	1425.64	1425.64
$\omega_6(a_1)$	1214.05	1208.18	1214.06	1214.06	1229.12	1214.09	1214.09
$\omega_7(a_1)$	809.17	808.00	809.19	809.19	833.56	809.30	809.30
$\omega_8(a_1)$	424.86	422.51	424.87	424.87	436.38	424.90	424.90
$\omega_9(a_2)$	3098.77	3124.78	3098.76	3098.76	3246.96	3098.73	3098.73
$\omega_{10}(a_2)$	1488.02	1488.90	1488.01	1488.01	1498.60	1488.08	1488.08
$\omega_{11}(a_2)$	952.43	950.74	952.45	952.45	965.08	952.46	952.46
$\omega_{12}(a_2)$	200.88	205.14	200.88	200.88	220.09	200.90	200.90
$\omega_{13\mathrm{a}}(e)$	3102.64	3127.37	3102.63	3102.63	3251.05	3102.60	3102.60
$\omega_{13\mathrm{b}}(e)$	3102.63	3127.30	3102.63	3102.63	3250.97	3102.60	3102.60
$\omega_{14\mathrm{a}}(e)$	3088.15	3113.83	3088.11	3088.11	3237.33	3088.03	3088.03
$\omega_{14\mathrm{b}}(e)$	3088.14	3113.77	3088.11	3088.11	3237.28	3088.03	3088.03
$\omega_{15\mathrm{a}}(e)$	3018.86	3031.65	3018.89	3018.89	3150.70	3018.91	3018.91
$\omega_{15\mathrm{b}}(e)$	3018.85	3031.61	3018.89	3018.89	3150.65	3018.91	3018.91
$\omega_{16\mathrm{a}}(e)$	1512.26	1513.98	1512.23	1512.23	1524.04	1512.11	1512.11
$\omega_{16\mathrm{b}}(e)$	1512.26	1513.98	1512.22	1512.22	1524.03	1512.10	1512.10
$\omega_{17\mathrm{a}}(e)$	1493.69	1495.18	1493.68	1493.68	1504.83	1493.73	1493.73
$\omega_{17\mathrm{b}}(e)$	1493.68	1495.16	1493.67	1493.67	1504.81	1493.73	1493.73
$\omega_{18\mathrm{a}}(e)$	1401.61	1391.22	1401.46	1401.46	1426.86	1400.62	1400.62
$\omega_{18\mathrm{b}}(e)$	1401.61	1391.20	1401.46	1401.46	1426.84	1400.62	1400.62
$\omega_{19\mathrm{a}}(e)$	1361.44	1353.29	1361.57	1361.57	1382.69	1362.08	1362.08
$\omega_{19\mathrm{b}}(e)$	1361.44	1353.29	1361.57	1361.57	1382.66	1362.08	1362.08
$\omega_{20\mathrm{a}}(e)$	1199.55	1196.84	1199.61	1199.62	1220.68	1199.63	1199.96
$\omega_{20\mathrm{b}}(e)$	1199.55	1196.83	1199.61	1199.62	1220.67	1199.63	1199.96
$\omega_{21\mathrm{a}}(e)$	984.50	983.83	984.53	984.52	1009.08	985.14	984.74
$\omega_{21\mathrm{b}}(e)$	984.50	983.81	984.53	984.52	1009.07	985.13	984.73
$\omega_{22\mathrm{a}}(e)$	921.91	920.33	921.93	921.93	937.06	922.18	922.18
$\omega_{22\mathrm{b}}(e)$	921.90	920.33	921.92	921.92	937.06	922.18	922.18
$\omega_{23\mathrm{a}}(e)$	357.91	356.10	357.90	357.90	368.03	357.83	357.83
$\omega_{23\mathrm{b}}(e)$	357.90	356.09	357.90	357.90	368.00	357.83	357.83
$\omega_{24\mathrm{a}}(e)$	259.53	263.45	259.56	259.56	276.17	259.70	259.70
$\omega_{24\mathrm{b}}(e)$	259.52	263.45	259.55	259.55	276.16	259.70	259.70

Table S307: CMA-0A and CMA-2A (ξ = 0.02) harmonic frequencies targeting CCSD(T)/cc-pVTZ for Level B = B3LYP/6-31G(2df,p).

	Pure B3LYP/ 6-31G(2df,p)	CMA-0A B3LYP/ 6-31G(2df,p)	CMA-2A B3LYP/ 6-31G(2df,p)
$\omega_1(a_1)$	3112.38	3097.52	3097.52
$\omega_2(a_1)$	3043.43	3023.98	3023.98
$\omega_3(a_1)$	3026.58	3019.87	3019.87
$\omega_4(a_1)$	1519.61	1518.97	1518.97
$\omega_5(a_1)$	1436.77	1425.64	1425.64
$\omega_6(a_1)$	1221.91	1214.05	1214.05
$\omega_7(a_1)$	809.21	809.26	809.26
$\omega_8(a_1)$	435.98	424.94	424.94
$\omega_9(a_2)$	3112.12	3098.76	3098.76
$\omega_{10}(a_2)$	1485.70	1487.99	1487.99
$\omega_{11}(a_2)$	962.00	952.48	952.48
$\omega_{12}(a_2)$	212.59	201.01	201.01
$\omega_{13\mathrm{a}}(e)$	3118.03	3102.59	3102.59
$\omega_{13\mathrm{b}}(e)$	3114.14	3102.54	3102.54
$\omega_{14\mathrm{a}}(e)$	3100.41	3088.22	3088.22
$\omega_{14\mathrm{b}}(e)$	3099.38	3088.12	3088.12
$\omega_{15\mathrm{a}}(e)$	3036.35	3018.85	3018.85
$\omega_{15\mathrm{b}}(e)$	3034.55	3018.83	3018.83
$\omega_{16\mathrm{a}}(e)$	1512.95	1512.22	1512.22
$\omega_{16\mathrm{b}}(e)$	1509.22	1512.10	1512.10
$\omega_{17\mathrm{a}}(e)$	1495.26	1493.75	1493.75
$\omega_{17\mathrm{b}}(e)$	1491.73	1493.60	1493.62
$\omega_{18\mathrm{a}}(e)$	1410.99	1401.06	1401.06
$\omega_{18\mathrm{b}}(e)$	1405.57	1400.69	1400.70
$\omega_{19\mathrm{a}}(e)$	1369.59	1362.05	1362.05
$\omega_{19\mathrm{b}}(e)$	1366.88	1361.82	1361.82
$\omega_{20\mathrm{a}}(e)$	1201.48	1199.45	1199.45
$\omega_{20\mathrm{b}}(e)$	1200.19	1199.35	1199.35
$\omega_{21\mathrm{a}}(e)$	982.40	984.77	984.77
$\omega_{21\mathrm{b}}(e)$	982.00	984.74	984.74
$\omega_{22\mathrm{a}}(e)$	932.51	922.55	922.56
$\omega_{22\mathrm{b}}(e)$	929.36	922.41	922.41
$\omega_{23\mathrm{a}}(e)$	366.54	357.88	358.11
$\omega_{23\mathrm{b}}(e)$	364.90	357.82	357.90
$\omega_{24\mathrm{a}}(e)$	259.22	260.04	259.82
$\omega_{24\mathrm{b}}(e)$	228.24	259.96	259.56

Table S308: Symmetrized, unnormalized natural internal coordinates for isobutane.

1	$r_{1,2}$
2	$r_{2,3} + r_{2,4} + r_{2,5}$
3	$2r_{2,3} - r_{2,4} - r_{2,5}$
4	$r_{2,4} - r_{2,5}$
5	$r_{3,6} + r_{3,7} + r_{3,8} + r_{4,9} + r_{4,10} + r_{4,11} + r_{5,12} + r_{5,13} + r_{5,14}$
6	$2r_{3,6} + 2r_{3,7} + 2r_{3,8} - r_{4,9} - r_{4,10} - r_{4,11} - r_{5,12} - r_{5,13} - r_{5,14}$
7	$r_{4,9} + r_{4,10} + r_{4,11} - r_{5,12} - r_{5,13} - r_{5,14}$
8	$2r_{3,6} - r_{3,7} - r_{3,8} + 2r_{4,9} - r_{4,10} - r_{4,11} + 2r_{5,12} - r_{5,13} - r_{5,14}$
9	$4r_{3,6} - 2r_{3,7} - 2r_{3,8} - 2r_{4,9} + r_{4,10} + r_{4,11} - 2r_{5,12} + r_{5,13} + r_{5,14}$
10	$2r_{4,9} - r_{4,10} - r_{4,11} - 2r_{5,12} + r_{5,13} + r_{5,14}$
11	$r_{3,7} - r_{3,8} + r_{4,10} - r_{4,11} + r_{5,13} - r_{5,14}$
12	$2r_{3,7} - 2r_{3,8} - r_{4,10} + r_{4,11} - r_{5,13} + r_{5,14}$
13	$r_{4,10} - r_{4,11} - r_{5,13} + r_{5,14}$
14	$\phi_{4,2,5} + \phi_{3,2,4} + \phi_{3,2,5} - \phi_{1,2,3} - \phi_{1,2,4} - \phi_{1,2,5}$
15	$2\phi_{4,2,5} - \phi_{3,2,4} - \phi_{3,2,5}$
16	$\phi_{3,2,4} - \phi_{3,2,5}$
17	$2\phi_{1,2,3} - \phi_{1,2,4} - \phi_{1,2,5}$
18	$\phi_{1,2,4} - \phi_{1,2,5}$
19	$\phi_{7,3,8} + \phi_{6,3,7} + \phi_{6,3,8} - \phi_{6,3,2} - \phi_{7,3,2} - \phi_{8,3,2} + \phi_{10,4,11} + \phi_{9,4,10} + \phi_{9,4,11} - \phi_{9,4,2}$ $-\phi_{10,4,2} - \phi_{11,4,2} + \phi_{13,5,14} + \phi_{12,5,13} + \phi_{12,5,14} - \phi_{12,5,2} - \phi_{13,5,2} - \phi_{14,5,2}$
20	$2\phi_{7,3,8} + 2\phi_{6,3,7} + 2\phi_{6,3,8} - 2\phi_{6,3,2} - 2\phi_{7,3,2} - 2\phi_{8,3,2} - \phi_{10,4,11} - \phi_{9,4,10} - \phi_{9,4,11} + \phi_{9,4,2}$ $+\phi_{10,4,2} + \phi_{11,4,2} - \phi_{13,5,14} - \phi_{12,5,13} - \phi_{12,5,14} + \phi_{12,5,2} + \phi_{13,5,2} + \phi_{14,5,2}$
21	$\phi_{10,4,11} + \phi_{9,4,10} + \phi_{9,4,11} - \phi_{9,4,2} - \phi_{10,4,2} - \phi_{11,4,2} - \phi_{13,5,14} - \phi_{12,5,13} - \phi_{12,5,14} + \phi_{12,5,2}$ $+\phi_{13,5,2} + \phi_{14,5,2}$
22	$2\phi_{7,3,8} - \phi_{6,3,7} - \phi_{6,3,8} + 2\phi_{10,4,11} - \phi_{9,4,10} - \phi_{9,4,11} + 2\phi_{13,5,14} - \phi_{12,5,13} - \phi_{12,5,14}$
23	$4\phi_{7,3,8} - 2\phi_{6,3,7} - 2\phi_{6,3,8} - 2\phi_{10,4,11} + \phi_{9,4,10} + \phi_{9,4,11} - 2\phi_{13,5,14} + \phi_{12,5,13} + \phi_{12,5,14}$
24	$2\phi_{10,4,11} - \phi_{9,4,10} - \phi_{9,4,11} - 2\phi_{13,5,14} + \phi_{12,5,13} + \phi_{12,5,14}$
25	$\phi_{6,3,7} - \phi_{6,3,8} + \phi_{9,4,10} - \phi_{9,4,11} + \phi_{12,5,13} - \phi_{12,5,14}$
26	$2\phi_{6,3,7} - 2\phi_{6,3,8} - \phi_{9,4,10} + \phi_{9,4,11} - \phi_{12,5,13} + \phi_{12,5,14}$
27	$\phi_{9,4,10} - \phi_{9,4,11} - \phi_{12,5,13} + \phi_{12,5,14}$
28	$2\phi_{6,3,2} - \phi_{7,3,2} - \phi_{8,3,2} + 2\phi_{9,4,2} - \phi_{10,4,2} - \phi_{11,4,2} + 2\phi_{12,5,2} - \phi_{13,5,2} - \phi_{14,5,2}$
29	$4\phi_{6,3,2} - 2\phi_{7,3,2} - 2\phi_{8,3,2} - 2\phi_{9,4,2} + \phi_{10,4,2} + \phi_{11,4,2} - 2\phi_{12,5,2} + \phi_{13,5,2} + \phi_{14,5,2}$
30	$2\phi_{9,4,2} - \phi_{10,4,2} - \phi_{11,4,2} - 2\phi_{12,5,2} + \phi_{13,5,2} + \phi_{14,5,2}$
31	$\phi_{7,3,2} - \phi_{8,3,2} + \phi_{10,4,2} - \phi_{11,4,2} + \phi_{13,5,2} - \phi_{14,5,2}$
32	$2\phi_{7,3,2} - 2\phi_{8,3,2} - \phi_{10,4,2} + \phi_{11,4,2} - \phi_{13,5,2} + \phi_{14,5,2}$
33	$\phi_{10,4,2} - \phi_{11,4,2} - \phi_{13,5,2} + \phi_{14,5,2}$
34	$\tau_{6,3,2,4} + \tau_{6,3,2,5} + \tau_{7,3,2,4} + \tau_{7,3,2,5} + \tau_{8,3,2,4} + \tau_{8,3,2,5} + \tau_{9,4,2,5} + \tau_{9,4,2,3} + \tau_{10,4,2,5} + \tau_{10,4,2,3}$ $+\tau_{11,4,2,5} + \tau_{11,4,2,3} + \tau_{12,5,2,3} + \tau_{13,5,2,3} + \tau_{14,5,2,3} + \tau_{12,5,2,4} + \tau_{13,5,2,4} + \tau_{14,5,2,4}$
35	$2\tau_{6,3,2,4} + 2\tau_{6,3,2,5} + 2\tau_{7,3,2,4} + 2\tau_{7,3,2,5} + 2\tau_{8,3,2,4} + 2\tau_{8,3,2,5} - \tau_{9,4,2,5} - \tau_{9,4,2,3} - \tau_{10,4,2,5} - \tau_{10,4,2,3}$ $-\tau_{11,4,2,5} - \tau_{11,4,2,3} - \tau_{12,5,2,3} - \tau_{13,5,2,3} - \tau_{14,5,2,3} - \tau_{12,5,2,4} - \tau_{13,5,2,4} - \tau_{14,5,2,4}$
36	$\tau_{9,4,2,5} + \tau_{9,4,2,3} + \tau_{10,4,2,5} + \tau_{10,4,2,3} + \tau_{11,4,2,5} + \tau_{11,4,2,3} - \tau_{12,5,2,3} - \tau_{13,5,2,3} - \tau_{14,5,2,3} - \tau_{12,5,2,4}$ $-\tau_{13,5,2,4} - \tau_{14,5,2,4}$

S4.78 *n*-butane

Table S309: CCSD(T)/cc-pVTZ Optimum Cartesian coordinates (bohr)

1	C	−3.68479476	0.24819108	−0.00000000
2	C	−1.06753027	−0.97316796	−0.00000000
3	C	1.06753027	0.97316796	−0.00000000
4	C	3.68479476	−0.24819108	0.00000000
5	H	−5.18541024	−1.16829556	−0.00000000
6	H	−3.93796232	1.44128610	−1.66759012
7	H	−3.93796232	1.44128610	1.66759012
8	H	−0.86739103	−2.19780833	1.65727692
9	H	−0.86739103	−2.19780833	−1.65727692
10	H	0.86739103	2.19780833	1.65727692
11	H	0.86739103	2.19780833	−1.65727692
12	H	5.18541024	1.16829556	0.00000000
13	H	3.93796232	−1.44128610	−1.66759012
14	H	3.93796232	−1.44128610	1.66759012

Table S310: Reference [CCSD(T)/cc-pVTZ] CMA-0A and CMA-2A ($\xi = 0.02$) harmonic frequencies for Level B = MP2/cc-pVTZ and CCSD(T)/cc-pVDZ.

	Reference CCSD(T)/ cc-pVTZ	Pure MP2/ cc-pVTZ	CMA-0A MP2/ cc-pVTZ	CMA-2A MP2/ cc-pVTZ	Pure CCSD(T)/ cc-pVDZ	CMA-0A CCSD(T)/ cc-pVDZ	CMA-2A CCSD(T)/ cc-pVDZ
$\omega_1(a_g)$	3105.51	3130.85	3105.48	3105.48	3254.39	3105.40	3105.40
$\omega_2(a_g)$	3028.91	3041.16	3028.78	3028.78	3162.80	3028.14	3028.14
$\omega_3(a_g)$	3016.77	3032.41	3016.93	3016.93	3152.35	3017.53	3017.53
$\omega_4(a_g)$	1511.27	1513.15	1511.14	1511.14	1523.44	1511.04	1511.04
$\omega_5(a_g)$	1490.65	1489.39	1490.72	1490.72	1504.85	1490.62	1490.62
$\omega_6(a_g)$	1412.55	1403.95	1411.89	1411.89	1441.47	1409.68	1410.20
$\omega_7(a_g)$	1398.23	1385.10	1398.90	1398.90	1421.98	1400.78	1400.78
$\omega_8(a_g)$	1175.12	1172.34	1175.16	1175.16	1192.43	1175.09	1175.18
$\omega_9(a_g)$	1082.98	1082.83	1083.02	1083.02	1114.33	1083.91	1083.23
$\omega_{10}(a_g)$	848.71	848.58	848.73	848.73	871.43	848.97	848.85
$\omega_{11}(a_g)$	422.71	421.23	422.72	422.72	431.98	422.78	422.77
$\omega_{12}(b_g)$	3097.42	3123.34	3097.42	3097.42	3247.91	3097.34	3097.34
$\omega_{13}(b_g)$	3042.42	3069.35	3042.42	3042.42	3191.81	3042.47	3042.47
$\omega_{14}(b_g)$	1505.13	1507.68	1505.12	1505.12	1516.25	1505.17	1505.17
$\omega_{15}(b_g)$	1332.59	1332.31	1332.59	1332.59	1347.18	1332.58	1332.58
$\omega_{16}(b_g)$	1213.01	1207.91	1213.03	1213.03	1227.19	1213.02	1213.02
$\omega_{17}(b_g)$	807.44	807.23	807.46	807.46	821.57	807.48	807.48
$\omega_{18}(b_g)$	257.32	261.41	257.33	257.32	274.55	257.37	257.37
$\omega_{19}(a_u)$	3101.23	3126.12	3101.21	3101.21	3250.80	3101.01	3101.01
$\omega_{20}(a_u)$	3064.03	3090.75	3064.05	3064.05	3211.56	3064.21	3064.21
$\omega_{21}(a_u)$	1506.70	1509.55	1506.69	1506.69	1517.71	1506.74	1506.74
$\omega_{22}(a_u)$	1290.42	1288.70	1290.43	1290.43	1305.47	1290.43	1290.43
$\omega_{23}(a_u)$	957.85	957.29	957.87	957.87	970.81	957.87	957.87
$\omega_{24}(a_u)$	733.72	734.90	733.73	733.73	750.06	733.76	733.77
$\omega_{25}(a_u)$	221.77	224.72	221.78	221.77	236.43	221.80	221.78
$\omega_{26}(a_u)$	115.69	117.41	115.69	115.69	124.02	115.76	115.74
$\omega_{27}(b_u)$	3106.19	3131.39	3106.16	3106.16	3255.01	3106.07	3106.07
$\omega_{28}(b_u)$	3028.05	3042.96	3026.26	3026.26	3160.36	3027.03	3027.03
$\omega_{29}(b_u)$	3023.91	3039.18	3025.73	3025.73	3157.46	3024.96	3024.96
$\omega_{30}(b_u)$	1515.99	1516.15	1515.76	1515.76	1527.68	1515.99	1515.99
$\omega_{31}(b_u)$	1496.91	1497.32	1497.13	1497.13	1508.92	1496.89	1496.89
$\omega_{32}(b_u)$	1414.03	1406.14	1413.99	1413.99	1436.14	1413.88	1413.88
$\omega_{33}(b_u)$	1318.77	1311.28	1318.79	1318.79	1335.23	1318.82	1318.82
$\omega_{34}(b_u)$	1032.28	1031.42	1032.31	1032.31	1063.50	1032.28	1032.69
$\omega_{35}(b_u)$	977.87	976.61	977.89	977.88	992.01	978.33	977.90
$\omega_{36}(b_u)$	253.43	251.97	253.43	253.43	260.21	253.44	253.44

Table S311: CMA-0A and CMA-2A (ξ = 0.02) harmonic frequencies targeting CCSD(T)/cc-pVTZ for Level B = B3LYP/6-31G(2*df*,*p*).

	Pure B3LYP/ 6-31G(2*df*,*p*)	CMA-0A B3LYP/ 6-31G(2*df*,*p*)	CMA-2A B3LYP/ 6-31G(2*df*,*p*)
$\omega_1(a_g)$	3119.95	3105.50	3105.50
$\omega_2(a_g)$	3047.82	3028.84	3028.84
$\omega_3(a_g)$	3027.92	3016.80	3016.80
$\omega_4(a_g)$	1511.01	1511.19	1511.19
$\omega_5(a_g)$	1491.13	1490.70	1490.70
$\omega_6(a_g)$	1419.59	1410.75	1410.75
$\omega_7(a_g)$	1404.25	1399.41	1399.41
$\omega_8(a_g)$	1178.22	1175.15	1175.15
$\omega_9(a_g)$	1074.20	1083.75	1083.75
$\omega_{10}(a_g)$	849.70	848.90	848.90
$\omega_{11}(a_g)$	429.24	422.83	422.83
$\omega_{12}(b_g)$	3112.66	3097.41	3097.41
$\omega_{13}(b_g)$	3051.73	3042.42	3042.42
$\omega_{14}(b_g)$	1505.39	1505.11	1505.11
$\omega_{15}(b_g)$	1338.03	1332.62	1332.62
$\omega_{16}(b_g)$	1220.81	1213.03	1213.03
$\omega_{17}(b_g)$	815.49	807.47	807.48
$\omega_{18}(b_g)$	261.19	257.38	257.35
$\omega_{19}(a_u)$	3116.68	3101.21	3101.21
$\omega_{20}(a_u)$	3074.58	3064.04	3064.04
$\omega_{21}(a_u)$	1506.81	1506.70	1506.70
$\omega_{22}(a_u)$	1296.36	1290.43	1290.43
$\omega_{23}(a_u)$	966.76	957.85	957.85
$\omega_{24}(a_u)$	740.61	733.75	733.75
$\omega_{25}(a_u)$	223.81	221.83	221.86
$\omega_{26}(a_u)$	122.05	115.86	115.81
$\omega_{27}(b_u)$	3120.84	3106.18	3106.18
$\omega_{28}(b_u)$	3047.18	3028.03	3028.03
$\omega_{29}(b_u)$	3035.70	3023.91	3023.91
$\omega_{30}(b_u)$	1519.46	1515.82	1515.82
$\omega_{31}(b_u)$	1496.02	1497.02	1497.02
$\omega_{32}(b_u)$	1421.08	1413.92	1413.92
$\omega_{33}(b_u)$	1332.82	1318.76	1318.76
$\omega_{34}(b_u)$	1027.25	1031.94	1032.51
$\omega_{35}(b_u)$	986.10	978.59	978.00
$\omega_{36}(b_u)$	261.68	253.49	253.49

Table S312: Symmetrized, unnormalized natural internal coordinates for n-butane.

1	$r_{1,2} + r_{3,4}$
2	$r_{1,2} - r_{3,4}$
3	$r_{2,3}$
4	$r_{2,8} + r_{2,9} + r_{3,10} + r_{3,11}$
5	$r_{2,8} + r_{2,9} - r_{3,10} - r_{3,11}$
6	$r_{2,8} - r_{2,9} + r_{3,10} - r_{3,11}$
7	$r_{2,8} - r_{2,9} - r_{3,10} + r_{3,11}$
8	$r_{1,5} + r_{1,6} + r_{1,7} + r_{4,12} + r_{4,13} + r_{4,14}$
9	$r_{1,5} + r_{1,6} + r_{1,7} - r_{4,12} - r_{4,13} - r_{4,14}$
10	$2r_{1,5} - r_{1,6} - r_{1,7} + 2r_{4,12} - r_{4,13} - r_{4,14}$
11	$2r_{1,5} - r_{1,6} - r_{1,7} - 2r_{4,12} + r_{4,13} + r_{4,14}$
12	$r_{1,6} - r_{1,7} + r_{4,13} - r_{4,14}$
13	$r_{1,6} - r_{1,7} - r_{4,13} + r_{4,14}$
14	$\phi_{1,2,3} + \phi_{2,3,4}$
15	$\phi_{1,2,3} - \phi_{2,3,4}$
16	$4\phi_{8,2,9} - \phi_{8,2,1} - \phi_{8,2,3} - \phi_{9,2,1} - \phi_{9,2,3} + 4\phi_{10,3,11} - \phi_{10,3,4} - \phi_{10,3,2} - \phi_{11,3,4} - \phi_{11,3,2}$
17	$4\phi_{8,2,9} - \phi_{8,2,1} - \phi_{8,2,3} - \phi_{9,2,1} - \phi_{9,2,3} - 4\phi_{10,3,11} + \phi_{10,3,4} + \phi_{10,3,2} + \phi_{11,3,4} + \phi_{11,3,2}$
18	$\phi_{8,2,1} + \phi_{8,2,3} - \phi_{9,2,1} - \phi_{9,2,3} + \phi_{10,3,4} + \phi_{10,3,2} - \phi_{11,3,4} - \phi_{11,3,2}$
19	$\phi_{8,2,1} + \phi_{8,2,3} - \phi_{9,2,1} - \phi_{9,2,3} - \phi_{10,3,4} - \phi_{10,3,2} + \phi_{11,3,4} + \phi_{11,3,2}$
20	$\phi_{8,2,1} - \phi_{8,2,3} + \phi_{9,2,1} - \phi_{9,2,3} + \phi_{10,3,4} - \phi_{10,3,2} + \phi_{11,3,4} - \phi_{11,3,2}$
21	$\phi_{8,2,1} - \phi_{8,2,3} + \phi_{9,2,1} - \phi_{9,2,3} - \phi_{10,3,4} + \phi_{10,3,2} - \phi_{11,3,4} + \phi_{11,3,2}$
22	$\phi_{8,2,1} - \phi_{8,2,3} - \phi_{9,2,1} + \phi_{9,2,3} + \phi_{10,3,4} - \phi_{10,3,2} - \phi_{11,3,4} + \phi_{11,3,2}$
23	$\phi_{8,2,1} - \phi_{8,2,3} - \phi_{9,2,1} + \phi_{9,2,3} - \phi_{10,3,4} + \phi_{10,3,2} + \phi_{11,3,4} - \phi_{11,3,2}$
24	$\phi_{5,1,2} + \phi_{6,1,2} + \phi_{7,1,2} - \phi_{6,1,7} - \phi_{5,1,6} - \phi_{5,1,7} + \phi_{12,4,3} + \phi_{13,4,3} + \phi_{14,4,3} - \phi_{13,4,14}$ $-\phi_{12,4,13} - \phi_{12,4,14}$
25	$\phi_{5,1,2} + \phi_{6,1,2} + \phi_{7,1,2} - \phi_{6,1,7} - \phi_{5,1,6} - \phi_{5,1,7} - \phi_{12,4,3} - \phi_{13,4,3} - \phi_{14,4,3} + \phi_{13,4,14}$ $+\phi_{12,4,13} + \phi_{12,4,14}$
26	$2\phi_{5,1,2} - \phi_{6,1,2} - \phi_{7,1,2} + 2\phi_{12,4,3} - \phi_{13,4,3} - \phi_{14,4,3}$
27	$2\phi_{5,1,2} - \phi_{6,1,2} - \phi_{7,1,2} - 2\phi_{12,4,3} + \phi_{13,4,3} + \phi_{14,4,3}$
28	$\phi_{6,1,2} - \phi_{7,1,2} + \phi_{13,4,3} - \phi_{14,4,3}$
29	$\phi_{6,1,2} - \phi_{7,1,2} - \phi_{13,4,3} + \phi_{14,4,3}$
30	$2\phi_{6,1,7} - \phi_{5,1,6} - \phi_{5,1,7} + 2\phi_{13,4,14} - \phi_{12,4,13} - \phi_{12,4,14}$
31	$2\phi_{6,1,7} - \phi_{5,1,6} - \phi_{5,1,7} - 2\phi_{13,4,14} + \phi_{12,4,13} + \phi_{12,4,14}$
32	$\phi_{5,1,6} - \phi_{5,1,7} + \phi_{12,4,13} - \phi_{12,4,14}$
33	$\phi_{5,1,6} - \phi_{5,1,7} - \phi_{12,4,13} + \phi_{12,4,14}$
34	$\tau_{1,2,3,4}$
35	$\tau_{5,1,2,3} + \tau_{6,1,2,3} + \tau_{7,1,2,3} + \tau_{12,4,3,2} + \tau_{13,4,3,2} + \tau_{14,4,3,2}$
36	$\tau_{5,1,2,3} + \tau_{6,1,2,3} + \tau_{7,1,2,3} - \tau_{12,4,3,2} - \tau_{13,4,3,2} - \tau_{14,4,3,2}$

S4.79 furan

Table S313: CCSD(T)/cc-pVTZ Optimum Cartesian coordinates (bohr)

1	X	0.00000000	0.00000000	−0.25784671
2	O	0.00000000	0.00000000	−2.14757323
3	C	−0.00000000	2.06569237	−0.60265967
4	C	−0.00000000	−2.06569237	−0.60265967
5	C	−0.00000000	−1.36012563	1.87072136
6	C	0.00000000	1.36012563	1.87072136
7	H	0.00000000	3.87067042	−1.53834798
8	H	−0.00000000	−3.87067042	−1.53834798
9	H	0.00000000	−2.60420194	3.48152751
10	H	0.00000000	2.60420194	3.48152751

Table S314: Reference [CCSD(T)/cc-pVTZ] CMA-0A and CMA-2A ($\xi = 0.02$) harmonic frequencies for Level B = MP2/cc-pVTZ and CCSD(T)/cc-pVDZ.

	Reference CCSD(T)/ cc-pVTZ	Pure MP2/ cc-pVTZ	CMA-0A MP2/ cc-pVTZ	CMA-2A MP2/ cc-pVTZ	Pure CCSD(T)/ cc-pVDZ	CMA-0A CCSD(T)/ cc-pVDZ	CMA-2A CCSD(T)/ cc-pVDZ
$\omega_1(a_1)$	3295.05	3310.29	3295.04	3295.04	3436.78	3295.04	3295.04
$\omega_2(a_1)$	3268.39	3284.34	3268.39	3268.39	3408.99	3268.39	3268.39
$\omega_3(a_1)$	1524.35	1512.97	1524.25	1524.34	1584.70	1521.43	1524.33
$\omega_4(a_1)$	1416.47	1398.89	1416.52	1416.46	1460.40	1418.00	1416.07
$\omega_5(a_1)$	1160.71	1156.62	1160.70	1160.69	1184.72	1160.28	1160.68
$\omega_6(a_1)$	1090.49	1093.31	1090.46	1090.49	1115.85	1092.32	1090.83
$\omega_7(a_1)$	1012.31	1009.76	1012.43	1012.35	1029.31	1012.90	1012.37
$\omega_8(a_1)$	877.52	872.04	877.55	877.55	888.51	877.76	877.76
$\omega_9(a_2)$	860.43	860.49	860.16	860.43	861.40	860.14	860.43
$\omega_{10}(a_2)$	731.42	727.34	731.57	731.33	744.94	731.39	731.39
$\omega_{11}(a_2)$	603.17	607.85	603.36	603.27	602.42	603.61	603.21
$\omega_{12}(b_1)$	844.41	838.39	844.34	844.41	852.79	844.39	844.41
$\omega_{13}(b_1)$	759.65	759.45	759.66	759.65	768.37	759.63	759.63
$\omega_{14}(b_1)$	612.43	624.20	612.52	612.43	619.31	612.49	612.46
$\omega_{15}(b_2)$	3287.59	3303.23	3287.57	3287.57	3429.25	3287.59	3287.59
$\omega_{16}(b_2)$	3257.60	3274.66	3257.62	3257.62	3397.51	3257.60	3257.60
$\omega_{17}(b_2)$	1591.82	1583.30	1591.71	1591.80	1654.25	1591.27	1591.63
$\omega_{18}(b_2)$	1289.60	1285.69	1289.59	1289.61	1303.68	1288.81	1289.60
$\omega_{19}(b_2)$	1217.77	1226.87	1217.73	1217.77	1242.49	1218.84	1217.80
$\omega_{20}(b_2)$	1061.14	1060.69	1061.35	1061.14	1080.63	1061.68	1061.38
$\omega_{21}(b_2)$	882.50	876.44	882.52	882.52	890.67	882.56	882.56

Table S315: CMA-0A and CMA-2A (ξ = 0.02) harmonic frequencies targeting CCSD(T)/cc-pVTZ for Level B = B3LYP/6-31G(2*df*,*p*).

	Pure B3LYP/ 6-31G(2*df*,*p*)	CMA-0A B3LYP/ 6-31G(2*df*,*p*)	CMA-2A B3LYP/ 6-31G(2*df*,*p*)
$\omega_1(a_1)$	3307.63	3294.74	3294.74
$\omega_2(a_1)$	3276.88	3268.68	3268.68
$\omega_3(a_1)$	1508.42	1523.75	1523.99
$\omega_4(a_1)$	1405.22	1416.69	1416.69
$\omega_5(a_1)$	1162.48	1160.86	1160.69
$\omega_6(a_1)$	1086.88	1090.69	1090.63
$\omega_7(a_1)$	1014.30	1012.45	1012.34
$\omega_8(a_1)$	880.50	877.67	877.67
$\omega_9(a_2)$	879.09	860.30	860.43
$\omega_{10}(a_2)$	740.22	731.39	731.36
$\omega_{11}(a_2)$	613.69	603.38	603.25
$\omega_{12}(b_1)$	847.30	844.19	844.41
$\omega_{13}(b_1)$	767.24	759.84	759.59
$\omega_{14}(b_1)$	622.50	612.51	612.51
$\omega_{15}(b_2)$	3300.94	3287.37	3287.37
$\omega_{16}(b_2)$	3266.59	3257.80	3257.80
$\omega_{17}(b_2)$	1589.28	1591.58	1591.65
$\omega_{18}(b_2)$	1283.72	1289.55	1289.55
$\omega_{19}(b_2)$	1204.02	1216.95	1217.94
$\omega_{20}(b_2)$	1060.81	1062.59	1061.35
$\omega_{21}(b_2)$	886.69	882.51	882.51

Table S316: Symmetrized, unnormalized natural internal coordinates for furan.

1	$r_{5,6} + r_{3,6} + r_{4,5} + r_{2,3} + r_{2,4}$
2	$3r_{5,6} + r_{3,6} + r_{4,5} - 3r_{2,3} - 3r_{2,4}$
3	$2r_{3,6} - 2r_{4,5} + r_{2,3} - r_{2,4}$
4	$3r_{5,6} - 3r_{3,6} - 3r_{4,5} + r_{2,3} + r_{2,4}$
5	$r_{3,6} - r_{4,5} - 2r_{2,3} + 2r_{2,4}$
6	$r_{3,7} + r_{4,8}$
7	$r_{3,7} - r_{4,8}$
8	$r_{6,10} + r_{5,9}$
9	$r_{6,10} - r_{5,9}$
10	$3\phi_{3,2,4} - 3\phi_{2,4,5} + \phi_{4,5,6} + \phi_{5,6,3} - 3\phi_{6,3,2}$
11	$-\phi_{2,4,5} + 2\phi_{4,5,6} - 2\phi_{5,6,3} + \phi_{6,3,2}$
12	$\phi_{7,3,2} - \phi_{7,3,6} + \phi_{8,4,2} - \phi_{8,4,5}$
13	$\phi_{7,3,2} - \phi_{7,3,6} - \phi_{8,4,2} + \phi_{8,4,5}$
14	$\phi_{10,6,3} - \phi_{10,6,5} + \phi_{9,5,4} - \phi_{9,5,6}$
15	$\phi_{10,6,3} - \phi_{10,6,5} - \phi_{9,5,4} + \phi_{9,5,6}$
16	$\tau_{3,2,4,5} - 3\tau_{2,4,5,6} + 3\tau_{4,5,6,3} - 3\tau_{5,6,3,2} + \tau_{6,3,2,4}$
17	$-2\tau_{3,2,4,5} + \tau_{2,4,5,6} - \tau_{5,6,3,2} + 2\tau_{6,3,2,4}$
18	$\tau_{7,3,2,4} + \tau_{8,4,2,3}$
19	$\tau_{10,6,5,9}$
20	$\gamma_{7,3,2,6} + \gamma_{8,4,5,2}$
21	$\gamma_{10,6,3,5} + \gamma_{9,5,6,4}$

S4.80 1,3−butadiene

Table S317: CCSD(T)/cc-pVTZ Optimum Cartesian coordinates (bohr)

1	C	0.14744698	−2.89273316	0.96440626
2	C	−0.23148527	−1.37327643	−1.03135868
3	C	0.23148527	1.37327643	−1.03135868
4	C	−0.14744698	2.89273316	0.96440626
5	H	0.89800063	−2.16342975	2.72536418
6	H	−0.26186351	−4.89440210	0.86513287
7	H	−0.89144700	−2.19256181	−2.79330602
8	H	0.89144700	2.19256181	−2.79330602
9	H	−0.89800063	2.16342975	2.72536418
10	H	0.26186351	4.89440210	0.86513287

Table S318: Reference [CCSD(T)/cc-pVTZ] CMA-0A and CMA-2A ($\xi = 0.02$) harmonic frequencies for Level B = MP2/cc-pVTZ and CCSD(T)/cc-pVDZ.

	Reference CCSD(T)/ cc-pVTZ	Pure MP2/ cc-pVTZ	CMA-0A MP2/ cc-pVTZ	CMA-2A MP2/ cc-pVTZ	Pure CCSD(T)/ cc-pVDZ	CMA-0A CCSD(T)/ cc-pVDZ	CMA-2A CCSD(T)/ cc-pVDZ
$\omega_1(a)$	3238.88	3260.07	3238.87	3238.87	3392.25	3238.83	3238.83
$\omega_2(a)$	3164.73	3181.75	3164.69	3164.69	3307.36	3164.76	3164.76
$\omega_3(a)$	3145.60	3161.34	3145.63	3145.63	3286.20	3145.59	3145.59
$\omega_4(a)$	1666.75	1656.10	1666.68	1666.73	1738.58	1665.85	1666.19
$\omega_5(a)$	1468.45	1465.89	1468.37	1468.37	1492.91	1468.95	1468.95
$\omega_6(a)$	1333.34	1330.94	1333.51	1333.43	1355.94	1333.66	1333.24
$\omega_7(a)$	1058.55	1056.31	1058.29	1058.29	1081.76	1058.38	1058.59
$\omega_8(a)$	998.56	1010.88	998.70	998.82	1010.54	998.63	998.63
$\omega_9(a)$	927.69	933.10	927.67	927.67	935.55	927.60	927.71
$\omega_{10}(a)$	886.41	884.96	886.50	886.49	914.83	886.95	886.65
$\omega_{11}(a)$	742.41	753.07	742.58	742.43	747.07	742.55	742.50
$\omega_{12}(a)$	271.87	267.75	271.84	271.88	279.55	271.80	271.93
$\omega_{13}(a)$	164.78	173.69	164.89	164.82	162.65	165.02	164.80
$\omega_{14}(b)$	3237.22	3258.54	3237.21	3237.21	3390.52	3237.18	3237.18
$\omega_{15}(b)$	3152.06	3169.92	3151.78	3151.78	3294.37	3152.08	3152.08
$\omega_{16}(b)$	3143.07	3159.12	3143.34	3143.34	3283.28	3143.07	3143.07
$\omega_{17}(b)$	1674.83	1666.91	1674.81	1674.83	1734.81	1674.04	1674.55
$\omega_{18}(b)$	1437.31	1435.30	1437.28	1437.28	1456.07	1437.42	1437.42
$\omega_{19}(b)$	1301.44	1299.57	1301.44	1301.42	1323.21	1302.17	1301.52
$\omega_{20}(b)$	1098.92	1095.22	1098.97	1098.97	1117.05	1099.10	1099.10
$\omega_{21}(b)$	1017.20	1032.35	1017.18	1017.21	1028.67	1017.19	1017.19
$\omega_{22}(b)$	929.55	934.49	929.55	929.55	937.35	929.53	929.53
$\omega_{23}(b)$	610.05	611.18	609.97	609.94	619.02	610.07	610.16
$\omega_{24}(b)$	466.84	468.31	467.02	467.01	471.86	467.00	466.88

Table S319: CMA-0A and CMA-2A (ξ = 0.02) harmonic frequencies targeting CCSD(T)/cc-pVTZ for Level B = B3LYP/6-31G(2df,p).

	Pure B3LYP/ 6-31G(2df,p)	CMA-0A B3LYP/ 6-31G(2df,p)	CMA-2A B3LYP/ 6-31G(2df,p)
$\omega_1(a)$	3253.35	3238.87	3238.87
$\omega_2(a)$	3175.72	3164.72	3164.72
$\omega_3(a)$	3157.69	3145.58	3145.58
$\omega_4(a)$	1650.47	1666.26	1666.69
$\omega_5(a)$	1459.96	1468.32	1468.32
$\omega_6(a)$	1335.64	1333.74	1333.43
$\omega_7(a)$	1061.63	1057.50	1057.50
$\omega_8(a)$	1014.80	999.42	999.42
$\omega_9(a)$	943.49	927.68	927.68
$\omega_{10}(a)$	880.14	887.22	886.89
$\omega_{11}(a)$	754.52	742.55	742.55
$\omega_{12}(a)$	277.07	271.67	271.93
$\omega_{13}(a)$	175.19	165.30	164.87
$\omega_{14}(b)$	3251.65	3237.21	3237.21
$\omega_{15}(b)$	3161.68	3152.03	3152.03
$\omega_{16}(b)$	3155.55	3143.08	3143.08
$\omega_{17}(b)$	1675.84	1674.57	1674.68
$\omega_{18}(b)$	1432.18	1437.41	1437.41
$\omega_{19}(b)$	1304.78	1301.58	1301.43
$\omega_{20}(b)$	1101.24	1098.97	1098.97
$\omega_{21}(b)$	1031.13	1017.08	1017.08
$\omega_{22}(b)$	944.70	929.70	929.70
$\omega_{23}(b)$	618.71	610.10	610.10
$\omega_{24}(b)$	470.79	466.98	466.98

Table S320: Symmetrized, unnormalized natural internal coordinates for 1,3−butadiene.

1	$r_{1,2} + r_{3,4}$
2	$r_{1,2} - r_{3,4}$
3	$r_{2,3}$
4	$r_{1,5} + r_{1,6} + r_{4,9} + r_{4,10}$
5	$r_{1,5} + r_{1,6} - r_{4,9} - r_{4,10}$
6	$r_{1,5} - r_{1,6} + r_{4,9} - r_{4,10}$
7	$r_{1,5} - r_{1,6} - r_{4,9} + r_{4,10}$
8	$r_{2,7} + r_{3,8}$
9	$r_{2,7} - r_{3,8}$
10	$\phi_{1,2,3} + \phi_{2,3,4}$
11	$\phi_{1,2,3} - \phi_{2,3,4}$
12	$2\phi_{5,1,6} - \phi_{5,1,2} - \phi_{6,1,2} + 2\phi_{9,4,10} - \phi_{9,4,3} - \phi_{10,4,3}$
13	$2\phi_{5,1,6} - \phi_{5,1,2} - \phi_{6,1,2} - 2\phi_{9,4,10} + \phi_{9,4,3} + \phi_{10,4,3}$
14	$\phi_{5,1,2} - \phi_{6,1,2} + \phi_{9,4,3} - \phi_{10,4,3}$
15	$\phi_{5,1,2} - \phi_{6,1,2} - \phi_{9,4,3} + \phi_{10,4,3}$
16	$\phi_{7,2,1} - \phi_{7,2,3} + \phi_{8,3,4} - \phi_{8,3,2}$
17	$\phi_{7,2,1} - \phi_{7,2,3} - \phi_{8,3,4} + \phi_{8,3,2}$
18	$\tau_{1,2,3,4}$
19	$\tau_{5,1,2,3} + \tau_{6,1,2,3} + \tau_{9,4,3,2} + \tau_{10,4,3,2}$
20	$\tau_{5,1,2,3} + \tau_{6,1,2,3} - \tau_{9,4,3,2} - \tau_{10,4,3,2}$
21	$\gamma_{2,1,5,6} + \gamma_{3,4,9,10}$
22	$\gamma_{2,1,5,6} - \gamma_{3,4,9,10}$
23	$\gamma_{7,2,1,3} + \gamma_{8,3,2,4}$
24	$\gamma_{7,2,1,3} - \gamma_{8,3,2,4}$

S4.81 2-butyne

Table S321: CCSD(T)/cc-pVTZ Optimum Cartesian coordinates (bohr)

1	C	−0.00000000	1.14516104	−0.00000003
2	C	0.00000000	−1.14516104	−0.00000003
3	C	−0.00000000	3.91831350	−0.00000003
4	C	0.00000000	−3.91831350	−0.00000003
5	H	1.67000200	4.64975861	−0.96417610
6	H	−1.67000200	4.64975861	−0.96417610
7	H	−0.00000000	4.64975861	1.92835298
8	H	0.00000000	−4.64975861	1.92835298
9	H	1.67000200	−4.64975861	−0.96417610
10	H	−1.67000200	−4.64975861	−0.96417610

Table S322: Reference [CCSD(T)/cc-pVTZ] CMA-0A and CMA-2A ($\xi = 0.02$) harmonic frequencies for Level B = MP2/cc-pVTZ and CCSD(T)/cc-pVDZ.

	Reference CCSD(T)/ cc-pVTZ	Pure MP2/ cc-pVTZ	CMA-0A MP2/ cc-pVTZ	CMA-2A MP2/ cc-pVTZ	Pure CCSD(T)/ cc-pVDZ	CMA-0A CCSD(T)/ cc-pVDZ	CMA-2A CCSD(T)/ cc-pVDZ
$\omega_1(a_1^{'})$	3042.19	3054.20	3042.17	3042.17	3171.56	3042.11	3042.11
$\omega_2(a_1^{'})$	2324.07	2302.31	2324.08	2324.08	2424.81	2324.11	2324.11
$\omega_3(a_1^{'})$	1420.58	1413.47	1420.60	1420.60	1441.38	1420.60	1420.60
$\omega_4(a_1^{'})$	716.44	714.98	716.45	716.45	746.51	716.60	716.60
$\omega_{5a}(e^{'})$	3113.29	3136.96	3113.29	3113.29	3261.92	3113.25	3113.25
$\omega_{5b}(e^{'})$	3113.29	3136.91	3113.29	3113.29	3261.86	3113.25	3113.25
$\omega_{6a}(e^{'})$	1493.16	1495.35	1493.15	1493.15	1502.46	1493.21	1493.21
$\omega_{6b}(e^{'})$	1493.15	1495.33	1493.14	1493.14	1502.44	1493.20	1493.20
$\omega_{7a}(e^{'})$	1067.93	1065.65	1067.95	1067.95	1081.94	1067.97	1067.97
$\omega_{7b}(e^{'})$	1067.93	1065.64	1067.95	1067.95	1081.93	1067.96	1067.96
$\omega_{8a}(e^{'})$	197.35	197.41	197.35	197.35	199.15	197.35	197.35
$\omega_{8b}(e^{'})$	197.34	197.41	197.35	197.35	199.15	197.35	197.35
$\omega_9(a_1^{''})$	18.84	19.93	18.84	18.84	19.61	18.84	18.84
$\omega_{10}(a_2^{''})$	3042.56	3055.26	3042.56	3042.56	3171.62	3042.51	3042.51
$\omega_{11}(a_2^{''})$	1415.88	1408.81	1415.87	1415.87	1439.87	1415.12	1415.12
$\omega_{12}(a_2^{''})$	1167.27	1165.94	1167.28	1167.28	1211.81	1168.31	1168.31
$\omega_{13a}(e^{''})$	3113.85	3137.67	3113.84	3113.84	3262.35	3113.81	3113.81
$\omega_{13b}(e^{''})$	3113.84	3137.61	3113.84	3113.84	3262.30	3113.81	3113.81
$\omega_{14a}(e^{''})$	1492.12	1494.22	1492.11	1492.11	1502.12	1492.16	1492.16
$\omega_{14b}(e^{''})$	1492.11	1494.21	1492.10	1492.10	1502.11	1492.16	1492.16
$\omega_{15a}(e^{''})$	1046.04	1046.55	1046.05	1046.06	1059.64	1046.08	1046.08
$\omega_{15b}(e^{''})$	1046.03	1046.54	1046.05	1046.05	1059.63	1046.08	1046.08
$\omega_{16a}(e^{''})$	348.82	354.29	348.82	348.81	352.81	348.84	348.83
$\omega_{16b}(e^{''})$	348.80	354.25	348.82	348.81	352.78	348.84	348.83

Table S323: CMA-0A and CMA-2A (ξ = 0.02) harmonic frequencies targeting CCSD(T)/cc-pVTZ for Level B = B3LYP/6-31G(2df,p).

	Pure B3LYP/ 6-31G(2df,p)	CMA-0A B3LYP/ 6-31G(2df,p)	CMA-2A B3LYP/ 6-31G(2df,p)
$\omega_1(a_1^{'})$	3059.53	3042.02	3042.02
$\omega_2(a_1^{'})$	2324.25	2324.07	2324.17
$\omega_3(a_1^{'})$	1419.21	1420.71	1420.71
$\omega_4(a_1^{'})$	708.67	716.91	716.58
$\omega_{5\mathrm{a}}(e^{'})$	3125.31	3113.29	3113.29
$\omega_{5\mathrm{b}}(e^{'})$	3125.23	3113.28	3113.28
$\omega_{6\mathrm{a}}(e^{'})$	1483.19	1493.09	1493.09
$\omega_{6\mathrm{b}}(e^{'})$	1483.12	1493.07	1493.07
$\omega_{7\mathrm{a}}(e^{'})$	1068.90	1068.04	1068.04
$\omega_{7\mathrm{b}}(e^{'})$	1068.88	1068.04	1068.04
$\omega_{8\mathrm{a}}(e^{'})$	200.10	197.36	197.36
$\omega_{8\mathrm{b}}(e^{'})$	17.59	18.84	18.84
$\omega_9(a_1^{''})$	200.08	197.36	197.36
$\omega_{10}(a_2^{''})$	3059.68	3042.52	3042.52
$\omega_{11}(a_2^{''})$	1411.48	1415.14	1415.14
$\omega_{12}(a_2^{''})$	1141.73	1168.27	1168.27
$\omega_{13\mathrm{a}}(e^{''})$	3125.85	3113.84	3113.84
$\omega_{13\mathrm{b}}(e^{''})$	3125.77	3113.84	3113.84
$\omega_{14\mathrm{a}}(e^{''})$	1482.77	1492.04	1492.04
$\omega_{14\mathrm{b}}(e^{''})$	1482.76	1492.04	1492.04
$\omega_{15\mathrm{a}}(e^{''})$	1043.84	1046.12	1046.14
$\omega_{15\mathrm{b}}(e^{''})$	1043.82	1046.12	1046.14
$\omega_{16\mathrm{a}}(e^{''})$	369.42	348.92	348.86
$\omega_{16\mathrm{b}}(e^{''})$	369.33	348.91	348.85

Table S324: Symmetrized, unnormalized natural internal coordinates for 2-butyne.

1	$r_{1,2}$
2	$r_{1,3} + r_{2,4}$
3	$r_{1,3} - r_{2,4}$
4	$r_{3,6} + r_{3,5} + r_{3,7} + r_{4,10} + r_{4,9} + r_{4,8}$
5	$r_{3,6} + r_{3,5} + r_{3,7} - r_{4,10} - r_{4,9} - r_{4,8}$
6	$2r_{3,6} - r_{3,5} - r_{3,7} + 2r_{4,10} - r_{4,9} - r_{4,8}$
7	$2r_{3,6} - r_{3,5} - r_{3,7} - 2r_{4,10} + r_{4,9} + r_{4,8}$
8	$r_{3,5} - r_{3,7} + r_{4,9} - r_{4,8}$
9	$r_{3,5} - r_{3,7} - r_{4,9} + r_{4,8}$
10	$\phi_{6,3,1} + \phi_{5,3,1} + \phi_{7,3,1} - \phi_{5,3,7} - \phi_{6,3,5} - \phi_{6,3,7} + \phi_{10,4,2} + \phi_{9,4,2} + \phi_{8,4,2} - \phi_{8,4,9}$ $-\phi_{10,4,9} - \phi_{10,4,8}$
11	$\phi_{6,3,1} + \phi_{5,3,1} + \phi_{7,3,1} - \phi_{5,3,7} - \phi_{6,3,5} - \phi_{6,3,7} - \phi_{10,4,2} - \phi_{9,4,2} - \phi_{8,4,2} + \phi_{8,4,9}$ $+\phi_{10,4,9} + \phi_{10,4,8}$
12	$2\phi_{6,3,1} - \phi_{5,3,1} - \phi_{7,3,1} + 2\phi_{10,4,2} - \phi_{9,4,2} - \phi_{8,4,2}$
13	$2\phi_{6,3,1} - \phi_{5,3,1} - \phi_{7,3,1} - 2\phi_{10,4,2} + \phi_{9,4,2} + \phi_{8,4,2}$
14	$\phi_{5,3,1} - \phi_{7,3,1} + \phi_{9,4,2} - \phi_{8,4,2}$
15	$\phi_{5,3,1} - \phi_{7,3,1} - \phi_{9,4,2} + \phi_{8,4,2}$
16	$2\phi_{5,3,7} - \phi_{6,3,5} - \phi_{6,3,7} + 2\phi_{8,4,9} - \phi_{10,4,9} - \phi_{10,4,8}$
17	$2\phi_{5,3,7} - \phi_{6,3,5} - \phi_{6,3,7} - 2\phi_{8,4,9} + \phi_{10,4,9} + \phi_{10,4,8}$
18	$\phi_{6,3,5} - \phi_{6,3,7} + \phi_{10,4,9} - \phi_{10,4,8}$
19	$\phi_{6,3,5} - \phi_{6,3,7} - \phi_{10,4,9} + \phi_{10,4,8}$
20	$\tau_{5,3,4,9} + \tau_{6,3,4,10} + \tau_{7,3,4,8}$
21	$2\alpha^x_{6,3,1,2} - \alpha^x_{5,3,1,2} - \alpha^x_{7,3,1,2} + 2\alpha^x_{10,4,2,1} - \alpha^x_{9,4,2,1} - \alpha^x_{8,4,2,1}$
22	$2\alpha^x_{6,3,1,2} - \alpha^x_{5,3,1,2} - \alpha^x_{7,3,1,2} - 2\alpha^x_{10,4,2,1} + \alpha^x_{9,4,2,1} + \alpha^x_{8,4,2,1}$
23	$\alpha^x_{5,3,1,2} - \alpha^x_{7,3,1,2} + \alpha^x_{9,4,2,1} - \alpha^x_{8,4,2,1}$
24	$\alpha^x_{5,3,1,2} - \alpha^x_{7,3,1,2} - \alpha^x_{9,4,2,1} + \alpha^x_{8,4,2,1}$

S4.82 bicyclobutane

Table S325: CCSD(T)/cc-pVTZ Optimum Cartesian coordinates (bohr)

1	C	−2.14477327	−0.00000001	0.59607608
2	X	−0.00000000	0.00000000	−0.59997220
3	C	2.14477327	0.00000001	0.59607608
4	C	0.00000000	−1.41792102	−0.59997220
5	C	−0.00000000	1.41792102	−0.59997220
6	H	0.00000005	−2.68099401	−2.19312163
7	H	−0.00000005	2.68099401	−2.19312163
8	H	−2.30669887	−0.00000001	2.64760291
9	H	−3.93191124	−0.00000001	−0.40809085
10	H	2.30669887	0.00000001	2.64760291
11	H	3.93191124	0.00000001	−0.40809085

Table S326: Reference [CCSD(T)/cc-pVTZ] CMA-0A and CMA-2A ($\xi = 0.02$) harmonic frequencies for Level B = MP2/cc-pVTZ and CCSD(T)/cc-pVDZ.

	Reference CCSD(T)/ cc-pVTZ	Pure MP2/ cc-pVTZ	CMA-0A MP2/ cc-pVTZ	CMA-2A MP2/ cc-pVTZ	Pure CCSD(T)/ cc-pVDZ	CMA-0A CCSD(T)/ cc-pVDZ	CMA-2A CCSD(T)/ cc-pVDZ
$\omega_1(a_1)$	3272.01	3288.96	3272.00	3272.00	3412.53	3271.98	3271.98
$\omega_2(a_1)$	3182.39	3203.94	3182.35	3182.35	3329.57	3182.30	3182.30
$\omega_3(a_1)$	3076.39	3091.77	3076.44	3076.44	3213.60	3076.47	3076.47
$\omega_4(a_1)$	1533.54	1531.42	1533.52	1533.52	1557.20	1532.31	1533.35
$\omega_5(a_1)$	1290.37	1287.31	1290.31	1290.35	1327.22	1290.73	1290.16
$\omega_6(a_1)$	1109.26	1106.53	1109.31	1109.26	1127.36	1110.40	1109.62
$\omega_7(a_1)$	867.00	870.32	866.95	866.95	893.20	866.17	866.26
$\omega_8(a_1)$	664.94	655.83	665.01	665.07	678.38	666.14	666.19
$\omega_9(a_1)$	413.18	409.76	413.31	413.20	422.45	413.61	413.35
$\omega_{10}(a_2)$	1185.40	1177.60	1185.26	1185.26	1207.30	1184.57	1184.90
$\omega_{11}(a_2)$	1091.12	1082.06	1090.91	1091.12	1104.57	1090.95	1090.95
$\omega_{12}(a_2)$	925.74	927.99	925.71	925.77	943.59	923.30	925.61
$\omega_{13}(a_2)$	865.50	859.13	866.00	865.66	891.80	869.46	866.54
$\omega_{14}(b_1)$	3259.49	3276.76	3259.49	3259.49	3399.55	3259.48	3259.48
$\omega_{15}(b_1)$	1172.20	1163.71	1170.76	1172.21	1196.72	1171.01	1171.35
$\omega_{16}(b_1)$	1145.12	1137.90	1146.40	1145.11	1157.61	1145.96	1145.96
$\omega_{17}(b_1)$	1003.90	997.61	1004.02	1003.90	1014.72	1003.91	1003.91
$\omega_{18}(b_1)$	751.54	749.53	751.66	751.54	778.20	752.12	751.57
$\omega_{19}(b_2)$	3184.18	3206.65	3184.11	3184.11	3330.82	3184.10	3184.10
$\omega_{20}(b_2)$	3082.35	3099.87	3082.41	3082.41	3218.06	3082.40	3082.40
$\omega_{21}(b_2)$	1498.45	1496.01	1498.36	1498.36	1516.48	1497.36	1497.36
$\omega_{22}(b_2)$	1320.75	1312.77	1320.80	1320.85	1360.98	1321.16	1321.84
$\omega_{23}(b_2)$	1106.79	1107.91	1106.86	1106.79	1126.13	1107.79	1106.98
$\omega_{24}(b_2)$	948.54	949.43	948.56	948.56	959.25	948.64	948.64

Table S327: CMA-0A and CMA-2A (ξ = 0.02) harmonic frequencies targeting CCSD(T)/cc-pVTZ for Level B = B3LYP/6-31G(2*df*,*p*).

	Pure B3LYP/ 6-31G(2*df*,*p*)	CMA-0A B3LYP/ 6-31G(2*df*,*p*)	CMA-2A B3LYP/ 6-31G(2*df*,*p*)
$\omega_1(a_1)$	3277.52	3271.96	3271.96
$\omega_2(a_1)$	3193.57	3182.41	3182.41
$\omega_3(a_1)$	3085.19	3076.38	3076.38
$\omega_4(a_1)$	1528.57	1533.53	1533.53
$\omega_5(a_1)$	1291.57	1290.33	1290.33
$\omega_6(a_1)$	1110.83	1109.18	1109.18
$\omega_7(a_1)$	863.54	867.17	867.17
$\omega_8(a_1)$	678.87	664.87	664.87
$\omega_9(a_1)$	422.90	413.49	413.49
$\omega_{10}(a_2)$	1184.24	1185.16	1185.16
$\omega_{11}(a_2)$	1093.26	1091.14	1091.14
$\omega_{12}(a_2)$	926.68	925.59	925.90
$\omega_{13}(a_2)$	848.90	865.96	865.64
$\omega_{14}(b_1)$	3264.67	3259.48	3259.48
$\omega_{15}(b_1)$	1177.22	1171.76	1171.76
$\omega_{16}(b_1)$	1146.74	1145.49	1145.49
$\omega_{17}(b_1)$	1009.79	1003.98	1004.01
$\omega_{18}(b_1)$	745.40	751.58	751.54
$\omega_{19}(b_2)$	3195.79	3184.15	3184.15
$\omega_{20}(b_2)$	3088.95	3082.34	3082.34
$\omega_{21}(b_2)$	1489.90	1498.26	1498.26
$\omega_{22}(b_2)$	1314.09	1320.89	1321.00
$\omega_{23}(b_2)$	1110.52	1106.95	1106.83
$\omega_{24}(b_2)$	951.54	948.58	948.58

Table S328: Symmetrized, unnormalized natural internal coordinates for bicyclobutane.

1	$r_{4,5}$
2	$r_{4,1} + r_{4,3} + r_{5,1} + r_{5,3}$
3	$r_{4,1} + r_{4,3} - r_{5,1} - r_{5,3}$
4	$r_{4,1} - r_{4,3} + r_{5,1} - r_{5,3}$
5	$r_{4,1} - r_{4,3} - r_{5,1} + r_{5,3}$
6	$r_{4,6} + r_{5,7}$
7	$r_{4,6} - r_{5,7}$
8	$r_{1,8} + r_{1,9} + r_{3,10} + r_{3,11}$
9	$r_{1,8} + r_{1,9} - r_{3,10} - r_{3,11}$
10	$r_{1,8} - r_{1,9} + r_{3,10} - r_{3,11}$
11	$r_{1,8} - r_{1,9} - r_{3,10} + r_{3,11}$
12	$\phi_{6,4,1} - \phi_{6,4,3} + \phi_{7,5,1} - \phi_{7,5,3}$
13	$\phi_{6,4,1} - \phi_{6,4,3} - \phi_{7,5,1} + \phi_{7,5,3}$
14	$4\phi_{8,1,9} - \phi_{8,1,4} - \phi_{8,1,5} - \phi_{9,1,4} - \phi_{9,1,5} + 4\phi_{10,3,11} - \phi_{10,3,4} - \phi_{10,3,5} - \phi_{11,3,4} - \phi_{11,3,5}$
15	$4\phi_{8,1,9} - \phi_{8,1,4} - \phi_{8,1,5} - \phi_{9,1,4} - \phi_{9,1,5} - 4\phi_{10,3,11} + \phi_{10,3,4} + \phi_{10,3,5} + \phi_{11,3,4} + \phi_{11,3,5}$
16	$\phi_{8,1,4} + \phi_{8,1,5} - \phi_{9,1,4} - \phi_{9,1,5} + \phi_{10,3,4} + \phi_{10,3,5} - \phi_{11,3,4} - \phi_{11,3,5}$
17	$\phi_{8,1,4} + \phi_{8,1,5} - \phi_{9,1,4} - \phi_{9,1,5} - \phi_{10,3,4} - \phi_{10,3,5} + \phi_{11,3,4} + \phi_{11,3,5}$
18	$\phi_{8,1,4} - \phi_{8,1,5} + \phi_{9,1,4} - \phi_{9,1,5} + \phi_{10,3,4} - \phi_{10,3,5} + \phi_{11,3,4} - \phi_{11,3,5}$
19	$\phi_{8,1,4} - \phi_{8,1,5} + \phi_{9,1,4} - \phi_{9,1,5} - \phi_{10,3,4} + \phi_{10,3,5} - \phi_{11,3,4} + \phi_{11,3,5}$
20	$\phi_{8,1,4} - \phi_{8,1,5} - \phi_{9,1,4} + \phi_{9,1,5} + \phi_{10,3,4} - \phi_{10,3,5} - \phi_{11,3,4} + \phi_{11,3,5}$
21	$\phi_{8,1,4} - \phi_{8,1,5} - \phi_{9,1,4} + \phi_{9,1,5} - \phi_{10,3,4} + \phi_{10,3,5} + \phi_{11,3,4} - \phi_{11,3,5}$
22	$\tau_{3,4,5,1}$
23	$\gamma_{6,4,3,1} + \gamma_{7,5,1,3}$
24	$\gamma_{6,4,3,1} - \gamma_{7,5,1,3}$

S4.83 cyclobutene

Table S329: CCSD(T)/cc-pVTZ Optimum Cartesian coordinates (bohr)

1	X	0.00000000	0.00000000	−1.56928405
2	C	−1.27271501	0.00000000	1.49443431
3	C	1.27271501	0.00000000	1.49443431
4	C	−1.48500902	−0.00000000	−1.37404402
5	C	1.48500902	0.00000000	−1.37404402
6	H	−2.68110230	−0.00000000	2.97904070
7	H	2.68110230	0.00000000	2.97904070
8	H	−2.34440091	1.68113477	−2.20625364
9	H	−2.34440091	−1.68113477	−2.20625364
10	H	2.34440091	−1.68113477	−2.20625364
11	H	2.34440091	1.68113477	−2.20625364

Table S330: Reference [CCSD(T)/cc-pVTZ] CMA-0A and CMA-2A ($\xi = 0.02$) harmonic frequencies for Level B = MP2/cc-pVTZ and CCSD(T)/cc-pVDZ.

	Reference CCSD(T)/ cc-pVTZ	Pure MP2/ cc-pVTZ	CMA-0A MP2/ cc-pVTZ	CMA-2A MP2/ cc-pVTZ	Pure CCSD(T)/ cc-pVDZ	CMA-0A CCSD(T)/ cc-pVDZ	CMA-2A CCSD(T)/ cc-pVDZ
$\omega_1(a_1)$	3213.66	3230.26	3213.65	3213.65	3358.18	3213.65	3213.65
$\omega_2(a_1)$	3058.21	3073.98	3058.22	3058.22	3192.46	3058.18	3058.18
$\omega_3(a_1)$	1604.92	1595.28	1604.87	1604.88	1670.39	1604.33	1604.44
$\omega_4(a_1)$	1493.88	1490.13	1493.91	1493.91	1511.07	1494.07	1494.07
$\omega_5(a_1)$	1219.17	1205.39	1218.92	1219.14	1244.98	1218.92	1218.77
$\omega_6(a_1)$	1134.81	1131.90	1135.05	1134.83	1159.69	1133.75	1135.14
$\omega_7(a_1)$	1002.35	1002.54	1002.36	1002.34	1020.53	1004.29	1002.72
$\omega_8(a_1)$	895.58	896.49	895.65	895.65	922.17	895.96	895.96
$\omega_9(a_2)$	3097.01	3123.92	3097.00	3097.00	3243.53	3096.99	3096.99
$\omega_{10}(a_2)$	1176.53	1173.10	1176.36	1176.37	1186.64	1176.51	1176.51
$\omega_{11}(a_2)$	1029.84	1024.24	1029.74	1029.93	1039.85	1029.87	1029.88
$\omega_{12}(a_2)$	916.36	927.16	916.68	916.48	926.78	916.39	916.39
$\omega_{13}(a_2)$	302.28	295.95	302.36	302.28	296.27	302.34	302.31
$\omega_{14}(b_1)$	3111.42	3136.64	3111.42	3111.42	3257.48	3111.39	3111.39
$\omega_{15}(b_1)$	1099.90	1100.17	1099.88	1099.88	1110.65	1099.89	1099.89
$\omega_{16}(b_1)$	864.96	868.94	864.92	864.92	878.05	864.98	865.02
$\omega_{17}(b_1)$	644.45	643.28	644.54	644.54	653.19	644.59	644.54
$\omega_{18}(b_2)$	3181.94	3198.25	3181.93	3181.93	3326.33	3181.93	3181.93
$\omega_{19}(b_2)$	3052.18	3070.00	3052.18	3052.18	3184.61	3052.17	3052.17
$\omega_{20}(b_2)$	1470.95	1467.98	1470.90	1470.90	1484.14	1470.78	1470.78
$\omega_{21}(b_2)$	1323.48	1315.33	1323.27	1323.50	1350.90	1322.97	1323.46
$\omega_{22}(b_2)$	1232.80	1226.09	1233.04	1232.80	1247.41	1232.97	1232.97
$\omega_{23}(b_2)$	901.87	902.24	901.88	901.88	924.62	902.65	901.93
$\omega_{24}(b_2)$	856.43	843.86	856.50	856.50	866.47	856.49	856.49

Table S331: CMA-0A and CMA-2A (ξ = 0.02) harmonic frequencies targeting CCSD(T)/cc-pVTZ for Level B = B3LYP/6-31G(2df,p).

	Pure B3LYP/ 6-31G(2df,p)	CMA-0A B3LYP/ 6-31G(2df,p)	CMA-2A B3LYP/ 6-31G(2df,p)
$\omega_1(a_1)$	3221.11	3213.66	3213.66
$\omega_2(a_1)$	3069.23	3058.18	3058.18
$\omega_3(a_1)$	1610.89	1604.54	1604.80
$\omega_4(a_1)$	1488.59	1493.85	1493.85
$\omega_5(a_1)$	1223.53	1218.95	1219.18
$\omega_6(a_1)$	1131.63	1135.05	1134.43
$\omega_7(a_1)$	1000.25	1002.98	1002.98
$\omega_8(a_1)$	896.17	895.74	895.74
$\omega_9(a_2)$	3106.40	3097.00	3097.00
$\omega_{10}(a_2)$	1176.27	1176.35	1176.35
$\omega_{11}(a_2)$	1042.29	1029.84	1029.88
$\omega_{12}(a_2)$	935.31	916.50	916.47
$\omega_{13}(a_2)$	319.68	302.61	302.56
$\omega_{14}(b_1)$	3121.46	3111.42	3111.42
$\omega_{15}(b_1)$	1101.91	1099.81	1099.81
$\omega_{16}(b_1)$	873.91	864.99	864.99
$\omega_{17}(b_1)$	655.30	644.59	644.59
$\omega_{18}(b_2)$	3189.01	3181.92	3181.92
$\omega_{19}(b_2)$	3063.32	3052.16	3052.16
$\omega_{20}(b_2)$	1470.78	1470.85	1470.85
$\omega_{21}(b_2)$	1319.90	1323.24	1323.24
$\omega_{22}(b_2)$	1241.22	1233.10	1233.10
$\omega_{23}(b_2)$	898.72	902.03	902.03
$\omega_{24}(b_2)$	858.70	856.47	856.47

Table S332: Symmetrized, unnormalized natural internal coordinates for cyclobutene.

1	$r_{2,3}$
2	$r_{4,5}$
3	$r_{2,4} + r_{3,5}$
4	$r_{2,4} - r_{3,5}$
5	$r_{2,6} + r_{3,7}$
6	$r_{2,6} - r_{3,7}$
7	$r_{4,8} + r_{4,9} + r_{5,11} + r_{5,10}$
8	$r_{4,8} + r_{4,9} - r_{5,11} - r_{5,10}$
9	$r_{4,8} - r_{4,9} + r_{5,11} - r_{5,10}$
10	$r_{4,8} - r_{4,9} - r_{5,11} + r_{5,10}$
11	$\phi_{2,3,5} - \phi_{3,5,4} + \phi_{5,4,2} - \phi_{4,2,3}$
12	$\phi_{6,2,3} - \phi_{6,2,4} + \phi_{7,3,2} - \phi_{7,3,5}$
13	$\phi_{6,2,3} - \phi_{6,2,4} - \phi_{7,3,2} + \phi_{7,3,5}$
14	$4\phi_{8,4,9} - \phi_{8,4,2} - \phi_{8,4,5} - \phi_{9,4,2} - \phi_{9,4,5} + 4\phi_{10,5,11} - \phi_{11,5,3} - \phi_{11,5,4} - \phi_{10,5,3} - \phi_{10,5,4}$
15	$4\phi_{8,4,9} - \phi_{8,4,2} - \phi_{8,4,5} - \phi_{9,4,2} - \phi_{9,4,5} - 4\phi_{10,5,11} + \phi_{11,5,3} + \phi_{11,5,4} + \phi_{10,5,3} + \phi_{10,5,4}$
16	$\phi_{8,4,2} + \phi_{8,4,5} - \phi_{9,4,2} - \phi_{9,4,5} + \phi_{11,5,3} + \phi_{11,5,4} - \phi_{10,5,3} - \phi_{10,5,4}$
17	$\phi_{8,4,2} + \phi_{8,4,5} - \phi_{9,4,2} - \phi_{9,4,5} - \phi_{11,5,3} - \phi_{11,5,4} + \phi_{10,5,3} + \phi_{10,5,4}$
18	$\phi_{8,4,2} - \phi_{8,4,5} + \phi_{9,4,2} - \phi_{9,4,5} + \phi_{11,5,3} - \phi_{11,5,4} + \phi_{10,5,3} - \phi_{10,5,4}$
19	$\phi_{8,4,2} - \phi_{8,4,5} + \phi_{9,4,2} - \phi_{9,4,5} - \phi_{11,5,3} + \phi_{11,5,4} - \phi_{10,5,3} + \phi_{10,5,4}$
20	$\phi_{8,4,2} - \phi_{8,4,5} - \phi_{9,4,2} + \phi_{9,4,5} + \phi_{11,5,3} - \phi_{11,5,4} - \phi_{10,5,3} + \phi_{10,5,4}$
21	$\phi_{8,4,2} - \phi_{8,4,5} - \phi_{9,4,2} + \phi_{9,4,5} - \phi_{11,5,3} + \phi_{11,5,4} + \phi_{10,5,3} - \phi_{10,5,4}$
22	$\tau_{2,3,5,4} - \tau_{3,5,4,2} + \tau_{5,4,2,3} - \tau_{4,2,3,5}$
23	$\tau_{6,2,3,7}$
24	$\gamma_{6,2,3,4} + \gamma_{7,3,5,2}$

S4.84 methylenecyclopropane

Table S333: CCSD(T)/cc-pVTZ Optimum Cartesian coordinates (bohr)

1	C	−0.00000000	−0.00000000	−3.08775292
2	C	−0.00000000	−0.00000000	−0.58010955
3	C	1.45965770	−0.00000000	1.78621232
4	C	−1.45965770	0.00000000	1.78621232
5	H	1.75483322	−0.00000000	−4.14277197
6	H	−1.75483322	0.00000000	−4.14277197
7	H	2.39963805	1.72748285	2.35547647
8	H	2.39963805	−1.72748285	2.35547647
9	H	−2.39963805	−1.72748285	2.35547647
10	H	−2.39963805	1.72748285	2.35547647

Table S334: Reference [CCSD(T)/cc-pVTZ] CMA-0A and CMA-2A ($\xi = 0.02$) harmonic frequencies for Level B = MP2/cc-pVTZ and CCSD(T)/cc-pVDZ.

	Reference CCSD(T)/ cc-pVTZ	Pure MP2/ cc-pVTZ	CMA-0A MP2/ cc-pVTZ	CMA-2A MP2/ cc-pVTZ	Pure CCSD(T)/ cc-pVDZ	CMA-0A CCSD(T)/ cc-pVDZ	CMA-2A CCSD(T)/ cc-pVDZ
$\omega_1(a_1)$	3139.91	3155.36	3139.88	3139.88	3277.45	3139.90	3139.90
$\omega_2(a_1)$	3130.33	3144.85	3130.35	3130.35	3267.83	3130.31	3130.31
$\omega_3(a_1)$	1819.35	1813.46	1819.30	1819.33	1900.93	1819.31	1819.31
$\omega_4(a_1)$	1493.03	1489.03	1493.01	1493.01	1515.51	1492.66	1492.66
$\omega_5(a_1)$	1450.53	1449.24	1450.58	1450.53	1467.42	1450.57	1450.57
$\omega_6(a_1)$	1055.32	1057.26	1055.09	1055.09	1093.16	1055.79	1055.79
$\omega_7(a_1)$	1041.01	1026.72	1041.24	1041.29	1059.23	1040.89	1040.89
$\omega_8(a_1)$	737.67	740.32	737.77	737.69	763.18	738.08	738.08
$\omega_9(a_2)$	3207.30	3229.97	3207.30	3207.30	3357.19	3207.28	3207.28
$\omega_{10}(a_2)$	1171.65	1170.62	1171.64	1171.64	1182.12	1171.65	1171.65
$\omega_{11}(a_2)$	953.99	960.99	953.80	954.01	963.56	954.01	954.01
$\omega_{12}(a_2)$	615.74	618.35	616.06	615.74	619.86	615.77	615.77
$\omega_{13}(b_1)$	3219.40	3241.35	3219.40	3219.40	3369.12	3219.38	3219.38
$\omega_{14}(b_1)$	1097.51	1095.79	1097.51	1097.51	1107.52	1097.54	1097.54
$\omega_{15}(b_1)$	907.70	914.08	907.70	907.70	915.81	907.70	907.70
$\omega_{16}(b_1)$	753.14	752.51	753.12	753.12	766.01	753.18	753.18
$\omega_{17}(b_1)$	277.65	277.48	277.75	277.72	281.00	277.66	277.66
$\omega_{18}(b_2)$	3225.95	3247.72	3225.95	3225.95	3378.09	3225.95	3225.95
$\omega_{19}(b_2)$	3126.92	3142.68	3126.92	3126.92	3263.42	3126.90	3126.90
$\omega_{20}(b_2)$	1455.65	1453.46	1455.61	1455.61	1468.19	1455.54	1455.54
$\omega_{21}(b_2)$	1147.39	1137.20	1147.30	1147.32	1176.57	1146.11	1146.95
$\omega_{22}(b_2)$	1072.85	1060.07	1072.96	1072.96	1088.85	1073.07	1073.37
$\omega_{23}(b_2)$	908.26	905.43	908.30	908.28	938.91	909.83	908.41
$\omega_{24}(b_2)$	349.27	348.13	349.32	349.29	353.45	349.30	349.30

Table S335: CMA-0A and CMA-2A (ξ = 0.02) harmonic frequencies targeting CCSD(T)/cc-pVTZ for Level B = B3LYP/6-31G(2df,p).

	Pure B3LYP/ 6-31G(2df,p)	CMA-0A B3LYP/ 6-31G(2df,p)	CMA-2A B3LYP/ 6-31G(2df,p)
$\omega_1(a_1)$	3148.88	3139.73	3139.73
$\omega_2(a_1)$	3138.31	3130.42	3130.42
$\omega_3(a_1)$	1808.49	1819.25	1819.40
$\omega_4(a_1)$	1479.59	1493.03	1493.03
$\omega_5(a_1)$	1441.81	1450.70	1450.52
$\omega_6(a_1)$	1055.70	1055.36	1055.43
$\omega_7(a_1)$	1045.89	1041.09	1041.02
$\omega_8(a_1)$	737.75	737.79	737.79
$\omega_9(a_2)$	3216.80	3207.30	3207.30
$\omega_{10}(a_2)$	1169.74	1171.60	1171.60
$\omega_{11}(a_2)$	959.53	954.01	954.01
$\omega_{12}(a_2)$	617.66	615.81	615.81
$\omega_{13}(b_1)$	3230.59	3219.40	3219.40
$\omega_{14}(b_1)$	1096.95	1097.32	1097.32
$\omega_{15}(b_1)$	918.87	907.73	907.73
$\omega_{16}(b_1)$	751.60	753.10	753.18
$\omega_{17}(b_1)$	296.66	278.39	278.17
$\omega_{18}(b_2)$	3238.85	3225.95	3225.95
$\omega_{19}(b_2)$	3136.69	3126.90	3126.90
$\omega_{20}(b_2)$	1447.99	1455.44	1455.44
$\omega_{21}(b_2)$	1141.34	1146.90	1146.90
$\omega_{22}(b_2)$	1075.74	1073.15	1073.15
$\omega_{23}(b_2)$	896.27	908.89	908.89
$\omega_{24}(b_2)$	357.06	349.31	349.31

Table S336: Symmetrized, unnormalized natural internal coordinates for methylenecyclopropane.

1	$r_{1,2}$
2	$r_{3,4} + r_{2,3} + r_{2,4}$
3	$2r_{3,4} - r_{2,3} - r_{2,4}$
4	$r_{2,3} - r_{2,4}$
5	$r_{1,5} + r_{1,6}$
6	$r_{1,5} - r_{1,6}$
7	$r_{3,7} + r_{3,8} + r_{4,10} + r_{4,9}$
8	$r_{3,7} + r_{3,8} - r_{4,10} - r_{4,9}$
9	$r_{3,7} - r_{3,8} + r_{4,10} - r_{4,9}$
10	$r_{3,7} - r_{3,8} - r_{4,10} + r_{4,9}$
11	$\phi_{1,2,3} - \phi_{1,2,4}$
12	$2\phi_{5,1,6} - \phi_{5,1,2} - \phi_{6,1,2}$
13	$\phi_{5,1,2} - \phi_{6,1,2}$
14	$4\phi_{7,3,8} - \phi_{7,3,2} - \phi_{7,3,4} - \phi_{8,3,2} - \phi_{8,3,4} + 4\phi_{9,4,10} - \phi_{10,4,2} - \phi_{10,4,3} - \phi_{9,4,2} - \phi_{9,4,3}$
15	$4\phi_{7,3,8} - \phi_{7,3,2} - \phi_{7,3,4} - \phi_{8,3,2} - \phi_{8,3,4} - 4\phi_{9,4,10} + \phi_{10,4,2} + \phi_{10,4,3} + \phi_{9,4,2} + \phi_{9,4,3}$
16	$\phi_{7,3,2} + \phi_{7,3,4} - \phi_{8,3,2} - \phi_{8,3,4} + \phi_{10,4,2} + \phi_{10,4,3} - \phi_{9,4,2} - \phi_{9,4,3}$
17	$\phi_{7,3,2} + \phi_{7,3,4} - \phi_{8,3,2} - \phi_{8,3,4} - \phi_{10,4,2} - \phi_{10,4,3} + \phi_{9,4,2} + \phi_{9,4,3}$
18	$\phi_{7,3,2} - \phi_{7,3,4} + \phi_{8,3,2} - \phi_{8,3,4} + \phi_{10,4,2} - \phi_{10,4,3} + \phi_{9,4,2} - \phi_{9,4,3}$
19	$\phi_{7,3,2} - \phi_{7,3,4} + \phi_{8,3,2} - \phi_{8,3,4} - \phi_{10,4,2} + \phi_{10,4,3} - \phi_{9,4,2} + \phi_{9,4,3}$
20	$\phi_{7,3,2} - \phi_{7,3,4} - \phi_{8,3,2} + \phi_{8,3,4} + \phi_{10,4,2} - \phi_{10,4,3} - \phi_{9,4,2} + \phi_{9,4,3}$
21	$\phi_{7,3,2} - \phi_{7,3,4} - \phi_{8,3,2} + \phi_{8,3,4} - \phi_{10,4,2} + \phi_{10,4,3} + \phi_{9,4,2} - \phi_{9,4,3}$
22	$\tau_{5,1,2,3} + \tau_{5,1,2,4} + \tau_{6,1,2,3} + \tau_{6,1,2,4}$
23	$\gamma_{1,2,3,4} + \gamma_{3,2,4,1} + \gamma_{4,2,1,3}$
24	$\gamma_{2,1,5,6}$

S4.85 cyclobutane

Table S337: CCSD(T)/cc-pVTZ Optimum Cartesian coordinates (bohr)

1	C	1.44092954	1.44092961	0.27437752
2	C	−1.44092954	−1.44092961	0.27437752
3	C	−1.44092961	1.44092954	−0.27437752
4	C	1.44092961	−1.44092954	−0.27437752
5	H	2.65209587	2.65209594	−0.87006940
6	H	1.80908702	1.80908712	2.27117006
7	H	−2.65209587	−2.65209594	−0.87006940
8	H	−1.80908702	−1.80908712	2.27117006
9	H	−2.65209600	2.65209581	0.87006940
10	H	−1.80908711	1.80908703	−2.27117006
11	H	2.65209600	−2.65209581	0.87006940
12	H	1.80908711	−1.80908703	−2.27117006

Table S338: Reference [CCSD(T)/cc-pVTZ] CMA-0A and CMA-2A (ξ = 0.02) harmonic frequencies for Level B = MP2/cc-pVTZ and CCSD(T)/cc-pVDZ.

	Reference CCSD(T)/ cc-pVTZ	Pure MP2/ cc-pVTZ	CMA-0A MP2/ cc-pVTZ	CMA-2A MP2/ cc-pVTZ	Pure CCSD(T)/ cc-pVDZ	CMA-0A CCSD(T)/ cc-pVDZ	CMA-2A CCSD(T)/ cc-pVDZ
$\omega_1(a_1)$	3104.84	3129.06	3104.36	3104.36	3250.94	3104.55	3104.55
$\omega_2(a_1)$	3063.38	3078.78	3063.86	3063.86	3201.97	3063.65	3063.65
$\omega_3(a_1)$	1526.19	1523.81	1526.18	1526.18	1541.42	1526.08	1526.08
$\omega_4(a_1)$	1180.55	1171.63	1180.56	1180.56	1191.43	1180.51	1180.51
$\omega_5(a_1)$	1025.35	1025.24	1025.37	1025.37	1054.32	1025.63	1025.63
$\omega_6(a_1)$	230.64	224.68	230.65	230.64	233.91	230.66	230.65
$\omega_7(a_2)$	1254.84	1249.31	1254.84	1254.84	1268.33	1254.82	1254.82
$\omega_8(a_2)$	958.26	959.86	958.26	958.26	970.94	958.28	958.28
$\omega_9(b_1)$	1259.25	1255.20	1258.78	1259.22	1282.74	1258.39	1258.39
$\omega_{10}(b_1)$	1167.70	1164.82	1168.16	1167.69	1184.82	1167.86	1168.47
$\omega_{11}(b_1)$	942.94	946.23	943.00	943.00	969.33	943.89	943.13
$\omega_{12}(b_2)$	3131.28	3155.76	3131.27	3131.27	3276.53	3131.24	3131.24
$\omega_{13}(b_2)$	3058.08	3075.59	3058.09	3058.09	3191.32	3058.09	3058.09
$\omega_{14}(b_2)$	1493.48	1492.08	1493.45	1493.45	1502.21	1493.49	1493.49
$\omega_{15}(b_2)$	909.40	903.57	909.34	909.34	921.39	909.44	909.44
$\omega_{16}(b_2)$	620.26	614.83	620.43	620.43	630.43	620.30	620.30
$\omega_{17a}(e)$	3115.63	3141.49	3115.58	3115.58	3261.81	3115.60	3115.60
$\omega_{17b}(e)$	3115.63	3141.39	3115.57	3115.57	3261.70	3115.60	3115.60
$\omega_{18a}(e)$	3058.79	3075.94	3058.85	3058.85	3193.59	3058.80	3058.80
$\omega_{18b}(e)$	3058.79	3075.88	3058.84	3058.84	3193.54	3058.79	3058.79
$\omega_{19a}(e)$	1486.52	1484.22	1486.49	1486.49	1496.71	1486.33	1486.33
$\omega_{19b}(e)$	1486.52	1484.21	1486.49	1486.49	1496.71	1486.33	1486.33
$\omega_{20a}(e)$	1288.37	1278.26	1287.95	1288.38	1313.42	1288.01	1288.01
$\omega_{20b}(e)$	1288.37	1278.25	1287.94	1288.38	1313.40	1288.00	1288.00
$\omega_{21a}(e)$	1253.51	1252.73	1253.97	1253.52	1265.91	1253.74	1253.74
$\omega_{21b}(e)$	1253.51	1252.71	1253.96	1253.52	1265.90	1253.73	1253.73
$\omega_{22a}(e)$	916.90	918.90	916.92	916.92	939.81	917.25	917.25
$\omega_{22b}(e)$	916.90	918.87	916.92	916.92	939.78	917.24	917.24
$\omega_{23a}(e)$	755.06	753.20	755.09	755.09	766.47	755.39	755.39
$\omega_{23b}(e)$	755.06	753.19	755.09	755.09	766.46	755.39	755.39

Table S339: CMA-0A and CMA-2A (ξ = 0.02) harmonic frequencies targeting CCSD(T)/cc-pVTZ for Level B = B3LYP/6-31G(2df,p).

	Pure B3LYP/ 6-31G(2df,p)	CMA-0A B3LYP/ 6-31G(2df,p)	CMA-2A B3LYP/ 6-31G(2df,p)
$\omega_1(a_1)$	3115.22	3104.82	3104.82
$\omega_2(a_1)$	3075.41	3063.35	3063.35
$\omega_3(a_1)$	1523.79	1526.16	1526.16
$\omega_4(a_1)$	1188.87	1180.60	1180.60
$\omega_5(a_1)$	1023.73	1025.45	1025.45
$\omega_6(a_1)$	238.68	230.68	230.68
$\omega_7(a_2)$	1265.76	1254.80	1254.80
$\omega_8(a_2)$	959.17	958.31	958.31
$\omega_9(b_1)$	1263.85	1259.19	1259.24
$\omega_{10}(b_1)$	1165.75	1167.51	1167.51
$\omega_{11}(b_1)$	937.85	943.24	943.18
$\omega_{12}(b_2)$	3147.03	3131.14	3131.14
$\omega_{13}(b_2)$	3073.95	3058.21	3058.21
$\omega_{14}(b_2)$	1500.61	1493.48	1493.48
$\omega_{15}(b_2)$	911.47	909.31	909.40
$\omega_{16}(b_2)$	632.00	620.43	620.30
$\omega_{17\text{a}}(e)$	3128.80	3115.60	3115.60
$\omega_{17\text{b}}(e)$	3124.83	3115.58	3115.58
$\omega_{18\text{a}}(e)$	3071.46	3058.82	3058.82
$\omega_{18\text{b}}(e)$	3069.19	3058.78	3058.78
$\omega_{19\text{a}}(e)$	1488.93	1486.47	1486.47
$\omega_{19\text{b}}(e)$	1484.03	1486.45	1486.45
$\omega_{20\text{a}}(e)$	1298.33	1288.37	1288.37
$\omega_{20\text{b}}(e)$	1296.81	1288.25	1288.25
$\omega_{21\text{a}}(e)$	1256.28	1253.67	1253.67
$\omega_{21\text{b}}(e)$	1253.39	1253.53	1253.53
$\omega_{22\text{a}}(e)$	920.34	916.92	916.92
$\omega_{22\text{b}}(e)$	918.64	916.90	916.90
$\omega_{23\text{a}}(e)$	763.85	755.30	755.30
$\omega_{23\text{b}}(e)$	762.27	755.17	755.17

Table S340: Symmetrized, unnormalized natural internal coordinates for cyclobutane.

1	$r_{1,3} + r_{3,2} + r_{2,4} + r_{4,1}$
2	$r_{1,3} + r_{3,2} - r_{2,4} - r_{4,1}$
3	$r_{1,3} - r_{3,2} + r_{2,4} - r_{4,1}$
4	$r_{1,3} - r_{3,2} - r_{2,4} + r_{4,1}$
5	$r_{1,5} + r_{1,6} + r_{3,9} + r_{3,10} + r_{2,7} + r_{2,8} + r_{4,11} + r_{4,12}$
6	$r_{1,5} + r_{1,6} + r_{3,9} + r_{3,10} - r_{2,7} - r_{2,8} - r_{4,11} - r_{4,12}$
7	$r_{1,5} + r_{1,6} - r_{3,9} - r_{3,10} + r_{2,7} + r_{2,8} - r_{4,11} - r_{4,12}$
8	$r_{1,5} + r_{1,6} - r_{3,9} - r_{3,10} - r_{2,7} - r_{2,8} + r_{4,11} + r_{4,12}$
9	$r_{1,5} - r_{1,6} + r_{3,9} - r_{3,10} + r_{2,7} - r_{2,8} + r_{4,11} - r_{4,12}$
10	$r_{1,5} - r_{1,6} + r_{3,9} - r_{3,10} - r_{2,7} + r_{2,8} - r_{4,11} + r_{4,12}$
11	$r_{1,5} - r_{1,6} - r_{3,9} + r_{3,10} + r_{2,7} - r_{2,8} - r_{4,11} + r_{4,12}$
12	$r_{1,5} - r_{1,6} - r_{3,9} + r_{3,10} - r_{2,7} + r_{2,8} + r_{4,11} - r_{4,12}$
13	$\phi_{4,1,3} - \phi_{1,3,2} + \phi_{3,2,4} - \phi_{2,4,1}$
14	$4\phi_{5,1,6} - \phi_{5,1,3} - \phi_{5,1,4} - \phi_{6,1,3} - \phi_{6,1,4} + 4\phi_{9,3,10} - \phi_{9,3,2} - \phi_{9,3,1} - \phi_{10,3,2} - \phi_{10,3,1}$ $+4\phi_{7,2,8} - \phi_{7,2,4} - \phi_{7,2,3} - \phi_{8,2,4} - \phi_{8,2,3} + 4\phi_{11,4,12} - \phi_{11,4,1} - \phi_{11,4,2} - \phi_{12,4,1} - \phi_{12,4,2}$
15	$4\phi_{5,1,6} - \phi_{5,1,3} - \phi_{5,1,4} - \phi_{6,1,3} - \phi_{6,1,4} + 4\phi_{9,3,10} - \phi_{9,3,2} - \phi_{9,3,1} - \phi_{10,3,2} - \phi_{10,3,1}$ $-4\phi_{7,2,8} + \phi_{7,2,4} + \phi_{7,2,3} + \phi_{8,2,4} + \phi_{8,2,3} - 4\phi_{11,4,12} + \phi_{11,4,1} + \phi_{11,4,2} + \phi_{12,4,1} + \phi_{12,4,2}$
16	$4\phi_{5,1,6} - \phi_{5,1,3} - \phi_{5,1,4} - \phi_{6,1,3} - \phi_{6,1,4} - 4\phi_{9,3,10} + \phi_{9,3,2} + \phi_{9,3,1} + \phi_{10,3,2} + \phi_{10,3,1}$ $+4\phi_{7,2,8} - \phi_{7,2,4} - \phi_{7,2,3} - \phi_{8,2,4} - \phi_{8,2,3} - 4\phi_{11,4,12} + \phi_{11,4,1} + \phi_{11,4,2} + \phi_{12,4,1} + \phi_{12,4,2}$
17	$4\phi_{5,1,6} - \phi_{5,1,3} - \phi_{5,1,4} - \phi_{6,1,3} - \phi_{6,1,4} - 4\phi_{9,3,10} + \phi_{9,3,2} + \phi_{9,3,1} + \phi_{10,3,2} + \phi_{10,3,1}$ $-4\phi_{7,2,8} + \phi_{7,2,4} + \phi_{7,2,3} + \phi_{8,2,4} + \phi_{8,2,3} + 4\phi_{11,4,12} - \phi_{11,4,1} - \phi_{11,4,2} - \phi_{12,4,1} - \phi_{12,4,2}$
18	$\phi_{5,1,3} + \phi_{5,1,4} - \phi_{6,1,3} - \phi_{6,1,4} + \phi_{9,3,2} + \phi_{9,3,1} - \phi_{10,3,2} - \phi_{10,3,1} + \phi_{7,2,4} + \phi_{7,2,3}$ $-\phi_{8,2,4} - \phi_{8,2,3} + \phi_{11,4,1} + \phi_{11,4,2} - \phi_{12,4,1} - \phi_{12,4,2}$
19	$\phi_{5,1,3} + \phi_{5,1,4} - \phi_{6,1,3} - \phi_{6,1,4} + \phi_{9,3,2} + \phi_{9,3,1} - \phi_{10,3,2} - \phi_{10,3,1} - \phi_{7,2,4} - \phi_{7,2,3}$ $+\phi_{8,2,4} + \phi_{8,2,3} - \phi_{11,4,1} - \phi_{11,4,2} + \phi_{12,4,1} + \phi_{12,4,2}$
20	$\phi_{5,1,3} + \phi_{5,1,4} - \phi_{6,1,3} - \phi_{6,1,4} - \phi_{9,3,2} - \phi_{9,3,1} + \phi_{10,3,2} + \phi_{10,3,1} + \phi_{7,2,4} + \phi_{7,2,3}$ $-\phi_{8,2,4} - \phi_{8,2,3} - \phi_{11,4,1} - \phi_{11,4,2} + \phi_{12,4,1} + \phi_{12,4,2}$
21	$\phi_{5,1,3} + \phi_{5,1,4} - \phi_{6,1,3} - \phi_{6,1,4} - \phi_{9,3,2} - \phi_{9,3,1} + \phi_{10,3,2} + \phi_{10,3,1} - \phi_{7,2,4} - \phi_{7,2,3}$ $+\phi_{8,2,4} + \phi_{8,2,3} + \phi_{11,4,1} + \phi_{11,4,2} - \phi_{12,4,1} - \phi_{12,4,2}$
22	$\phi_{5,1,3} - \phi_{5,1,4} + \phi_{6,1,3} - \phi_{6,1,4} + \phi_{9,3,2} - \phi_{9,3,1} + \phi_{10,3,2} - \phi_{10,3,1} + \phi_{7,2,4} - \phi_{7,2,3}$ $+\phi_{8,2,4} - \phi_{8,2,3} + \phi_{11,4,1} - \phi_{11,4,2} + \phi_{12,4,1} - \phi_{12,4,2}$
23	$\phi_{5,1,3} - \phi_{5,1,4} + \phi_{6,1,3} - \phi_{6,1,4} + \phi_{9,3,2} - \phi_{9,3,1} + \phi_{10,3,2} - \phi_{10,3,1} - \phi_{7,2,4} + \phi_{7,2,3}$ $-\phi_{8,2,4} + \phi_{8,2,3} - \phi_{11,4,1} + \phi_{11,4,2} - \phi_{12,4,1} + \phi_{12,4,2}$
24	$\phi_{5,1,3} - \phi_{5,1,4} + \phi_{6,1,3} - \phi_{6,1,4} - \phi_{9,3,2} + \phi_{9,3,1} - \phi_{10,3,2} + \phi_{10,3,1} + \phi_{7,2,4} - \phi_{7,2,3}$ $+\phi_{8,2,4} - \phi_{8,2,3} - \phi_{11,4,1} + \phi_{11,4,2} - \phi_{12,4,1} + \phi_{12,4,2}$
25	$\phi_{5,1,3} - \phi_{5,1,4} + \phi_{6,1,3} - \phi_{6,1,4} - \phi_{9,3,2} + \phi_{9,3,1} - \phi_{10,3,2} + \phi_{10,3,1} - \phi_{7,2,4} + \phi_{7,2,3}$ $-\phi_{8,2,4} + \phi_{8,2,3} + \phi_{11,4,1} - \phi_{11,4,2} + \phi_{12,4,1} - \phi_{12,4,2}$
26	$\phi_{5,1,3} - \phi_{5,1,4} - \phi_{6,1,3} + \phi_{6,1,4} + \phi_{9,3,2} - \phi_{9,3,1} - \phi_{10,3,2} + \phi_{10,3,1} + \phi_{7,2,4} - \phi_{7,2,3}$ $-\phi_{8,2,4} + \phi_{8,2,3} + \phi_{11,4,1} - \phi_{11,4,2} - \phi_{12,4,1} + \phi_{12,4,2}$
27	$\phi_{5,1,3} - \phi_{5,1,4} - \phi_{6,1,3} + \phi_{6,1,4} + \phi_{9,3,2} - \phi_{9,3,1} - \phi_{10,3,2} + \phi_{10,3,1} - \phi_{7,2,4} + \phi_{7,2,3}$ $+\phi_{8,2,4} - \phi_{8,2,3} - \phi_{11,4,1} + \phi_{11,4,2} + \phi_{12,4,1} - \phi_{12,4,2}$
28	$\phi_{5,1,3} - \phi_{5,1,4} - \phi_{6,1,3} + \phi_{6,1,4} - \phi_{9,3,2} + \phi_{9,3,1} + \phi_{10,3,2} - \phi_{10,3,1} + \phi_{7,2,4} - \phi_{7,2,3}$ $-\phi_{8,2,4} + \phi_{8,2,3} - \phi_{11,4,1} + \phi_{11,4,2} + \phi_{12,4,1} - \phi_{12,4,2}$
29	$\phi_{5,1,3} - \phi_{5,1,4} - \phi_{6,1,3} + \phi_{6,1,4} - \phi_{9,3,2} + \phi_{9,3,1} + \phi_{10,3,2} - \phi_{10,3,1} - \phi_{7,2,4} + \phi_{7,2,3}$ $+\phi_{8,2,4} - \phi_{8,2,3} + \phi_{11,4,1} - \phi_{11,4,2} - \phi_{12,4,1} + \phi_{12,4,2}$
30	$\tau_{1,3,2,4} - \tau_{3,2,4,1} + \tau_{2,4,1,3} - \tau_{4,1,3,2}$

S4.86 isobutene

Table S341: CCSD(T)/cc-pVTZ Optimum Cartesian coordinates (bohr)

1	C	−0.00000000	0.00000000	2.71646372
2	C	−0.00000000	0.00000000	0.18428612
3	C	0.00000001	2.41084481	−1.32906270
4	C	−0.00000001	−2.41084481	−1.32906270
5	H	0.00000000	1.75056828	3.77931442
6	H	−0.00000000	−1.75056828	3.77931442
7	H	0.00000001	4.07103635	−0.10803955
8	H	1.66120238	2.49911784	−2.55785943
9	H	−1.66120264	2.49911759	−2.55785931
10	H	−0.00000001	−4.07103635	−0.10803955
11	H	−1.66120238	−2.49911784	−2.55785943
12	H	1.66120264	−2.49911759	−2.55785931

Table S342: Reference [CCSD(T)/cc-pVTZ] CMA-0A and CMA-2A ($\xi = 0.02$) harmonic frequencies for Level B = MP2/cc-pVTZ and CCSD(T)/cc-pVDZ.

	Reference CCSD(T)/ cc-pVTZ	Pure MP2/ cc-pVTZ	CMA-0A MP2/ cc-pVTZ	CMA-2A MP2/ cc-pVTZ	Pure CCSD(T)/ cc-pVDZ	CMA-0A CCSD(T)/ cc-pVDZ	CMA-2A CCSD(T)/ cc-pVDZ
$\omega_1(a_1)$	3140.78	3155.98	3140.76	3140.76	3278.74	3140.69	3140.69
$\omega_2(a_1)$	3121.46	3144.54	3121.41	3121.41	3268.92	3121.42	3121.42
$\omega_3(a_1)$	3024.21	3036.24	3024.28	3024.28	3155.01	3024.27	3024.27
$\omega_4(a_1)$	1710.58	1703.28	1710.53	1710.54	1777.47	1709.78	1709.96
$\omega_5(a_1)$	1506.87	1507.55	1506.89	1506.89	1518.85	1506.76	1506.76
$\omega_6(a_1)$	1447.53	1445.18	1447.34	1447.35	1469.78	1448.41	1448.41
$\omega_7(a_1)$	1410.43	1402.74	1410.64	1410.64	1430.24	1410.44	1410.44
$\omega_8(a_1)$	1081.39	1078.02	1081.42	1081.40	1096.60	1081.75	1081.46
$\omega_9(a_1)$	818.69	817.06	818.71	818.71	846.03	818.81	818.81
$\omega_{10}(a_1)$	368.49	367.10	368.49	368.49	376.37	368.52	368.52
$\omega_{11}(a_2)$	3077.66	3103.21	3077.65	3077.65	3226.57	3077.62	3077.62
$\omega_{12}(a_2)$	1478.41	1478.94	1478.40	1478.40	1488.84	1478.45	1478.45
$\omega_{13}(a_2)$	1019.76	1021.15	1019.60	1019.78	1029.30	1019.79	1019.79
$\omega_{14}(a_2)$	702.97	710.00	703.22	702.97	708.11	702.98	702.98
$\omega_{15}(a_2)$	161.02	164.98	161.05	161.05	169.36	161.04	161.04
$\omega_{16}(b_1)$	3079.75	3104.29	3079.75	3079.75	3228.61	3079.72	3079.72
$\omega_{17}(b_1)$	1495.10	1496.74	1495.09	1495.09	1505.84	1495.12	1495.12
$\omega_{18}(b_1)$	1103.26	1099.41	1103.28	1103.28	1112.96	1103.26	1103.28
$\omega_{19}(b_1)$	905.22	910.60	905.23	905.23	918.16	905.26	905.27
$\omega_{20}(b_1)$	424.36	425.38	424.37	424.37	430.99	424.30	424.36
$\omega_{21}(b_1)$	206.05	210.55	206.05	206.05	210.48	206.31	206.07
$\omega_{22}(b_2)$	3226.12	3248.14	3226.11	3226.11	3377.57	3226.11	3226.11
$\omega_{23}(b_2)$	3119.46	3143.28	3119.40	3119.40	3267.28	3119.34	3119.34
$\omega_{24}(b_2)$	3020.52	3033.41	3020.58	3020.58	3151.17	3020.57	3020.57
$\omega_{25}(b_2)$	1491.26	1491.93	1491.25	1491.25	1501.48	1491.30	1491.30
$\omega_{26}(b_2)$	1414.34	1402.46	1414.26	1414.26	1442.63	1413.15	1413.15
$\omega_{27}(b_2)$	1305.99	1297.70	1306.08	1306.08	1334.40	1306.78	1307.02
$\omega_{28}(b_2)$	988.25	985.44	988.26	988.25	1012.97	988.41	988.15
$\omega_{29}(b_2)$	963.57	959.98	963.59	963.59	978.43	964.23	964.17
$\omega_{30}(b_2)$	426.37	424.74	426.38	426.38	435.89	426.43	426.43

Table S343: CMA-0A and CMA-2A (ξ = 0.02) harmonic frequencies targeting CCSD(T)/cc-pVTZ for Level B = B3LYP/6-31G(2df,p).

	Pure B3LYP/ 6-31G(2df,p)	CMA-0A B3LYP/ 6-31G(2df,p)	CMA-2A B3LYP/ 6-31G(2df,p)
$\omega_1(a_1)$	3149.76	3140.72	3140.72
$\omega_2(a_1)$	3135.99	3121.48	3121.48
$\omega_3(a_1)$	3042.35	3024.20	3024.20
$\omega_4(a_1)$	1706.21	1710.31	1710.31
$\omega_5(a_1)$	1503.53	1506.75	1506.75
$\omega_6(a_1)$	1443.51	1447.41	1447.41
$\omega_7(a_1)$	1416.12	1410.89	1410.89
$\omega_8(a_1)$	1087.88	1081.47	1081.47
$\omega_9(a_1)$	816.06	818.97	818.97
$\omega_{10}(a_1)$	376.56	368.54	368.54
$\omega_{11}(a_2)$	3089.16	3077.65	3077.65
$\omega_{12}(a_2)$	1475.31	1478.36	1478.36
$\omega_{13}(a_2)$	1025.32	1019.80	1019.80
$\omega_{14}(a_2)$	703.70	703.00	703.00
$\omega_{15}(a_2)$	172.11	161.16	161.16
$\omega_{16}(b_1)$	3092.20	3079.74	3079.74
$\omega_{17}(b_1)$	1492.42	1495.07	1495.07
$\omega_{18}(b_1)$	1109.78	1103.23	1103.23
$\omega_{19}(b_1)$	920.39	905.23	905.23
$\omega_{20}(b_1)$	441.01	424.49	424.49
$\omega_{21}(b_1)$	209.67	206.25	206.25
$\omega_{22}(b_2)$	3237.64	3226.11	3226.11
$\omega_{23}(b_2)$	3133.77	3119.44	3119.44
$\omega_{24}(b_2)$	3036.96	3020.49	3020.49
$\omega_{25}(b_2)$	1487.87	1490.98	1490.98
$\omega_{26}(b_2)$	1411.36	1412.85	1412.85
$\omega_{27}(b_2)$	1294.78	1307.29	1307.29
$\omega_{28}(b_2)$	986.67	986.66	986.66
$\omega_{29}(b_2)$	965.74	966.21	966.21
$\omega_{30}(b_2)$	432.59	426.41	426.41

Table S344: Symmetrized, unnormalized natural internal coordinates for isobutene.

1	$r_{1,2}$
2	$r_{2,3} + r_{2,4}$
3	$r_{2,3} - r_{2,4}$
4	$r_{1,5} + r_{1,6}$
5	$r_{1,5} - r_{1,6}$
6	$r_{3,7} + r_{3,9} + r_{3,8} + r_{4,10} + r_{4,11} + r_{4,12}$
7	$r_{3,7} + r_{3,9} + r_{3,8} - r_{4,10} - r_{4,11} - r_{4,12}$
8	$2r_{3,7} - r_{3,9} - r_{3,8} + 2r_{4,10} - r_{4,11} - r_{4,12}$
9	$2r_{3,7} - r_{3,9} - r_{3,8} - 2r_{4,10} + r_{4,11} + r_{4,12}$
10	$r_{3,9} - r_{3,8} + r_{4,11} - r_{4,12}$
11	$r_{3,9} - r_{3,8} - r_{4,11} + r_{4,12}$
12	$2\phi_{3,2,4} - \phi_{3,2,1} - \phi_{4,2,1}$
13	$\phi_{3,2,1} - \phi_{4,2,1}$
14	$2\phi_{5,1,6} - \phi_{5,1,2} - \phi_{6,1,2}$
15	$\phi_{5,1,2} - \phi_{6,1,2}$
16	$\phi_{7,3,2} + \phi_{9,3,2} + \phi_{8,3,2} - \phi_{8,3,9} - \phi_{7,3,9} - \phi_{7,3,8} + \phi_{10,4,2} + \phi_{11,4,2} + \phi_{12,4,2} - \phi_{11,4,12}$ $-\phi_{10,4,11} - \phi_{10,4,12}$
17	$\phi_{7,3,2} + \phi_{9,3,2} + \phi_{8,3,2} - \phi_{8,3,9} - \phi_{7,3,9} - \phi_{7,3,8} - \phi_{10,4,2} - \phi_{11,4,2} - \phi_{12,4,2} + \phi_{11,4,12}$ $+\phi_{10,4,11} + \phi_{10,4,12}$
18	$2\phi_{7,3,2} - \phi_{9,3,2} - \phi_{8,3,2} + 2\phi_{10,4,2} - \phi_{11,4,2} - \phi_{12,4,2}$
19	$2\phi_{7,3,2} - \phi_{9,3,2} - \phi_{8,3,2} - 2\phi_{10,4,2} + \phi_{11,4,2} + \phi_{12,4,2}$
20	$\phi_{9,3,2} - \phi_{8,3,2} + \phi_{11,4,2} - \phi_{12,4,2}$
21	$\phi_{9,3,2} - \phi_{8,3,2} - \phi_{11,4,2} + \phi_{12,4,2}$
22	$2\phi_{8,3,9} - \phi_{7,3,9} - \phi_{7,3,8} + 2\phi_{11,4,12} - \phi_{10,4,11} - \phi_{10,4,12}$
23	$2\phi_{8,3,9} - \phi_{7,3,9} - \phi_{7,3,8} - 2\phi_{11,4,12} + \phi_{10,4,11} + \phi_{10,4,12}$
24	$\phi_{7,3,9} - \phi_{7,3,8} + \phi_{10,4,11} - \phi_{10,4,12}$
25	$\phi_{7,3,9} - \phi_{7,3,8} - \phi_{10,4,11} + \phi_{10,4,12}$
26	$\tau_{6,1,2,4} + \tau_{5,1,2,3}$
27	$\tau_{7,3,2,1} + \tau_{9,3,2,1} + \tau_{8,3,2,1} + \tau_{7,3,2,4} + \tau_{9,3,2,4} + \tau_{8,3,2,4} + \tau_{10,4,2,1} + \tau_{11,4,2,1} + \tau_{12,4,2,1} + \tau_{10,4,2,3}$ $+\tau_{11,4,2,3} + \tau_{12,4,2,3}$
28	$\tau_{7,3,2,1} + \tau_{9,3,2,1} + \tau_{8,3,2,1} + \tau_{7,3,2,4} + \tau_{9,3,2,4} + \tau_{8,3,2,4} - \tau_{10,4,2,1} - \tau_{11,4,2,1} - \tau_{12,4,2,1} - \tau_{10,4,2,3}$ $-\tau_{11,4,2,3} - \tau_{12,4,2,3}$
29	$\gamma_{1,2,3,4}$
30	$\gamma_{2,1,5,6}$

S4.87 pyrrole

Table S345: CCSD(T)/cc-pVTZ Optimum Cartesian coordinates (bohr)

1	N	0.00000000	0.00000000	−2.12001408
2	C	−0.00000000	2.12543160	−0.62715050
3	C	−0.00000000	−2.12543160	−0.62715050
4	C	−0.00000000	−1.34928425	1.86128652
5	C	0.00000000	1.34928425	1.86128652
6	H	0.00000000	3.98802740	−1.44697684
7	H	−0.00000000	−3.98802740	−1.44697684
8	H	0.00000000	−2.57429947	3.48805942
9	H	0.00000000	2.57429947	3.48805942
10	H	0.00000000	0.00000000	−4.01523914

Table S346: Reference [CCSD(T)/cc-pVTZ] CMA-0A and CMA-2A (ξ = 0.02) harmonic frequencies for Level B = MP2/cc-pVTZ and CCSD(T)/cc-pVDZ.

	Reference CCSD(T)/ cc-pVTZ	Pure MP2/ cc-pVTZ	CMA-0A MP2/ cc-pVTZ	CMA-2A MP2/ cc-pVTZ	Pure CCSD(T)/ cc-pVDZ	CMA-0A CCSD(T)/ cc-pVDZ	CMA-2A CCSD(T)/ cc-pVDZ
$\omega_1(a_1)$	3700.44	3711.27	3700.44	3700.44	3803.79	3700.44	3700.44
$\omega_2(a_1)$	3279.10	3294.26	3279.07	3279.07	3419.76	3279.08	3279.08
$\omega_3(a_1)$	3257.75	3273.66	3257.77	3257.77	3396.92	3257.75	3257.75
$\omega_4(a_1)$	1508.88	1506.22	1508.04	1508.86	1559.25	1505.54	1508.68
$\omega_5(a_1)$	1423.16	1415.40	1423.60	1423.13	1481.63	1425.05	1423.21
$\omega_6(a_1)$	1167.23	1164.53	1167.09	1167.27	1203.98	1166.54	1167.26
$\omega_7(a_1)$	1094.84	1100.08	1095.54	1094.83	1116.97	1096.73	1094.71
$\omega_8(a_1)$	1032.96	1031.24	1033.01	1033.01	1053.30	1033.98	1033.26
$\omega_9(a_1)$	889.60	884.03	889.61	889.61	899.23	889.71	889.71
$\omega_{10}(a_2)$	854.80	853.97	854.55	854.80	857.91	854.52	854.80
$\omega_{11}(a_2)$	689.51	679.29	689.71	689.49	697.04	689.52	689.48
$\omega_{12}(a_2)$	615.62	619.21	615.74	615.64	615.29	615.99	615.65
$\omega_{13}(b_1)$	825.18	817.75	824.81	825.17	831.66	825.02	825.17
$\omega_{14}(b_1)$	732.17	729.73	732.24	732.06	738.30	732.18	732.14
$\omega_{15}(b_1)$	634.23	643.44	634.28	634.36	642.47	634.14	634.27
$\omega_{16}(b_1)$	441.88	475.21	442.37	441.88	448.46	442.28	441.88
$\omega_{17}(b_2)$	3272.13	3287.47	3272.09	3272.09	3412.20	3272.12	3272.12
$\omega_{18}(b_2)$	3246.88	3264.03	3246.91	3246.91	3385.64	3246.87	3246.87
$\omega_{19}(b_2)$	1572.76	1562.94	1571.83	1572.75	1626.02	1570.82	1572.73
$\omega_{20}(b_2)$	1465.60	1468.23	1465.76	1465.55	1520.15	1466.34	1465.60
$\omega_{21}(b_2)$	1310.43	1305.09	1310.64	1310.45	1327.81	1310.78	1310.35
$\omega_{22}(b_2)$	1159.51	1160.93	1160.22	1159.51	1178.02	1159.91	1159.39
$\omega_{23}(b_2)$	1065.99	1063.66	1066.05	1065.98	1087.62	1066.93	1066.21
$\omega_{24}(b_2)$	868.69	862.38	868.76	868.76	878.02	868.78	868.78

Table S347: CMA-0A and CMA-2A (ξ = 0.02) harmonic frequencies targeting CCSD(T)/cc-pVTZ for Level B = B3LYP/6-31G(2df,p).

	Pure B3LYP/ 6-31G(2df,p)	CMA-0A B3LYP/ 6-31G(2df,p)	CMA-2A B3LYP/ 6-31G(2df,p)
$\omega_1(a_1)$	3714.09	3700.44	3700.44
$\omega_2(a_1)$	3286.94	3279.03	3279.03
$\omega_3(a_1)$	3265.01	3257.79	3257.79
$\omega_4(a_1)$	1499.87	1508.42	1508.86
$\omega_5(a_1)$	1408.25	1423.47	1423.07
$\omega_6(a_1)$	1169.09	1167.30	1167.35
$\omega_7(a_1)$	1095.44	1094.83	1094.69
$\omega_8(a_1)$	1034.83	1033.07	1033.07
$\omega_9(a_1)$	895.57	889.74	889.74
$\omega_{10}(a_2)$	868.80	854.67	854.80
$\omega_{11}(a_2)$	693.15	689.56	689.50
$\omega_{12}(a_2)$	625.40	615.74	615.63
$\omega_{13}(b_1)$	828.04	824.92	825.17
$\omega_{14}(b_1)$	739.49	732.11	731.94
$\omega_{15}(b_1)$	638.18	634.51	634.49
$\omega_{16}(b_1)$	472.58	442.05	441.89
$\omega_{17}(b_2)$	3284.00	3272.11	3272.11
$\omega_{18}(b_2)$	3254.73	3246.86	3246.86
$\omega_{19}(b_2)$	1574.22	1572.44	1572.76
$\omega_{20}(b_2)$	1451.06	1465.71	1465.58
$\omega_{21}(b_2)$	1313.81	1310.62	1310.43
$\omega_{22}(b_2)$	1159.85	1159.51	1159.44
$\omega_{23}(b_2)$	1070.84	1066.16	1066.16
$\omega_{24}(b_2)$	872.30	868.72	868.72

Table S348: Symmetrized, unnormalized natural internal coordinates for pyrrole.

1	$r_{1,10}$
2	$r_{4,5} + r_{2,5} + r_{3,4} + r_{1,2} + r_{1,3}$
3	$3r_{4,5} + r_{2,5} + r_{3,4} - 3r_{1,2} - 3r_{1,3}$
4	$2r_{2,5} - 2r_{3,4} + r_{1,2} - r_{1,3}$
5	$3r_{4,5} - 3r_{2,5} - 3r_{3,4} + r_{1,2} + r_{1,3}$
6	$r_{2,5} - r_{3,4} - 2r_{1,2} + 2r_{1,3}$
7	$r_{2,6} + r_{3,7}$
8	$r_{2,6} - r_{3,7}$
9	$r_{4,8} + r_{5,9}$
10	$r_{4,8} - r_{5,9}$
11	$\phi_{10,1,2} - \phi_{10,1,3}$
12	$3\phi_{2,1,3} - 3\phi_{1,2,5} - 3\phi_{1,3,4} + \phi_{2,5,4} + \phi_{3,4,5}$
13	$-\phi_{1,2,5} + \phi_{1,3,4} + 2\phi_{2,5,4} - 2\phi_{3,4,5}$
14	$\phi_{6,2,1} - \phi_{6,2,5} + \phi_{7,3,1} - \phi_{7,3,4}$
15	$\phi_{6,2,1} - \phi_{6,2,5} - \phi_{7,3,1} + \phi_{7,3,4}$
16	$\phi_{8,4,3} - \phi_{8,4,5} + \phi_{9,5,2} - \phi_{9,5,4}$
17	$\phi_{8,4,3} - \phi_{8,4,5} - \phi_{9,5,2} + \phi_{9,5,4}$
18	$3\tau_{2,5,4,3} + \tau_{4,3,1,2} + \tau_{3,1,2,5} - 3\tau_{5,4,3,1} - 3\tau_{1,2,5,4}$
19	$2\tau_{4,3,1,2} - 2\tau_{3,1,2,5} - \tau_{5,4,3,1} + \tau_{1,2,5,4}$
20	$\gamma_{10,1,3,2}$
21	$\gamma_{6,2,1,5} + \gamma_{7,3,4,1}$
22	$\gamma_{6,2,1,5} - \gamma_{7,3,4,1}$
23	$\gamma_{8,4,5,3} + \gamma_{9,5,2,4}$
24	$\gamma_{8,4,5,3} - \gamma_{9,5,2,4}$

S4.88 sulfur dioxide

Table S349: CCSD(T)/cc-pVTZ Optimum Cartesian coordinates (bohr)

1	S	0.00000000	0.00000000	0.68492417
2	O	0.00000000	−2.35105565	−0.68454394
3	O	0.00000000	2.35105565	−0.68454394

Table S350: Reference [CCSD(T)/cc-pVTZ] CMA-0A and CMA-2A ($\xi = 0.02$) harmonic frequencies for Level B = MP2/cc-pVTZ and CCSD(T)/cc-pVDZ.

	Reference CCSD(T)/ cc-pVTZ	Pure MP2/ cc-pVTZ	CMA-0A MP2/ cc-pVTZ	CMA-2A MP2/ cc-pVTZ	Pure CCSD(T)/ cc-pVDZ	CMA-0A CCSD(T)/ cc-pVDZ	CMA-2A CCSD(T)/ cc-pVDZ
$\omega_1(a_1)$	1169.10	1190.77	1169.07	1169.07	1178.07	1169.10	1169.10
$\omega_2(a_1)$	519.25	518.10	519.31	519.31	527.44	519.25	519.25
$\omega_2(b_2)$	1388.91	1420.92	1388.91	1388.91	1409.58	1388.91	1388.91

Table S351: CMA-0A and CMA-2A ($\xi = 0.02$) harmonic frequencies targeting CCSD(T)/cc-pVTZ for Level B = B3LYP/6-31G(2*df*,*p*).

	Pure B3LYP/ 6-31G(2*df*,*p*)	CMA-0A B3LYP/ 6-31G(2*df*,*p*)	CMA-2A B3LYP/ 6-31G(2*df*,*p*)
$\omega_1(a_1)$	1186.62	1169.10	1169.10
$\omega_2(a_1)$	520.19	519.25	519.25
$\omega_2(b_2)$	1399.97	1388.91	1388.91

Table S352: Symmetrized, unnormalized natural internal coordinates for sulfur dioxide.

1	$r_{1,2} + r_{1,3}$
2	$r_{1,2} - r_{1,3}$
3	$\phi_{2,1,3}$

S4.89 hydrogen sulfide

Table S353: CCSD(T)/cc-pVTZ Optimum Cartesian coordinates (bohr)

1	S	0.00000000	0.00000000	0.10390451
2	H	0.00000000	−1.82244988	−1.64812453
3	H	0.00000000	1.82244988	−1.64812453

Table S354: Reference [CCSD(T)/cc-pVTZ] CMA-0A and CMA-2A ($\xi = 0.02$) harmonic frequencies for Level B = MP2/cc-pVTZ and CCSD(T)/cc-pVDZ.

	Reference CCSD(T)/ cc-pVTZ	Pure MP2/ cc-pVTZ	CMA-0A MP2/ cc-pVTZ	CMA-2A MP2/ cc-pVTZ	Pure CCSD(T)/ cc-pVDZ	CMA-0A CCSD(T)/ cc-pVDZ	CMA-2A CCSD(T)/ cc-pVDZ
$\omega_1(a_1)$	2722.07	2761.49	2722.07	2722.07	2807.33	2722.07	2722.07
$\omega_2(a_1)$	1209.65	1206.85	1209.65	1209.65	1229.67	1209.66	1209.66
$\omega_2(b_2)$	2736.76	2780.97	2736.76	2736.76	2827.77	2736.76	2736.76

Table S355: CMA-0A and CMA-2A ($\xi = 0.02$) harmonic frequencies targeting CCSD(T)/cc-pVTZ for Level B = B3LYP/6-31G(2df,p).

	Pure B3LYP/ 6-31G(2df,p)	CMA-0A B3LYP/ 6-31G(2df,p)	CMA-2A B3LYP/ 6-31G(2df,p)
$\omega_1(a_1)$	2747.08	2722.07	2722.07
$\omega_2(a_1)$	1220.85	1209.65	1209.65
$\omega_2(b_2)$	2761.81	2736.76	2736.76

Table S356: Symmetrized, unnormalized natural internal coordinates for hydrogen sulfide.

1	$r_{1,2} + r_{1,3}$
2	$r_{1,2} - r_{1,3}$
3	$\phi_{2,1,3}$

S4.90 carbonyl sulfide

Table S357: CCSD(T)/cc-pVTZ Optimum Cartesian coordinates (bohr)

1	S	−0.00000000	−0.00000000	1.97114916
2	C	0.00000000	−0.00000000	−0.99620667
3	X	1.88972652	−0.00000000	−0.99620667
4	O	0.00000000	0.00000000	−3.19271727
5	X	0.00000000	1.88972652	−0.99620667

Table S358: Reference [CCSD(T)/cc-pVTZ] CMA-0A and CMA-2A (ξ = 0.02) harmonic frequencies for Level B = MP2/cc-pVTZ and CCSD(T)/cc-pVDZ.

	Reference CCSD(T)/ cc-pVTZ	Pure MP2/ cc-pVTZ	CMA-0A MP2/ cc-pVTZ	CMA-2A MP2/ cc-pVTZ	Pure CCSD(T)/ cc-pVDZ	CMA-0A CCSD(T)/ cc-pVDZ	CMA-2A CCSD(T)/ cc-pVDZ
$\omega_1(\sigma^+)$	2091.68	2128.22	2091.47	2091.68	2158.00	2091.65	2091.68
$\omega_{2a}(\pi)$	523.09	524.86	523.09	523.09	522.79	523.09	523.09
$\omega_{2b}(\pi)$	523.09	524.84	523.09	523.09	522.78	523.09	523.09
$\omega_3(\sigma^+)$	869.09	884.60	869.59	869.09	899.50	869.16	869.09

Table S359: CMA-0A and CMA-2A (ξ = 0.02) harmonic frequencies targeting CCSD(T)/cc-pVTZ for Level B = B3LYP/6-31G(2*df*,*p*).

	Pure B3LYP/ 6-31G(2*df*,*p*)	CMA-0A B3LYP/ 6-31G(2*df*,*p*)	CMA-2A B3LYP/ 6-31G(2*df*,*p*)
$\omega_1(\sigma^+)$	2112.74	2091.65	2091.68
$\omega_{2a}(\pi)$	531.88	523.09	523.09
$\omega_{2b}(\pi)$	531.87	523.09	523.09
$\omega_3(\sigma^+)$	867.79	869.15	869.09

Table S360: Symmetrized, unnormalized natural internal coordinates for carbonyl sulfide.

1	$r_{1,2}$
2	$r_{2,4}$
3	$\theta_{1,2,4,3}$
4	$\theta_{1,2,4,5}$

S4.91 thiirane

Table S361: CCSD(T)/cc-pVTZ Optimum Cartesian coordinates (bohr)

1	S	−0.00000000	0.00000000	−1.50417512
2	C	−1.40520404	0.00000000	1.64054308
3	C	1.40520404	0.00000000	1.64054308
4	H	−2.36308175	1.72968642	2.16271655
5	H	−2.36308175	−1.72968642	2.16271655
6	H	2.36308175	−1.72968642	2.16271655
7	H	2.36308175	1.72968642	2.16271655

Table S362: Reference [CCSD(T)/cc-pVTZ] CMA-0A and CMA-2A ($\xi = 0.02$) harmonic frequencies for Level B = MP2/cc-pVTZ and CCSD(T)/cc-pVDZ.

	Reference CCSD(T)/ cc-pVTZ	Pure MP2/ cc-pVTZ	CMA-0A MP2/ cc-pVTZ	CMA-2A MP2/ cc-pVTZ	Pure CCSD(T)/ cc-pVDZ	CMA-0A CCSD(T)/ cc-pVDZ	CMA-2A CCSD(T)/ cc-pVDZ
$\omega_1(a_1)$	3142.69	3157.07	3142.68	3142.68	3282.83	3142.67	3142.67
$\omega_2(a_1)$	1502.57	1499.93	1502.52	1502.52	1524.60	1502.18	1502.18
$\omega_3(a_1)$	1139.93	1140.04	1139.91	1139.98	1176.66	1140.25	1140.47
$\omega_4(a_1)$	1048.53	1046.36	1048.62	1048.54	1067.61	1048.56	1048.52
$\omega_5(a_1)$	640.95	650.08	640.99	640.98	664.08	641.34	641.02
$\omega_6(a_2)$	3224.34	3246.36	3224.34	3224.34	3376.58	3224.33	3224.33
$\omega_7(a_2)$	1198.60	1196.20	1198.59	1198.59	1209.64	1198.60	1198.60
$\omega_8(a_1)$	904.36	910.85	904.39	904.39	915.07	904.39	904.39
$\omega_9(b_1)$	3238.02	3259.13	3238.01	3238.01	3389.78	3238.00	3238.00
$\omega_{10}(b_1)$	962.71	963.05	962.70	962.70	978.23	962.69	962.69
$\omega_{11}(b_1)$	833.27	834.59	833.28	833.28	848.89	833.34	833.34
$\omega_{12}(b_2)$	3139.04	3154.94	3139.04	3139.04	3277.34	3139.04	3139.04
$\omega_{13}(b_2)$	1477.31	1477.74	1477.31	1477.31	1490.92	1477.31	1477.31
$\omega_{14}(b_2)$	1073.84	1066.17	1073.84	1073.84	1089.95	1073.82	1073.82
$\omega_{15}(b_2)$	679.82	692.20	679.83	679.83	689.48	679.85	679.85

Table S363: CMA-0A and CMA-2A ($\xi = 0.02$) harmonic frequencies targeting CCSD(T)/cc-pVTZ for Level B = B3LYP/6-31G(2*df*,*p*).

	Pure B3LYP/ 6-31G(2*df*,*p*)	CMA-0A B3LYP/ 6-31G(2*df*,*p*)	CMA-2A B3LYP/ 6-31G(2*df*,*p*)
$\omega_1(a_1)$	3155.52	3142.65	3142.65
$\omega_2(a_1)$	1491.27	1502.53	1502.53
$\omega_3(a_1)$	1139.85	1139.90	1140.01
$\omega_4(a_1)$	1050.63	1048.72	1048.61
$\omega_5(a_1)$	639.23	640.98	640.96
$\omega_6(a_2)$	3241.20	3224.34	3224.34
$\omega_7(a_2)$	1196.59	1198.58	1198.58
$\omega_8(a_1)$	899.85	904.41	904.41
$\omega_9(b_1)$	3256.76	3238.01	3238.01
$\omega_{10}(b_1)$	957.83	962.70	962.72
$\omega_{11}(b_1)$	835.39	833.30	833.27
$\omega_{12}(b_2)$	3154.02	3139.03	3139.03
$\omega_{13}(b_2)$	1471.96	1477.30	1477.30
$\omega_{14}(b_2)$	1081.70	1073.87	1073.87
$\omega_{15}(b_2)$	678.64	679.87	679.87

Table S364: Symmetrized, unnormalized natural internal coordinates for thiirane.

1	$r_{1,2} + r_{1,3} + r_{2,3}$
2	$r_{1,2} - r_{1,3}$
3	$-r_{1,2} - r_{1,3} + 2r_{2,3}$
4	$r_{2,4} + r_{2,5} + r_{3,6} + r_{3,7}$
5	$r_{2,4} + r_{2,5} - r_{3,6} - r_{3,7}$
6	$r_{2,4} - r_{2,5} + r_{3,6} - r_{3,7}$
7	$r_{2,4} - r_{2,5} - r_{3,6} + r_{3,7}$
8	$4\phi_{4,2,5} + 4\phi_{6,3,7} - \phi_{1,2,4} - \phi_{3,2,4} - \phi_{1,2,5} - \phi_{3,2,5} - \phi_{1,3,6} - \phi_{2,3,6} - \phi_{1,3,7} - \phi_{2,3,7}$
9	$4\phi_{4,2,5} - 4\phi_{6,3,7} - \phi_{1,2,4} - \phi_{3,2,4} - \phi_{1,2,5} - \phi_{3,2,5} + \phi_{1,3,6} + \phi_{2,3,6} + \phi_{1,3,7} + \phi_{2,3,7}$
10	$\phi_{1,2,4} + \phi_{3,2,4} - \phi_{1,2,5} - \phi_{3,2,5} + \phi_{1,3,6} + \phi_{2,3,6} - \phi_{1,3,7} - \phi_{2,3,7}$
11	$\phi_{1,2,4} + \phi_{3,2,4} - \phi_{1,2,5} - \phi_{3,2,5} - \phi_{1,3,6} - \phi_{2,3,6} + \phi_{1,3,7} + \phi_{2,3,7}$
12	$\phi_{1,2,4} - \phi_{3,2,4} + \phi_{1,2,5} - \phi_{3,2,5} + \phi_{1,3,6} - \phi_{2,3,6} + \phi_{1,3,7} - \phi_{2,3,7}$
13	$\phi_{1,2,4} - \phi_{3,2,4} + \phi_{1,2,5} - \phi_{3,2,5} - \phi_{1,3,6} + \phi_{2,3,6} - \phi_{1,3,7} + \phi_{2,3,7}$
14	$\phi_{1,2,4} - \phi_{3,2,4} - \phi_{1,2,5} + \phi_{3,2,5} + \phi_{1,3,6} - \phi_{2,3,6} - \phi_{1,3,7} + \phi_{2,3,7}$
15	$\phi_{1,2,4} - \phi_{3,2,4} - \phi_{1,2,5} + \phi_{3,2,5} - \phi_{1,3,6} + \phi_{2,3,6} + \phi_{1,3,7} - \phi_{2,3,7}$

S4.92 dimethyl sulfide

Table S365: CCSD(T)/cc-pVTZ Optimum Cartesian coordinates (bohr)

1	S	−0.00000000	−0.00000000	1.12636820
2	C	−0.00000000	−2.58599764	−1.11200562
3	C	−0.00000000	2.58599764	−1.11200562
4	H	0.00000000	−4.33900174	−0.03161213
5	H	1.68595712	−2.53090490	−2.29714225
6	H	−1.68595712	−2.53090490	−2.29714225
7	H	−0.00000000	4.33900174	−0.03161213
8	H	−1.68595712	2.53090490	−2.29714225
9	H	1.68595712	2.53090490	−2.29714225

Table S366: Reference [CCSD(T)/cc-pVTZ] CMA-0A and CMA-2A ($\xi = 0.02$) harmonic frequencies for Level B = MP2/cc-pVTZ and CCSD(T)/cc-pVDZ.

	Reference CCSD(T)/ cc-pVTZ	Pure MP2/ cc-pVTZ	CMA-0A MP2/ cc-pVTZ	CMA-2A MP2/ cc-pVTZ	Pure CCSD(T)/ cc-pVDZ	CMA-0A CCSD(T)/ cc-pVDZ	CMA-2A CCSD(T)/ cc-pVDZ
$\omega_1(a_1)$	3138.71	3161.78	3138.70	3138.70	3290.71	3138.61	3138.61
$\omega_2(a_1)$	3034.90	3046.90	3034.91	3034.91	3170.28	3034.94	3034.94
$\omega_3(a_1)$	1494.03	1497.17	1494.01	1494.01	1507.49	1494.05	1494.05
$\omega_4(a_1)$	1368.12	1364.18	1368.12	1368.12	1390.00	1368.13	1368.13
$\omega_5(a_1)$	1050.49	1050.65	1050.52	1050.52	1064.72	1050.59	1050.59
$\omega_6(a_1)$	708.45	715.50	708.46	708.46	720.20	708.52	708.52
$\omega_7(a_1)$	262.33	259.78	262.34	262.34	270.35	262.35	262.35
$\omega_8(a_2)$	3120.93	3145.50	3120.93	3120.93	3274.49	3120.89	3120.89
$\omega_9(a_2)$	1469.61	1472.60	1469.59	1469.59	1482.23	1469.65	1469.65
$\omega_{10}(a_2)$	953.94	957.67	953.96	953.96	969.58	954.00	954.00
$\omega_{11}(a_2)$	174.71	178.33	174.71	174.71	181.11	174.72	174.72
$\omega_{12}(b_1)$	3113.45	3136.86	3113.45	3113.45	3267.60	3113.41	3113.41
$\omega_{13}(b_1)$	1478.62	1481.94	1478.61	1478.61	1491.72	1478.65	1478.65
$\omega_{14}(b_1)$	990.25	993.10	990.27	990.27	1008.66	990.30	990.31
$\omega_{15}(b_1)$	186.14	189.00	186.14	186.14	192.75	186.18	186.15
$\omega_{16}(b_2)$	3139.38	3163.09	3139.36	3139.36	3291.17	3139.26	3139.26
$\omega_{17}(b_2)$	3038.72	3052.14	3038.74	3038.74	3173.26	3038.77	3038.77
$\omega_{18}(b_2)$	1486.48	1489.11	1486.47	1486.47	1500.06	1486.50	1486.50
$\omega_{19}(b_2)$	1343.74	1339.54	1343.74	1343.74	1363.34	1343.76	1343.76
$\omega_{20}(b_2)$	911.61	916.02	911.63	911.63	924.90	911.70	911.70
$\omega_{21}(b_2)$	762.13	768.68	762.15	762.15	775.03	762.19	762.19

Table S367: CMA-0A and CMA-2A (ξ = 0.02) harmonic frequencies targeting CCSD(T)/cc-pVTZ for Level B = B3LYP/6-31G(2*df*,*p*).

	Pure B3LYP/ 6-31G(2*df*,*p*)	CMA-0A B3LYP/ 6-31G(2*df*,*p*)	CMA-2A B3LYP/ 6-31G(2*df*,*p*)
$\omega_1(a_1)$	3157.49	3138.69	3138.69
$\omega_2(a_1)$	3055.19	3034.87	3034.87
$\omega_3(a_1)$	1492.25	1494.00	1494.00
$\omega_4(a_1)$	1374.52	1368.13	1368.17
$\omega_5(a_1)$	1057.82	1050.58	1050.58
$\omega_6(a_1)$	704.98	708.56	708.49
$\omega_7(a_1)$	270.60	262.36	262.36
$\omega_8(a_2)$	3139.15	3120.91	3120.91
$\omega_9(a_2)$	1469.76	1469.59	1469.59
$\omega_{10}(a_2)$	958.68	954.01	954.03
$\omega_{11}(a_2)$	187.11	174.83	174.72
$\omega_{12}(b_1)$	3132.10	3113.43	3113.43
$\omega_{13}(b_1)$	1479.82	1478.61	1478.61
$\omega_{14}(b_1)$	994.89	990.31	990.32
$\omega_{15}(b_1)$	189.01	186.24	186.18
$\omega_{16}(b_2)$	3158.13	3139.35	3139.35
$\omega_{17}(b_2)$	3058.17	3038.69	3038.69
$\omega_{18}(b_2)$	1484.92	1486.42	1486.42
$\omega_{19}(b_2)$	1349.50	1343.75	1343.81
$\omega_{20}(b_2)$	917.81	911.74	911.74
$\omega_{21}(b_2)$	753.15	762.27	762.16

Table S368: Symmetrized, unnormalized natural internal coordinates for dimethyl sulfide.

1	$r_{1,2} + r_{1,3}$
2	$r_{1,2} - r_{1,3}$
3	$r_{2,4} + r_{2,5} + r_{2,6} + r_{3,7} + r_{3,8} + r_{3,9}$
4	$r_{2,4} + r_{2,5} + r_{2,6} - r_{3,7} - r_{3,8} - r_{3,9}$
5	$2r_{2,4} - r_{2,5} - r_{2,6} + 2r_{3,7} - r_{3,8} - r_{3,9}$
6	$2r_{2,4} - r_{2,5} - r_{2,6} - 2r_{3,7} + r_{3,8} + r_{3,9}$
7	$r_{2,5} - r_{2,6} + r_{3,8} - r_{3,9}$
8	$r_{2,5} - r_{2,6} - r_{3,8} + r_{3,9}$
9	$\phi_{2,1,3}$
10	$\phi_{4,2,1} + \phi_{5,2,1} + \phi_{6,2,1} - \phi_{5,2,6} - \phi_{4,2,5} - \phi_{4,2,6} + \phi_{7,3,1} + \phi_{8,3,1} + \phi_{9,3,1} - \phi_{8,3,9}$ $-\phi_{7,3,8} - \phi_{7,3,9}$
11	$\phi_{4,2,1} + \phi_{5,2,1} + \phi_{6,2,1} - \phi_{5,2,6} - \phi_{4,2,5} - \phi_{4,2,6} - \phi_{7,3,1} - \phi_{8,3,1} - \phi_{9,3,1} + \phi_{8,3,9}$ $+\phi_{7,3,8} + \phi_{7,3,9}$
12	$2\phi_{4,2,1} - \phi_{5,2,1} - \phi_{6,2,1} + 2\phi_{7,3,1} - \phi_{8,3,1} - \phi_{9,3,1}$
13	$2\phi_{4,2,1} - \phi_{5,2,1} - \phi_{6,2,1} - 2\phi_{7,3,1} + \phi_{8,3,1} + \phi_{9,3,1}$
14	$\phi_{5,2,1} - \phi_{6,2,1} + \phi_{8,3,1} - \phi_{9,3,1}$
15	$\phi_{5,2,1} - \phi_{6,2,1} - \phi_{8,3,1} + \phi_{9,3,1}$
16	$2\phi_{5,2,6} - \phi_{4,2,5} - \phi_{4,2,6} + 2\phi_{8,3,9} - \phi_{7,3,8} - \phi_{7,3,9}$
17	$2\phi_{5,2,6} - \phi_{4,2,5} - \phi_{4,2,6} - 2\phi_{8,3,9} + \phi_{7,3,8} + \phi_{7,3,9}$
18	$\phi_{4,2,5} - \phi_{4,2,6} + \phi_{7,3,8} - \phi_{7,3,9}$
19	$\phi_{4,2,5} - \phi_{4,2,6} - \phi_{7,3,8} + \phi_{7,3,9}$
20	$\tau_{4,2,1,3} + \tau_{5,2,1,3} + \tau_{6,2,1,3} + \tau_{7,3,1,2} + \tau_{8,3,1,2} + \tau_{9,3,1,2}$
21	$\tau_{4,2,1,3} + \tau_{5,2,1,3} + \tau_{6,2,1,3} - \tau_{7,3,1,2} - \tau_{8,3,1,2} - \tau_{9,3,1,2}$

S4.93 thioethanol

Table S369: CCSD(T)/cc-pVTZ Optimum Cartesian coordinates (bohr)

1	S	−1.94413062	0.32139246	0.00000000
2	C	1.11416252	−1.29182836	0.00000000
3	C	3.20028202	0.69835977	0.00000000
4	H	−3.38597804	−1.75712103	−0.00000000
5	H	1.25064962	−2.48270008	1.67469531
6	H	1.25064962	−2.48270008	−1.67469531
7	H	5.05305419	−0.20846079	0.00000000
8	H	3.06777149	1.90075538	1.67101309
9	H	3.06777149	1.90075538	−1.67101309

Table S370: Reference [CCSD(T)/cc-pVTZ] CMA-0A and CMA-2A (ξ = 0.02) harmonic frequencies for Level B = MP2/cc-pVTZ and CCSD(T)/cc-pVDZ.

	Reference CCSD(T)/ cc-pVTZ	Pure MP2/ cc-pVTZ	CMA-0A MP2/ cc-pVTZ	CMA-2A MP2/ cc-pVTZ	Pure CCSD(T)/ cc-pVDZ	CMA-0A CCSD(T)/ cc-pVDZ	CMA-2A CCSD(T)/ cc-pVDZ
$\omega_1(a^{'})$	3118.15	3142.85	3118.13	3118.13	3269.41	3118.03	3118.03
$\omega_2(a^{'})$	3068.16	3084.39	3068.14	3068.14	3206.96	3068.14	3068.14
$\omega_3(a^{'})$	3040.58	3052.91	3040.63	3040.63	3174.67	3040.65	3040.65
$\omega_4(a^{'})$	2709.33	2751.21	2709.33	2709.33	2796.68	2709.33	2709.33
$\omega_5(a^{'})$	1510.01	1512.18	1509.95	1509.95	1522.66	1510.05	1510.05
$\omega_6(a^{'})$	1496.40	1496.71	1496.41	1496.41	1512.96	1496.26	1496.26
$\omega_7(a^{'})$	1413.61	1405.70	1413.60	1413.60	1433.27	1413.34	1413.34
$\omega_8(a^{'})$	1302.20	1296.35	1302.20	1302.20	1325.81	1302.27	1302.27
$\omega_9(a^{'})$	1116.09	1113.71	1116.10	1116.12	1135.58	1115.88	1116.44
$\omega_{10}(a^{'})$	1001.77	1001.61	1001.79	1001.79	1027.55	1002.49	1001.88
$\omega_{11}(a^{'})$	863.95	860.16	863.96	863.94	882.54	864.16	864.15
$\omega_{12}(a^{'})$	687.08	694.65	687.14	687.14	698.22	687.11	687.12
$\omega_{13}(a^{'})$	301.94	302.79	301.97	301.97	308.37	301.97	301.96
$\omega_{14}(a^{''})$	3129.76	3153.78	3129.76	3129.76	3280.44	3129.68	3129.68
$\omega_{15}(a^{''})$	3107.06	3132.09	3107.06	3107.06	3260.50	3107.11	3107.11
$\omega_{16}(a^{''})$	1500.20	1502.55	1500.19	1500.19	1512.10	1500.23	1500.23
$\omega_{17}(a^{''})$	1271.87	1271.55	1271.82	1271.82	1285.97	1271.86	1271.86
$\omega_{18}(a^{''})$	1046.64	1048.45	1046.71	1046.71	1063.04	1046.67	1046.67
$\omega_{19}(a^{''})$	789.89	790.35	789.90	789.90	803.96	789.94	789.95
$\omega_{20}(a^{''})$	252.94	257.41	252.93	252.94	268.48	252.83	252.95
$\omega_{21}(a^{''})$	177.40	180.15	177.42	177.41	186.61	177.66	177.45

Table S371: CMA-0A and CMA-2A (ξ = 0.02) harmonic frequencies targeting CCSD(T)/cc-pVTZ for Level B = B3LYP/6-31G(2*df*,*p*).

	Pure B3LYP/ 6-31G(2*df*,*p*)	CMA-0A B3LYP/ 6-31G(2*df*,*p*)	CMA-2A B3LYP/ 6-31G(2*df*,*p*)
$\omega_1(a^{'})$	3133.91	3118.14	3118.14
$\omega_2(a^{'})$	3082.36	3068.13	3068.13
$\omega_3(a^{'})$	3060.71	3040.59	3040.59
$\omega_4(a^{'})$	2732.64	2709.32	2709.32
$\omega_5(a^{'})$	1509.70	1509.93	1509.93
$\omega_6(a^{'})$	1494.76	1496.40	1496.40
$\omega_7(a^{'})$	1421.74	1413.46	1413.46
$\omega_8(a^{'})$	1305.25	1302.14	1302.14
$\omega_9(a^{'})$	1117.87	1115.89	1115.89
$\omega_{10}(a^{'})$	999.04	1002.13	1002.13
$\omega_{11}(a^{'})$	866.30	864.23	864.23
$\omega_{12}(a^{'})$	681.02	687.30	687.30
$\omega_{13}(a^{'})$	303.81	302.03	302.03
$\omega_{14}(a^{''})$	3146.90	3129.73	3129.73
$\omega_{15}(a^{''})$	3122.57	3107.07	3107.07
$\omega_{16}(a^{''})$	1499.64	1500.20	1500.20
$\omega_{17}(a^{''})$	1274.42	1271.79	1271.79
$\omega_{18}(a^{''})$	1047.44	1046.72	1046.72
$\omega_{19}(a^{''})$	795.85	789.98	789.98
$\omega_{20}(a^{''})$	252.75	252.94	252.98
$\omega_{21}(a^{''})$	184.36	177.48	177.42

Table S372: Symmetrized, unnormalized natural internal coordinates for thioethanol.

1	$r_{3,2}$
2	$r_{3,1}$
3	$r_{1,4}$
4	$r_{3,7} + r_{3,8} + r_{3,9}$
5	$2r_{3,7} - r_{3,8} - r_{3,9}$
6	$r_{3,8} - r_{3,9}$
7	$r_{2,5} + r_{2,6}$
8	$r_{2,5} - r_{2,6}$
9	$\phi_{3,2,1}$
10	$\phi_{2,1,4}$
11	$\phi_{7,3,2} + \phi_{8,3,2} + \phi_{9,3,2} - \phi_{8,3,9} - \phi_{7,3,8} - \phi_{7,3,9}$
12	$2\phi_{7,3,2} - \phi_{8,3,2} - \phi_{9,3,2}$
13	$\phi_{8,3,2} - \phi_{9,3,2}$
14	$2\phi_{8,3,9} - \phi_{7,3,8} - \phi_{7,3,9}$
15	$\phi_{7,3,8} - \phi_{7,3,9}$
16	$4\phi_{5,2,6} - \phi_{5,2,3} - \phi_{5,2,1} - \phi_{6,2,3} - \phi_{6,2,1}$
17	$\phi_{5,2,3} + \phi_{5,2,1} - \phi_{6,2,3} - \phi_{6,2,1}$
18	$\phi_{5,2,3} - \phi_{5,2,1} + \phi_{6,2,3} - \phi_{6,2,1}$
19	$\phi_{5,2,3} - \phi_{5,2,1} - \phi_{6,2,3} + \phi_{6,2,1}$
20	$\tau_{7,3,2,1} + \tau_{8,3,2,1} + \tau_{9,3,2,1}$
21	$\tau_{3,2,1,4}$

S4.94 dimethyl sulfoxide

Table S373: CCSD(T)/cc-pVTZ Optimum Cartesian coordinates (bohr)

1	S	−0.79551986	−0.28979430	−0.00000000
2	O	0.70249582	−2.66735948	−0.00000000
3	C	0.42748211	1.65658768	2.53093481
4	C	0.42748211	1.65658768	−2.53093481
5	H	−0.13039689	0.76873497	4.30213494
6	H	−0.39926512	3.54058228	2.39620963
7	H	2.48361526	1.72913497	2.39620963
8	H	−0.13039689	0.76873497	−4.30213494
9	H	2.48361526	1.72913497	−2.39620963
10	H	−0.39926512	3.54058228	−2.39620963

Table S374: Reference [CCSD(T)/cc-pVTZ] CMA-0A and CMA-2A (ξ = 0.02) harmonic frequencies for Level B = MP2/cc-pVTZ and CCSD(T)/cc-pVDZ.

	Reference CCSD(T)/ cc-pVTZ	Pure MP2/ cc-pVTZ	CMA-0A MP2/ cc-pVTZ	CMA-2A MP2/ cc-pVTZ	Pure CCSD(T)/ cc-pVDZ	CMA-0A CCSD(T)/ cc-pVDZ	CMA-2A CCSD(T)/ cc-pVDZ
$\omega_1(a^{'})$	3153.52	3175.39	3153.50	3153.50	3302.98	3153.35	3153.35
$\omega_2(a^{'})$	3145.65	3167.15	3145.67	3145.67	3294.70	3145.68	3145.68
$\omega_3(a^{'})$	3041.26	3052.14	3041.27	3041.27	3172.11	3041.31	3041.31
$\omega_4(a^{'})$	1482.86	1486.44	1482.83	1482.83	1496.98	1482.85	1482.85
$\omega_5(a^{'})$	1460.90	1462.67	1460.87	1460.87	1472.87	1460.82	1460.82
$\omega_6(a^{'})$	1337.37	1331.30	1337.36	1337.36	1353.06	1337.37	1337.37
$\omega_7(a^{'})$	1134.06	1175.93	1133.63	1134.10	1153.12	1134.09	1134.09
$\omega_8(a^{'})$	1024.64	1021.74	1024.63	1024.64	1032.52	1024.74	1024.79
$\omega_9(a^{'})$	955.70	958.39	956.23	955.77	966.06	955.85	955.90
$\omega_{10}(a^{'})$	669.27	677.21	669.41	669.29	677.03	669.39	669.39
$\omega_{11}(a^{'})$	368.56	367.11	368.56	368.57	368.45	368.38	368.37
$\omega_{12}(a^{'})$	290.44	290.07	290.31	290.30	295.66	290.61	290.61
$\omega_{13}(a^{'})$	238.88	241.15	239.13	239.08	240.29	239.53	239.17
$\omega_{14}(a^{''})$	3152.29	3174.41	3152.28	3152.28	3301.83	3152.13	3152.13
$\omega_{15}(a^{''})$	3141.10	3163.22	3141.10	3141.10	3290.50	3141.12	3141.12
$\omega_{16}(a^{''})$	3039.48	3051.26	3039.49	3039.49	3170.30	3039.51	3039.51
$\omega_{17}(a^{''})$	1463.94	1466.98	1463.91	1463.91	1478.28	1463.92	1463.92
$\omega_{18}(a^{''})$	1446.99	1447.72	1446.99	1446.99	1458.92	1446.97	1446.97
$\omega_{19}(a^{''})$	1315.06	1309.06	1315.06	1315.06	1328.64	1315.04	1315.04
$\omega_{20}(a^{''})$	924.12	926.54	924.11	924.13	929.22	924.11	924.12
$\omega_{21}(a^{''})$	886.07	886.07	886.10	886.13	890.73	886.38	886.47
$\omega_{22}(a^{''})$	693.75	698.90	693.78	693.76	702.99	693.89	693.89
$\omega_{23}(a^{''})$	314.56	314.07	314.55	314.56	310.09	314.56	314.59
$\omega_{24}(a^{''})$	179.27	180.29	179.45	179.28	174.66	179.83	179.33

Table S375: CMA-0A and CMA-2A (ξ = 0.02) harmonic frequencies targeting CCSD(T)/cc-pVTZ for Level B = B3LYP/6-31G(2df,p).

	Pure B3LYP/ 6-31G(2df,p)	CMA-0A B3LYP/ 6-31G(2df,p)	CMA-2A B3LYP/ 6-31G(2df,p)
$\omega_1(a^{'})$	3169.82	3153.47	3153.47
$\omega_2(a^{'})$	3161.96	3145.66	3145.66
$\omega_3(a^{'})$	3058.89	3041.21	3041.21
$\omega_4(a^{'})$	1482.53	1482.79	1482.79
$\omega_5(a^{'})$	1457.21	1460.83	1460.83
$\omega_6(a^{'})$	1340.69	1337.49	1337.49
$\omega_7(a^{'})$	1139.34	1134.07	1134.07
$\omega_8(a^{'})$	1029.20	1024.75	1024.75
$\omega_9(a^{'})$	959.20	955.83	955.83
$\omega_{10}(a^{'})$	668.59	669.37	669.37
$\omega_{11}(a^{'})$	368.87	368.25	368.25
$\omega_{12}(a^{'})$	297.04	290.56	290.56
$\omega_{13}(a^{'})$	227.74	239.37	239.36
$\omega_{14}(a^{''})$	3168.78	3152.22	3152.22
$\omega_{15}(a^{''})$	3156.76	3141.12	3141.12
$\omega_{16}(a^{''})$	3056.12	3039.44	3039.44
$\omega_{17}(a^{''})$	1462.48	1463.87	1463.87
$\omega_{18}(a^{''})$	1443.27	1446.93	1446.93
$\omega_{19}(a^{''})$	1318.65	1315.17	1315.17
$\omega_{20}(a^{''})$	931.44	924.20	924.22
$\omega_{21}(a^{''})$	889.74	886.22	886.22
$\omega_{22}(a^{''})$	690.70	693.89	693.86
$\omega_{23}(a^{''})$	319.24	314.59	314.59
$\omega_{24}(a^{''})$	176.64	179.38	179.31

Table S376: Symmetrized, unnormalized natural internal coordinates for dimethyl sulfoxide.

1	$r_{1,2}$
2	$r_{1,3} + r_{1,4}$
3	$r_{1,3} - r_{1,4}$
4	$r_{3,5} + r_{3,6} + r_{3,7} + r_{4,8} + r_{4,9} + r_{4,10}$
5	$r_{3,5} + r_{3,6} + r_{3,7} - r_{4,8} - r_{4,9} - r_{4,10}$
6	$2r_{3,5} - r_{3,6} - r_{3,7} + 2r_{4,8} - r_{4,9} - r_{4,10}$
7	$2r_{3,5} - r_{3,6} - r_{3,7} - 2r_{4,8} + r_{4,9} + r_{4,10}$
8	$r_{3,6} - r_{3,7} + r_{4,9} - r_{4,10}$
9	$r_{3,6} - r_{3,7} - r_{4,9} + r_{4,10}$
10	$2\phi_{3,1,4} - \phi_{2,1,3} - \phi_{2,1,4}$
11	$\phi_{2,1,3} - \phi_{2,1,4}$
12	$\phi_{5,3,1} + \phi_{6,3,1} + \phi_{7,3,1} - \phi_{6,3,7} - \phi_{5,3,6} - \phi_{5,3,7} + \phi_{8,4,1} + \phi_{9,4,1} + \phi_{10,4,1} - \phi_{9,4,10}$ $-\phi_{8,4,9} - \phi_{8,4,10}$
13	$\phi_{5,3,1} + \phi_{6,3,1} + \phi_{7,3,1} - \phi_{6,3,7} - \phi_{5,3,6} - \phi_{5,3,7} - \phi_{8,4,1} - \phi_{9,4,1} - \phi_{10,4,1} + \phi_{9,4,10}$ $+\phi_{8,4,9} + \phi_{8,4,10}$
14	$2\phi_{5,3,1} - \phi_{6,3,1} - \phi_{7,3,1} + 2\phi_{8,4,1} - \phi_{9,4,1} - \phi_{10,4,1}$
15	$2\phi_{5,3,1} - \phi_{6,3,1} - \phi_{7,3,1} - 2\phi_{8,4,1} + \phi_{9,4,1} + \phi_{10,4,1}$
16	$\phi_{6,3,1} - \phi_{7,3,1} + \phi_{9,4,1} - \phi_{10,4,1}$
17	$\phi_{6,3,1} - \phi_{7,3,1} - \phi_{9,4,1} + \phi_{10,4,1}$
18	$2\phi_{6,3,7} - \phi_{5,3,6} - \phi_{5,3,7} + 2\phi_{9,4,10} - \phi_{8,4,9} - \phi_{8,4,10}$
19	$2\phi_{6,3,7} - \phi_{5,3,6} - \phi_{5,3,7} - 2\phi_{9,4,10} + \phi_{8,4,9} + \phi_{8,4,10}$
20	$\phi_{5,3,6} - \phi_{5,3,7} + \phi_{8,4,9} - \phi_{8,4,10}$
21	$\phi_{5,3,6} - \phi_{5,3,7} - \phi_{8,4,9} + \phi_{8,4,10}$
22	$\tau_{5,3,1,4} + \tau_{6,3,1,4} + \tau_{7,3,1,4} + \tau_{8,4,1,3} + \tau_{9,4,1,3} + \tau_{10,4,1,3}$
23	$\tau_{5,3,1,4} + \tau_{6,3,1,4} + \tau_{7,3,1,4} - \tau_{8,4,1,3} - \tau_{9,4,1,3} - \tau_{10,4,1,3}$
24	$\gamma_{2,1,3,4}$

S4.95 thiophene

Table S377: CCSD(T)/cc-pVTZ Optimum Cartesian coordinates (bohr)

1	X	0.00000000	0.00000000	−0.28192818
2	S	0.00000000	0.00000000	−2.17165471
3	C	0.00000000	2.33762887	0.09177468
4	C	−0.00000000	−2.33762887	0.09177468
5	C	−0.00000000	−1.35030259	2.48887766
6	C	0.00000000	1.35030259	2.48887766
7	H	0.00000000	4.29941975	−0.45873605
8	H	−0.00000000	−4.29941975	−0.45873605
9	H	−0.00000000	−2.49824704	4.17795384
10	H	0.00000000	2.49824704	4.17795384

Table S378: Reference [CCSD(T)/cc-pVTZ] CMA-0A and CMA-2A (ξ = 0.02) harmonic frequencies for Level B = MP2/cc-pVTZ and CCSD(T)/cc-pVDZ.

	Reference CCSD(T)/ cc-pVTZ	Pure MP2/ cc-pVTZ	CMA-0A MP2/ cc-pVTZ	CMA-2A MP2/ cc-pVTZ	Pure CCSD(T)/ cc-pVDZ	CMA-0A CCSD(T)/ cc-pVDZ	CMA-2A CCSD(T)/ cc-pVDZ
$\omega_1(a_1)$	3259.29	3274.28	3259.29	3259.29	3401.91	3259.28	3259.28
$\omega_2(a_1)$	3227.81	3243.48	3227.80	3227.80	3368.35	3227.81	3227.81
$\omega_3(a_1)$	1442.59	1431.52	1441.50	1442.60	1515.72	1440.39	1442.65
$\omega_4(a_1)$	1396.08	1388.53	1397.06	1396.07	1435.04	1397.20	1395.90
$\omega_5(a_1)$	1098.26	1093.31	1098.26	1098.25	1113.84	1098.50	1098.10
$\omega_6(a_1)$	1051.60	1050.22	1051.59	1051.60	1080.54	1052.61	1051.86
$\omega_7(a_1)$	845.49	854.01	845.62	845.50	867.61	845.77	845.60
$\omega_8(a_1)$	609.94	608.96	610.15	609.96	621.67	610.04	609.96
$\omega_9(a_2)$	902.59	899.84	902.33	902.59	908.03	902.43	902.59
$\omega_{10}(a_2)$	688.05	680.50	688.19	688.05	699.39	688.05	688.06
$\omega_{11}(a_2)$	565.53	572.20	565.77	565.53	564.75	565.79	565.53
$\omega_{12}(b_1)$	875.51	867.08	875.46	875.51	884.63	875.49	875.51
$\omega_{13}(b_1)$	725.76	728.43	725.75	725.76	734.62	725.76	725.76
$\omega_{14}(b_1)$	453.25	459.09	453.36	453.25	453.82	453.28	453.25
$\omega_{15}(b_2)$	3256.05	3271.39	3256.05	3256.05	3398.66	3256.05	3256.05
$\omega_{16}(b_2)$	3213.82	3230.68	3213.82	3213.82	3353.87	3213.82	3213.82
$\omega_{17}(b_2)$	1545.40	1532.18	1545.38	1545.39	1605.59	1544.78	1545.07
$\omega_{18}(b_2)$	1278.90	1270.27	1278.89	1278.89	1299.45	1279.20	1279.20
$\omega_{19}(b_2)$	1100.85	1098.79	1100.85	1100.84	1118.24	1101.29	1100.87
$\omega_{20}(b_2)$	878.28	879.42	878.27	878.27	896.34	878.10	878.33
$\omega_{21}(b_2)$	758.88	757.22	758.93	758.93	775.53	759.23	758.97

Table S379: CMA-0A and CMA-2A (ξ = 0.02) harmonic frequencies targeting CCSD(T)/cc-pVTZ for Level B = B3LYP/6-31G(2*df*,*p*).

	Pure B3LYP/ 6-31G(2*df*,*p*)	CMA-0A B3LYP/ 6-31G(2*df*,*p*)	CMA-2A B3LYP/ 6-31G(2*df*,*p*)
$\omega_1(a_1)$	3270.18	3259.24	3259.24
$\omega_2(a_1)$	3236.44	3227.83	3227.83
$\omega_3(a_1)$	1430.55	1442.22	1442.55
$\omega_4(a_1)$	1390.04	1396.31	1396.06
$\omega_5(a_1)$	1102.89	1098.26	1098.17
$\omega_6(a_1)$	1052.43	1051.76	1051.76
$\omega_7(a_1)$	844.89	845.58	845.57
$\omega_8(a_1)$	614.02	610.03	609.98
$\omega_9(a_2)$	919.45	902.49	902.59
$\omega_{10}(a_2)$	692.92	688.08	688.05
$\omega_{11}(a_2)$	576.77	565.65	565.53
$\omega_{12}(b_1)$	883.73	875.50	875.51
$\omega_{13}(b_1)$	734.63	725.77	725.77
$\omega_{14}(b_1)$	458.60	453.26	453.25
$\omega_{15}(b_2)$	3267.32	3256.02	3256.02
$\omega_{16}(b_2)$	3222.27	3213.82	3213.82
$\omega_{17}(b_2)$	1541.54	1545.29	1545.33
$\omega_{18}(b_2)$	1273.57	1278.88	1278.88
$\omega_{19}(b_2)$	1105.82	1100.95	1100.90
$\omega_{20}(b_2)$	875.32	878.12	878.12
$\omega_{21}(b_2)$	758.22	759.27	759.27

Table S380: Symmetrized, unnormalized natural internal coordinates for thiophene.

1	$r_{5,6} + r_{3,6} + r_{4,5} + r_{2,3} + r_{2,4}$
2	$3r_{5,6} + r_{3,6} + r_{4,5} - 3r_{2,3} - 3r_{2,4}$
3	$2r_{3,6} - 2r_{4,5} + r_{2,3} - r_{2,4}$
4	$3r_{5,6} - 3r_{3,6} - 3r_{4,5} + r_{2,3} + r_{2,4}$
5	$r_{3,6} - r_{4,5} - 2r_{2,3} + 2r_{2,4}$
6	$r_{3,7} + r_{4,8}$
7	$r_{3,7} - r_{4,8}$
8	$r_{6,10} + r_{5,9}$
9	$r_{6,10} - r_{5,9}$
10	$3\phi_{3,2,4} - 3\phi_{2,3,6} - 3\phi_{2,4,5} + \phi_{3,6,5} + \phi_{4,5,6}$
11	$-\phi_{2,3,6} + \phi_{2,4,5} + 2\phi_{3,6,5} - 2\phi_{4,5,6}$
12	$\phi_{7,3,2} - \phi_{7,3,6} + \phi_{8,4,2} - \phi_{8,4,5}$
13	$\phi_{7,3,2} - \phi_{7,3,6} - \phi_{8,4,2} + \phi_{8,4,5}$
14	$\phi_{10,6,3} - \phi_{10,6,5} + \phi_{9,5,4} - \phi_{9,5,6}$
15	$\phi_{10,6,3} - \phi_{10,6,5} - \phi_{9,5,4} + \phi_{9,5,6}$
16	$3\tau_{3,6,5,4} + \tau_{6,3,2,4} + \tau_{3,2,4,5} - 3\tau_{5,6,3,2} - 3\tau_{2,4,5,6}$
17	$2\tau_{6,3,2,4} - 2\tau_{3,2,4,5} - \tau_{5,6,3,2} + \tau_{2,4,5,6}$
18	$\gamma_{7,3,2,6} + \gamma_{8,4,5,2}$
19	$\gamma_{7,3,2,6} - \gamma_{8,4,5,2}$
20	$\gamma_{10,6,3,5} + \gamma_{9,5,6,4}$
21	$\gamma_{10,6,3,5} - \gamma_{9,5,6,4}$

S4.96 methanol

Table S381: CCSD(T)/cc-pVTZ Optimum Cartesian coordinates (bohr)

1	C	−1.37507311	−0.02427686	0.00000035
2	O	1.30601636	0.12070128	0.00000030
3	H	−2.07787395	1.90961292	−0.00007991
4	H	−2.10898758	−0.97927269	1.68197389
5	H	−2.10898055	−0.97940393	−1.68190173
6	H	1.94117467	−1.57749284	−0.00000131

Table S382: Reference [CCSD(T)/cc-pVTZ] CMA-0A and CMA-2A ($\xi = 0.02$) harmonic frequencies for Level B = MP2/cc-pVTZ and CCSD(T)/cc-pVDZ.

	Reference CCSD(T)/ cc-pVTZ	Pure MP2/ cc-pVTZ	CMA-0A MP2/ cc-pVTZ	CMA-2A MP2/ cc-pVTZ	Pure CCSD(T)/ cc-pVDZ	CMA-0A CCSD(T)/ cc-pVDZ	CMA-2A CCSD(T)/ cc-pVDZ
$\omega_1(a^{'})$	3864.98	3883.16	3864.97	3864.97	3934.93	3864.97	3864.97
$\omega_2(a^{'})$	3128.11	3153.00	3128.03	3128.03	3280.15	3127.99	3127.99
$\omega_3(a^{'})$	3009.29	3023.79	3009.36	3009.36	3142.54	3009.35	3009.35
$\omega_4(a^{'})$	1523.24	1529.06	1523.19	1523.19	1535.29	1523.15	1523.15
$\omega_5(a^{'})$	1487.64	1489.02	1487.66	1487.66	1508.03	1487.73	1487.73
$\omega_6(a^{'})$	1393.88	1383.49	1393.72	1393.72	1409.01	1393.81	1393.89
$\omega_7(a^{'})$	1095.54	1097.63	1095.56	1095.56	1105.59	1095.22	1095.60
$\omega_8(a^{'})$	1065.02	1062.30	1065.28	1065.28	1083.09	1065.66	1065.16
$\omega_9(a^{''})$	3064.49	3090.54	3064.48	3064.48	3212.32	3064.44	3064.44
$\omega_{10}(a^{''})$	1507.67	1514.46	1507.66	1507.66	1519.56	1507.74	1507.74
$\omega_{11}(a^{''})$	1179.62	1182.53	1179.63	1179.63	1190.26	1179.64	1179.65
$\omega_{12}(a^{''})$	304.11	306.45	304.13	304.13	343.42	304.14	304.14

Table S383: CMA-0A and CMA-2A ($\xi = 0.02$) harmonic frequencies targeting CCSD(T)/cc-pVTZ for Level B = B3LYP/6-31G(2*df*,*p*).

	Pure B3LYP/ 6-31G(2*df*,*p*)	CMA-0A B3LYP/ 6-31G(2*df*,*p*)	CMA-2A B3LYP/ 6-31G(2*df*,*p*)
$\omega_1(a^{'})$	3864.98	3864.97	3864.97
$\omega_2(a^{'})$	3149.92	3128.06	3128.06
$\omega_3(a^{'})$	3028.93	3009.28	3009.28
$\omega_4(a^{'})$	1518.99	1523.17	1523.17
$\omega_5(a^{'})$	1483.41	1487.56	1487.56
$\omega_6(a^{'})$	1391.33	1393.94	1393.96
$\omega_7(a^{'})$	1092.21	1095.40	1095.60
$\omega_8(a^{'})$	1049.10	1065.47	1065.23
$\omega_9(a^{''})$	3079.55	3064.48	3064.48
$\omega_{10}(a^{''})$	1505.70	1507.61	1507.61
$\omega_{11}(a^{''})$	1172.61	1179.68	1179.68
$\omega_{12}(a^{''})$	305.10	304.29	304.20

Table S384: Symmetrized, unnormalized natural internal coordinates for methanol.

1	$r_{1,2}$
2	$r_{2,6}$
3	$r_{1,3} + r_{1,4} + r_{1,5}$
4	$r_{1,4} - r_{1,5}$
5	$2r_{1,3} - r_{1,4} - r_{1,5}$
6	$\phi_{1,2,6}$
7	$2\phi_{3,1,2} - \phi_{4,1,2} - \phi_{5,1,2}$
8	$\phi_{3,1,2} + \phi_{4,1,2} + \phi_{5,1,2}$
9	$\phi_{4,1,2} - \phi_{5,1,2}$
10	$-\phi_{3,1,4} - \phi_{3,1,5} + 2\phi_{4,1,5}$
11	$\phi_{3,1,4} - \phi_{3,1,5}$
12	$\tau_{3,1,2,6}$

S4.97 propene

Table S385: CCSD(T)/cc-pVTZ Optimum Cartesian coordinates (bohr)

1	C	−2.37687297	0.33833882	0.00000007
2	C	0.19870321	−0.85510046	−0.00000006
3	C	2.37314150	0.43515597	0.00000003
4	H	0.26291668	−2.90753182	−0.00000011
5	H	4.18218979	−0.52060166	−0.00000005
6	H	2.38999877	2.48486451	0.00000016
7	H	−2.23672093	2.39600543	−0.00000030
8	H	−3.45993967	−0.24053555	−1.66145087
9	H	−3.45993980	−0.24053625	1.66145068

Table S386: Reference [CCSD(T)/cc-pVTZ] CMA-0A and CMA-2A (ξ = 0.02) harmonic frequencies for Level B = MP2/cc-pVTZ and CCSD(T)/cc-pVDZ.

	Reference CCSD(T)/ cc-pVTZ	Pure MP2/ cc-pVTZ	CMA-0A MP2/ cc-pVTZ	CMA-2A MP2/ cc-pVTZ	Pure CCSD(T)/ cc-pVDZ	CMA-0A CCSD(T)/ cc-pVDZ	CMA-2A CCSD(T)/ cc-pVDZ
$\omega_1(a^{'})$	3230.27	3252.34	3230.27	3230.27	3384.61	3230.23	3230.23
$\omega_2(a^{'})$	3151.66	3170.07	3151.53	3151.53	3296.91	3151.65	3151.65
$\omega_3(a^{'})$	3138.19	3154.55	3138.26	3138.26	3279.75	3138.11	3138.11
$\omega_4(a^{'})$	3113.17	3137.28	3113.20	3113.20	3263.39	3113.22	3113.22
$\omega_5(a^{'})$	3029.48	3042.19	3029.51	3029.51	3161.65	3029.47	3029.47
$\omega_6(a^{'})$	1696.39	1688.41	1696.34	1696.36	1762.85	1695.53	1695.90
$\omega_7(a^{'})$	1501.66	1503.15	1501.66	1501.66	1512.85	1501.49	1501.49
$\omega_8(a^{'})$	1455.83	1453.34	1455.66	1455.66	1477.19	1456.25	1456.25
$\omega_9(a^{'})$	1408.10	1401.19	1408.25	1408.25	1427.02	1408.08	1408.08
$\omega_{10}(a^{'})$	1320.29	1319.32	1320.34	1320.31	1340.13	1320.86	1320.38
$\omega_{11}(a^{'})$	1191.64	1187.57	1191.68	1191.69	1210.06	1191.66	1191.85
$\omega_{12}(a^{'})$	942.38	939.57	942.39	942.39	960.44	941.12	941.12
$\omega_{13}(a^{'})$	930.94	929.75	930.97	930.97	956.81	932.83	932.59
$\omega_{14}(a^{'})$	418.07	417.01	418.08	418.08	427.33	418.14	418.14
$\omega_{15}(a^{''})$	3089.80	3115.11	3089.80	3089.80	3240.61	3089.77	3089.77
$\omega_{16}(a^{''})$	1488.14	1489.92	1488.13	1488.13	1498.45	1488.18	1488.18
$\omega_{17}(a^{''})$	1067.99	1066.45	1067.98	1067.99	1077.74	1067.98	1068.01
$\omega_{18}(a^{''})$	1014.26	1029.36	1014.22	1014.26	1027.57	1014.28	1014.26
$\omega_{19}(a^{''})$	925.24	931.87	925.24	925.24	935.55	925.26	925.26
$\omega_{20}(a^{''})$	582.65	587.09	582.77	582.69	587.71	582.67	582.66
$\omega_{21}(a^{''})$	199.43	203.88	199.44	199.44	201.25	199.47	199.45

Table S387: CMA-0A and CMA-2A (ξ = 0.02) harmonic frequencies targeting CCSD(T)/cc-pVTZ for Level B = B3LYP/6-31G(2*df*,*p*).

	Pure B3LYP/ 6-31G(2*df*,*p*)	CMA-0A B3LYP/ 6-31G(2*df*,*p*)	CMA-2A B3LYP/ 6-31G(2*df*,*p*)
$\omega_1(a^{'})$	3244.41	3230.27	3230.27
$\omega_2(a^{'})$	3163.35	3151.47	3151.47
$\omega_3(a^{'})$	3150.55	3138.27	3138.27
$\omega_4(a^{'})$	3128.21	3113.23	3113.23
$\omega_5(a^{'})$	3047.71	3029.46	3029.46
$\omega_6(a^{'})$	1692.38	1695.99	1696.11
$\omega_7(a^{'})$	1497.94	1501.48	1501.48
$\omega_8(a^{'})$	1448.12	1455.67	1455.67
$\omega_9(a^{'})$	1410.82	1408.43	1408.43
$\omega_{10}(a^{'})$	1324.00	1320.48	1320.33
$\omega_{11}(a^{'})$	1191.13	1191.73	1191.73
$\omega_{12}(a^{'})$	946.35	942.41	942.41
$\omega_{13}(a^{'})$	924.92	931.52	931.52
$\omega_{14}(a^{'})$	424.69	418.11	418.11
$\omega_{15}(a^{''})$	3102.53	3089.80	3089.80
$\omega_{16}(a^{''})$	1484.24	1488.05	1488.06
$\omega_{17}(a^{''})$	1074.72	1067.13	1067.14
$\omega_{18}(a^{''})$	1030.49	1015.00	1015.00
$\omega_{19}(a^{''})$	943.32	925.36	925.36
$\omega_{20}(a^{''})$	590.64	582.96	582.96
$\omega_{21}(a^{''})$	203.54	199.56	199.48

Table S388: Symmetrized, unnormalized natural internal coordinates for propene.

1	$r_{1,2}$
2	$r_{2,3}$
3	$r_{1,7} + r_{1,8} + r_{1,9}$
4	$2r_{1,7} - r_{1,8} - r_{1,9}$
5	$r_{1,8} - r_{1,9}$
6	$r_{2,4}$
7	$r_{3,5} + r_{3,6}$
8	$r_{3,5} - r_{3,6}$
9	$2\phi_{1,2,3} - \phi_{4,2,1} - \phi_{4,2,3}$
10	$\phi_{4,2,1} - \phi_{4,2,3}$
11	$\phi_{7,1,2} + \phi_{8,1,2} + \phi_{9,1,2} - \phi_{8,1,9} - \phi_{7,1,8} - \phi_{7,1,9}$
12	$2\phi_{7,1,2} - \phi_{8,1,2} - \phi_{9,1,2}$
13	$\phi_{8,1,2} - \phi_{9,1,2}$
14	$2\phi_{8,1,9} - \phi_{7,1,8} - \phi_{7,1,9}$
15	$\phi_{7,1,8} - \phi_{7,1,9}$
16	$2\phi_{5,3,6} - \phi_{5,3,2} - \phi_{6,3,2}$
17	$\phi_{5,3,2} - \phi_{6,3,2}$
18	$\tau_{7,1,2,3} + \tau_{8,1,2,3} + \tau_{9,1,2,3}$
19	$\tau_{5,3,2,1} + \tau_{6,3,2,1}$
20	$\gamma_{4,2,1,3}$
21	$\gamma_{2,3,5,6}$

S4.98 oxirane

Table S389: CCSD(T)/cc-pVTZ Optimum Cartesian coordinates (bohr)

1	O	0.00000000	−0.00000000	1.51705162
2	C	0.00000000	−1.38721508	−0.80610774
3	C	−0.00000000	1.38721508	−0.80610774
4	H	−1.73830814	−2.39048176	−1.22008415
5	H	1.73830814	−2.39048176	−1.22008415
6	H	−1.73830814	2.39048176	−1.22008415
7	H	1.73830814	2.39048176	−1.22008415

Table S390: Reference [CCSD(T)/cc-pVTZ] CMA-0A and CMA-2A ($\xi = 0.02$) harmonic frequencies for Level B = MP2/cc-pVTZ and CCSD(T)/cc-pVDZ.

	Reference CCSD(T)/ cc-pVTZ	Pure MP2/ cc-pVTZ	CMA-0A MP2/ cc-pVTZ	CMA-2A MP2/ cc-pVTZ	Pure CCSD(T)/ cc-pVDZ	CMA-0A CCSD(T)/ cc-pVDZ	CMA-2A CCSD(T)/ cc-pVDZ
$\omega_1(a_1)$	3117.42	3132.89	3117.41	3117.41	3254.14	3117.40	3117.40
$\omega_2(a_1)$	1549.96	1548.52	1549.91	1549.91	1577.30	1549.12	1549.13
$\omega_3(a_1)$	1300.14	1301.10	1300.15	1300.18	1332.79	1300.83	1300.87
$\omega_4(a_1)$	1157.92	1150.36	1157.89	1157.86	1178.14	1157.92	1157.93
$\omega_5(a_1)$	899.68	900.96	899.78	899.78	920.80	900.18	900.10
$\omega_6(a_2)$	3210.77	3233.78	3210.76	3210.76	3357.92	3210.75	3210.75
$\omega_7(a_2)$	1175.07	1175.40	1175.07	1175.07	1185.51	1175.00	1175.07
$\omega_8(a_2)$	816.26	819.76	816.27	816.27	831.51	816.40	816.30
$\omega_9(b_1)$	3196.11	3219.80	3196.11	3196.11	3342.68	3196.11	3196.11
$\omega_{10}(b_1)$	1176.57	1175.53	1176.58	1176.58	1191.61	1176.43	1176.57
$\omega_{11}(b_1)$	1052.00	1054.88	1052.00	1052.00	1058.46	1052.17	1052.01
$\omega_{12}(b_2)$	3109.14	3125.29	3109.14	3109.14	3243.70	3109.13	3109.13
$\omega_{13}(b_2)$	1513.26	1516.14	1513.26	1513.26	1527.74	1513.26	1513.26
$\omega_{14}(b_2)$	1156.67	1151.71	1156.61	1156.67	1167.24	1156.65	1156.65
$\omega_{15}(b_2)$	849.93	856.01	850.01	849.94	864.94	850.00	850.00

Table S391: CMA-0A and CMA-2A ($\xi = 0.02$) harmonic frequencies targeting CCSD(T)/cc-pVTZ for Level B = B3LYP/6-31G(2*df*,*p*).

	Pure B3LYP/ 6-31G(2*df*,*p*)	CMA-0A B3LYP/ 6-31G(2*df*,*p*)	CMA-2A B3LYP/ 6-31G(2*df*,*p*)
$\omega_1(a_1)$	3131.15	3117.39	3117.39
$\omega_2(a_1)$	1537.79	1549.95	1549.95
$\omega_3(a_1)$	1302.69	1300.02	1300.17
$\omega_4(a_1)$	1153.94	1158.10	1157.93
$\omega_5(a_1)$	898.22	899.72	899.72
$\omega_6(a_2)$	3228.15	3210.76	3210.76
$\omega_7(a_2)$	1165.24	1175.02	1175.07
$\omega_8(a_2)$	817.64	816.34	816.27
$\omega_9(b_1)$	3211.37	3196.11	3196.11
$\omega_{10}(b_1)$	1177.61	1176.57	1176.58
$\omega_{11}(b_1)$	1042.30	1052.01	1052.00
$\omega_{12}(b_2)$	3123.09	3109.11	3109.11
$\omega_{13}(b_2)$	1502.43	1513.13	1513.13
$\omega_{14}(b_2)$	1155.15	1156.73	1156.73
$\omega_{15}(b_2)$	846.43	850.18	850.18

Table S392: Symmetrized, unnormalized natural internal coordinates for oxirane.

1	$r_{1,2} + r_{1,3} + r_{2,3}$
2	$r_{1,2} - r_{1,3}$
3	$-r_{1,2} - r_{1,3} + 2r_{2,3}$
4	$r_{2,4} + r_{2,5} + r_{3,6} + r_{3,7}$
5	$r_{2,4} + r_{2,5} - r_{3,6} - r_{3,7}$
6	$r_{2,4} - r_{2,5} + r_{3,6} - r_{3,7}$
7	$r_{2,4} - r_{2,5} - r_{3,6} + r_{3,7}$
8	$4\phi_{4,2,5} + 4\phi_{6,3,7} - \phi_{1,2,4} - \phi_{3,2,4} - \phi_{1,2,5} - \phi_{3,2,5} - \phi_{1,3,6} - \phi_{2,3,6} - \phi_{1,3,7} - \phi_{2,3,7}$
9	$4\phi_{4,2,5} - 4\phi_{6,3,7} - \phi_{1,2,4} - \phi_{3,2,4} - \phi_{1,2,5} - \phi_{3,2,5} + \phi_{1,3,6} + \phi_{2,3,6} + \phi_{1,3,7} + \phi_{2,3,7}$
10	$\phi_{1,2,4} + \phi_{3,2,4} - \phi_{1,2,5} - \phi_{3,2,5} + \phi_{1,3,6} + \phi_{2,3,6} - \phi_{1,3,7} - \phi_{2,3,7}$
11	$\phi_{1,2,4} + \phi_{3,2,4} - \phi_{1,2,5} - \phi_{3,2,5} - \phi_{1,3,6} - \phi_{2,3,6} + \phi_{1,3,7} + \phi_{2,3,7}$
12	$\phi_{1,2,4} - \phi_{3,2,4} + \phi_{1,2,5} - \phi_{3,2,5} + \phi_{1,3,6} - \phi_{2,3,6} + \phi_{1,3,7} - \phi_{2,3,7}$
13	$\phi_{1,2,4} - \phi_{3,2,4} + \phi_{1,2,5} - \phi_{3,2,5} - \phi_{1,3,6} + \phi_{2,3,6} - \phi_{1,3,7} + \phi_{2,3,7}$
14	$\phi_{1,2,4} - \phi_{3,2,4} - \phi_{1,2,5} + \phi_{3,2,5} + \phi_{1,3,6} - \phi_{2,3,6} - \phi_{1,3,7} + \phi_{2,3,7}$
15	$\phi_{1,2,4} - \phi_{3,2,4} - \phi_{1,2,5} + \phi_{3,2,5} - \phi_{1,3,6} + \phi_{2,3,6} + \phi_{1,3,7} - \phi_{2,3,7}$

S4.99 hydrogen cyanide

Table S393: CCSD(T)/cc-pVTZ Optimum Cartesian coordinates (bohr)

1	X	−0.00000000	−1.00000000	−1.06129487
2	C	−0.00000000	0.00000000	−1.06129487
3	X	−1.00000000	0.00000000	−1.06129487
4	H	−0.00000000	−0.00000000	−3.07732615
5	N	0.00000000	−0.00000000	1.13096201

Table S394: Reference [CCSD(T)/cc-pVTZ] CMA-0A and CMA-2A (ξ = 0.02) harmonic frequencies for Level B = MP2/cc-pVTZ and CCSD(T)/cc-pVDZ.

	Reference CCSD(T)/ cc-pVTZ	Pure MP2/ cc-pVTZ	CMA-0A MP2/ cc-pVTZ	CMA-2A MP2/ cc-pVTZ	Pure CCSD(T)/ cc-pVDZ	CMA-0A CCSD(T)/ cc-pVDZ	CMA-2A CCSD(T)/ cc-pVDZ
$\omega_1(\sigma^+)$	3443.43	3455.99	3443.35	3443.35	3603.62	3443.41	3443.41
$\omega_{2a}(\pi)$	716.01	723.19	716.01	716.01	736.37	716.01	716.01
$\omega_{2b}(\pi)$	716.01	723.18	716.01	716.01	736.37	716.01	716.01
$\omega_3(\sigma^+)$	2111.38	2072.67	2111.51	2111.51	2206.41	2111.41	2111.41

Table S395: CMA-0A and CMA-2A (ξ = 0.02) harmonic frequencies targeting CCSD(T)/cc-pVTZ for Level B = B3LYP/6-31G(2*df*,*p*).

	Pure B3LYP/ 6-31G(2*df*,*p*)	CMA-0A B3LYP/ 6-31G(2*df*,*p*)	CMA-2A B3LYP/ 6-31G(2*df*,*p*)
$\omega_1(\sigma^+)$	3476.33	3443.42	3443.42
$\omega_{2a}(\pi)$	776.85	716.01	716.01
$\omega_{2b}(\pi)$	776.82	716.01	716.01
$\omega_3(\sigma^+)$	2147.46	2111.39	2111.39

Table S396: Symmetrized, unnormalized natural internal coordinates for hydrogen cyanide.

1	$r_{2,4}$
2	$r_{2,5}$
3	$\theta_{4,2,5,1}$
4	$\theta_{4,2,5,3}$

S4.100 triplet methylene

Table S397: CCSD(T)/cc-pVTZ Optimum Cartesian coordinates (bohr)

1	C	0.00000000	0.00000000	0.11580983
2	H	0.00000000	−1.87219122	−0.68946391
3	H	0.00000000	1.87219122	−0.68946391

Table S398: Reference [CCSD(T)/cc-pVTZ] CMA-0A and CMA-2A ($\xi = 0.02$) harmonic frequencies for Level B = MP2/cc-pVTZ and CCSD(T)/cc-pVDZ.

	Reference CCSD(T)/ cc-pVTZ	Pure MP2/ cc-pVTZ	CMA-0A MP2/ cc-pVTZ	CMA-2A MP2/ cc-pVTZ	Pure CCSD(T)/ cc-pVDZ	CMA-0A CCSD(T)/ cc-pVDZ	CMA-2A CCSD(T)/ cc-pVDZ
$\omega_1(a_1)$	3139.22	3154.90	3139.22	3139.22	3296.46	3139.20	3139.20
$\omega_2(a_1)$	1105.77	1122.31	1105.77	1105.77	1124.62	1105.83	1105.83
$\omega_3(b_2)$	3365.40	3391.02	3365.40	3365.40	3534.89	3365.40	3365.40

Table S399: CMA-0A and CMA-2A ($\xi = 0.02$) harmonic frequencies targeting CCSD(T)/cc-pVTZ for Level B = B3LYP/6-31G(2df,p).

	Pure B3LYP/ 6-31G(2df,p)	CMA-0A B3LYP/ 6-31G(2df,p)	CMA-2A B3LYP/ 6-31G(2df,p)
$\omega_1(a_1)$	3152.26	3139.16	3139.22
$\omega_2(a_1)$	1092.86	1105.94	1105.77
$\omega_3(b_2)$	3392.18	3365.40	3365.40

Table S400: Symmetrized, unnormalized natural internal coordinates for triplet methylene.

1	$r_{1,2} + r_{1,3}$
2	$r_{1,2} - r_{1,3}$
3	$\phi_{2,1,3}$

S4.101 formyl radical

Table S401: CCSD(T)/cc-pVTZ Optimum Cartesian coordinates (bohr)

1	C	−1.17736294	0.18900598	0.00000000
2	H	−2.55461370	−1.42021423	0.00000000
3	O	1.04426684	−0.05231315	0.00000000

Table S402: Reference [CCSD(T)/cc-pVTZ] CMA-0A and CMA-2A ($\xi = 0.02$) harmonic frequencies for Level B = MP2/cc-pVTZ and CCSD(T)/cc-pVDZ.

	Reference CCSD(T)/ cc-pVTZ	Pure MP2/ cc-pVTZ	CMA-0A MP2/ cc-pVTZ	CMA-2A MP2/ cc-pVTZ	Pure CCSD(T)/ cc-pVDZ	CMA-0A CCSD(T)/ cc-pVDZ	CMA-2A CCSD(T)/ cc-pVDZ
$\omega_1(a^{'})$	2691.42	2706.97	2691.37	2691.37	2829.67	2691.39	2691.39
$\omega_2(a^{'})$	1888.38	1898.35	1888.27	1888.45	1942.88	1888.34	1888.38
$\omega_3(a^{'})$	1122.70	1077.12	1123.00	1122.70	1143.81	1122.84	1122.76

Table S403: CMA-0A and CMA-2A ($\xi = 0.02$) harmonic frequencies targeting CCSD(T)/cc-pVTZ for Level B = B3LYP/6-31G(2*df*,*p*).

	Pure B3LYP/ 6-31G(2*df*,*p*)	CMA-0A B3LYP/ 6-31G(2*df*,*p*)	CMA-2A B3LYP/ 6-31G(2*df*,*p*)
$\omega_1(a^{'})$	2716.84	2691.42	2691.42
$\omega_2(a^{'})$	1912.60	1888.38	1888.38
$\omega_3(a^{'})$	1105.62	1122.70	1122.70

Table S404: Symmetrized, unnormalized natural internal coordinates for formyl radical.

1	$r_{1,2}$
2	$r_{1,3}$
3	$\phi_{2,1,3}$

S4.102 vinyl radical

Table S405: CCSD(T)/cc-pVTZ Optimum Cartesian coordinates (bohr)

1	C	1.17395959	−0.03986833	0.00000000
2	H	2.10304999	−1.87918602	0.00000000
3	H	2.39183520	1.60942624	0.00000000
4	C	−1.30772223	0.15626308	0.00000000
5	H	−2.90219632	−1.11613248	0.00000000

Table S406: Reference [CCSD(T)/cc-pVTZ] CMA-0A and CMA-2A (ξ = 0.02) harmonic frequencies for Level B = MP2/cc-pVTZ and CCSD(T)/cc-pVDZ.

	Reference CCSD(T)/ cc-pVTZ	Pure MP2/ cc-pVTZ	CMA-0A MP2/ cc-pVTZ	CMA-2A MP2/ cc-pVTZ	Pure CCSD(T)/ cc-pVDZ	CMA-0A CCSD(T)/ cc-pVDZ	CMA-2A CCSD(T)/ cc-pVDZ
$\omega_1(a^{'})$	3246.53	3263.15	3246.51	3246.51	3402.80	3246.52	3246.52
$\omega_2(a^{'})$	3178.90	3204.67	3178.70	3178.70	3335.88	3178.70	3178.70
$\omega_3(a^{'})$	3074.49	3099.49	3074.69	3074.69	3220.87	3074.65	3074.65
$\omega_4(a^{'})$	1614.03	1615.88	1614.06	1614.07	1688.83	1613.26	1614.08
$\omega_5(a^{'})$	1395.98	1398.31	1395.98	1395.98	1413.91	1396.91	1395.97
$\omega_6(a^{'})$	1070.93	1067.67	1070.84	1070.84	1086.60	1070.95	1070.95
$\omega_7(a^{'})$	726.00	722.51	726.16	726.15	740.00	726.06	726.06
$\omega_8(a^{''})$	914.78	924.29	914.68	914.78	920.82	914.60	914.60
$\omega_9(a^{''})$	806.12	818.34	806.24	806.12	815.55	806.33	806.33

Table S407: CMA-0A and CMA-2A (ξ = 0.02) harmonic frequencies targeting CCSD(T)/cc-pVTZ for Level B = B3LYP/6-31G(2df,p).

	Pure B3LYP/ 6-31G(2df,p)	CMA-0A B3LYP/ 6-31G(2df,p)	CMA-2A B3LYP/ 6-31G(2df,p)
$\omega_1(a^{'})$	3257.03	3246.50	3246.50
$\omega_2(a^{'})$	3192.87	3178.90	3178.90
$\omega_3(a^{'})$	3088.33	3074.47	3074.47
$\omega_4(a^{'})$	1615.72	1613.39	1614.05
$\omega_5(a^{'})$	1390.68	1396.71	1395.97
$\omega_6(a^{'})$	1069.55	1070.98	1070.99
$\omega_7(a^{'})$	725.69	726.16	726.08
$\omega_8(a^{''})$	929.47	914.69	914.69
$\omega_9(a^{''})$	804.26	806.23	806.23

Table S408: Symmetrized, unnormalized natural internal coordinates for vinyl radical.

1	$r_{1,2} + r_{1,3}$
2	$r_{1,2} - r_{1,3}$
3	$r_{1,4}$
4	$r_{4,5}$
5	$\phi_{1,4,5}$
6	$2\phi_{2,1,3} - \phi_{2,1,4} - \phi_{3,1,4}$
7	$\phi_{2,1,4} - \phi_{3,1,4}$
8	$\tau_{2,1,4,5} + \tau_{3,1,4,5}$
9	$\gamma_{4,1,2,3}$

S4.103 acetyl radical

Table S409: CCSD(T)/cc-pVTZ Optimum Cartesian coordinates (bohr)

1	C	−0.25873690	0.80450304	0.00000000
2	O	−2.19121944	−0.33285589	0.00000000
3	C	2.40971826	−0.24563821	0.00000000
4	H	2.38285691	−2.30895956	0.00000000
5	H	3.39100957	0.46865813	−1.66422836
6	H	3.39100957	0.46865813	1.66422836

Table S410: Reference [CCSD(T)/cc-pVTZ] CMA-0A and CMA-2A ($\xi = 0.02$) harmonic frequencies for Level B = MP2/cc-pVTZ and CCSD(T)/cc-pVDZ.

	Reference CCSD(T)/ cc-pVTZ	Pure MP2/ cc-pVTZ	CMA-0A MP2/ cc-pVTZ	CMA-2A MP2/ cc-pVTZ	Pure CCSD(T)/ cc-pVDZ	CMA-0A CCSD(T)/ cc-pVDZ	CMA-2A CCSD(T)/ cc-pVDZ
$\omega_1(a^{'})$	3137.39	3159.37	3137.38	3137.38	3287.71	3137.25	3137.25
$\omega_2(a^{'})$	3039.37	3052.44	3039.38	3039.38	3172.30	3039.45	3039.45
$\omega_3(a^{'})$	1899.32	1911.57	1899.29	1899.31	1956.44	1899.27	1899.28
$\omega_4(a^{'})$	1468.66	1471.61	1468.61	1468.61	1478.81	1468.73	1468.73
$\omega_5(a^{'})$	1355.85	1349.67	1355.84	1355.85	1373.86	1355.65	1355.65
$\omega_6(a^{'})$	1050.41	1040.22	1050.28	1050.41	1063.43	1049.86	1049.85
$\omega_7(a^{'})$	861.02	859.24	861.13	861.12	896.74	862.24	862.24
$\omega_8(a^{'})$	466.47	462.95	466.86	466.49	468.83	466.50	466.49
$\omega_9(a^{''})$	3143.08	3166.75	3143.08	3143.08	3294.28	3143.05	3143.05
$\omega_{10}(a^{''})$	1467.61	1470.71	1467.61	1467.61	1481.13	1467.63	1467.63
$\omega_{11}(a^{''})$	954.71	953.10	954.72	954.72	964.17	954.80	954.80
$\omega_{12}(a^{''})$	98.52	90.81	98.61	98.60	111.07	98.56	98.56

Table S411: CMA-0A and CMA-2A ($\xi = 0.02$) harmonic frequencies targeting CCSD(T)/cc-pVTZ for Level B = B3LYP/6-31G(2*df*,*p*).

	Pure B3LYP/ 6-31G(2*df*,*p*)	CMA-0A B3LYP/ 6-31G(2*df*,*p*)	CMA-2A B3LYP/ 6-31G(2*df*,*p*)
$\omega_1(a^{'})$	3153.68	3137.29	3137.29
$\omega_2(a^{'})$	3056.59	3039.41	3039.41
$\omega_3(a^{'})$	1916.45	1899.20	1899.33
$\omega_4(a^{'})$	1462.95	1468.59	1468.59
$\omega_5(a^{'})$	1355.90	1355.70	1355.93
$\omega_6(a^{'})$	1051.48	1050.30	1050.31
$\omega_7(a^{'})$	841.34	861.92	861.37
$\omega_8(a^{'})$	460.62	466.70	466.50
$\omega_9(a^{''})$	3158.92	3143.08	3143.08
$\omega_{10}(a^{''})$	1461.45	1467.52	1467.52
$\omega_{11}(a^{''})$	948.48	954.85	954.85
$\omega_{12}(a^{''})$	112.00	98.65	98.52

Table S412: Symmetrized, unnormalized natural internal coordinates for acetyl radical.

1	$r_{3,1}$
2	$r_{1,2}$
3	$r_{3,4} + r_{3,5} + r_{3,6}$
4	$2r_{3,4} - r_{3,5} - r_{3,6}$
5	$r_{3,5} - r_{3,6}$
6	$\phi_{3,1,2}$
7	$\phi_{4,3,1} + \phi_{5,3,1} + \phi_{6,3,1} - \phi_{5,3,6} - \phi_{4,3,5} - \phi_{4,3,6}$
8	$2\phi_{4,3,1} - \phi_{5,3,1} - \phi_{6,3,1}$
9	$\phi_{5,3,1} - \phi_{6,3,1}$
10	$2\phi_{5,3,6} - \phi_{4,3,5} - \phi_{4,3,6}$
11	$\phi_{4,3,5} - \phi_{4,3,6}$
12	$\tau_{4,3,1,2}$

S4.104 hydroxymethyl radical

Table S413: CCSD(T)/cc-pVTZ Optimum Cartesian coordinates (bohr)

1	C	−1.37903910	0.02323044	0.05754946
2	O	1.20513902	−0.11712615	−0.00535801
3	H	1.86898304	1.56393483	0.15950651
4	H	−2.27904852	1.78239326	−0.47148778
5	H	−2.29638299	−1.76405192	−0.28821473

Table S414: Reference [CCSD(T)/cc-pVTZ] CMA-0A and CMA-2A (ξ = 0.02) harmonic frequencies for Level B = MP2/cc-pVTZ and CCSD(T)/cc-pVDZ.

	Reference CCSD(T)/ cc-pVTZ	Pure MP2/ cc-pVTZ	CMA-0A MP2/ cc-pVTZ	CMA-2A MP2/ cc-pVTZ	Pure CCSD(T)/ cc-pVDZ	CMA-0A CCSD(T)/ cc-pVDZ	CMA-2A CCSD(T)/ cc-pVDZ
$\omega_1(a)$	3861.95	3878.86	3861.95	3861.95	3933.21	3861.95	3861.95
$\omega_2(a)$	3280.03	3305.28	3280.01	3280.01	3436.56	3280.02	3280.02
$\omega_3(a)$	3139.51	3156.76	3139.52	3139.52	3285.35	3139.50	3139.50
$\omega_4(a)$	1498.77	1504.89	1498.71	1498.72	1522.11	1498.50	1498.79
$\omega_5(a)$	1383.73	1375.68	1383.54	1383.58	1396.11	1383.64	1383.74
$\omega_6(a)$	1209.21	1205.02	1209.26	1209.20	1236.77	1209.49	1209.05
$\omega_7(a)$	1064.75	1067.31	1065.03	1065.03	1073.65	1064.95	1064.95
$\omega_8(a)$	620.09	613.53	620.00	620.12	619.89	619.93	620.16
$\omega_9(a)$	432.11	433.96	432.33	432.13	444.20	432.58	432.13

Table S415: CMA-0A and CMA-2A (ξ = 0.02) harmonic frequencies targeting CCSD(T)/cc-pVTZ for Level B = B3LYP/6-31G(2df,p).

	Pure B3LYP/ 6-31G(2df,p)	CMA-0A B3LYP/ 6-31G(2df,p)	CMA-2A B3LYP/ 6-31G(2df,p)
$\omega_1(a)$	3861.92	3861.95	3861.95
$\omega_2(a)$	3301.34	3280.01	3280.01
$\omega_3(a)$	3153.83	3139.49	3139.49
$\omega_4(a)$	1483.76	1498.73	1498.74
$\omega_5(a)$	1374.10	1383.73	1383.78
$\omega_6(a)$	1207.68	1209.22	1209.16
$\omega_7(a)$	1057.26	1064.88	1064.88
$\omega_8(a)$	600.34	620.13	620.13
$\omega_9(a)$	430.97	432.22	432.16

Table S416: Symmetrized, unnormalized natural internal coordinates for hydroxymethyl radical.

1	$r_{1,4} + r_{1,5}$
2	$r_{1,4} - r_{1,5}$
3	$r_{1,2}$
4	$r_{2,3}$
5	$\phi_{1,2,3}$
6	$2\phi_{4,1,5} - \phi_{4,1,2} - \phi_{5,1,2}$
7	$\phi_{4,1,2} - \phi_{5,1,2}$
8	$\tau_{4,1,2,3} + \tau_{5,1,2,3}$
9	$\gamma_{2,1,4,5}$

S4.105 triplet silylene

Table S417: CCSD(T)/cc-pVTZ Optimum Cartesian coordinates (bohr)

1	Si	0.00000000	0.00000000	0.09643776
2	H	0.00000000	−2.40430265	−1.33854198
3	H	0.00000000	2.40430265	−1.33854198

Table S418: Reference [CCSD(T)/cc-pVTZ] CMA-0A and CMA-2A (ξ = 0.02) harmonic frequencies for Level B = MP2/cc-pVTZ and CCSD(T)/cc-pVDZ.

	Reference CCSD(T)/ cc-pVTZ	Pure MP2/ cc-pVTZ	CMA-0A MP2/ cc-pVTZ	CMA-2A MP2/ cc-pVTZ	Pure CCSD(T)/ cc-pVDZ	CMA-0A CCSD(T)/ cc-pVDZ	CMA-2A CCSD(T)/ cc-pVDZ
$\omega_1(a_1)$	2189.75	2230.91	2189.75	2189.75	2224.25	2189.75	2189.75
$\omega_2(a_1)$	890.15	908.08	890.16	890.16	894.72	890.15	890.15
$\omega_3(b_2)$	2249.86	2289.00	2249.86	2249.86	2283.77	2249.86	2249.86

Table S419: CMA-0A and CMA-2A (ξ = 0.02) harmonic frequencies targeting CCSD(T)/cc-pVTZ for Level B = B3LYP/6-31G(2*df*,*p*).

	Pure B3LYP/ 6-31G(2*df*,*p*)	CMA-0A B3LYP/ 6-31G(2*df*,*p*)	CMA-2A B3LYP/ 6-31G(2*df*,*p*)
$\omega_1(a_1)$	2196.14	2189.73	2189.73
$\omega_2(a_1)$	894.56	890.20	890.20
$\omega_3(b_2)$	2266.32	2249.86	2249.86

Table S420: Symmetrized, unnormalized natural internal coordinates for triplet silylene.

1	$r_{1,2} + r_{1,3}$
2	$r_{1,2} - r_{1,3}$
3	$\phi_{2,1,3}$

S4.106 silyl radical

Table S421: CCSD(T)/cc-pVTZ Optimum Cartesian coordinates (bohr)

1	X	−1.97286216	−0.00000000	0.00000000
2	SI	−0.08313564	−0.00000000	0.00000000
3	H	0.76927368	−1.33284055	−2.30854755
4	H	0.76927368	2.66568110	0.00000000
5	H	0.76927368	−1.33284055	2.30854755

Table S422: Reference [CCSD(T)/cc-pVTZ] CMA-0A and CMA-2A ($\xi = 0.02$) harmonic frequencies for Level B = MP2/cc-pVTZ and CCSD(T)/cc-pVDZ.

	Reference CCSD(T)/ cc-pVTZ	Pure MP2/ cc-pVTZ	CMA-0A MP2/ cc-pVTZ	CMA-2A MP2/ cc-pVTZ	Pure CCSD(T)/ cc-pVDZ	CMA-0A CCSD(T)/ cc-pVDZ	CMA-2A CCSD(T)/ cc-pVDZ
$\omega_1(a_1)$	2217.25	2252.32	2217.25	2217.25	2247.99	2217.25	2217.25
$\omega_2(a_1)$	778.72	799.51	778.72	778.72	788.20	778.73	778.73
$\omega_{3\mathrm{a}}(e)$	2250.40	2287.37	2250.39	2250.39	2283.33	2250.40	2250.40
$\omega_{3\mathrm{b}}(e)$	2250.40	2287.32	2250.39	2250.39	2283.29	2250.40	2250.40
$\omega_{4\mathrm{a}}(e)$	943.18	958.95	943.19	943.19	947.17	943.18	943.18
$\omega_{4\mathrm{b}}(e)$	943.18	958.94	943.19	943.19	947.16	943.18	943.18

Table S423: CMA-0A and CMA-2A ($\xi = 0.02$) harmonic frequencies targeting CCSD(T)/cc-pVTZ for Level B = B3LYP/6-31G(2*df*,*p*).

	Pure B3LYP/ 6-31G(2*df*,*p*)	CMA-0A B3LYP/ 6-31G(2*df*,*p*)	CMA-2A B3LYP/ 6-31G(2*df*,*p*)
$\omega_1(a_1)$	2226.23	2217.23	2217.23
$\omega_2(a_1)$	761.93	778.78	778.78
$\omega_{3\mathrm{a}}(e)$	2267.86	2250.39	2250.39
$\omega_{3\mathrm{b}}(e)$	2267.34	2250.39	2250.39
$\omega_{4\mathrm{a}}(e)$	939.55	943.19	943.19
$\omega_{4\mathrm{b}}(e)$	939.12	943.19	943.19

Table S424: Symmetrized, unnormalized natural internal coordinates for silyl radical.

1	$r_{2,3} + r_{2,4} + r_{2,5}$
2	$2r_{2,3} - r_{2,4} - r_{2,5}$
3	$r_{2,4} - r_{2,5}$
4	$2\phi_{3,2,4} - \phi_{3,2,5} - \phi_{4,2,5}$
5	$\phi_{3,2,5} - \phi_{4,2,5}$
6	$\gamma_{3,2,4,5} + \gamma_{4,2,5,3} + \gamma_{5,2,3,4}$

S4.107 phosphino radical

Table S425: CCSD(T)/cc-pVTZ Optimum Cartesian coordinates (bohr)

1	P	0.00000000	0.00000000	0.11404634
2	H	0.00000000	−1.92750368	−1.75250871
3	H	0.00000000	1.92750368	−1.75250871

Table S426: Reference [CCSD(T)/cc-pVTZ] CMA-0A and CMA-2A (ξ = 0.02) harmonic frequencies for Level B = MP2/cc-pVTZ and CCSD(T)/cc-pVDZ.

	Reference CCSD(T)/ cc-pVTZ	Pure MP2/ cc-pVTZ	CMA-0A MP2/ cc-pVTZ	CMA-2A MP2/ cc-pVTZ	Pure CCSD(T)/ cc-pVDZ	CMA-0A CCSD(T)/ cc-pVDZ	CMA-2A CCSD(T)/ cc-pVDZ
$\omega_1(a_1)$	2389.74	2431.69	2389.74	2389.74	2455.76	2389.74	2389.74
$\omega_2(a_1)$	1128.13	1137.03	1128.13	1128.13	1149.78	1128.14	1128.14
$\omega_3(b_2)$	2397.35	2442.41	2397.35	2397.35	2465.37	2397.35	2397.35

Table S427: CMA-0A and CMA-2A (ξ = 0.02) harmonic frequencies targeting CCSD(T)/cc-pVTZ for Level B = B3LYP/6-31G(2df,p).

	Pure B3LYP/ 6-31G(2df,p)	CMA-0A B3LYP/ 6-31G(2df,p)	CMA-2A B3LYP/ 6-31G(2df,p)
$\omega_1(a_1)$	2413.11	2389.74	2389.74
$\omega_2(a_1)$	1135.38	1128.14	1128.14
$\omega_3(b_2)$	2424.47	2397.35	2397.35

Table S428: Symmetrized, unnormalized natural internal coordinates for phosphino radical.

1	$r_{1,2} + r_{1,3}$
2	$r_{1,2} - r_{1,3}$
3	$\phi_{2,1,3}$

S4.108 nitrogen dioxide

Table S429: CCSD(T)/cc-pVTZ Optimum Cartesian coordinates (bohr)

1	O	0.00000000	−2.08779511	0.26863662
2	N	0.00000000	0.00000000	−0.61369664
3	O	0.00000000	2.08779511	0.26863662

Table S430: Reference [CCSD(T)/cc-pVTZ] CMA-0A and CMA-2A ($\xi = 0.02$) harmonic frequencies for Level B = MP2/cc-pVTZ and CCSD(T)/cc-pVDZ.

	Reference CCSD(T)/ cc-pVTZ	Pure MP2/ cc-pVTZ	CMA-0A MP2/ cc-pVTZ	CMA-2A MP2/ cc-pVTZ	Pure CCSD(T)/ cc-pVDZ	CMA-0A CCSD(T)/ cc-pVDZ	CMA-2A CCSD(T)/ cc-pVDZ
$\omega_1(a_1)$	1350.21	1349.16	1349.80	1350.21	1399.79	1350.20	1350.20
$\omega_2(a_1)$	758.21	766.81	758.94	758.21	765.08	758.22	758.22
$\omega_3(b_2)$	1679.70	1888.26	1679.70	1679.70	1756.92	1679.70	1679.70

Table S431: CMA-0A and CMA-2A ($\xi = 0.02$) harmonic frequencies targeting CCSD(T)/cc-pVTZ for Level B = B3LYP/6-31G(2*df*,*p*).

	Pure B3LYP/ 6-31G(2*df*,*p*)	CMA-0A B3LYP/ 6-31G(2*df*,*p*)	CMA-2A B3LYP/ 6-31G(2*df*,*p*)
$\omega_1(a_1)$	1380.18	1350.18	1350.18
$\omega_2(a_1)$	752.72	758.27	758.27
$\omega_3(b_2)$	1690.29	1679.70	1679.70

Table S432: Symmetrized, unnormalized natural internal coordinates for nitrogen dioxide.

1	$r_{1,2} + r_{2,3}$
2	$r_{1,2} - r_{2,3}$
3	$\phi_{1,2,3}$

S4.109 amino radical

Table S433: CCSD(T)/cc-pVTZ Optimum Cartesian coordinates (bohr)

1	H	0.00000000	−1.51058428	1.06481029
2	N	0.00000000	0.00000000	−0.15327241
3	H	0.00000000	1.51058428	1.06481029

Table S434: Reference [CCSD(T)/cc-pVTZ] CMA-0A and CMA-2A (ξ = 0.02) harmonic frequencies for Level B = MP2/cc-pVTZ and CCSD(T)/cc-pVDZ.

	Reference CCSD(T)/ cc-pVTZ	Pure MP2/ cc-pVTZ	CMA-0A MP2/ cc-pVTZ	CMA-2A MP2/ cc-pVTZ	Pure CCSD(T)/ cc-pVDZ	CMA-0A CCSD(T)/ cc-pVDZ	CMA-2A CCSD(T)/ cc-pVDZ
$\omega_1(a_1)$	3364.81	3388.84	3364.81	3364.81	3470.47	3364.74	3364.74
$\omega_2(a_1)$	1557.74	1551.54	1557.75	1557.75	1579.09	1557.90	1557.90
$\omega_3(b_2)$	3457.67	3491.44	3457.67	3457.67	3578.97	3457.67	3457.67

Table S435: CMA-0A and CMA-2A (ξ = 0.02) harmonic frequencies targeting CCSD(T)/cc-pVTZ for Level B = B3LYP/6-31G(2*df*,*p*).

	Pure B3LYP/ 6-31G(2*df*,*p*)	CMA-0A B3LYP/ 6-31G(2*df*,*p*)	CMA-2A B3LYP/ 6-31G(2*df*,*p*)
$\omega_1(a_1)$	3391.59	3364.79	3364.79
$\omega_2(a_1)$	1557.21	1557.77	1557.77
$\omega_3(b_2)$	3482.98	3457.67	3457.67

Table S436: Symmetrized, unnormalized natural internal coordinates for amino radical.

1	$r_{1,2} + r_{2,3}$
2	$r_{1,2} - r_{2,3}$
3	$\phi_{1,2,3}$

S4.110 ethyl radical

Table S437: CCSD(T)/cc-pVTZ Optimum Cartesian coordinates (bohr)

1	C	−0.00547866	−1.35440696	0.00000000
2	C	−0.01937298	1.46550160	0.00000000
3	H	1.92815108	−2.11168584	0.00000000
4	H	−0.94432912	−2.11610453	1.67223598
5	H	−0.94432912	−2.11610453	−1.67223598
6	H	0.12820569	2.51055499	−1.74949553
7	H	0.12820569	2.51055499	1.74949553

Table S438: Reference [CCSD(T)/cc-pVTZ] CMA-0A and CMA-2A (ξ = 0.02) harmonic frequencies for Level B = MP2/cc-pVTZ and CCSD(T)/cc-pVDZ.

	Reference CCSD(T)/ cc-pVTZ	Pure MP2/ cc-pVTZ	CMA-0A MP2/ cc-pVTZ	CMA-2A MP2/ cc-pVTZ	Pure CCSD(T)/ cc-pVDZ	CMA-0A CCSD(T)/ cc-pVDZ	CMA-2A CCSD(T)/ cc-pVDZ
$\omega_1(a^{'})$	3157.24	3175.87	3157.22	3157.22	3303.60	3157.22	3157.22
$\omega_2(a^{'})$	3064.92	3088.67	3064.22	3064.22	3212.51	3064.23	3064.23
$\omega_3(a^{'})$	2983.43	3005.26	2984.15	2984.15	3121.49	2984.06	2984.06
$\omega_4(a^{'})$	1493.37	1497.86	1493.27	1493.27	1511.21	1492.44	1492.44
$\omega_5(a^{'})$	1479.97	1485.11	1479.99	1479.99	1492.35	1480.37	1480.37
$\omega_6(a^{'})$	1403.42	1401.45	1403.49	1403.49	1421.63	1403.63	1403.63
$\omega_7(a^{'})$	1069.91	1072.27	1069.94	1069.94	1107.51	1070.48	1070.48
$\omega_8(a^{'})$	987.41	985.90	987.43	987.45	997.83	987.54	987.59
$\omega_9(a^{'})$	469.48	486.59	469.53	469.48	444.20	469.61	469.50
$\omega_{10}(a^{''})$	3260.35	3286.01	3260.35	3260.35	3417.56	3260.34	3260.34
$\omega_{11}(a^{''})$	3108.80	3135.14	3108.80	3108.80	3262.08	3108.77	3108.77
$\omega_{12}(a^{''})$	1492.29	1497.05	1492.27	1492.27	1502.59	1492.35	1492.35
$\omega_{13}(a^{''})$	1200.87	1202.11	1200.89	1200.91	1211.02	1200.89	1200.90
$\omega_{14}(a^{''})$	809.09	812.51	809.11	809.10	819.68	809.12	809.12
$\omega_{15}(a^{''})$	128.41	128.67	128.45	128.41	131.37	128.57	128.50

Table S439: CMA-0A and CMA-2A (ξ = 0.02) harmonic frequencies targeting CCSD(T)/cc-pVTZ for Level B = B3LYP/6-31G(2*df*,*p*).

	Pure B3LYP/ 6-31G(2*df*,*p*)	CMA-0A B3LYP/ 6-31G(2*df*,*p*)	CMA-2A B3LYP/ 6-31G(2*df*,*p*)
$\omega_1(a^{'})$	3171.44	3157.22	3157.22
$\omega_2(a^{'})$	3080.13	3064.75	3064.75
$\omega_3(a^{'})$	2998.42	2983.57	2983.57
$\omega_4(a^{'})$	1486.48	1490.60	1490.60
$\omega_5(a^{'})$	1470.19	1482.33	1482.33
$\omega_6(a^{'})$	1406.28	1403.50	1403.71
$\omega_7(a^{'})$	1057.73	1070.41	1070.15
$\omega_8(a^{'})$	988.33	987.53	987.54
$\omega_9(a^{'})$	475.00	469.51	469.48
$\omega_{10}(a^{''})$	3277.99	3260.34	3260.34
$\omega_{11}(a^{''})$	3124.37	3108.79	3108.80
$\omega_{12}(a^{''})$	1488.96	1492.24	1492.26
$\omega_{13}(a^{''})$	1200.57	1200.89	1200.89
$\omega_{14}(a^{''})$	810.15	809.15	809.15
$\omega_{15}(a^{''})$	117.82	128.71	128.41

Table S440: Symmetrized, unnormalized natural internal coordinates for ethyl radical.

1	$r_{1,2}$
2	$r_{2,6} + r_{2,7}$
3	$r_{2,6} - r_{2,7}$
4	$r_{1,4} + r_{1,5} + r_{1,3}$
5	$-r_{1,4} - r_{1,5} + 2r_{1,3}$
6	$r_{1,4} - r_{1,5}$
7	$2\phi_{6,2,7} - \phi_{6,2,1} - \phi_{7,2,1}$
8	$\phi_{6,2,1} - \phi_{7,2,1}$
9	$\phi_{2,1,3} + \phi_{2,1,4} + \phi_{2,1,5} - \phi_{4,1,5} - \phi_{4,1,3} - \phi_{5,1,3}$
10	$2\phi_{2,1,3} - \phi_{2,1,4} - \phi_{2,1,5}$
11	$\phi_{2,1,4} - \phi_{2,1,5}$
12	$2\phi_{4,1,5} - \phi_{4,1,3} - \phi_{5,1,3}$
13	$\phi_{4,1,3} - \phi_{5,1,3}$
14	$\tau_{6,2,1,3} + \tau_{6,2,1,4} + \tau_{6,2,1,5} + \tau_{7,2,1,3} + \tau_{7,2,1,4} + \tau_{7,2,1,5}$
15	$\gamma_{1,2,6,7}$

S4.111 *tert*−butyl radical

Table S441: CCSD(T)/cc-pVTZ Optimum Cartesian coordinates (bohr)

1	C	−0.32334039	−0.00000205	0.00000000
2	C	0.06114007	−1.40087768	−2.42638089
3	C	0.06102027	2.80175965	0.00000000
4	C	0.06114007	−1.40087768	2.42638089
5	H	2.08693792	−1.64409780	−2.84744106
6	H	−0.76540572	−0.38822110	−4.02548203
7	H	−0.76556895	−3.29200016	−2.34898777
8	H	2.08679900	3.28810311	0.00000000
9	H	−0.76564669	3.68025423	1.67650059
10	H	−0.76564669	3.68025423	−1.67650059
11	H	2.08693792	−1.64409780	2.84744106
12	H	−0.76556895	−3.29200016	2.34898777
13	H	−0.76540572	−0.38822110	4.02548203

Table S442: Reference [CCSD(T)/cc-pVTZ] CMA-0A and CMA-2A ($\xi = 0.02$) harmonic frequencies for Level B = MP2/cc-pVTZ and CCSD(T)/cc-pVDZ.

	Reference CCSD(T)/ cc-pVTZ	Pure MP2/ cc-pVTZ	CMA-0A MP2/ cc-pVTZ	CMA-2A MP2/ cc-pVTZ	Pure CCSD(T)/ cc-pVDZ	CMA-0A CCSD(T)/ cc-pVDZ	CMA-2A CCSD(T)/ cc-pVDZ
$\omega_1(a_1)$	3054.29	3074.49	3053.60	3053.60	3196.48	3053.54	3053.54
$\omega_2(a_1)$	2954.07	2973.89	2954.77	2954.77	3091.82	2954.77	2954.77
$\omega_3(a_1)$	1496.35	1498.88	1496.33	1496.33	1507.59	1496.27	1496.27
$\omega_4(a_1)$	1419.84	1412.99	1419.86	1419.86	1440.01	1419.95	1419.95
$\omega_5(a_1)$	1105.28	1098.54	1105.28	1105.28	1117.58	1105.35	1105.35
$\omega_6(a_1)$	767.88	765.54	767.91	767.91	796.17	768.03	768.03
$\omega_7(a_1)$	261.22	256.88	261.24	261.23	268.92	261.25	261.25
$\omega_8(a_2)$	3098.03	3124.14	3098.02	3098.02	3247.15	3097.98	3097.98
$\omega_9(a_2)$	1474.27	1475.98	1474.26	1474.26	1484.23	1474.33	1474.34
$\omega_{10}(a_2)$	964.11	963.08	964.13	964.13	976.62	964.13	964.14
$\omega_{11}(a_2)$	132.72	133.74	132.73	132.72	143.77	132.76	132.72
$\omega_{12a}(e)$	3102.34	3127.31	3102.34	3102.34	3251.41	3102.30	3102.30
$\omega_{12b}(e)$	3102.32	3127.25	3102.32	3102.32	3251.35	3102.29	3102.29
$\omega_{13a}(e)$	3052.84	3074.86	3052.20	3052.20	3195.34	3052.12	3052.12
$\omega_{13b}(e)$	3052.83	3074.85	3052.20	3052.20	3195.32	3052.12	3052.12
$\omega_{14a}(e)$	2947.91	2970.82	2948.56	2948.56	3084.69	2948.55	2948.55
$\omega_{14b}(e)$	2947.90	2970.74	2948.55	2948.55	3084.61	2948.54	2948.54
$\omega_{15a}(e)$	1497.74	1500.48	1497.70	1497.70	1508.83	1497.21	1497.21
$\omega_{15b}(e)$	1497.72	1500.47	1497.70	1497.70	1508.82	1497.21	1497.21
$\omega_{16a}(e)$	1477.70	1479.53	1477.66	1477.66	1489.29	1477.62	1477.62
$\omega_{16b}(e)$	1477.68	1479.52	1477.66	1477.66	1489.28	1477.61	1477.61
$\omega_{17a}(e)$	1397.76	1390.34	1397.79	1397.79	1424.52	1396.71	1396.71
$\omega_{17b}(e)$	1397.75	1390.31	1397.79	1397.79	1424.50	1396.71	1396.71
$\omega_{18a}(e)$	1303.90	1299.35	1303.91	1303.91	1334.70	1305.22	1305.49
$\omega_{18b}(e)$	1303.88	1299.34	1303.90	1303.90	1334.69	1305.21	1305.48
$\omega_{19a}(e)$	1013.95	1013.43	1013.97	1013.97	1034.95	1014.67	1014.32
$\omega_{19b}(e)$	1013.92	1013.43	1013.95	1013.95	1034.94	1014.65	1014.30
$\omega_{20a}(e)$	933.83	929.76	933.85	933.85	946.09	934.14	934.14
$\omega_{20b}(e)$	933.80	929.75	933.82	933.82	946.09	934.11	934.11
$\omega_{21a}(e)$	367.07	366.69	367.06	367.06	378.49	367.04	367.09
$\omega_{21b}(e)$	367.05	366.68	367.05	367.05	378.49	367.03	367.08
$\omega_{22a}(e)$	147.75	149.43	147.78	147.78	152.02	147.91	147.78
$\omega_{22b}(e)$	147.62	149.41	147.65	147.65	152.00	147.77	147.65

Table S443: CMA-0A and CMA-2A (ξ = 0.02) harmonic frequencies targeting CCSD(T)/cc-pVTZ for Level B = B3LYP/6-31G(2df,p).

	Pure B3LYP/ 6-31G(2df,p)	CMA-0A B3LYP/ 6-31G(2df,p)	CMA-2A B3LYP/ 6-31G(2df,p)
$\omega_1(a_1)$	3068.73	3054.14	3054.14
$\omega_2(a_1)$	2969.10	2954.20	2954.20
$\omega_3(a_1)$	1494.67	1496.19	1496.19
$\omega_4(a_1)$	1428.47	1419.95	1419.95
$\omega_5(a_1)$	1112.95	1105.36	1105.37
$\omega_6(a_1)$	762.60	767.97	767.98
$\omega_7(a_1)$	280.34	261.32	261.25
$\omega_8(a_2)$	3111.07	3098.02	3098.02
$\omega_9(a_2)$	1472.33	1474.23	1474.23
$\omega_{10}(a_2)$	972.55	964.18	964.18
$\omega_{11}(a_2)$	141.71	132.84	132.81
$\omega_{12a}(e)$	3118.47	3102.31	3102.31
$\omega_{12b}(e)$	3114.28	3102.28	3102.28
$\omega_{13a}(e)$	3062.42	3052.77	3052.77
$\omega_{13b}(e)$	3060.89	3052.62	3052.62
$\omega_{14a}(e)$	2964.69	2948.09	2948.09
$\omega_{14b}(e)$	2954.20	2947.93	2947.93
$\omega_{15a}(e)$	1499.34	1497.31	1497.31
$\omega_{15b}(e)$	1493.82	1497.29	1497.29
$\omega_{16a}(e)$	1477.36	1477.96	1477.96
$\omega_{16b}(e)$	1475.66	1477.70	1477.70
$\omega_{17a}(e)$	1403.87	1396.68	1397.63
$\omega_{17b}(e)$	1398.27	1396.35	1396.68
$\omega_{18a}(e)$	1296.71	1305.06	1304.90
$\omega_{18b}(e)$	1295.84	1304.87	1303.67
$\omega_{19a}(e)$	1013.36	1014.79	1014.79
$\omega_{19b}(e)$	1012.05	1014.76	1014.76
$\omega_{20a}(e)$	942.78	934.01	934.02
$\omega_{20b}(e)$	937.09	933.86	933.86
$\omega_{21a}(e)$	376.29	367.11	367.11
$\omega_{21b}(e)$	374.55	367.05	367.11
$\omega_{22a}(e)$	152.80	148.13	147.78
$\omega_{22b}(e)$	131.09	147.80	147.73

Table S444: Symmetrized, unnormalized natural internal coordinates for *tert*−butyl radical.

1	$r_{1,2} + r_{1,3} + r_{1,4}$
2	$2r_{1,2} - r_{1,3} - r_{1,4}$
3	$r_{1,3} - r_{1,4}$
4	$r_{2,5} + r_{2,6} + r_{2,7} + r_{3,8} + r_{3,9} + r_{3,10} + r_{4,11} + r_{4,12} + r_{4,13}$
5	$2r_{2,5} + 2r_{2,6} + 2r_{2,7} - r_{3,8} - r_{3,9} - r_{3,10} - r_{4,11} - r_{4,12} - r_{4,13}$
6	$r_{3,8} + r_{3,9} + r_{3,10} - r_{4,11} - r_{4,12} - r_{4,13}$
7	$2r_{2,5} - r_{2,6} - r_{2,7} + 2r_{3,8} - r_{3,9} - r_{3,10} + 2r_{4,11} - r_{4,12} - r_{4,13}$
8	$4r_{2,5} - 2r_{2,6} - 2r_{2,7} - 2r_{3,8} + r_{3,9} + r_{3,10} - 2r_{4,11} + r_{4,12} + r_{4,13}$
9	$2r_{3,8} - r_{3,9} - r_{3,10} - 2r_{4,11} + r_{4,12} + r_{4,13}$
10	$r_{2,6} - r_{2,7} + r_{3,9} - r_{3,10} + r_{4,12} - r_{4,13}$
11	$2r_{2,6} - 2r_{2,7} - r_{3,9} + r_{3,10} - r_{4,12} + r_{4,13}$
12	$r_{3,9} - r_{3,10} - r_{4,12} + r_{4,13}$
13	$2\phi_{3,1,4} - \phi_{2,1,3} - \phi_{2,1,4}$
14	$\phi_{2,1,3} - \phi_{2,1,4}$
15	$\phi_{1,2,5} + \phi_{1,2,6} + \phi_{1,2,7} - \phi_{6,2,7} - \phi_{5,2,6} - \phi_{5,2,7} + \phi_{1,3,8} + \phi_{1,3,9} + \phi_{1,3,10} - \phi_{9,3,10}$ $- \phi_{8,3,9} - \phi_{8,3,10} + \phi_{1,4,11} + \phi_{1,4,12} + \phi_{1,4,13} - \phi_{12,4,13} - \phi_{11,4,12} - \phi_{11,4,13}$
16	$2\phi_{1,2,5} + 2\phi_{1,2,6} + 2\phi_{1,2,7} - 2\phi_{6,2,7} - 2\phi_{5,2,6} - 2\phi_{5,2,7} - \phi_{1,3,8} - \phi_{1,3,9} - \phi_{1,3,10} + \phi_{9,3,10}$ $+ \phi_{8,3,9} + \phi_{8,3,10} - \phi_{1,4,11} - \phi_{1,4,12} - \phi_{1,4,13} + \phi_{12,4,13} + \phi_{11,4,12} + \phi_{11,4,13}$
17	$\phi_{1,3,8} + \phi_{1,3,9} + \phi_{1,3,10} - \phi_{9,3,10} - \phi_{8,3,9} - \phi_{8,3,10} - \phi_{1,4,11} - \phi_{1,4,12} - \phi_{1,4,13} + \phi_{12,4,13}$ $+ \phi_{11,4,12} + \phi_{11,4,13}$
18	$2\phi_{1,2,5} - \phi_{1,2,6} - \phi_{1,2,7} + 2\phi_{1,3,8} - \phi_{1,3,9} - \phi_{1,3,10} + 2\phi_{1,4,11} - \phi_{1,4,12} - \phi_{1,4,13}$
19	$4\phi_{1,2,5} - 2\phi_{1,2,6} - 2\phi_{1,2,7} - 2\phi_{1,3,8} + \phi_{1,3,9} + \phi_{1,3,10} - 2\phi_{1,4,11} + \phi_{1,4,12} + \phi_{1,4,13}$
20	$2\phi_{1,3,8} - \phi_{1,3,9} - \phi_{1,3,10} - 2\phi_{1,4,11} + \phi_{1,4,12} + \phi_{1,4,13}$
21	$\phi_{1,2,6} - \phi_{1,2,7} + \phi_{1,3,9} - \phi_{1,3,10} + \phi_{1,4,12} - \phi_{1,4,13}$
22	$2\phi_{1,2,6} - 2\phi_{1,2,7} - \phi_{1,3,9} + \phi_{1,3,10} - \phi_{1,4,12} + \phi_{1,4,13}$
23	$\phi_{1,3,9} - \phi_{1,3,10} - \phi_{1,4,12} + \phi_{1,4,13}$
24	$2\phi_{6,2,7} - \phi_{5,2,6} - \phi_{5,2,7} + 2\phi_{9,3,10} - \phi_{8,3,9} - \phi_{8,3,10} + 2\phi_{12,4,13} - \phi_{11,4,12} - \phi_{11,4,13}$
25	$4\phi_{6,2,7} - 2\phi_{5,2,6} - 2\phi_{5,2,7} - 2\phi_{9,3,10} + \phi_{8,3,9} + \phi_{8,3,10} - 2\phi_{12,4,13} + \phi_{11,4,12} + \phi_{11,4,13}$
26	$2\phi_{9,3,10} - \phi_{8,3,9} - \phi_{8,3,10} - 2\phi_{12,4,13} + \phi_{11,4,12} + \phi_{11,4,13}$
27	$\phi_{5,2,6} - \phi_{5,2,7} + \phi_{8,3,9} - \phi_{8,3,10} + \phi_{11,4,12} - \phi_{11,4,13}$
28	$2\phi_{5,2,6} - 2\phi_{5,2,7} - \phi_{8,3,9} + \phi_{8,3,10} - \phi_{11,4,12} + \phi_{11,4,13}$
29	$\phi_{8,3,9} - \phi_{8,3,10} - \phi_{11,4,12} + \phi_{11,4,13}$
30	$\tau_{5,2,1,3} + \tau_{5,2,1,4} + \tau_{6,2,1,3} + \tau_{6,2,1,4} + \tau_{7,2,1,3} + \tau_{7,2,1,4} + \tau_{8,3,1,2} + \tau_{8,3,1,4} + \tau_{9,3,1,2} + \tau_{9,3,1,4}$ $+ \tau_{10,3,1,2} + \tau_{10,3,1,4} + \tau_{11,4,1,2} + \tau_{11,4,1,3} + \tau_{12,4,1,2} + \tau_{12,4,1,3} + \tau_{13,4,1,2} + \tau_{13,4,1,3}$
31	$2\tau_{5,2,1,3} + 2\tau_{5,2,1,4} + 2\tau_{6,2,1,3} + 2\tau_{6,2,1,4} + 2\tau_{7,2,1,3} + 2\tau_{7,2,1,4} - \tau_{8,3,1,2} - \tau_{8,3,1,4} - \tau_{9,3,1,2} - \tau_{9,3,1,4}$ $- \tau_{10,3,1,2} - \tau_{10,3,1,4} - \tau_{11,4,1,2} - \tau_{11,4,1,3} - \tau_{12,4,1,2} - \tau_{12,4,1,3} - \tau_{13,4,1,2} - \tau_{13,4,1,3}$
32	$\tau_{8,3,1,2} + \tau_{8,3,1,4} + \tau_{9,3,1,2} + \tau_{9,3,1,4} + \tau_{10,3,1,2} + \tau_{10,3,1,4} - \tau_{11,4,1,2} - \tau_{11,4,1,3} - \tau_{12,4,1,2} - \tau_{12,4,1,3}$ $- \tau_{13,4,1,2} - \tau_{13,4,1,3}$
33	$\gamma_{2,1,3,4} + \gamma_{3,1,4,2} + \gamma_{4,1,2,3}$
